# Carbon–nitrogen transmutation in polycyclic arenol skeletons to access *N*-heteroarenes

**DOI:** 10.1038/s41467-024-48265-6

**Published:** 2024-05-04

**Authors:** Hong Lu, Yu Zhang, Xiu-Hong Wang, Ran Zhang, Peng-Fei Xu, Hao Wei

**Affiliations:** 1 Key Laboratory of Synthetic and Natural Functional Molecule of the Ministry of Education, College of Chemistry & Materials Science, Northwest University, Xi’an, 710069 China; 2 State Key Laboratory of Applied Organic Chemistry, College of Chemistry and Chemical Engineering, Lanzhou University, Lanzhou, 730000 China

**Keywords:** Synthetic chemistry methodology, Synthetic chemistry methodology, Catalyst synthesis

## Abstract

Developing skeletal editing tools is not a trivial task, and realizing the corresponding single-atom transmutation in a ring system without altering the ring size is even more challenging. Here, we introduce a skeletal editing strategy that enables polycyclic arenols, a highly prevalent motif in bioactive molecules, to be readily converted into *N*-heteroarenes through carbon–nitrogen transmutation. The reaction features selective nitrogen insertion into the C–C bond of the arenol frameworks by azidative dearomatization and aryl migration, followed by ring-opening, and ring-closing (ANRORC) to achieve carbon-to-nitrogen transmutation in the aromatic framework of the arenol. Using widely available arenols as *N*-heteroarene precursors, this alternative approach allows the streamlined assembly of complex polycyclic heteroaromatics with broad functional group tolerance. Finally, pertinent transformations of the products, including synthesis complex biheteroarene skeletons, were conducted and exhibited significant potential in materials chemistry.

## Abstract (web summary)

Developing skeletal editing tools, especially to realize the single-atom transmutation in a ring system without altering the ring size is challenging. Here, the authors introduce a skeletal editing strategy that enables polycyclic arenols to be readily converted into *N*-heteroarenes through carbon–nitrogen transmutation.

## Introduction

**Fig. 1:**
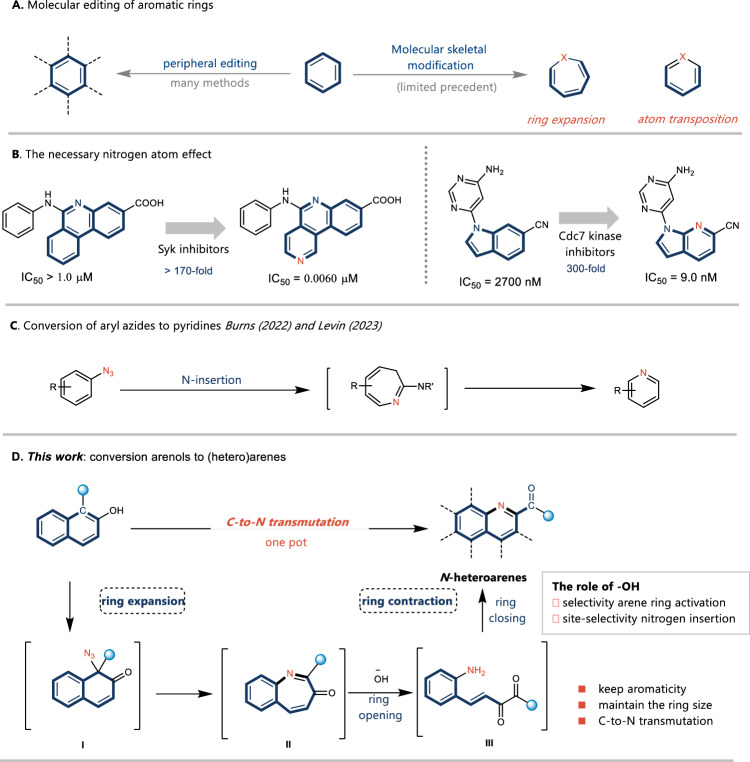
Examples of carbon–nitrogen transmutation and our reaction design. **A** Molecular editing of aromatic rings. **B** Examples of the necessary nitrogen atom effect. **C** Conversion of aryl azides to pyridines. **D** This study.

Organic synthesis underpins the evolution and advancement of broad areas of science, from materials to medicine. Arenes are among the most widely used rings in medicine and natural products. The functionalization of arenes is a particularly attractive tool for the production of pharmaceuticals, natural products, and molecular materials^1–4^. However, their application has so far been largely focused on C–H functionalization chemistry (peripheral editing), and the precise modification of the aromatic ring skeleton remains largely unexplored (Fig. 1A)^5–7^. Single-atom skeletal editing has become an extremely powerful tool for straightforwardly modifying the core skeleton of organic molecules. Recently, a limited number of single–atom insertion or deletion reactions have been developed to reshape the underlying molecular skeletons^8–21^. However, the direct modification of valuable core structures by replacing one atom in a ring system without changing the ring size and aromaticity remains elusive^22–31^, although it has been recognized as a highly desirable transformation.

One of the most studied among *N*-heteroarenes are pyridines, which serve as a bioisosteric replacement of benzene counterparts within the parent molecules^32–34^. The replacement of carbon with nitrogen in aromatic ring systems can have several important effects on the molecular and physicochemical properties relevant to multiparameter optimization (Fig. 1B). This necessary nitrogen atom effect is a versatile high-impact design element for multiparameter optimization, which has been shown to improve various key pharmacological parameters^35^. Recently, Burns and Levin independently reported groundbreaking methods for the direct conversion of arenes to pyridines via nitrene internalization (Fig. 1C)^36,37^. In these process, additional steps for installation and isolation of aryl azides are always requried, which indicated that a selective, and straightforward transformation of diverse arenes into *N*-heteroarenes remains an important goal^38^.

A key challenge in this transformation is the stability of the aromatic compounds. Our design overcomes this intrinsic challenge using arenols as substrates. Dearomatization of arenols disrupts the stability of the aromatic ring and promotes subsequent skeletal transformations^39–48^. Arenol can also act as a selectivity controlling element in site-selective skeletal editing. Our group’s recent work employing this dearomative strategy to promote ring expansion of arenols inspired us to continue investigating this strategy to more complex skeletal editing transformation^49^. In this work, we describe the direct carbon-to-nitrogen transmutations in arenols. This reaction involves two stages: ring expansion and contraction (Fig. 1D). In the first stage, the insertion of nitrogen atoms is achieved by azidative dearomatization of an arenol and intramolecular aryl migration. In the second stage, a carbon atom moves out of the ring skeleton through ring-opening, and ring-closing (ANRORC), which ultimately furnishes desired carbon–nitrogen transmutation in polycyclic arenol skeletons^50–52^.

## Results

### Reaction optimization

We began our investigation using methylphenanthren-9-ol **1a** as the reaction partner. (PhO)_2_POOH, NBS, and N(*n*Bu)_4_N_3_ were employed as reagents for the in situ formation of the azido ketone intermediate (see Supplementary Information, section 2.2.2). For optimization, we observed the formation of desired product **1** in 80% yield using FeBr_2_ and Cy_3_PO as an effective catalyst–ligand combination in PhCl (Table 1, entry 1). A control experiment revealed that an iron salt was essential for obtaining the desired product (Table 1, entry 2). Other iron salts, including FeCl_2_, Fe(OTf)_2_, Fe(OAc)_2_, and Fe(acac)_3_, exhibited lower efficiency than inexpensive FeBr_2_ (Table 1, entries 3 − 6). Furthermore, when other established metal nitrenoid formation catalysts, including copper, rhodium, cobalt, and ruthenium, were used, the desired product was not obtained satisfyingly (Table 1, entries 7–10)^53,54^. Further optimization showed that this reaction was slightly improved using Cy_3_PO (Table 1, entry 11). The reaction appeared to be less sensitive to solvents, as replacing the PhCl with either toluene or THF furnished **1** in good yield (Table 1, entries 12 and 13). The yield decreased slightly when 10 mol% FeBr_2_ and 15 mol% Cy_3_PO were used (Table 1, entry 14).

**Table 1:** Screening of reaction conditions.^a^


Entry	Variation from ‘*standard conditions*’	Yield (%)^b^
1	None	80 (61)^c^
2	w/o FeBr_2_	trace
3	FeCl_2_ instead of FeBr_2_	71
4	Fe(OTf)_2_ instead of FeBr_2_	75
5	Fe(OAc)_2_ instead of FeBr_2_	32
6	Fe(acac)_3_ instead of FeBr_2_	10
7	Cu(OTf)_2_ instead of FeBr_2_	<10
8	[[CH_3_(CH_2_)_6_CO_2_]_2_Rh]_2_ instead of FeBr_2_	34
9	TMOPP-Co instead of FeBr_2_	trace
10	Ru_3_(CO)_13_ instead of FeBr_2_	<10
11	w/o Cy_3_PO	70
12	In toluene	75
13	In THF	70
14	10 mol% FeBr_2_ and 15 mol% Cy_3_PO instead	61

^a^Unless otherwise specified, all reactions were carried out using **1a** (0.1 mmol), NBS (0.18 mmol), N(*n*Bu_4_)N_3_ (0.3 mmol), (PhO)_2_POOH (0.075 mmol), FeBr_2_ (0.015 mmol), and Cy_3_PO (0.02 mmol) in PhCl (1.0 mL) at 100 °C for 36 h.

^b^Isolated yields after chromatography.

^c^Scale-up reaction by using 1.0 mmol of **1a**.

### Substrate scope

**Fig. 2:**
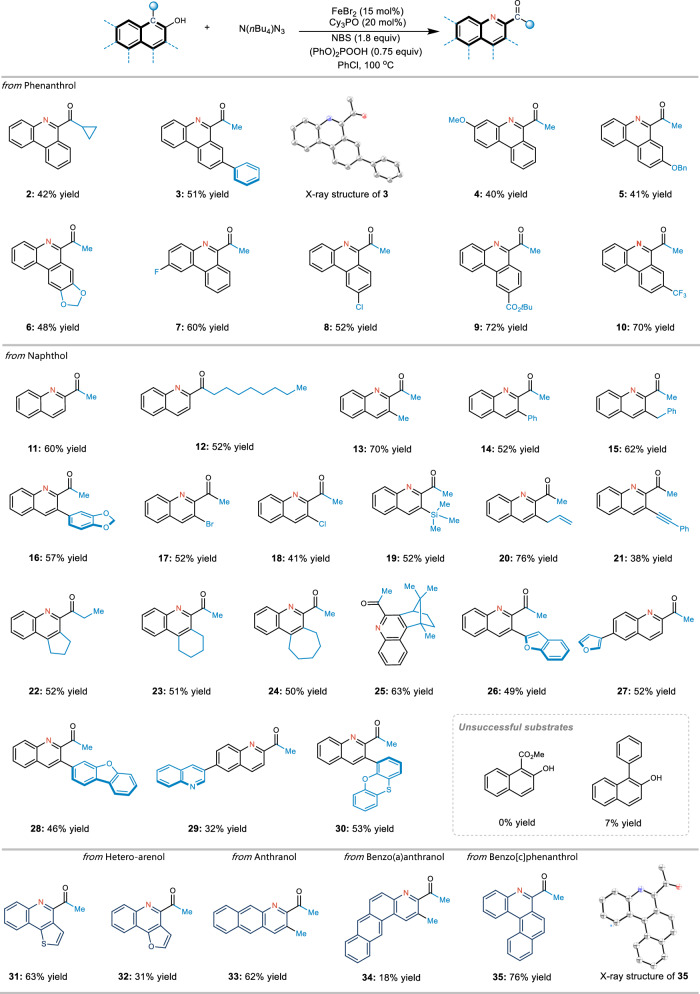
Substrate scope of the carbon–nitrogen transmutation in polycyclic arenol. ^a,b a^Isolated yields after chromatography are shown. ^b^ Reaction conditions: substrate (0.1 mmol), NBS (0.18 mmol), N(*n*Bu_4_)N_3_ (0.3 mmol), (PhO)_2_POOH (0.075 mmol), FeBr_2_ (0.015 mmol), and Cy_3_PO (0.02 mmol) in PhCl (1.0 mL) at 100 °C for 36 h.

Considering the optimal reaction conditions, the substrate scope was determined (Fig. 2). Various arenols, including phenanthrol (**1**–**10**), naphthol (**11**–**30**), anthranol (**33**) benzo(*a*)anthranol (**34**), and benzo[*c*]phenanthrol (**35**), can effectively undergo the desired carbon–nitrogen transmutation. Both electron-rich and electron-deficient aromatic substrates were suitable for the process. It was found that the substituents at the *ortho* positions of the arenol are significant. When the substituent was an alkyl group, the corresponding arenols underwent atom transmutation smoothly in moderate-to-good yield and chemoselectivity. The presence of a phenyl group or an electron-withdrawing group such as CO_2_Me at the *ortho*-position can inhibit this reaction. However, various functional groups, such as ether (**4** and **5**), acetals (**6** and **16**), aryl halides (**7,** **8,** **17** and **18**), esters (**9**), trifluoromethyl (**10**), trimethylsilyl (TMS) (**19**), alkenes (**20**), and alkynes (**21**), were tolerated in this transformation. In addition, several naphthyl-fused rings (**22**–**25**) were suitable substrates, affording the desired products in moderate-to-good yields. Heterocyclic moieties such as benzofuran (**26**), furan (**27**), dibenzofuran (**28**), quinoline (**29**), and phenoxathiine (**30**) were also compatible. Moreover, fused heteroarenols such as naphtho[1,2-*b*]thiophene (**31**) and naphtho[1,2-*b*]furan (**32**) can be incorporated, providing pharmaceutically interesting fused-ring skeletons that are non-trivial to prepare. The structures of **3** and **35** were identified using X-ray crystallography.

### Synthetic utility

**Fig. 3:**
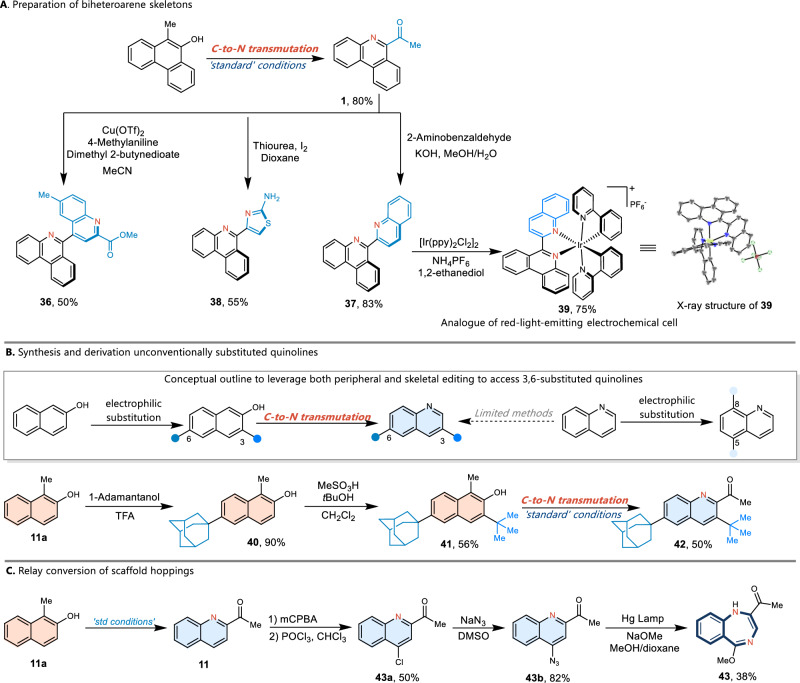
Application potential of carbon–nitrogen transmutation. **A** Applications that allow access to complex biheteroarene skeletons. **B** Preparation of unconventionally 3,6-substituted quinolines. **C** Sequential skeletal editing transformations of naphthol.

The successful development of the atom transmutation protocol offers a rapid and modular approach to access complex biheteroarene skeleton, a common structural motif found in bioactive compounds (Fig. 3A). Compound **37** could be easily transformed to the iridium complexes **39**, which could serve as the red-light-emitting electrochemical cell^55,56^. Next, the synthetic versatility of the C-to-N transmutation was demonstrated through the preparation of 3,6-disubstituted quinolines **42**, which could not be obtained from directly electrophilic substitution of quinolines (Fig. 3B). Specifically, the successful development of the carbon–nitrogen transmutation offers exciting opportunities to devise more complex skeletal editing transformations via combinations of atom insertions and deletions. Benzo[1,4]diazepine **43** can be accessed through a C–H azidation and aryl migration sequence from **11**, presently the formal carbon deletion and two nitrogen insertion products of starting naphthol **11a** (Fig. 3C).

### Mechanistic considerations

**Fig. 4:**
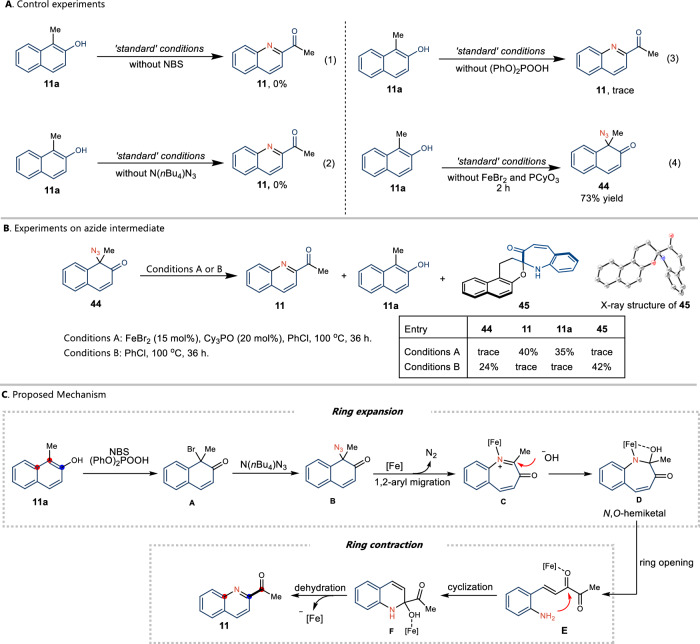
Mechanistic studies. **A** Control experiments. **B** Control experiments using azide intermediate showing that Fe catalyst is essential for aryl migration and ring contraction process. **C** Proposed mechanism.

To elucidate the mechanism of this transformation, series control experiments were first conducted. The reactions without addition of NBS or N(*n*Bu_4_)N_3_ failed to produce the desired product (Fig. 4A, equations 1 and 2). And trace amount of **11** was observed when (PhO)_2_POOH was absent from the reaction mixture (Fig. 4A, equation 3). It’s worth noting that azide ketone **44** could be isolated in 73% yield in the absense of Fe catalyst after 2 h (Fig. 4A, equation 4). These results indicated that the proposed azidative dearomatization of arenol might be involved (Fig. 1D)^57^. The azide ketone **44** was then tested with and without the addition of the Fe catalyst (Fig. 4B). It was found that the desired product **11** was formed in 40% yield, and 35% yield of **11a** was isolated in presence of Fe catalyst, which demonstrated that the proposed azidation is a reversible process via successive single-electron transfer (SET) from Fe(II) to eliminate azide^58^. On the contrary, only trace amount of product **11** and **11a** was observed without Fe catalyst. And a byproduct **45** was detected in 42% yield and recovered **44** in 24%^59^. These results revealed that the Fe catalyst is not only involved in aryl migration, but is also essential for the ring contraction process^60^.

Based on the literature reports and our observations, a plausible mechanism is proposed (Fig. 4C). Initially, the *N*-bromosuccinimide-mediated dearomatization of the corresponding naphthol of **11a** afforded the brominated ketone intermediate **A**, which subsequently reacted with N(*n*Bu)_4_N_3_ to generate azido ketone **B**. Then iron salt reacted with **B** can form metal−nitrene species, which would then undergo 1,2-aryl migration to form ring expansion intermediate **C**. Subsequently, addition of hydroxide anion to the imine group of **C** induces *N*,*O*-hemiketal **D**. The collapse of **D** with assistance of iron salt produces ring-opening amino-ketone intermediate **E**^61,62^, which undergoes re-cyclization and dehydration to form stable benzoquinoline **11** and release Fe catalyst.

In conclusion, this study proposed a unique strategy that enables straightforward carbon-to-nitrogen transmutations in arenols through a one-pot ring expansion-contraction sequence. This site-selective atom transformation is based on sequentially combining three transformations in one pot using aryl migration and imine transposition as key steps and opens new opportunities for single-atom skeletal edit design. Further preparation of complex biheteroarene skeleton and unconventionally substituted quinoline highlights the potential of this study. This provides an alternative for the development of *N*-heteroarenes and demonstrates significant potential in materials chemistry.

## Methods

### General condition for carbon–nitrogen transmutation

Substrate (0.1 mmol), NBS (0.12 mmol), N(*n*Bu_4_)N_3_ (0.2 mmol), FeBr_2_ (0.015 mmol), tricyclohexylphosphine oxide (0.02 mmol), (PhO)_2_POOH (0.05 mmol), and PhCl (1.0 mL) was successively added to an 10 mL sealed tube equipped with a Teflon-coated magnetic stir bar. The tube then was sealed with a Teflon screw cap and placed on a hotplate pre-heated to 100 °C with vigorous stirring. After 18 h, the reaction was cooled to room temperature and another portion of NBS (0.06 mmol, 0.6 equiv), N(*n*Bu_4_)N_3 _(0.1 mmol) and (PhO)_2_POOH (0.025 mmol) was successively added to the sealed tube. The tube then reacted at 100 °C with vigorous stirring. After 18 h, the reaction was cooled to room temperature. The solvent was evaporated and the residue was directly purified by flash column chromatography on silica gel (petroleum ether/ethyl acetate = 20/1) to give the desired products.

### Supplementary information

Supplementary Information

Peer Review File

## Data Availability

Data relating to the optimization studies, mechanistic studies, general methods, and the characterization data of materials and products, are available in the Supplementary Information. Crystallographic data for the structures reported in this article have been deposited at the Cambridge Crystallographic Data Center, under deposition numbers CCDC 2285580 (**3**), 2285872 (**35**), 2310917 (**39**) and 2308629 (**45**). Copies of the data can be obtained free of charge via https://www.ccdc.cam.ac.uk/structures. All data are available from the corresponding author upon request.

## References

[1] Engle KM, Mei T-S, Wasa M, Yu J-Q (2012). Weak coordination as a powerful means for developing broadly useful C–H functionalization reactions. Acc. Chem. Res..

[2] Neufeldt SR, Sanford MS (2012). Controlling site selectivity in palladiumcatalyzed C–H bond functionalization. Acc. Chem. Res..

[3] Wencel-Delord J, Glorius F (2013). C–H bond activation enables the rapid construction and late-stage diversification of functional molecules. Nat. Chem..

[4] Abrams DJ, Provencher PA, Sorensen EJ (2018). Recent applications of C–H functionalization in complex natural product synthesis. Chem. Soc. Rev..

[5] Jurczyk J (2022). Single-Atom Logic for Heterocycle Editing. Nat. Synth..

[6] Liu, F., Anand, L. & Szostak, M. Diversification of indoles and pyrroles by molecular editing: New frontiers in heterocycle-to-heterocycle transmutation. *Chem. Eur. J*. **29**, e202300096 (2023). 10.1002/chem.202300096 PMC10192006 36730110

[7] Zhaozhong L, Paramasivam S, Yongquan N, Yong W, Xihe B (2023). Skeletal editing of (hetero)arenes using carbenes. Chem. Eur. J..

[8] Roque JB, Kuroda Y, Göttemann LT, Sarpong R (2018). Deconstructive diversification of cyclic amines. Nature.

[9] Dherange BD, Kelly PQ, Liles JP, Sigman MS, Levin MD (2021). Carbon atom insertion into pyrroles and indoles promoted by chlorodiazirines. J. Am. Chem. Soc..

[10] Jurczyk J (2021). Photomediated ring contraction of saturated heterocycles. Science.

[11] Kennedy SH, Dherange BD, Berger KJ, Levin MD (2021). Skeletal editing through direct nitrogen deletion of secondary amines. Nature.

[12] Woo J (2022). Scaffold hopping by net photochemical carbon deletion of azaarenes. Science.

[13] Bartholomew GL, Carpaneto F, Sarpong R (2022). Skeletal editing of pyrimidines to pyrazoles by formal carbon deletion. J. Am. Chem. Soc..

[14] Reisenbauer JC, Green O, Franchino A, Finkelstein P, Morandi B (2022). Late-stage diversification of indole skeletons through nitrogen atom insertion. Science.

[15] Liu S, Cheng X (2022). Insertion of ammonia into alkenes to build aromatic N-heterocycles. Nat. Commun..

[16] Kelly PQ, Filatov AS, Levin MD (2022). A synthetic cycle for heteroarene synthesis by nitride insertion. Angew. Chem. Int. Ed..

[17] Wang J, Lu H, He Y, Jing C, Wei H (2022). Cobalt-catalyzed nitrogen atom insertion in arylcycloalkenes. J. Am. Chem. Soc..

[18] Finkelstein P (2023). Nitrogen atom insertion into indenes to access isoquinolines. Chem. Sci..

[19] Wight BA (2023). Skeletal editing approach to bridge-functionalized bicyclo[1.1.1]pentanes from azabicyclo[2.1.1]hexanes. J. Am. Chem. Soc..

[20] Hang L (2023). Rhodium-catalyzed intramolecular nitrogen atom insertion into arene rings. J. Am. Chem. Soc..

[21] Zhong H (2023). Skeletal metalation of lactams through a carbonyl-to-nickel-exchange logic. Nat. Commun..

[22] Blakemore DC (2018). Organic synthesis provides opportunities to transform drug discovery. Nat. Chem..

[23] Campos KR (2019). The importance of synthetic chemistry in the pharmaceutical industry. Science.

[24] Bartholomew GL (2024). ^14^N to ^15^N isotopic exchange of nitrogen heteroaromatics through skeletal editing. J. Am. Chem. Soc..

[25] Tolchin ZA, Smith JM (2024). ^15^NRORC: An azine labeling protocol. J. Am. Chem. Soc..

[26] Cheng, Q. et al. Skeletal editing of pyridines through atom-pair swap from CN to CC. *Nat. Chem*. 10.1038/s41557-023-01428-2 (2024). 10.1038/s41557-023-01428-2 PMC11087273 38238464

[27] Morofuji T, Nagai S, Watanabe A, Inagawa K, Kano N (2023). Streptocyanine as an activation mode of amine catalysis for the conversion of pyridine rings to benzene rings. Chem. Sci..

[28] Morofuji T, Inagawa K, Kano N (2021). Sequential ring-opening and ring-closing reactions for converting para-substituted pyridines into meta-substituted anilines. Org. Lett..

[29] Morofuji T, Kinoshita H, Kano N (2019). Connecting a carbonyl and a π-conjugated group through a *p*-phenylene linker by (5+1) benzene ring formation. Chem. Commun..

[30] Cabrera-Pardo JR, Chai DI, Kozmin SA (2013). Silver-promoted benzannulations of siloxyalkynes withpyridinium and isoquinolinium salts. Adv. Synth. Catal..

[31] Fout AR, Bailey BC, Tomaszewski J, Mindiola DJ (2007). Cyclic denitrogenation of n-heterocycles applying a homogeneous titanium reagent. J. Am. Chem. Soc..

[32] Karmacharya U (2021). Novel pyridine bioisostere of cabozantinib as a potent c-met kinase inhibitor: synthesis and anti-tumor activity against hepatocellular carcinoma. Int. J. Mol. Sci..

[33] Dossetter AG, Douglas A, O’Donnell C (2012). A matched molecular pair analysis of in vitro human microsomal metabolic stability measurements for heterocyclic replacements of di-substituted benzene containing compounds − identification of those isosteres more likely to have beneficial effects. Med. Chem. Commun..

[34] Sodano TM, Combee LA, Stephenson CRJ (2020). Recent advances and outlook for the isosteric replacement of anilines. ACS Med. Chem. Lett..

[35] Pennington LD, Moustakas DT (2017). The necessary nitrogen atom: a versatile high-impact design element for multiparameter optimization. J. Med. Chem..

[36] Patel SC, Burns NZ (2022). Conversion of aryl azides to aminopyridines. J. Am. Chem. Soc..

[37] Pearson TJ (2023). Aromatic nitrogen scanning by *ipso*-selective nitrene internalization. Science.

[38] Woo J, Stein C, Christian AH, Levin MD (2023). Carbon-to-nitrogen single-atom transmutation of azaarenes. Nature.

[39] Ding Q, Ye Y, Fan R (2012). Recent advances in phenol dearomatization and its application in complex syntheses. Synthesis.

[40] Zheng C, You S-L (2016). Catalytic asymmetricdearomatization by transition-metal catalysis: a method for trans-formations of aromatic compounds. Chem.

[41] Wertjes WC, Southgate EH, Sarlah D (2018). Recent advances in chemical dearomatization of nonactivated arenes. Chem. Soc. Rev..

[42] Huck CJ, Sarlah D (2020). Shaping molecular landscapes:Recent advances, opportunities, and challenges in dearomatization. Chem.

[43] Li B, Ruffoni A, Leonori D (2023). A photochemical strategy for ortho-aminophenol synthesis via dearomative-rearomative coupling between aryl azides and alcohols. Angew. Chem. Int. Ed..

[44] Mykura, R. et al. Synthesis of polysubstituted azepanes by dearomative ring expansion of nitroarenes. *Nat. Chem*. 10.1038/s41557-023-01429-1. 10.1038/s41557-023-01429-1 38273027

[45] Li G, Lavagnino MN, Ali SZ, Hu S, Radosevich AT (2023). Tandem C/N-Difunctionalization of Nitroarenes: Reductive Amination and Annulation by a Ring Expansion/Contraction Sequence. J. Am. Chem. Soc..

[46] Sundberg RJ, Suter SR, Brenner M (1972). Photolysis of 0-substituted aryl azides in diethylamine. Formation and autoxidation of 2-diethylamino-1H-azepine intermediates. J. Am. Chem. Soc..

[47] Sundberg RJ, Suter SR (1970). Structural rearrangements of aryl nitrenes and related intermediates. J. Org. Chem..

[48] Sundberg RJ, Das BP, Smith RH (1969). Photochemical deoxygenation of aromatic nitro compounds in triethyl phosphite. Substituent effects and evidence for the involvement aryl nitrenes. J. Am. Chem. Soc..

[49] He Y, Wang J, Zhu Z, Wei H (2024). Nitrogen atom insertion into arenols to access benzazepines. Chem. Sci..

[50] Schneider N, Lowe DM, Sayle RA, Tarselli MA, Landrum GA (2016). Big data from pharmaceutical patents: a computational analysis of medicinal chemists’ bread and butter. J. Med. Chem..

[51] Taylor RD, MacCoss M, Lawson AD (2014). Rings in drugs. J. Med. Chem..

[52] Brown DG, Boström J (2016). Analysis of past and present synthetic methodologies on medicinal chemistry: where have all the new reactions gone?. J. Med. Chem..

[53] Yungeng L, Ka-Pang S, Vanessa Kar-Yan L, Chi-Ming C (2023). Iron- and Ruthenium-Catalyzed C–N Bond Formation Reactions. Reactive Metal Imido/Nitrene Intermediates. ACS Catal..

[54] Luo Y, Zhang X, Xia Y (2024). Recent advances in transition-metal catalyzed nitrene transfer reactions with carbamates. Chin. Chem. Lett..

[55] Sun H-C (2008). Solid-state white light-emitting electrochemical cells using iridium-based cationic transition metal complexes. J. Am. Chem. Soc..

[56] Namanga JE (2020). Fluorinated cationic iridium(iii) complex yielding an exceptional, efficient, and long-lived red-light-emitting electrochemical cell. ACS Appl. Energy Mater..

[57] Feng S (2020). Catalytic asymmetric [4 + 2] cycloaddition of ortho-alkenyl naphthols/phenols with ortho-quinone methides: Highly stereoselective synthesis of chiral 2,3,4-trisubstituted chromans. J. Org. Chem..

[58] Patonay, T., Micskei, K., Juhász-Tóth, É., Fekete, S. & Pardi-Tóth, V. C. α-Azido ketones, Part 6^†^. Reduction of acyclic and cyclic α-azido ketones into α-amino ketones: old problems and new solutions. *ARKIVOC***vi**, 270−290 (2009).

[59] We proposed that the byproduct **45** might be derived from [4+2] cyclization between ring expansion intermediate (Fig. 1D, **II**) and azide elimination product of **44**. The detailed procedure can be seen in Supplementary Information, section 2.4.

[60] Wei K, Yang T, Chen Q, Liang S, Yu W (2020). Iron-catalysed 1,2-aryl migration of tertiary azides. Chem. Commun..

[61] Szostak M, Yao L, Aubé J (2010). Proximity effects in nucleophilic addition reactions to medium-bridged twisted lactams: remarkably stable tetrahedral intermediates. J. Am. Chem. Soc..

[62] Bagdanoff, J. T., Behenna, D. C., Stockdill, J. L. & B. M. Stoltz. Enantioselective synthesis of caprolactam and enone precursors to the heterocyclic DEFG ring system of zoanthenol. *Eur. J. Org. Chem*. **2016**, 2101–2104 (2016). 10.1002/ejoc.201600223 PMC5225988 28090188

