# Supplementary material for: Carbon–nitrogen transmutation in polycyclic arenol skeletons to access N-heteroarenes
Source: Nat Commun. 2024 May 4;15:3772. doi: 10.1038/s41467-024-48265-6 (PMC11069502; doi:10.1038/s41467-024-48265-6)
Supplement: Supplementary file 1 — Supplementary Information [file 41467_2024_48265_MOESM1_ESM.pdf]

Supplementary Information for

Carbon–Nitrogen Transmutation in Polycyclic Arenol Skeletons to Access *N*-Heteroarenes

Hong Lu,[1,‡] Yu Zhang,[1,‡] Xiu-Hong Wang,[1] Ran Zhang,[1] Peng-Fei Xu[2,*] and Hao Wei[1,*]

[1]Key Laboratory of Synthetic and Natural Functional Molecule Chemistry of the Ministry of Education, College of Chemistry & Materials Science, Northwest University, Xi'an, China.

[2]State Key Laboratory of Applied Organic Chemistry, School of Chemistry and Chemical Engineering, Lanzhou University, Lanzhou 730000, China.

‡ These authors contributed equally to this work.

* Correspondence: haow@nwu.edu.cn, xupf@lzu.edu.cn

Table of Contents

1. General information 2

2. Supplementary methods 2

   2.1 Substrate synthesis 2

   2.2. Optimization of reaction conditions 6

   2.3 General experimental procedure for the investigation of substrate scope 9

   2.4 Control experiment of carbon–nitrogen transmutation 10

   2.5 Synthetic applications 11

3. Supporting Spectra 14

   3.1 X-Ray Crystallography 14

   3.2 Characterization of starting materials 18

   3.3 Characterization of carbon–nitrogen transmutation products 29

   3.4 Characterization of synthetic application products 41

   3.5 Characterization of unsuccessful example of naphthol 45

   3.6 NMR Spectra 46

4. References 130

1. General information

All reagents were obtained commercially unless otherwise noted. Anhydrous solvent (with Adamas Reagent, Ltd.) were used without further purification. Unless otherwise noted, all the reactions were carried out under nitrogen atmosphere with a stir bar in a sealed vial. Reaction temperatures were reported as the temperatures of the bather surrounding the flasks or vials. Sensitive ligands, catalysts and solvents were transferred under nitrogen into a nitrogen-filled glovebox with standard techniques. Glass wares were heat-dried and cooled down under vacuum prior to use. Column chromatography was carried out on silica gel (300−400 mesh) using a forced flow of eluent at 0.3−0.5 bar pressure. Flash column chromatography was carried out using silica gel (200−300 mesh) at increased pressure. HRMS were performed on Bruker Daltonics MicroTof-Q II mass spectrometer. Cyclic Voltammetry studies were performed using a Shanghai Chenhua CHI760E workstation. UV-vis absorption spectra were were performed using a SHIMADZU UV-1700 device. ^{1}H NMR, ^{13}C NMR and ^{19}F NMR spectra were recorded on a WNMR-I spectrometer (400 MHz ^{1}H and 100 MHz ^{13}C). The spectra were recorded in $CDCl_3$ as the solvent at room temperature. ^{1}H and ^{13}C chemical shifts are reported in ppm relative to either the residual solvent peak (^{13}C) or TMS (^{1}H) as an internal standard.

Caution! Heating tetrabutylammonium azide with acid in a sealed tube will potentially generate hydrazoic acid which is toxic and explosive. While we encountered no issues with the handling of these materials, proper protective measures should be used at all time.

2. Supplementary methods

2.1 Substrate synthesis

2.1.1 General procedure A for preparation of substrates

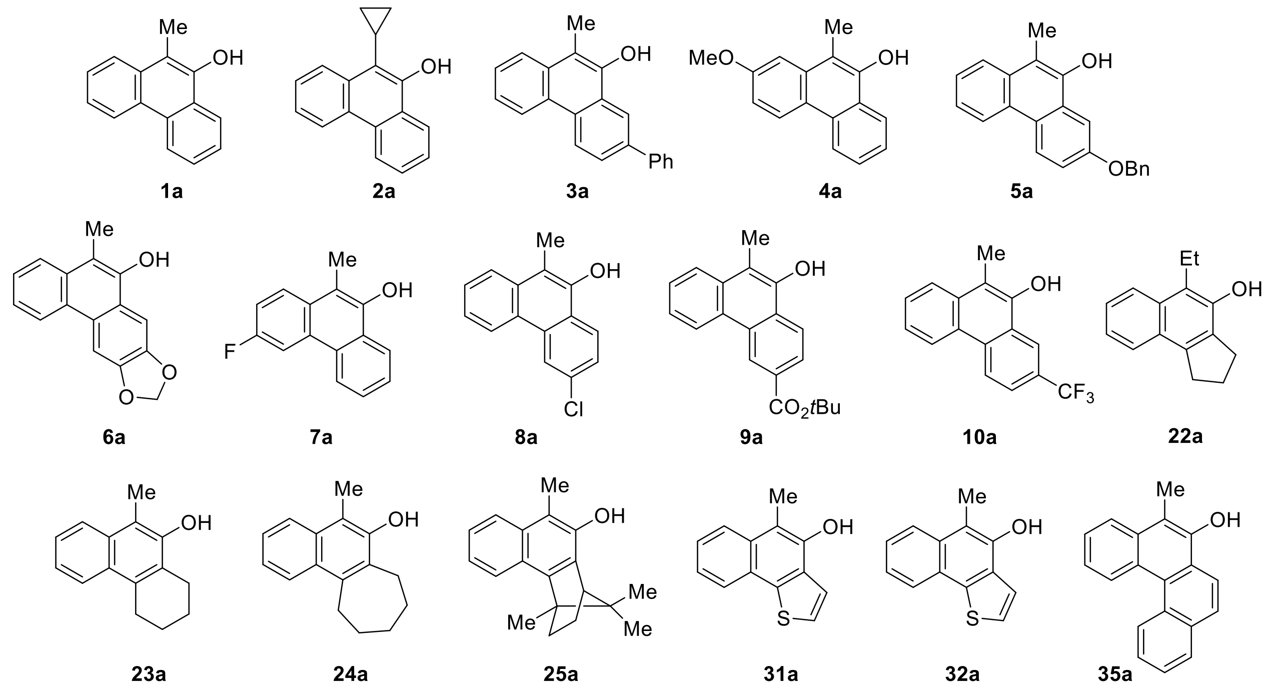

Supplementary Figure 1. Substrates used in this study.

In Supplementary Figure 1, substrates were synthesized according to literature procedure with little modification[1].

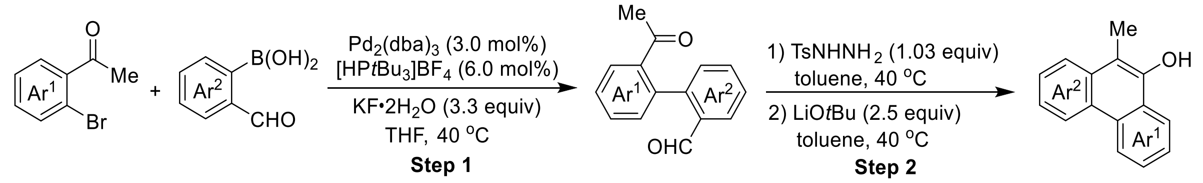

Step 1: Under an argon atmosphere, $Pd_2(dba)_3$ (0.015 mmol), $[HP(tBu)_3]BF_4$ (0.03 mmol), aryl halide (0.5 mmol), boronic acid (0.55 mmol), $KF \cdot 2H_2O$ (1.65 mmol) and THF (1.0 mL) were successively added to a 10 mL round-bottomed flask equipped with a condenser. The mixture was reacted at 40 °C until the reaction was complete (monitored by TLC). The mixture was diluted with EtOAc (2.0 mL) and filtered through a plug of silica gel. The filtrate was concentrated, and the residue was purified by silica gel column chromatography to afford the ketoaldehyde product.

Step 2: Under an argon atmosphere, the ketoaldehydes (0.30 mmol), $TsNHNH_2$ (57.5 mg, 0.309 mmol, 1.03 eq) and toluene (6.0 mL) were successively added to a flame-dried 10 mL Schlenk flask. The reaction was heated at 40 ºC until the ketoaldehydes was consumed (monitored by TLC). At this point LiO*t*Bu (60.0 mg, 0.75 mmol, 2.5 equiv) was added to the flask and the resulting solution was stirred at 40 °C for eaction was complete (monitored by TLC). The reaction was then quenched by adding AcOH (45.0 mg, 0.75 mmol, 2.5 eq) and cooled to room temperature. Then the reaction solution was filtered through a short plug of silica gel [washed with petroleum ether/EtOAc = 1/1 (10 mL)]. Solvent was then removed in vacuo to provide a crude mixture, which was purified by silica gel column chromatography to afford the desired product.

2.1.2 General procedure B to D for preparation of β-naphthol derivatives

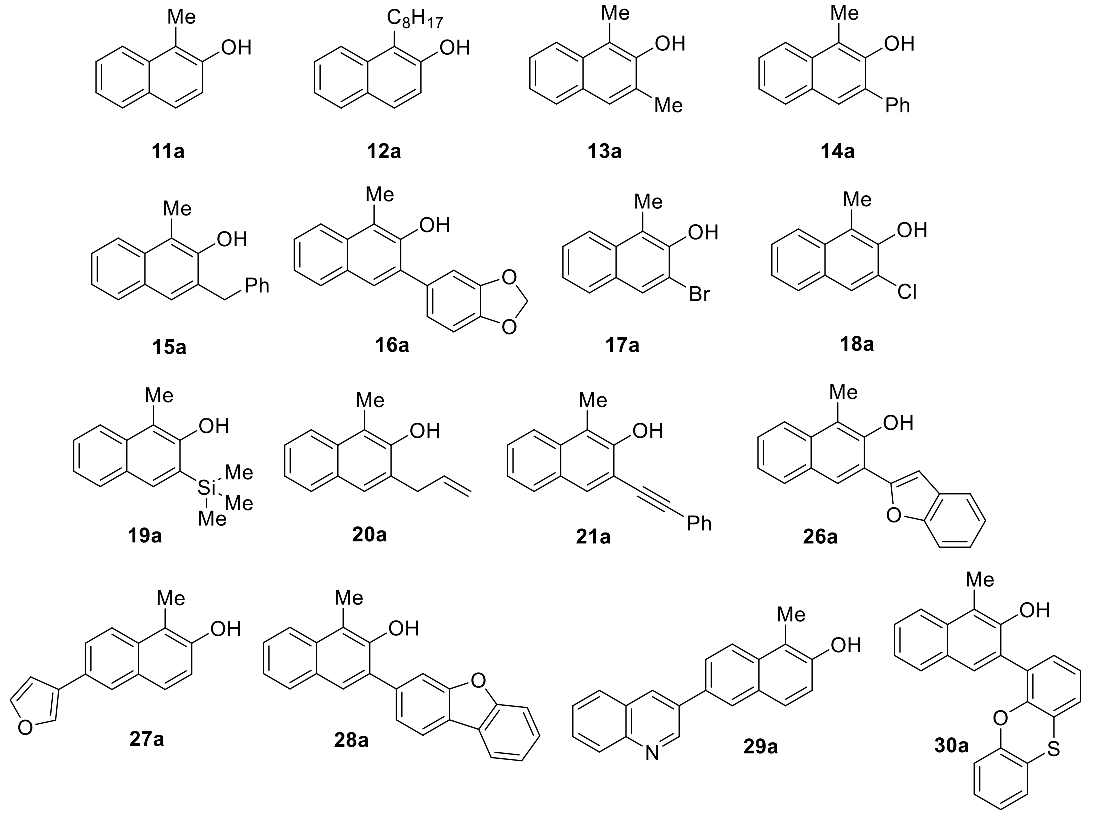

Supplementary Figure 2 Naphthol derivatives used in this study.

In Supplementary Figure 2, compounds **11a** to **14a**,[2] **15a**,[3] **17a**,[2] **18a**,[2] **19a**,[4] **20a**,[2] **21a**,[2] were prepared according to the literature, **16a**, **26a** to **30a** were prepared according to the following procedure (*vide infra*).

Procedure B for preparation of 16a, 26a, 28a

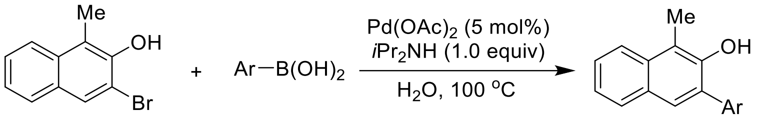

Under an argon atmosphere, 3-bromo-2-(methoxymethoxy)-1-methylnaphthalene (1.0 mmol)[2], aryl boronic acid (1.5 mmol), *i*Pr_2NH (1.0 mmol), $Pd(OAc)_2$ (0.02 mmol) and H_2O (2.0 mL) were successively added to a 10 mL two-neck round-bottomed flask equipped with a condenser. The mixture was reacted at 100 °C until the reaction was complete (monitored by TLC). The mixture was filtered through a pad of celite and washed with EtOAc. The aqueous layer was extracted with EtOAc (3 × 5 mL). The organic layer was washed with brine, dried over Na_2SO_4, filtered and then concentrated. The residue was purified by silica gel column chromatography to afford the desired product.

Procedure C for preparation of 27a and 29a

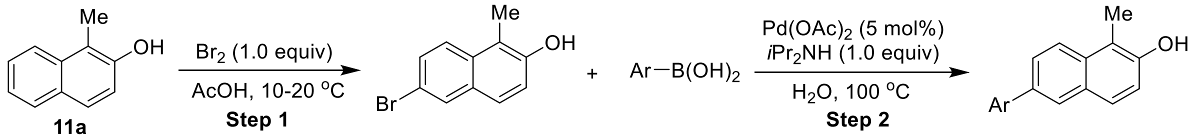

Step 1 [5]: A solution of bromine (1.6 g, 10.1 mL) in AcOH (3.0 mL) was added dropwise to a cooled (10 °C) solution of **11a** (10.1 mmol) in AcOH (5.0 mL) in 25 mL round-bottomed flask; the temperature of the solution was maintained at 10–20 °C. After stirring for 15 min, the mixture was poured into water. The precipitate was filtered, washed with water, and dried in vacuo to give 6-bromo-1-methyl-2-naphthol as a reddish powder.

Step 2: Under an argon atmosphere, 6-bromo-1-methyl-2-naphthol (2.0 mmol), aryl boronic acid (3.0 mmol), *i*Pr_2NH (2.0 mmol), $Pd(OAc)_2$ (0.04 mmol) and H_2O (4.0 mL) were successively added to a 25 mL two-neck round-bottomed flask equipped with a condenser. The mixture was reacted at 100 °C until the reaction was complete (monitored by TLC). The mixture was filtered through a pad of celite and washed with EtOAc. The aqueous layer was extracted with EtOAc (3 × 10 mL). The organic layer was washed with brine, dried over Na_2SO_4, filtered and then concentrated. The residue was purified by silica gel column chromatography to afford the desired product.

Procedure D for preparation of 30a

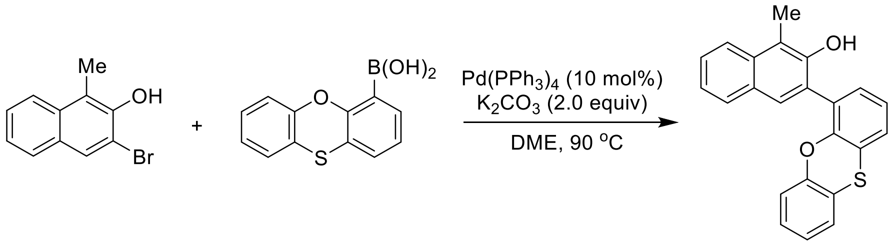

Under an argon atmosphere, 3-bromo-2-(methoxymethoxy)-1-methylnaphthalene (1.0 mmol), phenoxathiin-4-ylboronic acid (1.4 mmol), $Pd(PPh_3)_4$ (0.01 mmol), K_2CO_3 (2.0 M, 2.0 equiv) and 1,2-dimethoxyethane (DME) (6.0 mL) were successively added to a 25 mL two-neck round-bottomed flask equipped with a condenser. The mixture was reacted at 90 °C until the reaction was complete (monitored by TLC). The mixture was filtered through a pad of celite and washed with EtOAc. The aqueous layer was extracted with EtOAc (2 × 5 mL). The organic layer was washed with brine, dried over Na_2SO_4, filtered and then concentrated. The residue was purified by silica gel column chromatography to afford the 6-arylnaphthol.

2.1.3 General procedure E for preparation of 1,3-dimethylanthracen-2-ol (33a)

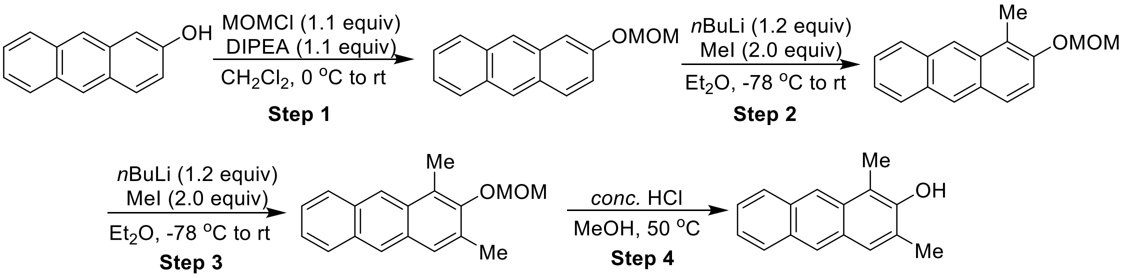

Step 1: Anthracen-2-ol (0.5 mmol), CH_2Cl_2 (4.0 mL) and diisopropylethylamine (DIPEA, 0.55 mmol) were successively added to 10 mL round-bottomed flask. The mixture was cooled to 0 ºC and chloromethyl methyl ether (MOMCl, 0.55 mmol) was slowly added. The reaction was stirred overnight at room temperature. After completion (monitored by TLC), the reaction was quenched by methanol (3.0 mL). The mixture was extracted with CH_2Cl_2 (3 × 5 mL), washed with brine, dried over Na_2SO_4, and concentrated. The products were purified by column chromatography to give 2-(methoxymethoxy)anthracene.

Step 2: 2-(methoxymethoxy)Anthracene (1.0 mmol) and ether (4.0 mL) was added to a flame-dried 25 mL Schlenk tube. The mixture was cooled to 0 °C and *n*BuLi (2.5 M in *n*hexane, 1.1 mmol) was slowly added. After stirring for 1 h at room temperature, iodomethane (2.0 mmol) was slowly added and stirring for 1 h at room temperature. After completion (monitored by TLC), the reaction was quenched by NH_4Cl aq. The aqueous layer was extracted with EtOAc (3 × 10 mL) and the combined organic layers were washed with brine, dried over Na_2SO_4 and concentrated. The crude product was purified by column chromatography to give 2-(methoxymethoxy)-1-methylanthracene.

Step 3: 2-(methoxymethoxy)-1-methylanthracene (1.0 mmol) and ether (4.0 mL) was added to a flame-dried 25 mL

Schlenk tube. The mixture was cooled to 0 °C and *n*BuLi (2.5 M in *n*hexane, 1.1 mmol) was slowly added. After stirring for 1 h at room temperature, iodomethane (2.0 mmol) was slowly added and stirring for 1 h at room temperature. After completion (monitored by TLC), the reaction was quenched by NH_4Cl aq. The aqueous layer was extracted with EtOAc (3 × 10 mL) and the combined organic layers were washed with brine, dried over Na_2SO_4 and concentrated. The crude product was purified by column chromatography to give 2-(methoxymethoxy)-1,3-dimethylanthracene.

Step 4: 2-(methoxymethoxy)-1,3-Dimethylanthracene (0.5 mmol), MeOH (3.5 mL) and conc. HCl (35%, 3 drops) were successively added to 10 mL round-bottomed flask equipped with a condenser. The reaction was warmed to 50 °C and stirred for 1 h. After completion (monitored by TLC), the reaction was cooled to room temperature and water was added. The mixture was extracted with EtOAc (3 × 8 mL), The combined organic layers were washed with brine, dried over Na_2SO_4, and concentrated. The crude product was purified by column chromatography to give 1,3-dimethylanthracen-2-ol.

2.1.4 General procedure F for preparation of 2,4-dimethyltetraphen-3-ol (34a)

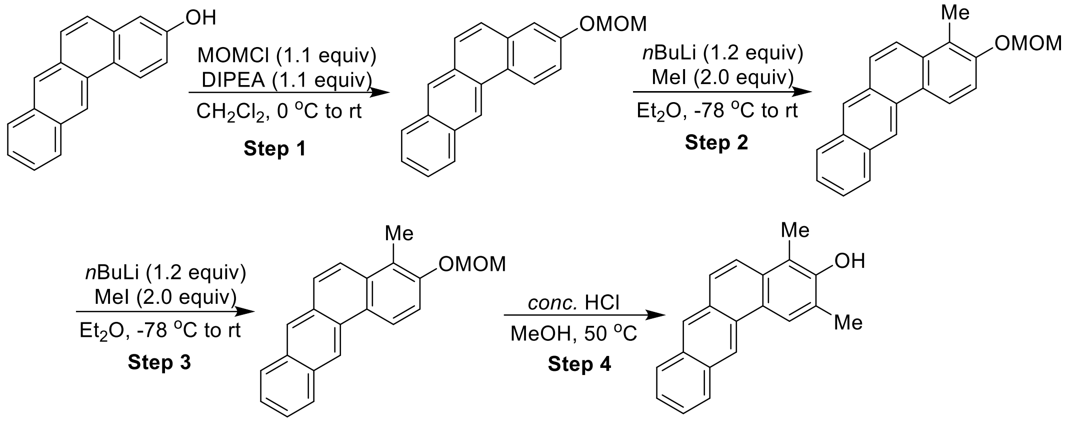

Step 1: Tetraphen-3-ol[6] (0.5 mmol), CH_2Cl_2 (4.0 mL) and diisopropylethylamine (DIPEA, 0.55 mmol) were successively added to 10 mL round-bottomed flask. The mixture was cooled to 0 °C and chloromethyl methyl ether (MOMCl, 0.55 mmol) was slowly added. The reaction was stirred overnight at room temperature. After completion (monitored by TLC), the reaction was quenched by methanol (3.0 mL). The mixture was extracted with CH_2Cl_2 (3 × 5 mL), washed with brine, dried over Na_2SO_4, and concentrated. The products were purified by column chromatography to give 3-(methoxymethoxy)tetraphene.

Step 2: 3-(methoxymethoxy)Tetraphene (1.0 mmol) and ether (4.0 mL) was added to a flame-dried 25 mL Schlenk tube. The mixture was cooled to 0 °C and *n*BuLi (2.5 M in *n*hexane, 1.1 mmol) was slowly added. After stirring for 1 h at room temperature, iodomethane (2.0 mmol) was slowly added and stirring for 1 h at room temperature. After completion (monitored by TLC), the reaction was quenched by NH_4Cl aq. The aqueous layer was extracted with EtOAc (3 × 10 mL) and the combined organic layers were washed with brine, dried over Na_2SO_4 and concentrated. The crude product was purified by column chromatography to give 3-(methoxymethoxy)-4-methyltetraphene.

Step 3: 3-(methoxymethoxy)-4-Methyltetraphene (1.0 mmol) and ether (4.0 mL) was added to a flame-dried 25 mL Schlenk tube. The mixture was cooled to 0 °C and *n*BuLi (2.5 M in *n*hexane, 1.1 mmol) was slowly added. After stirring for 1 h at room temperature, iodomethane (2.0 mmol) was slowly added and stirring for 1 h at room temperature. After completion (monitored by TLC), the reaction was quenched by NH_4Cl aq. The aqueous layer was extracted with EtOAc (3 × 10 mL) and the combined organic layers were washed with brine, dried over Na_2SO_4 and concentrated. The crude product was purified by column chromatography to give 3-(methoxymethoxy)-2,4-dimethyltetraphene.

Step 4: 3-(methoxymethoxy)-2,4-Dimethyltetraphene (0.5 mmol), MeOH (3.5 mL) and conc. HCl (35%, 3 drops) were

successively added to 10 mL round-bottomed flask equipped with a condenser. The reaction was warmed to 50 ºC and stirred for 1 h. After completion (monitored by TLC), the reaction was cooled to room temperature and water was added. The mixture was extracted with EtOAc (3 × 10 mL), The combined organic layers were washed with brine, dried over Na_2SO_4, and concentrated. The crude product was purified by column chromatography to give 2,4-dimethyltetraphen-3-ol.

2.2. Optimization of reaction conditions

2.2.1 General procedure for optimization

10-Methylphenanthren-9-ol (0.1 mmol), *N*-Bromosuccinimide (NBS) (0.12 mmol), N(*n*Bu_4)N_3 (0.12 mmol), $(PhO)_2POOH$ (0.05 mmol), catalyst (0.015 mmol), ligand (0.02 mmol), additives (0.01 mmol) and solvent (1.0 mL) was successively added to a 10 mL sealed tube equipped with a Teflon-coated magnetic stir bar. The tube then was sealed with a Teflon screw cap and placed on a hotplate pre-heated to 100 °C with vigorous stirring. After 18 h, the reaction was cooled to room temperature and another portion of NBS (0.06 mmol), N(*n*Bu_4)N_3 (0.1 mmol) and $(PhO)_2POOH$ (0.025 mmol) was successively added to the sealed tube. The tube then reacted at 100 °C with vigorous stirring. After 18 h, the reaction was cooled to room temperature. The solvent was evaporated and the residue was directly purified by flash column chromatography on silica gel to give the desired products.

Supplementary Table 1. Screening for catalyst[a]

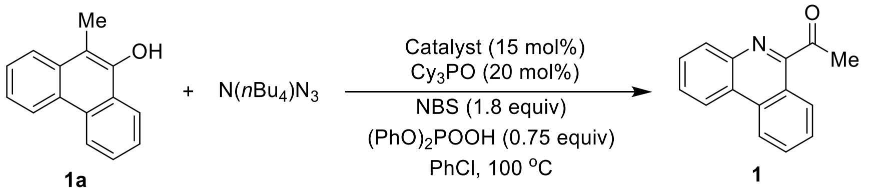

Entry	Catalyst	Yiled (%)[b]
1	$FeBr_2$	80
2	$FeCl_2$	71
3	$Fe(OTf)_2$	75
4	$Fe(OAc)_2$	32
5	$Fe(acac)_3$	10
6	$Cu(OTf)_2$	<10
7	$[[CH_3(CH_2)_6CO_2]_2Rh]_2$	34
8	TMOPP-Co	trace
9	$RuCl_3$	24
10	$Ru_3(CO)_{13}$	<10
11	[(*p*-cymene)$RuCl_2$]$_2$	34
12[c]	$FeBr_2$	59
13[d]	$FeBr_2$	68
14[e]	$FeBr_2$	NR
15[f]	$FeBr_2$	NR
16[g]	$FeBr_2$	76

[a] Unless otherwise specified, all reactions were carried out using **1a** (0.1 mmol), NBS (0.12 mmol), N(*n*Bu_4)N_3 (0.2 mmol), $FeBr_2$ (0.015 mmol), Cy_3PO (0.02 mmol) and $(PhO)_2POOH$ (0.05 mmol) in PhCl (1.0 mL) at 100 °C. After 18 h. another portion of NBS (0.06 mmol),

$N(nBu_4)N_3$ (0.1 mmol) and $(PhO)_2POOH$ (0.025 mmo) were added and reacted at 100 °C for 18 h. [b] Isolated yield. [c] $FeBr_2$ (0.01 mmol) and Cy_3PO (0.015 mmol) were added. [d] NBS (0.18 mmol), $N(nBu_4)N_3$ (0.3 mmol), $FeBr_2$ (0.015 mmol), Cy_3PO (0.02 mmol) and $(PhO)_2POOH$ (0.075 mmol) were added in PhCl (1.0 mL) and reacted at 100 ºC for 36 h. [e] The reaction was carried out without NBS. [f] The reaction was carried out without $N(nBu_4)N_3$. [g] The reaction was carried out with addition of 5.0 equiv H_2O.

Supplementary Table 2. Screening for ligand[a]

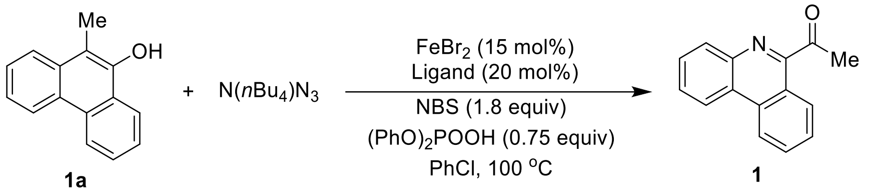

Entry	Ligand	Yiled (%)[b]
1	Cy_3PO	80
2	Ph_3PO	57
3	nBu_3PO	55
4	PCy_3	51
5	Dppb	54
6	2,2'-Bipyridine	64
7	–	70

[a] Unless otherwise specified, all reactions were carried out using **1a** (0.1 mmol), NBS (0.12 mmol), $N(nBu_4)N_3$ (0.2 mmol), $FeBr_2$ (0.015 mmol), ligand (0.02 mmol) and $(PhO)_2POOH$ (0.05 mmol) in PhCl (1.0 mL) at 100 °C. After 18 h. another portion of NBS (0.06 mmol), $N(nBu_4)N_3$ (0.1 mmol) and $(PhO)_2POOH$ (0.025 mmo) were added and reacted at 100 °C for 18 h. [b] Isolated yield.

Supplementary Table 3. Screening for solvent[a]

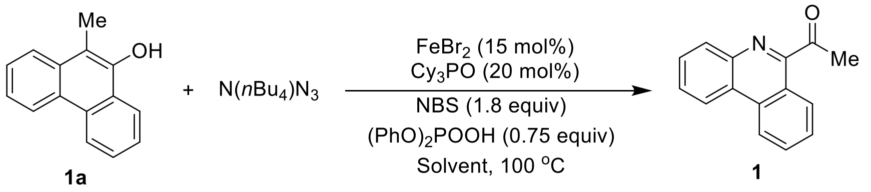

Entry	Solvent	Yiled (%)[b]
1	PhCl	80
2	Toluene	75
3	THF	70
4	1,4-dioxane	55
5	MeCN	48
6	MTBE	30

[a] Unless otherwise specified, all reactions were carried out using **1a** (0.1 mmol), NBS (0.12 mmol), $N(nBu_4)N_3$ (0.2 mmol), $FeBr_2$ (0.015 mmol), Cy_3PO (0.02 mmol) and $(PhO)_2POOH$ (0.05 mmol) in solvent (1.0 mL) at 100 °C. After 18 h. another portion of NBS (0.06 mmol), $N(nBu_4)N_3$ (0.1 mmol) and $(PhO)_2POOH$ (0.025 mmo) were added and reacted at 100 °C for 18 h. [b] Isolated yield.

2.2.2 Conditions for azidation

10-Methylphenanthren-9-ol (0.1 mmol), Br source, azide precursors, $FeBr_2$ (0.015 mmol), Cy_3PO (0.02 mmol), additives and solvent (1.0 mL) were successively added to a 10 mL sealed tube equipped with a Teflon-coated magnetic stir bar. The tube then was sealed with a Teflon screw cap and placed on a hotplate pre-heated to 100 °C with vigorous stirring. After 36 h, the reaction was cooled to room temperature. The solvent was evaporated and the residue was directly purified

by flash column chromatography on silica gel to give the desired products.

Condition A[7]

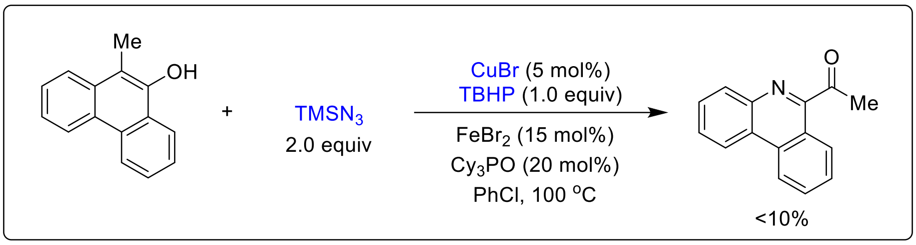

Condition B[8]

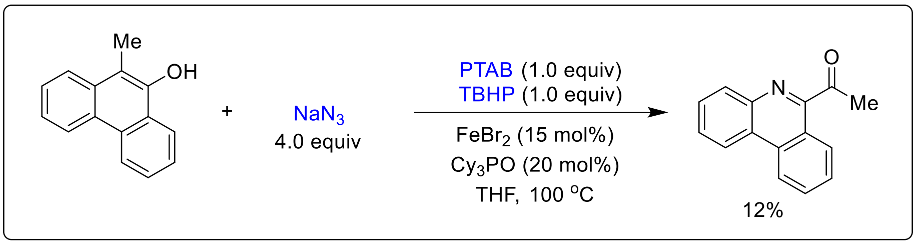

Condition C[9]

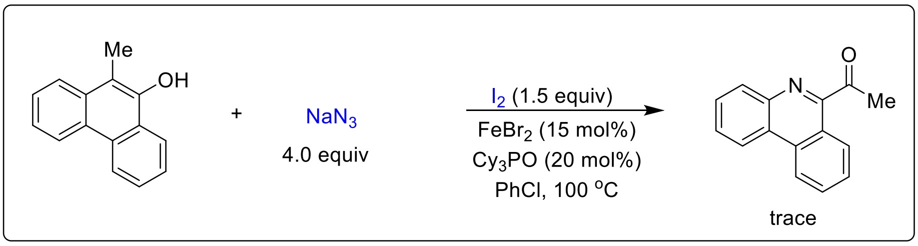

Condition D[10]

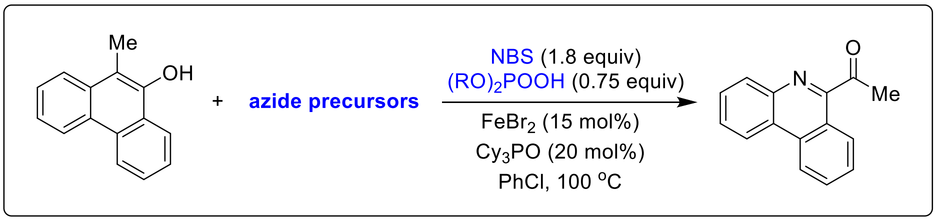

The following conditions of Supplementary Table 4 were based on condition D.

Supplementary Table 4. Optimization of azidation based on condition D[a]

Condtions	Azide precursors	R_2POOH	Yiled (%)[b]
1	N(*n*Bu$_4$)N$_3$	(PhO)$_2$POOH	68
2	NaN$_3$	(PhO)$_2$POOH	trace
3	TMSN$_3$	(PhO)$_2$POOH	N.R.
4	Togni-N$_3$	(PhO)$_2$POOH	N.R.
5	N(*n*Bu$_4$)N$_3$	(BnO)$_2$POOH	45
6	N(*n*Bu$_4$)N$_3$	BINOL	42
7[c]	N(*n*Bu$_4$)N$_3$	(PhO)$_2$POOH	39
8[d]	N(*n*Bu$_4$)N$_3$	(PhO)$_2$POOH	67
9[e]	N(*n*Bu$_4$)N$_3$	(PhO)$_2$POOH	80
10[f]	N(*n*Bu$_4$)N$_3$	–	trace

[a] Unless otherwise specified, all reactions were carried out using **1a** (0.1 mmol), NBS (0.18 mmol), azide precursors (0.3 mmol), FeBr$_2$ (0.015 mmol), Cy$_3$PO (0.02 mmol) and (RO)$_2$POOH (0.075 mmol) in solvent (1.0 mL) at 100 °C for 36 h. [b] Isolated yield. [c] 20 mol% (PhO)$_2$POOH was used. [d] 1.0 equiv (PhO)$_2$POOH was used. [e] **1a** (0.1 mmol), NBS (0.12 mmol), N(*n*Bu$_4$)N$_3$ (0.2 mmol), FeBr$_2$ (0.015 mmol), Cy$_3$PO (0.02 mmol) and (PhO)$_2$POOH (0.05 mmol) in PhCl (1.0 mL) at 100 °C. After 18 h. another portion of NBS (0.06 mmol), N(*n*Bu$_4$)N$_3$ (0.1 mmol) and (PhO)$_2$POOH (0.025 mmo) were added and reacted at 100 °C for 18 h. [f] The reaction was carried out without (PhO)$_2$POOH.

2.2.3 Unsuccessful examples of naphthol

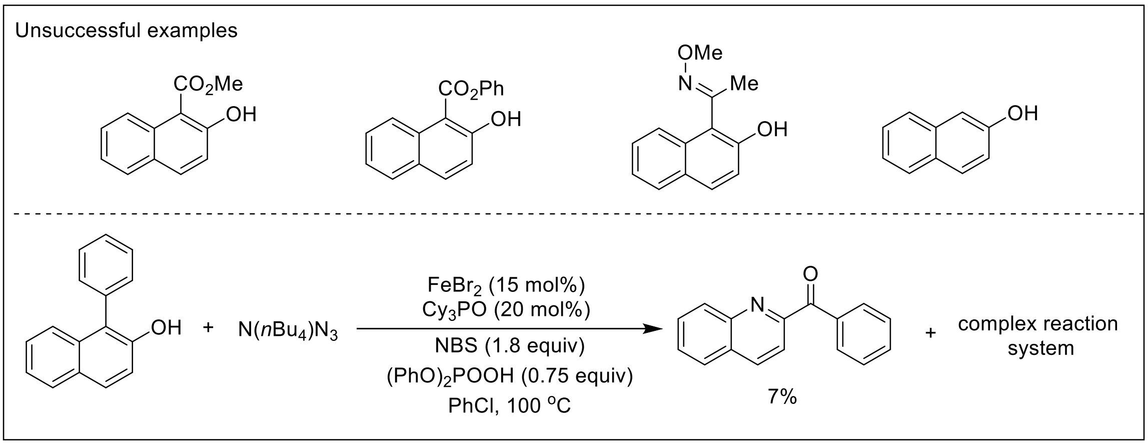

2.3 General experimental procedure for the investigation of substrate scope

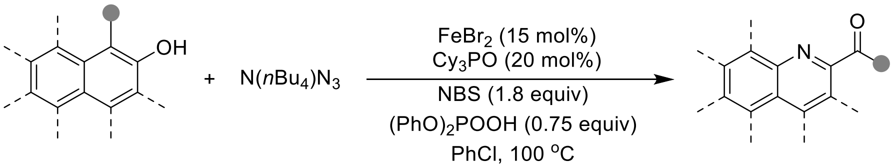

Substrate (0.1 mmol), NBS (0.12 mmol), N(*n*Bu$_4$)N$_3$ (0.2 mmol), FeBr$_2$ (0.015 mmol), tricyclohexylphosphine oxide (0.02 mmol), (PhO)$_2$POOH (0.05 mmol), and PhCl (1.0 mL) was successively added to an 10 mL sealed tube equipped with a Teflon-coated magnetic stir bar. The tube then was sealed with a Teflon screw cap and placed on a hotplate pre-heated to 100 °C with vigorous stirring. After 18 h, the reaction was cooled to room temperature and another portion of NBS (0.06 mmol, 0.6 equiv), N(*n*Bu$_4$)N$_3$(0.1 mmol) and (PhO)$_2$POOH (0.025 mmol) was successively added to the sealed tube. The tube then reacted at 100 °C with vigorous stirring. After 18 h, the reaction was cooled to room temperature. The solvent was evaporated and the residue was directly purified by flash column chromatography on silica

gel to give the desired products.

2.4 Control experiment of carbon–nitrogen transmutation

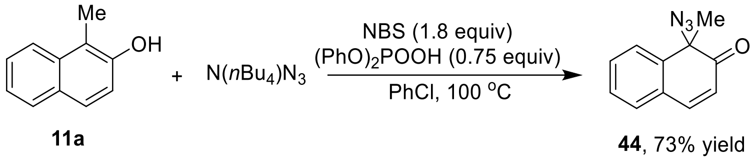

1-Methylnaphthalen-2-ol (0.1 mmol), NBS (0.18 mmol), N(nBu$_4$)N$_3$ (0.3 mmol), (PhO)$_2$POOH (0.075 mmol, 75 mol%) and PhCl (1.0 mL) was successively added to a 10 mL sealed tube equipped with a Teflon-coated magnetic stir bar. The tube then was sealed with a Teflon screw cap and placed on a hotplate pre-heated to 100 °C with vigorous stirring. After 2 h, the reaction was cooled to room temperature. The solvent was evaporated and the residue was directly purified by flash column chromatography on silica gel to give the azide product.

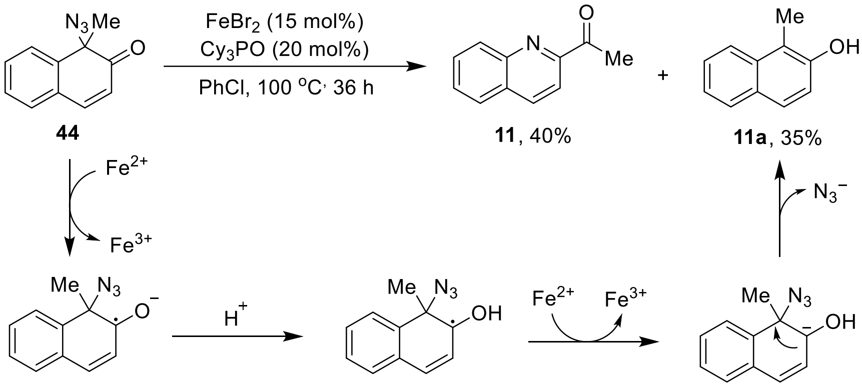

1-azido-1-methylnaphthalen-2(1*H*)-one (19.9 mg, 0.1 mmol), FeBr$_2$ (0.015 mmol), tricyclohexylphosphine oxide (0.02 mmol), and PhCl (1.0 mL) was successively added to a 10 mL sealed tube equipped with a Teflon-coated magnetic stir bar. The tube then was sealed with a Teflon screw cap and placed on a hotplate pre-heated to 100 °C with vigorous stirring. After 36 h, the reaction was monitored by TLC, **11a** (35% yield) and **11** (40% yield) were observed respectively, which demonstrated that the proposed azidation is a reversible process via successive single-electron transfer (SET) from Fe(II) to eliminate azide.[11]

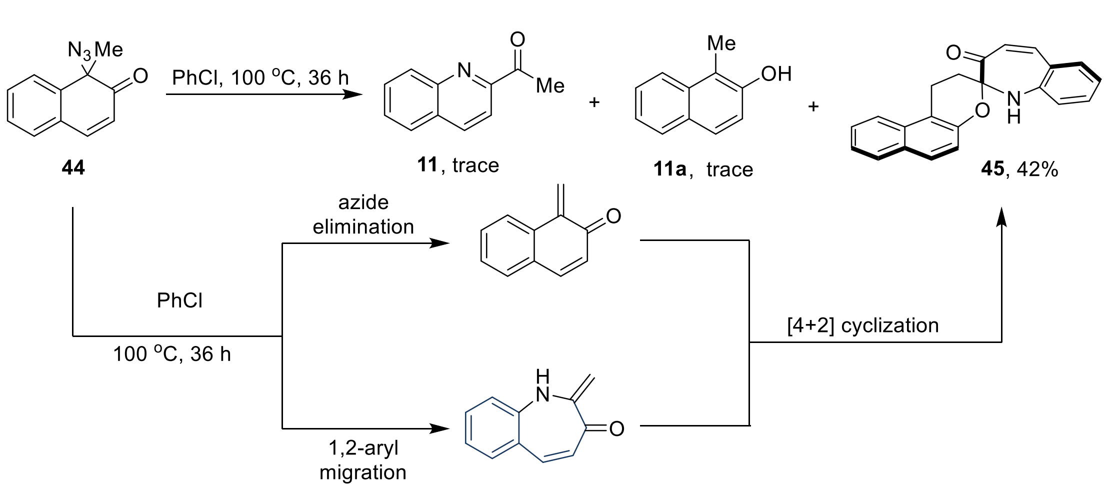

1-Azido-1-methylnaphthalen-2(1*H*)-one (19.9 mg, 0.1 mmol) and PhCl (1.0 mL) was successively added to a 10 mL sealed tube equipped with a Teflon-coated magnetic stir bar. The tube then was sealed with a Teflon screw cap and placed

on a hotplate pre-heated to 100 °C with vigorous stirring. After 36 h, the reaction was monitored by TLC, byproduct **45** was isolated in 42% yield and recovered **44** in 27%, and only trace amount of **11a** and **11** were observed. We speculated that the byproduct **45** is synthesized by [4+2] cyclization between azide elimination[12] and aryl migration intermediate.

2.5 Synthetic applications

2.5.1 General procedure G for synthesis of 36 [13]

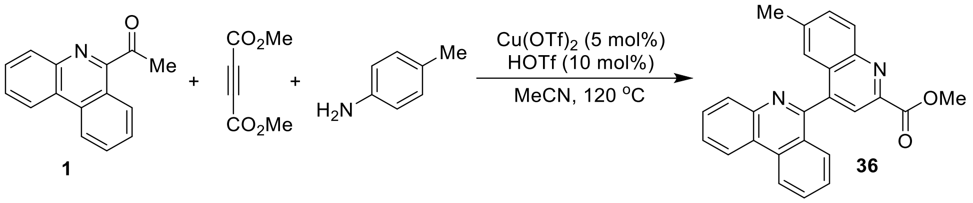

Dimethyl but-2-ynedioate (0.1 mmol), *p*-toluidine (0.1 mmol), 1-(phenanthridin-6-yl)ethan-1-one (0.12 mmol), $Cu(OTf)_2$ (5 mol%), HOTf (0.1 mmol) and MeCN (0.3 mL) successively added to an oven-dried seal tube. Then the tube was sealed and heated to 120 °C for 24 h until substrate was consumed (monitored by TLC). The crude mixture was quenched by saturated $NaHCO_3$ (2 mL) and dilute with CH_2Cl_2 (3 mL). The aqueous was extracted with CH_2Cl_2 (2 × 5 mL). The combined organic layer was dried over anhydrous Na_2SO_4, filtrated and concentrated. The crude product was then purified by column chromatography to give the methyl 6-methyl-4-(phenanthridin-6-yl)quinoline-2-carboxylate.

2.5.2 General procedure H for synthesis of 37

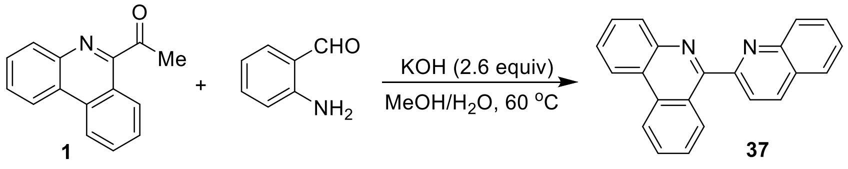

2-Aminobenzaldehyde (0.11 mmol), 1-(phenanthridin-6-yl)ethan-1-one (0.1 mmol) (**1**), MeOH (0.3 mL) and a solution of potassium hydroxide (14.9 mg in 0.3 mL of water) were successively added to a 10 mL Schlenk tube. The mixture was allowed to stir at 60 °C for 12 h until substrate was consumed (monitored by TLC). The reaction was quenched by the addition of 4 mL of water and placed in an ice bath prior to vacuum filtration. The solid was washed with ice cold water and dried under vacuum to yield the 6-(quinolin-2-yl)phenanthridine.

2.5.3 General procedure I for synthesis of 38

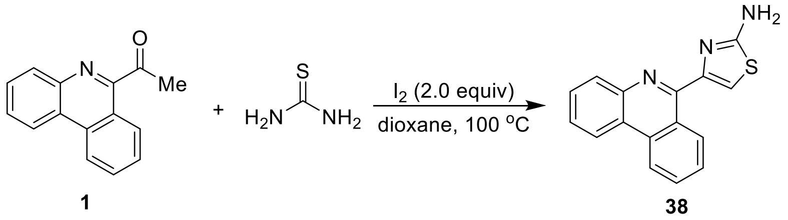

1-(phenanthridin-6-yl)Ethan-1-one (0.1 mmol), thiourea (0.2 mmol) and iodine (0.2 mmol) were dissolved in 1,4-dioxane (1.0 mL) and the mixture was stirred at 100°C for 3 hours. Further portions of thiourea (0.1 mmol) and iodine (0.1 mmol) were added and the mixture was stirred at 100°C for another 4 hours until substrate was consumed (monitored by TLC). Saturated aqueous $NaHCO_3$ solution (2.0 mL) was added and the mixture was extracted with CH_2Cl_2 (3 × 2 mL). The organic phase was washed with aqueous sodium thiosulfate solution (3 mL) and water (3 mL). The combined

organic layer was dried over anhydrous Na_2SO_4, filtrated and concentrated. The crude product was then purified by column chromatography to give the 4-(phenanthridin-6-yl)thiazol-2-amine as a brown solid.

2.5.4 General procedure J for synthesis of 39

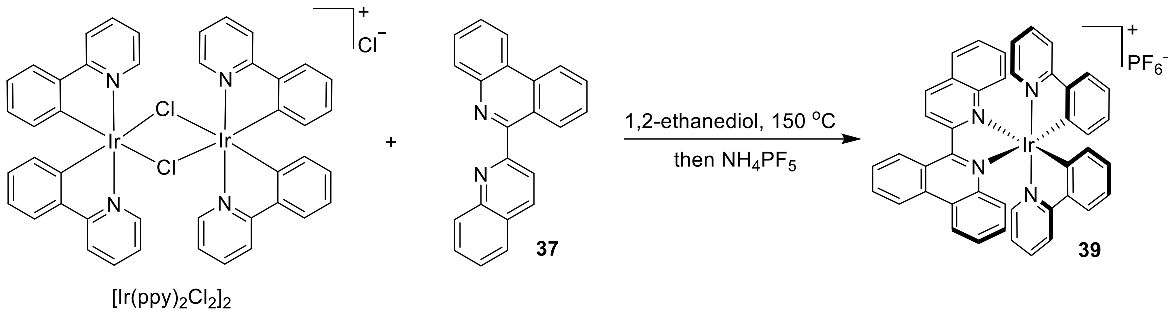

$[Ir(ppy)_2Cl_2]_2$ (0.15 mmol), **37** (0.32 mmol) and 1,2-ethanediol (10 mL) were successively added to an oven-dried 20 mL sealed tube equipped with a Teflon-coated magnetic stir bar. The tube then was sealed with a Teflon screw cap, moved out of the glovebox, and placed on a hotplate pre-heated to 160 °C with vigorous stirring. After 36 h, the reaction was cooled to room temperature, and an aqueous solution of NH_4PF_6 (400 mg in 4 mL of deionized water) was added. The red precipitate was filtered and purified by column chromatography to give compound **39** as a red solid in 75% yield.

2.5.5 General procedure K for synthesis of 40

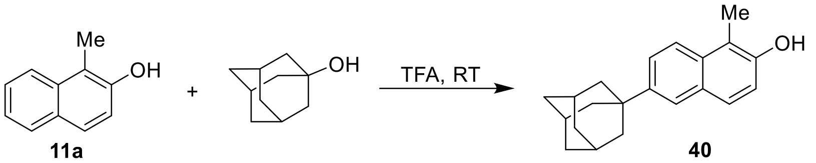

Adamantan-1-ol (1.0 mmol) and 1-methylnaphthalen-2-ol (**11a**) (1.0 mmol) were dissolved in trifluoroacetic acid and the mixture was stirred at room temperature for 12 h until 1-methylnaphthalen-2-ol (**11a**) was consumed (monitored by TLC). Water (4.0 mL) was added to dilute the reaction mixture. The precipitate was filtered, washed with a solution of sodium carbonate, and dried in vacuo to give 6-((3r,5r,7r)-adamantan-1-yl)-1-methylnaphthalen-2-ol (**40**) as pale yellow solid.

2.5.7 General procedure L for synthesis of 41

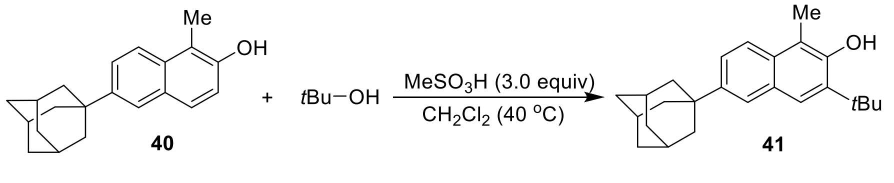

40 (0.5 mmol), *t*BuOH (0.75 mmol) and CH_2Cl_2 (2.0 mL) were successively added to a 10 mL round-bottomed flask and stirred at room temperature. The mixture was added $MeSO_3H$ dropwise with external cooling by a water bath and stirred for 18 h at room temperature until **40** was consumed (monitored by TLC). After that, the reaction mixture was poured onto ice water (2.0 mL) with vigorous stirring and warmed to room temperature. The aqueous layer was extracted with CH_2Cl_2 (3 × 2 mL). The combined organic layer was washed with brine, dried over anhydrous Na_2SO_4, filtrated and concentrated. The crude product was then purified by column chromatography to give the desired product.

2.5.8 General porcedure N for the synthesis of 43 [14]

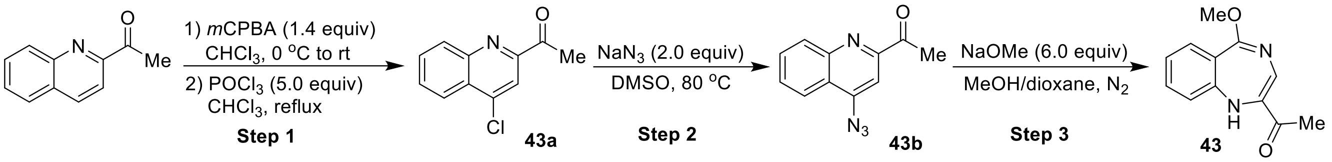

Step 1: *m*CBPA (0.7 mmol) and 1-(quinolin-2-yl)ethan-1-one (0.5 mmol) and $CHCl_3$ (4.0 mL) were successively added to a 25 mL round bottom flask equipped with a stirring bar at 0 °C. The reaction was warmed to room temprature and stirred for 24 h until the substrate was consumed (monitored by TLC). Then, the reaction was washed with saturated $NaHCO_3$ (2 × 5 mL). The organic phases were dried over Na_2SO_4 and concentrated. The crude product was dissolved in $CHCl_3$ (3 mL) and $POCl_3$ (0.23 mL, 2.5 mmol) was added. The mixture was reflux for 3 h, and then poured into ice-water. The mixture was wash with $NaHCO_3$ (3 × 5 mL). The organic phase was dried over Na_2SO_4, concentrated and purified by flash column chromatography to afford 1-(4-chloroquinolin-2-yl)ethan-1-one.

Step 2: 1-(4-chloroquinolin-2-yl)ethan-1-one (0.5 mmol), sodium azide (1.0 mmol) and DMSO (2.0 mL) were successively added to a 10 mL a round bottom flask equipped with a stirring bar. The reaction was heated at 80 °C for 4 h until the substrate was consumed (monitored by TLC). The reaction was poured into ice-water (15 mL). The aqueous was extacted with CH_2Cl_2 (2 × 5 mL), and the combined organic phase was dried over Na_2SO_4, filtered, and concentrated under reduced pressure. The crude product was purified by flash column chromatography to give 1-(4-azidoquinolin-2-yl)ethan-1-one.

Step 3: (4-azidoquinolin-2-yl)Ethan-1-one (0.5 mmol), sodium methoxide (3.0 mmol) and MeOH/dioxane (1/1, 3.0 mL) were successively added to a 10 mL Schlenk tube under N_2 atmosphere. The reaction was irradiated (400 W, high-pressure Hg Lamp) for 30 min until the substrate was consumed (monitored by TLC). After removal of solvent, the ice-water (2 mL) was added. The seperated aqueous was extacted with CH_2Cl_2 (2 × 5 mL), and the combined organic phase was dried over Na_2SO_4, filtered, and concentrated under reduced pressure. The crude product was purified by flash column chromatography to give 1-(5-methoxy-1*H*-benzo[*e*][1,4]diazepin-2-yl)ethan-1-one.

3. Supporting Spectra

3.1 X-Ray Crystallography

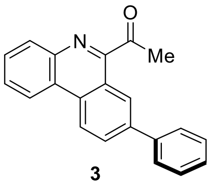

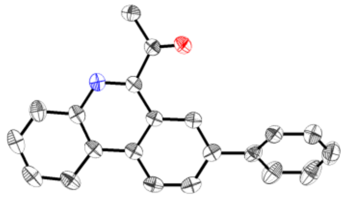

CCDC 2285580

Supplementary Table 5 Crystal data and structure refinement for 3

Empirical formula	$C_{21}H_{15}NO$
Formula weight	297.34
Temperature/K	200
Crystal system	orthorhombic
Space group	-P 2ac 2ab
a/Å	7.3527
b/Å	16.9695
c/Å	24.5823
α/°	90
β/°	90
γ/°	90
Volume/Å^3	3067.17
Z	8
Density (calculated) g/cm^3	1.288
μ/mm^{-1}	0.394
F(000)	1248.0
Crystal size/mm^3	0.32×0.24×0.17
Radiation	Ga Kα (λ = 1.34139)
2Θ range for data collection	3.128 to 57.087°
Index ranges	$-8 \le h \le 9$, $-20 \le k \le 21$, $-30 \le l \le 29$
Reflections collected	2413
Independent reflections	3148
Data/restraints/parameters	3184/0/210
Goodness-of-fit on F^2	0.970
Final R indexes [I>=2σ (I)]	R_1 = 0.0444, wR_2 = 0.1244
Final R indexes [all data]	R_1 = 0.0613, wR_2 = 0.1403
Largest diff. peak/hole / e Å^{-3}	0.247/-0.180
Displacement ellipsoids are drawn at 50% probability evel	

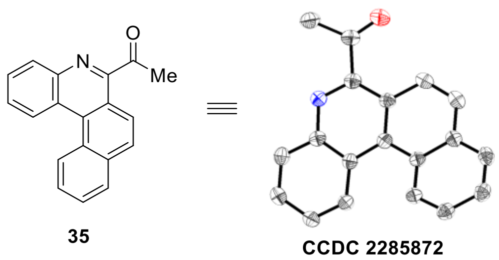

Supplementary Table 6 Crystal data and structure refinement for 35

Empirical formula	$C_{19}H_{13}NO$
Formula weight	271.30
Temperature/K	150
Crystal system	monoclinic
Space group	P 21/c
a/Å	9.4767
b/Å	18.528
c/Å	7.4466
α/°	90
β/°	92.516(6)
γ/°	90
Volume/Å^3	1306.2
Z	4
Density (calculated) g/cm^3	1.380
μ/mm^{-1}	0.427
F(000)	568
Crystal size/mm^3	0.30×0.25×0.18
Radiation	Ga Kα (λ = 1.34139)
2Θ range for data collection	4.062 to 52.214°
Index ranges	$-10 \leq h \leq 11, -21 \leq k \leq 20, -8 \leq l \leq 8$
Reflections collected	2205
Independent reflections	1788
Data/restraints/parameters	2205/0/191
Goodness-of-fit on F^2	1.133
Final R indexes [I>=2σ (I)]	R_1 = 0.0609, wR_2 = 0.1869
Final R indexes [all data]	R_1 = 0.0752, wR_2 = 0.1963
Largest diff. peak/hole / e Å^{-3}	0.267/-0.288
Displacement ellipsoids are drawn at 50% probability evel	

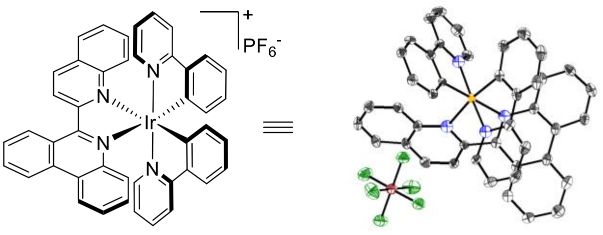

39 CCDC 2310917

Supplementary Table 7 Crystal data and structure refinement for 39

Empirical formula	$C_{44}H_{30}IrN_4F_6P$
Formula weight	951.89
Temperature/K	150
Crystal system	orthorhombic
Space group	P n a 21
a/Å	21.380
b/Å	7.919
c/Å	23.029
α/°	90
β/°	90
γ/°	90
Volume/Å^3	3899.0
Z	4
Density (calculated) g/cm^3	1.622
μ/mm^{-1}	5.016
F(000)	1872.0
Crystal size/mm^3	0.16 × 0.12 × 0.1
Radiation	Ga Kα (λ = 1.34139)
2Θ range for data collection	3.597 to 57.032°
Index ranges	-26 ≤ h ≤ 26, -9 ≤ k ≤ 9, -28 ≤ l ≤ 24
Reflections collected	43189
Independent reflections	7572
Data/restraints/parameters	7572/1/505
Goodness-of-fit on F^2	1.084
Final R indexes [I>=2σ (I)]	R_1 = 0.0261, wR_2 = 0.0581
Final R indexes [all data]	R_1 = 0.0341, wR_2 = 0.0609
Largest diff. peak/hole / e Å^{-3}	1.144/-0.927
Displacement ellipsoids are drawn at 50% probability evel	

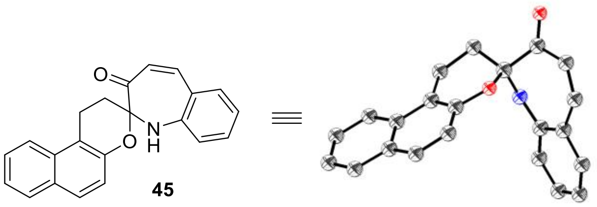

45

CCDC 2308629

Supplementary Table 8 Crystal data and structure refinement for 45

Empirical formula	$C_{22}H_{17}NO_2$
Formula weight	327.36
Temperature/K	150
Crystal system	tetragonal
Space group	P-4$2_1$c
a/Å	20.3486
b/Å	20.3486
c/Å	7.9296
α/°	90
β/°	90
γ/°	90
Volume/Å^3	3283.4
Z	8
Density (calculated) g/cm^3	1.324
μ/mm^{-1}	0.430
F(000)	1376.0
Crystal size/mm^3	0.53 × 0.42 × 0.12
Radiation	Ga Kα (λ = 1.34139)
2Θ range for data collection	5.344 to 114.162°
Index ranges	-25 ≤ h ≤ 24, -25 ≤ k ≤ 24, -9 ≤ l ≤ 9
Reflections collected	28871
Independent reflections	3356
Data/restraints/parameters	3356/0/228
Goodness-of-fit on F^2	1.114
Final R indexes [I>=2σ (I)]	R_1 = 0.0654, wR_2 = 0.1548
Final R indexes [all data]	R_1 = 0.0677, wR_2 = 0.1585
Largest diff. peak/hole / e Å^{-3}	0.55/-0.42
Displacement ellipsoids are drawn at 50% probability evel	

3.2 Characterization of starting materials

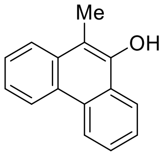

10-Methylphenanthren-9-ol. (**1a**) The title compound was prepared following the general procedure A from 1-(2-bromophenyl)ethan-1-one (99.5 mg, 0.5 mmol) and (2-formylphenyl)boronic acid (82.5 mg, 0.55 mmol). Purification was carried out by silica gel column chromatography (petroleum ether/ethyl acetate = 20/1) to afford the product as a white solid (66.6 mg, 64% yield over two steps, m.p. = 59–60 °C).

^{1}H NMR (400 MHz, DMSO-d_6) δ = 9.22 (s, 1H), 8.77 – 8.67 (m, 2H), 8.39 (dd, *J* = 7.9 Hz, *J* = 1.5 Hz, 1H), 7.95 (d, *J* = 7.7 Hz, 1H), 7.69 – 7.57 (m, 3H), 7.53 – 7.46 (m, 1H), 2.59 (s, 3H).

^{13}C NMR (100 MHz, DMSO-d_6) δ = 147.2, 133.1, 129.9, 127.1, 126.9, 126.5, 126.4, 125.9, 123.8, 123.7, 123.0, 122.8, 122.7, 112.4, 11.9.

HRMS (ESI) m/z calculated for $C_{15}H_{12}O$ $[M-H]^-$ 207.0804, found 207.0791.

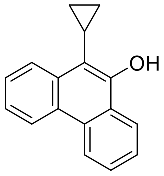

10-Cyclopropylphenanthren-9-ol. (**2a**) The title compound was prepared following the general procedure A from (2-bromophenyl)(cyclopropyl)methanone (112.5 mg, 0.5 mmol) and (2-formylphenyl)boronic acid (73.4 mg, 0.55 mmol). Purification was carried out by silica gel column chromatography (petroleum ether/ethyl acetate = 20/1) to afford the product as a white solid (59.7 mg, 51% yield over two steps, m.p. = 74–75 °C).

^{1}H NMR (400 MHz, $CDCl_3$) δ = 8.70 – 8.64 (m, 2H), 8.46 – 8.40 (m, 2H), 7.72 – 7.67 (m, 2H), 7.65 (dd, *J* = 8.2 Hz, *J* = 1.3 Hz, 1H), 7.55 (m, 1H), 6.69 (s, 1H), 1.99 (m, 1H), 1.37 – 1.33 (m, 2H), 0.86 – 0.82 (m, 2H).

^{13}C NMR (100 MHz, $CDCl_3$) δ = 149.1, 133.1, 130.8, 126.8, 126.6, 126.4, 124.9, 124.3, 123.6, 122.7, 122.6, 122.3, 112.9, 7.1, 6.6.

HRMS (ESI) m/z calculated for $C_{17}H_{13}O$ $[M-H]^-$ 233.0961, found 233.0963.

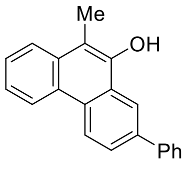

10-Methyl-7-phenylphenanthren-9-ol. (**3a**) The title compound was prepared following the general procedure A from 1-(4-bromo-[1,1'-biphenyl]-3-yl)ethan-1-one (137.6 mg, 0.5 mmol) and (2-formylphenyl)boronic acid (73.4 mg, 0.55 mmol). Purification was carried out by silica gel column chromatography (petroleum ether/ethyl acetate = 20/1) to afford the product as a white solid (75.4 mg, 53% yield over two steps, m.p. = 106–107 °C).

^{1}H NMR (400 MHz, $CDCl_3$) δ = 8.71 (d, *J* = 8.4 Hz, 1H), 8.66 (d, *J* = 8.0 Hz, 1H), 8.49 (s, 1H), 7.96 (d, *J* = 8.0 Hz, 1H), 7.90 (d, *J* = 8.3 Hz, 1H), 7.81 (d, *J* = 7.4 Hz, 2H), 7.63 (t, *J* = 7.4 Hz, 1H), 7.57 – 7.50 (m, 3H), 7.41 (t, *J* = 7.2 Hz, 1H), 5.27 (s, 1H), 2.60 (s, 3H).

^{13}C NMR (100 MHz, $CDCl_3$) δ = 146.3, 141.0, 139.1, 132.6, 129.3, 128.9, 127.4, 126.9, 126.8, 126.6, 125.8, 125.6, 124.1, 123.6, 123.2, 122.9, 120.0, 111.0, 11.3.

HRMS (ESI) m/z calculated for $C_{21}H_{15}O$ $[M-H]^-$ 283.1117, found 283.1112.

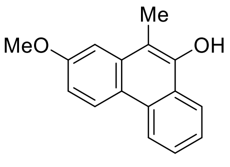

2-Methoxy-10-methylphenanthren-9-ol. (**4a**) The title compound was prepared following the general procedure A from 1-(2-bromophenyl)ethan-1-one (99.5 mg, 0.5 mmol) and (2-formyl-5-methoxyphenyl)boronic acid (98.9 mg, 0.55 mmol). Purification was carried out by silica gel column chromatography (petroleum ether/ethyl acetate = 15/1) to afford the product as a yellow solid (76.3 mg, 67% yield over two steps, m.p. = 90–92 °C).

^{1}H NMR (400 MHz, $CDCl_3$) δ = 8.55 – 8.51 (m, 2H), 8.23 – 8.16 (m, 1H), 7.64 – 7.53 (m, 2H), 7.26 (d, *J* = 2.5 Hz, 1H), 7.15 (dd, *J* = 9.0 Hz, *J* = 2.5 Hz, 1H), 5.30 (s, 1H), 3.96 (s, 3H), 2.50 (s, 3H).

^{13}C NMR (100 MHz, $CDCl_3$) δ = 158.6, 146.7, 134.2, 130.2, 126.4, 125.5, 124.4, 124.3, 122.1, 121.6, 120.9, 113.3, 110. 5, 105.1, 55.3, 11.3.

HRMS (ESI) m/z calculated for $C_{16}H_{13}O_2$ $[M-H]^-$ 237.0910, found 237.0916.

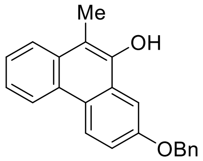

7-(benzyloxy)-10-Methylphenanthren-9-ol. (**5a**) The title compound was prepared following the general procedure A from 1-(5-(benzyloxy)-2-bromophenyl)ethan-1-one (152.6 mg, 0.5 mmol) and (2-formylphenyl)boronic acid (73.4 mg, 0.55 mmol). Purification was carried out by silica gel column chromatography (petroleum ether/ethyl acetate = 20/1) to afford the product as a white solid (105.3 mg, 67% yield over two steps, m.p. = 107–110 °C).

^{1}H NMR (400 MHz, $CDCl_3$) δ = 8.59 – 8.54 (m, 2H), 7.93 (d, *J* = 8.1 Hz, 1H), 7.75 (d, *J* = 2.3 Hz, 1H), 7.59 – 7.49 (m, 4H), 7.43 (t, *J* = 7.6 Hz, 2H), 7.40 – 7.32 (m, 2H), 5.25 (s, 2H), 5.22 – 5.10 (br, 1H), 2.57 (s, 3H).

^{13}C NMR (100 MHz, $CDCl_3$) δ = 157.6, 145.8, 136.9, 131.4, 128.6, 128.0, 127.7, 126.8, 125.9, 124.7, 124.3, 124.1, 123.5, 122.3, 118.8, 117.2, 111.2, 103.8, 70.1, 11.3.

HRMS (ESI) m/z calculated for $C_{22}H_{17}O_2$ $[M-H]^-$ 313.1223, found 313.1216.

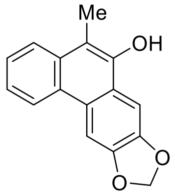

5-Methylphenanthro[2,3-*d*][1,3]dioxol-6-ol. (**6a**) The title compound was prepared following the general procedure A from 1-(6-bromobenzo[*d*][1,3]dioxol-5-yl)ethan-1-one (121.5 mg, 0.5 mmol) and (2-formylphenyl)boronic acid (73.4 mg, 0.55 mmol). Purification was carried out by silica gel column chromatography (petroleum ether/ethyl acetate = 15/1) to afford the product as a white solid (54.2 mg, 43% yield over two steps, m.p. = 128–129 °C).

^{1}H NMR (400 MHz, DMSO-d_6) δ = 9.09 (s, 1H), 8.58 (d, *J* = 7.9 Hz, 1H), 8.24 (s, 1H), 7.92 – 7.84 (m, 1H), 7.68 (s, 1H), 7.55 – 7.48 (m, 1H), 7.45 – 7.40 (m, 1H), 6.17 (s, 2H), 2.52 (s, 3H).

^{13}C NMR (100 MHz, DMSO-d_6) δ = 147.7, 147.5, 147.1, 132.3, 126.2, 126.2, 125.9, 123.7, 123.6, 123.2, 123.1, 111.3, 101.6, 101.3, 100.4, 11.9.

HRMS (ESI) m/z calculated for $C_{16}H_{11}O_3$ $[M-H]^-$ 251.0703, found 251.0693.

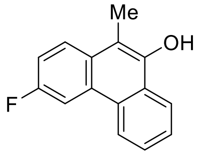

3-Fluoro-10-methylphenanthren-9-ol. (**7a**) The title compound was prepared following the general procedure A from 1-(2-bromophenyl)ethan-1-one (99.5 mg, 0.5 mmol) and (5-fluoro-2-formylphenyl)boronic acid (92.4 mg, 0.55 mmol). Purification was carried out by silica gel column chromatography (petroleum ether/ethyl acetate = 20/1) to afford the product as a white solid (66.7 mg, 59% yield over two steps, m.p. = 80–82 °C).

^{1}H NMR (400 MHz, $CDCl_3$) δ = 8.49 – 8.45 (m, 1H), 8.22 – 8.19 (m, 2H), 7.84 (dd, *J* = 9.0 Hz, *J* = 5.8 Hz, 1H), 7.66 – 7.59 (m, 2H), 7.32 (td, *J* = 8.8 Hz, *J* = 2.4 Hz, 1H), 5.18 (s, 1H), 2.51 (s, 3H).

^{13}C NMR (100 MHz, $CDCl_3$) δ = 161.3, 158.9, 145.5, 129.4 (d, *J* = 3.5 Hz), 129.2, 128.1 (d, *J* = 7.9 Hz), 127.1, 126.4, 125.9, 125.5 (d, *J* = 8.5 Hz), 122.7, 121.8, 115.6 (d, *J* = 23.1 Hz), 110.7, 107.9 (d, *J* = 22.0 Hz), 11.3.

^{19}F NMR (376 MHz, $CDCl_3$) δ = −117.85 (s, 1F)

HRMS (ESI) m/z calculated for $C_{15}H_{11}OF$ $[M-H]^-$ 225.0710, found 225.0708.

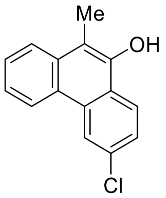

6-Chloro-10-methylphenanthren-9-ol. (**8a**) The title compound was prepared following the general procedure A from 1-(2-bromo-4-chlorophenyl)ethan-1-one (111.7 mg, 0.5 mmol) and (2-formylphenyl)boronic acid (73.4 mg, 0.55 mmol). Purification was carried out by silica gel column chromatography (petroleum ether/ethyl acetate = 20/1) to afford the product as a white solid (72.8 mg, 60% yield over two steps, m.p. = 91–92 °C).

^{1}H NMR (400 MHz, DMSO-*d*$_6$) δ = 8.55 – 8.51 (m, 2H), 8.23 – 8.16 (m, 1H), 7.64 – 7.53 (m, 2H), 7.26 (d, *J* = 2.5 Hz, 1H), 7.15 (dd, *J* = 9.0 Hz, *J* = 2.5 Hz, 1H), 5.30 (s, 1H), 3.96 (s, 3H), 2.50 (s, 3H).

^{13}C NMR (100 MHz, DMSO-*d*$_6$) δ = 158.6, 146.7, 134.2, 130.2, 126.4, 125.5, 124.4, 124.3, 122.1, 121.6, 120.9, 113.3, 110.5, 105.1, 55.3, 11.3.

HRMS (ESI) m/z calculated for $C_{15}H_{11}OCl$ $[M-H]^-$ 241.0415, found 241.0401.

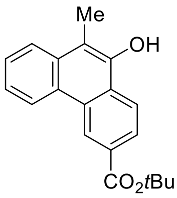

***tert*-Butyl 10-hydroxy-9-methylphenanthrene-3-carboxylate**. (**9a**) The title compound was prepared following the general procedure A from *tert*-butyl 4-acetyl-3-bromobenzoate (149.6 mg, 0.5 mmol) and (2-formylphenyl)boronic acid (73.4 mg, 0.55 mmol). Purification was carried out by silica gel column chromatography (petroleum ether/ethyl acetate = 15/1) to afford the product as a white solid (78.6 mg, 51% yield over two steps, m.p. = 110–112 °C).

^{1}H NMR (400 MHz, $CDCl_3$) δ = 9.34 (d, *J* = 1.7 Hz, 1H), 8.70 (d, *J* = 8.2 Hz, 1H), 8.29 (d, *J* = 8.6 Hz, 1H), 8.17 (dd, *J* = 8.6 Hz, *J* = 1.7 Hz, 1H), 7.94 – 7.92 (m, 1H), 7.66 – 7.58 (m, 1H), 7.54 – 7.52 (m, 1H), 5.71 (s, 1H), 2.59 (s, 3H), 1.71 (s, 9H).

^{13}C NMR (100 MHz, $CDCl_3$) δ = 166.3, 146.0, 132.7, 129.5, 129.3, 128.1, 127.3, 126.8, 126.4, 124.7, 124.4, 123.6, 122.9, 121.9, 113.6, 81.4, 28.3, 11.4.

HRMS (ESI) m/z calculated for $C_{20}H_{19}O_3$ $[M{-}H]^-$ 307.1329, found 307.1339.

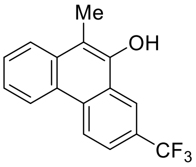

10-Methyl-7-(trifluoromethyl)phenanthren-9-ol. (**10a**) The title compound was prepared following the general procedure A from 1-(2-bromo-5-(trifluoromethyl)phenyl)ethan-1-one (133.5 mg, 0.5 mmol) and (2-formylphenyl)boronic acid (73.4 mg, 0.55 mmol). Purification was carried out by silica gel column chromatography (petroleum ether/ethyl acetate = 20/1) to afford the product as a white solid (74.6 mg, 54% yield over two steps, m.p. = 68–71 ^{o}C).

^{1}H NMR (400 MHz, $CDCl_3$) δ = 8.65 (d, *J* = 8.7 Hz, 1H), 8.58 (d, *J* = 9.4 Hz, 2H), 7.92 (d, *J* = 8.3 Hz, 1H), 7.78 (dd, *J* = 8.7 Hz, *J* = 2.0 Hz, 1H), 7.65 (m, 1H), 7.55 (m, 1H), 5.27 (s, 1H), 2.53 (s, 3H).

^{13}C NMR (100 MHz, $CDCl_3$) δ = 145.9, 133.2, 132.0, 127.9, 125.9, 125.8, 124.9, 124.5, 123.7, 123.2, 123.1, 122.2, 122.1, 119.9 (q, *J* = 43.0 Hz), 111.8, 11.2.

^{19}F NMR (376 MHz, $CDCl_3$) δ = −61.76 (s, 3F)

HRMS (ESI) m/z calculated for $C_{16}H_{14}OF_3$ $[M{-}H]^-$ 275.0678, found 275.0666.

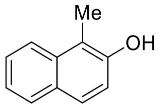

1-Methylnaphthalen-2-ol. (**11a**) The title compound was prepared following the literature from 1-bromonaphthalen-2-ol (223.1 mg, 1.0 mmol)[2]. Purification was carried out by silica gel column chromatography (petroleum ether/ethyl acetate = 30/1) to afford the product as a white solid (102.8 mg, 65% yield over three steps, m.p. = 75–76 ^{o}C).

^{1}H NMR (400 MHz, $CDCl_3$) δ = 7.99 (d, *J* = 8.6 Hz, 1H), 7.85 (d, *J* = 8.2 Hz, 1H), 7.67 (d, *J* = 8.8 Hz, 1H), 7.61 – 7.55 (m, 1H), 7.47 – 7.40 (m, 1H), 7.10 (dd, *J* = 8.8 Hz, *J* = 1.1 Hz, 1H), 5.15 (s, 1H), 2.60 (s, 3H).

^{13}C NMR (100 MHz, $CDCl_3$) δ = 150.3, 133.8, 129.2, 128.4, 127.3, 126.3, 123.1, 117.5, 115.4, 10.4.

HRMS (ESI) m/z calculated for $C_{11}H_9O$ $[M{-}H]^-$ 157.0648, found 157.0646.

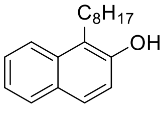

1-Octylnaphthalen-2-ol. (**12a**) The title compound was prepared following the literature from 1-bromonaphthalen-2-ol (223.1 mg, 1.0 mmol)[2]. Purification was carried out by silica gel column chromatography (petroleum ether/ethyl acetate = 30/1) to afford the product as a yellow oil (141.0 mg, 55% yield over three steps).

^{1}H NMR (400 MHz, $CDCl_3$) δ = 7.94 (d, *J* = 8.6 Hz, 1H), 7.78 (d, *J* = 8.1 Hz, 1H), 7.62 (dd, *J* = 8.7 Hz, *J* = 1.9 Hz, 1H), 7.52 – 7.47 (m, 1H), 7.34 (d, *J* = 15.1 Hz, 1H), 7.06 (dd, *J* = 8.8 Hz, *J* = 1.9 Hz, 1H), 5.04 (s, 1H), 3.05 – 3.00 (m, 2H), 1.71 – 1.63 (m, 2H), 1.49 – 1.45 (m, 2H), 1.37 – 1.28 (m, 8H), 0.90 (t, *J* = 5.8 Hz, 3H).

^{13}C NMR (100 MHz, $CDCl_3$) δ = 150.3, 133.1, 129.4, 128.6, 127.5, 126.2, 123.0, 122.9, 120.3, 117.6, 31.9, 30.0, 29.8, 29.6, 29.3, 25.1, 22.7, 14.1.

HRMS (ESI) m/z calculated for $C_{18}H_{23}O$ $[M-H]^-$ 255.1743, found 255.1756.

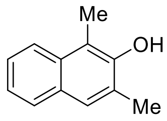

1,3-Dimethylnaphthalen-2-ol. (**13a**) The title compound was prepared following the literature from 1-bromonaphthalen-2-ol (223.1 mg, 1.0 mmol)[2]. Purification was carried out by silica gel column chromatography (petroleum ether/ethyl acetate = 30/1) to afford the product as a pale yellow solid (70.6 mg, 41% yield over four steps, m.p. = 86–88 °C).

^{1}H NMR (400 MHz, $CDCl_3$) δ = 7.96 (dd, *J* = 8.5 Hz, *J* = 1.1 Hz, 1H), 7.83 – 7.78 (m, 1H), 7.59 – 7.52 (m, 2H), 7.44 (m, 1H), 5.05 (s, 1H), 2.60 (s, 3H), 2.50 (s, 3H).

^{13}C NMR (100 MHz, $CDCl_3$) δ = 149.9, 132.5, 129.0, 127.6, 126.9, 125.5, 125.3, 123.1, 122.9, 114.5, 16.8, 10.5.

HRMS (ESI) m/z calculated for $C_{12}H_{11}O$ $[M-H]^-$ 171.0804, found 171.0804.

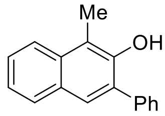

1-Methyl-3-phenylnaphthalen-2-ol. (**14a**) The title compound was prepared following the general procedure H from 3-bromo-1-methylnaphthalen-2-ol (118.6 mg, 0.5 mmol)[2]. Purification was carried out by silica gel column chromatography (petroleum ether/ethyl acetate = 30/1) to afford the product as a white solid (85.5 mg, 73% yield, m.p. = 84–87 °C).

^{1}H NMR (400 MHz, $CDCl_3$) δ = 8.02 (dq, *J* = 8.5 Hz, *J* = 0.9 Hz, 1H), 7.86 – 7.82 (m, 1H), 7.67 (s, 1H), 7.62 – 7.58 (m, 3H), 7.58 – 7.54 (m, 2H), 7.53 – 7.48 (m, 1H), 7.42 (m, 1H), 5.39 (s, 1H), 2.69 (s, 3H).

^{13}C NMR (100 MHz, $CDCl_3$) δ = 147.9, 137.2, 133.5, 130.0, 129.3, 129.3, 128.7, 128.4, 128.2, 127.1, 126.2, 123.4, 123.1, 116.2, 10.8.

HRMS (ESI) m/z calculated for $C_{17}H_{13}O$ $[M-H]^-$ 233.0961, found 233.0953.

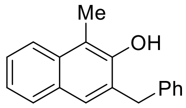

3-Benzyl-1-methylnaphthalen-2-ol. (**15a**) The title compound was prepared following the literature from 1-bromonaphthalen-2-ol (223.1 mg, 1.0 mmol)[3]. Purification was carried out by silica gel column chromatography (petroleum ether/ethyl acetate = 30/1) to afford the product as a brown solid (101.8 mg, 41% yield over four steps, m.p. = 46–49 °C).

^{1}H NMR (400 MHz, $CDCl_3$) δ = 7.92 (dd, *J* = 8.5 Hz, *J* = 1.0 Hz, 1H), 7.80 – 7.76 (m, 1H), 7.55 (s, 1H), 7.51 (m, 1H), 7.41 – 7.28 (m, 6H), 4.90 (s, 1H), 4.21 (s, 2H), 2.53 (s, 3H).

^{13}C NMR (100 MHz, $CDCl_3$) δ = 149.8, 139.3, 132.9, 129.0, 128.9, 128.8, 128.6, 128.4, 128.0, 127.5, 125.7, 123.3, 122.9, 115.7, 37.7, 10.6.

HRMS (ESI) m/z calculated for $C_{18}H_{15}O$ $[M-H]^-$ 247.1117, found 247.1104.

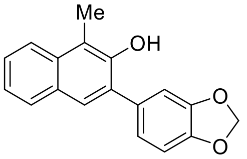

3-(benzo[*d*][1,3]dioxol-5-yl)-1-Methylnaphthalen-2-ol. (**16a**) The title compound was prepared following the general procedure B from 3-bromo-1-methylnaphthalen-2-ol (118.6 mg, 0.5 mmol). Purification was carried out by silica gel column chromatography (petroleum ether/ethyl acetate = 20/1) to afford the product as a white solid (84.9 mg, 61% yield, m.p. = 84–86 °C).

^{1}H NMR (400 MHz, $CDCl_3$) δ = 7.95 (dd, *J* = 8.5 Hz, *J* = 1.0 Hz, 1H), 7.80 – 7.75 (m, 1H), 7.58 (s, 1H), 7.51 (m, 1H), 7.36 (m, 1H), 7.03 – 6.95 (m, 3H), 6.05 (s, 2H), 5.41 (s, 1H), 2.62 (s, 3H).

^{13}C NMR (100 MHz, $CDCl_3$) δ = 148.5, 147.9, 147.7, 133.4, 130.8, 129.7, 128.6, 128.3, 126.9, 126.2, 123.4, 123.1, 122.6, 116.1, 109.9, 109.0, 101.4, 10.9.

HRMS (ESI) m/z calculated for $C_{18}H_{13}O_3$ $[M-H]^-$ 277.0859, found 277.0863.

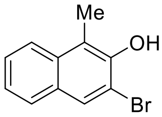

3-Bromo-1-methylnaphthalen-2-ol. (**17a**) The title compound was prepared following the literature from 1-methylnaphthalen-2-ol (158.2 mg, 1.0 mmol)[1]. Purification was carried out by silica gel column chromatography (petroleum ether/ethyl acetate = 30/1) to afford the product as a yellow solid (111.4 mg, 47% yield over three steps, m.p. = 60–61 °C).

^{1}H NMR (400 MHz, $CDCl_3$) δ = 7.93 – 7.88 (m, 2H), 7.70 – 7.66 (m, 1H), 7.51 (m, 1H), 7.37 (m, 1H), 5.74 (s, 1H), 2.63 (s, 3H)

^{13}C NMR (100 MHz, $CDCl_3$) δ = 146.5, 133.2, 129.3, 128.7, 127.4, 126.5, 123.9, 123.4, 117.7, 112.7, 11.6.

HRMS (ESI) m/z calculated for $C_{11}H_8OBr$ $[M-H]^-$ 234.9753, found 234.9740.

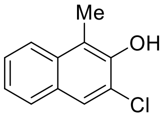

3-Chloro-1-methylnaphthalen-2-ol. (**18a**) The title compound was prepared following the literature from 1-methylnaphthalen-2-ol (158.2 mg, 1.0 mmol)[1]. Purification was carried out by silica gel column chromatography (petroleum ether/ethyl acetate = 30/1) to afford the product as a yellow solid (107.9 mg, 56% yield over three steps, m.p. = 48–51 °C).

^{1}H NMR (400 MHz, $CDCl_3$) δ = 7.90 (dd, *J* = 8.5 Hz, *J* = 1.0 Hz, 1H), 7.73 (s, 1H), 7.71 – 7.66 (m, 1H), 7.51 (ddd, *J* = 8.4 Hz, *J* = 6.8 Hz, *J* = 1.4 Hz, 1H), 7.39 (ddd, *J* = 8.1 Hz, *J* = 6.8 Hz, *J* = 1.2 Hz, 1H), 5.81 (s, 1H), 2.62 (s, 3H).

^{13}C NMR (100 MHz, $CDCl_3$) δ = 145.9, 132.7, 128.7, 127.5, 126.4, 125.2, 124.0, 123.3, 121.6, 117.8, 11.3.

HRMS (ESI) m/z calculated for $C_{11}H_8OCl$ $[M-H]^-$ 191.0258, found 191.0257.

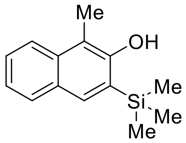

1-Methyl-3-(trimethylsilyl)naphthalen-2-ol. (**19a**) The title compound was prepared following the literature from 1-bromonaphthalen-2-ol (223.1 mg, 1.0 mmol)[4]. Purification was carried out by silica gel column chromatography

(petroleum ether/ethyl acetate = 70/1) to afford the product as a white solid (71.4 mg, 31% yield over four steps, m.p. = 67–70 °C).

^{1}H NMR (400 MHz, $CDCl_3$) δ = 7.90 (dd, *J* = 8.5 Hz, *J* = 1.1 Hz, 1H), 7.85 – 7.82 (m, 2H), 7.53 (m, 1H), 7.38 (m, 1H), 5.10 (s, 1H), 2.52 (s, 3H), 0.46 (s, 9H).

^{13}C NMR (100 MHz, $CDCl_3$) δ = 155.1, 134.3, 134.2, 128.9, 128.7, 128.5, 126.6, 122.9, 122.7, 112.4, 10.4, -0.7.

HRMS (ESI) m/z calculated for $C_{17}H_{17}OSi$ $[M-H]^-$ 229.1043, found 229.1029.

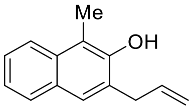

3-Allyl-1-methylnaphthalen-2-ol. (**20a**) The title compound was prepared following the literature from 1-bromonaphthalen-2-ol (223.1 mg, 1.0 mmol)[2] Purification was carried out by silica gel column chromatography (petroleum ether/ethyl acetate = 30/1) to afford the product as a yellow oil (67.4 mg, 34% yield over four steps).

^{1}H NMR (400 MHz, $CDCl_3$) δ = 7.92 (dq, *J* = 8.5 Hz, *J* = 0.9 Hz, 1H), 7.78 – 7.73 (m, 1H), 7.51 (s, 1H), 7.48 (m, 1H), 7.36 (m, 1H), 6.14 (m, 1H), 5.28 (dq, *J* = 5.5 Hz, *J* = 1.7 Hz, 1H), 5.25 (t, *J* = 1.8 Hz, 1H), 5.22 (s, 1H), 3.62 (dq, *J* = 6.5 Hz, *J* = 1.4 Hz, 2H), 2.57 (s, 3H).

^{13}C NMR (100 MHz, $CDCl_3$) δ = 150.1, 136.4, 132.9, 129.0, 127.9, 127.0, 126.9, 125.6, 123.2, 123.0, 117.1, 116.0, 36.3, 10.6.

HRMS (ESI) m/z calculated for $C_{14}H_{13}O$ $[M-H]^-$ 197.0956, found197.0961.

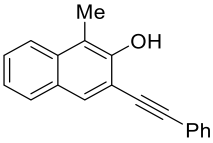

1-Methyl-3-(phenylethynyl)naphthalen-2-ol. (**21a**) The title compound was prepared following the literature from 1-bromonaphthalen-2-ol (223.1 mg, 1.0 mmol)[2]. Purification was carried out by silica gel column chromatography (petroleum ether/ethyl acetate = 50/1) to afford the product as a yellow solid (170.5 mg, 66% yield over four steps, m.p. = 56–61 °C).

^{1}H NMR (400 MHz, $CDCl_3$) δ = 7.93 – 7.89 (m, 2H), 7.76 (dd, *J* = 8.2 Hz, *J* = 1.2 Hz, 1H), 7.61 (m, 2H), 7.52 (m, 1H), 7.43 – 7.39 (m, 3H), 7.36 (td, *J* = 7.4 Hz, *J* = 6.8 Hz, *J* = 0.9 Hz, 1H), 6.05 (s, 1H), 2.61 (s, 3H).

^{13}C NMR (100 MHz, $CDCl_3$) δ = 149.6, 134.1, 131.7, 129.9, 128.9, 128.5, 128.3, 128.2, 127.1, 123.6, 123.2, 122.2, 115.6, 111.3, 96.4, 83.7, 10.8.

HRMS (ESI) m/z calculated for $C_{19}H_{13}O$ $[M-H]^-$ 257.0961, found 257.0960.

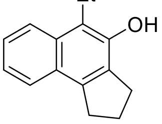

5-Ethyl-2,3-dihydro-1*H*-cyclopenta[*a*]naphthalen-4-ol. (**22a**) The title compound was prepared following the general procedure A from 1-(2-bromocyclopent-1-en-1-yl)propan-1-one (101.5 mg, 0.5 mmol) and (2-formylphenyl)boronic acid (73.4 mg, 0.55 mmol). Purification was carried out by silica gel column chromatography (petroleum ether/ethyl acetate = 30/1) to afford the product as a white solid (54.1 mg, 51% yield over two steps, m.p. = 83–82 °C).

^{1}H NMR (400 MHz, $CDCl_3$) δ = 7.95 (d, *J* = 8.5 Hz, 1H), 7.76 (dd, *J* = 8.4 Hz, *J* = 1.3 Hz, 1H), 7.46 (m, 1H), 7.36 (m,

1H), 4.79 (s, 1H), 3.30 (dd, J = 8.2 Hz, J = 7.0 Hz, 2H), 3.11 – 3.05 (m, 4H), 2.30 (p, J = 7.5 Hz, 2H), 1.30 (t, J = 7.6 Hz, 4H).

^{13}C NMR (100 MHz, $CDCl_3$) δ = 147.7, 139.6, 132.5, 130.4, 126.4, 125.3, 125.0, 123.5, 122.9, 119.8, 31.7, 30.2, 24.2, 18.1, 14.4.

HRMS (ESI) m/z calculated for $C_{15}H_{15}O$ $[M-H]^-$ 211.1117, found 211.1107.

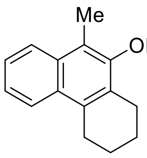

10-Methyl-5,6,7,8-tetrahydrophenanthren-9-ol. (**23a**) The title compound was prepared following the general procedure A from 1-(2-bromocyclopent-1-en-1-yl)propan-1-one (101.5 mg, 0.5 mmol) and (2-formylphenyl)boronic acid (73.4 mg, 0.55 mmol). Purification was carried out by silica gel column chromatography (petroleum ether/ethyl acetate = 30/1) to afford the product as a pale yellow solid (62.6 mg, 59% yield over two steps, m.p. = 77–78 °C).

^{1}H NMR (400 MHz, $CDCl_3$) δ = 7.98 (d, J = 8.4 Hz, 1H), 7.95 (d, J = 8.5 Hz, 1H), 7.54 – 7.48 (m, 1H), 7.44 – 7.39 (m, 1H), 4.93 (s, 1H), 3.15 (t, J = 5.6 Hz, 2H), 2.80 (td, J = 5.3 Hz, J = 2.7 Hz, 2H), 2.56 (s, 3H), 1.98 – 1.94 (m, 4H).

^{13}C NMR (100 MHz, $CDCl_3$) δ = 149.3, 131.9, 131.3, 127.9, 125.1, 124.7, 123.3, 123.2, 122.9, 111.8, 25.8, 24.0, 22.7, 22.4, 10.5.

HRMS (ESI) m/z calculated for $C_{15}H_{15}O$ $[M-H]^-$ 211.1117, found 211.1103.

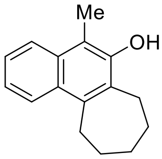

5-Methyl-8,9,10,11-tetrahydro-7*H*-cyclohepta[*a*]naphthalen-6-ol. (**24a**) The title compound was prepared following the general procedure A from 1-(2-bromocyclohept-1-en-1-yl)ethan-1-one (108.6 mg, 0.5 mmol) and (2-formylphenyl)boronic acid (73.4 mg, 0.55 mmol). Purification was carried out by silica gel column chromatography (petroleum ether/ethyl acetate = 30/1) to afford the product as a white solid (56.6 mg, 50% yield over two steps, m.p. = 88–90 °C).

^{1}H NMR (400 MHz, $CDCl_3$) δ = 8.08 (d, J = 8.5 Hz, 1H), 7.90 (d, J = 8.4 Hz, 1H), 7.46 – 7.43 (m, 1H), 7.38 – 7.34 (m, 1H), 4.96 (s, 1H), 3.30 – 3.24 (m, 2H), 3.13 – 3.08 (m, 2H), 2.54 (s, 3H), 1.94 – 1.88 (m, 2H), 1.73 – 1.66 (m, 4H).

^{13}C NMR (100 MHz, $CDCl_3$) δ = 148.6, 138.9, 132.3, 130.9, 127.2, 125.1, 123.9, 123.5, 122.9, 112.0, 32.1, 27.9, 26.7, 26.3, 11.1.

HRMS (ESI) m/z calculated for $C_{16}H_{17}O$ $[M-H]^-$ 225.1274, found 225.1273.

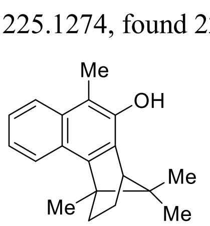

4,9,11,11-Tetramethyl-1,2,3,4-tetrahydro-1,4-methanophenanthren-10-ol. (**25a**) The title compound was prepared following the general procedure A from 1-(3-bromo-4,7,7-trimethylbicyclo[2.2.1]hept-2-en-2-yl)ethan-1-one (128.6 mg, 0.5 mmol) and (2-formylphenyl)boronic acid (73.4 mg, 0.55 mmol). Purification was carried out by silica gel column chromatography (petroleum ether/ethyl acetate = 20/1) to afford the product as a yellow liquid (57.3 mg,

43% yield over two steps).

^{1}H NMR (400 MHz, $CDCl_3$) δ = 8.29 (d, *J* = 8.5 Hz, 1H), 8.00 (d, *J* = 8.6 Hz, 1H), 7.45 (t, *J* = 7.6 Hz, 1H), 7.36 (t, *J* = 7.6 Hz, 1H), 4.96 (s, 1H), 3.10 (d, *J* = 3.8 Hz, 1H), 2.61 (s, 3H), 2.17 (m, 1H), 1.96 (m, 1H), 1.79 (s, 3H), 1.34 (m, 1H), 1.16 (m, 1H), 1.05 (s, 3H), 0.72 (s, 3H).

^{13}C NMR (100 MHz, $CDCl_3$) δ = 146.2, 142.9, 134.3, 133.1, 125.5, 124.3, 124.1, 123.7, 122.3, 113.1, 58.8, 56.2, 48.9, 33.2, 26.0, 20.1, 19.4, 15.4, 10.8.

HRMS (ESI) m/z calculated for $C_{19}H_{21}O$ $[M-H]^-$ 265.1587, found 265.1586.

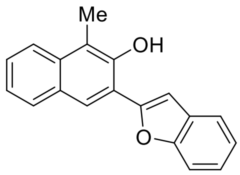

3-(benzofuran-2-yl)-1-Methylnaphthalen-2-ol. (**26a**) The title compound was prepared following the general procedure B from 3-bromo-1-methylnaphthalen-2-ol (118.6 mg, 0.5 mmol). Purification was carried out by silica gel column chromatography (petroleum ether/ethyl acetate = 10/1) to afford the product as a pale yellow solid (91.6 mg, 67% yield, m.p. = 91–93 °C).

^{1}H NMR (400 MHz, DMSO-*d*$_6$) δ = 9.40 (s, 1H), 8.38 (s, 1H), 7.97 (dd, *J* = 8.3 Hz, *J* = 1.2 Hz, 1H), 7.91 (d, *J* = 8.5 Hz, 1H), 7.73 (dd, *J* = 7.8 Hz, *J* = 1.3 Hz, 1H), 7.69 – 7.64 (m, 2H), 7.54 – 7.46 (m, 1H), 7.41 – 7.32 (m, 2H), 7.27 (t, *J* = 7.4 Hz, 1H), 2.58 (s, 3H).

^{13}C NMR (100 MHz, DMSO-*d*$_6$) δ = 153.6, 152.7, 149.1, 142.9, 133.2, 129.4, 128.9, 128.3, 126.9, 124.8, 124.1, 123.7, 123.1, 121.5, 120.5, 117.7, 110.9, 107.1, 11.6.

HRMS (ESI) m/z calculated for $C_{19}H_{13}O_2$ $[M-H]^-$ 273.0910, found 273.0894.

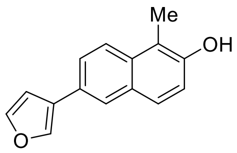

6-(furan-3-yl)-1-Methylnaphthalen-2-ol. (**27a**) The title compound was prepared following the general procedure C from 1-methylnaphthalen-2-ol (79.1 mg, 0.5 mmol). Purification was carried out by silica gel column chromatography (petroleum ether/ethyl acetate = 10/1) to afford the product as a pale brown solid (68.4 mg, 61% yield over two steps, m.p. = 83–85 °C).

^{1}H NMR (400 MHz, $CDCl_3$) δ = 9.54 (s, 1H), 8.23 (s, 1H), 7.98 (d, *J* = 2.0 Hz, 1H), 7.84 (d, *J* = 8.8 Hz, 1H), 7.76 (t, *J* = 1.8 Hz, 1H), 7.70 (dd, *J* = 8.8 Hz, *J* = 1.8 Hz, 1H), 7.62 (d, *J* = 8.7 Hz, 1H), 7.19 (dd, *J* = 8.9 Hz, *J* = 2.1 Hz, 1H), 7.05 (d, *J* = 2.1 Hz, 1H), 2.42 (s, 3H)

^{13}C NMR (100 MHz, $CDCl_3$) δ = 152.3, 144.4, 139.2, 132.9, 128.3, 126.8, 126.1, 125.9, 124.4, 124.2, 123.5, 118.6, 114.9, 108.8, 10.6.

HRMS (ESI) m/z calculated for $C_{15}H_{11}O_2$ $[M-H]^-$ 223.0754, found 223.0754.

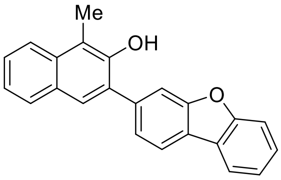

3-(dibenzo[*b*,*d*]furan-3-yl)-1-Methylnaphthalen-2-ol. (**28a**) The title compound was prepared following the general procedure B from 3-bromo-1-methylnaphthalen-2-ol (118.6 mg, 0.5 mmol). Purification was carried out by silica gel column chromatography (petroleum ether/ethyl acetate = 10/1) to afford the product as a white solid (118.4 mg, 73% yield, m.p. = 103–104 ^{o}C).

^{1}H NMR (400 MHz, $CDCl_3$) δ = 8.08 (d, *J* = 7.9 Hz, 1H), 8.00 (t, *J* = 8.8 Hz, 2H), 7.82 (dd, *J* = 8.2 Hz, *J* = 1.3 Hz, 1H), 7.77 (d, *J* = 1.3 Hz, 1H), 7.70 (s, 1H), 7.63 (d, *J* = 8.3 Hz, 1H), 7.53 (m, 3H), 7.43 – 7.37 (m, 2H), 5.46 (s, 1H), 2.66 (s, 3H).

^{13}C NMR (100 MHz, $CDCl_3$) δ = 156.6, 156.5, 147.8, 136.3, 133.6, 129.8, 128.7, 128.5, 127.5, 127.4, 126.3, 124.1, 123.8, 123.7, 123.5, 123.1, 122.9, 121.3, 120.8, 116.4, 112.5, 111.8, 10.9.

HRMS (ESI) m/z calculated for $C_{23}H_{15}O_2$ $[M-H]^-$ 323.1067, found 323.1068.

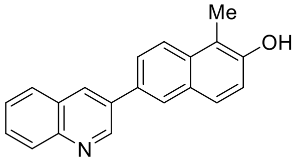

1-Methyl-6-(quinolin-3-yl)naphthalen-2-ol. (**29a**) The title compound was prepared following the general procedure C from 1-methyl-2-naphthol (79.1 mg, 0.5 mmol). Purification was carried out by silica gel column chromatography (petroleum ether/ethyl acetate = 5/1) to afford the product as a orange solid (75.6 mg, 53% yield over two steps, m.p. = 97–100 ^{o}C).

^{1}H NMR (400 MHz, DMSO-*d*$_6$) δ = 9.70 (s, 1H), 9.43 – 9.38 (m, 1H), 8.78 – 8.73 (m, 1H), 8.35 (s, 1H), 8.07 (d, *J* = 8.4 Hz, 2H), 8.00 (d, *J* = 5.4 Hz, 2H), 7.77 (dt, *J* = 8.4 Hz, *J* = 3.2 Hz, 2H), 7.68 – 7.63 (m, 1H), 7.25 (dd, *J* = 8.9 Hz, *J* = 1.1 Hz, 1H), 2.46 (s, 3H).

^{13}C NMR (100 MHz, DMSO-*d*$_6$) δ = 153.0, 149.6, 146.5, 133.5, 132.9, 132.6, 130.6, 129.5, 128.6, 128.5, 128.4, 127.9, 127.5, 127.2, 126.6, 124.9, 124.1, 118.8, 114.8, 10.6.

HRMS (ESI) m/z calculated for $C_{20}H_{14}NO$ $[M-H]^-$ 284.1070, found 284.1088.

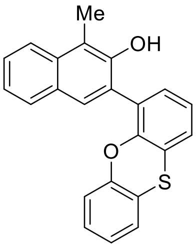

1-Methyl-3-(phenoxathiin-4-yl)naphthalen-2-ol. (**30a**) The title compound was prepared following the general procedure D from 3-bromo-1-methylnaphthalen-2-ol (118.6 mg, 0.5 mmol). Purification was carried out by silica gel column chromatography (petroleum ether/ethyl acetate = 10/1) to afford the product as a white solid (142.6 mg, 80% yield, m.p. = 91–94 ^{o}C).

^{1}H NMR (400 MHz, $CDCl_3$) δ = 7.92 – 7.88 (m, 1H), 7.69 (d, *J* = 8.1 Hz, 1H), 7.51 (s, 1H), 7.44 (ddd, *J* = 8.4 Hz , *J* = 6.8 Hz, *J* = 1.4 Hz, 1H), 7.28 (ddd, *J* = 8.1 Hz , *J* = 6.8 Hz, *J* = 1.2 Hz, 1H), 7.16 – 7.12 (m, 2H), 7.09 – 7.03 (m, 2H),

6.99 – 6.90 (m, 2H), 6.69 (dd, *J* = 7.9 Hz, *J* = 1.5 Hz, 1H), 5.15 (s, 1H), 2.57 (s, 3H)

^{13}C NMR (100 MHz, $CDCl_3$) δ = 151.9, 149.9, 148.4, 133.8, 130.4, 128.7, 128.5, 128.1, 127.9, 127.2, 127.0, 126.7, 126.3, 126.1, 125.0, 124.9, 123.4, 123.2, 122.1, 120.7, 118.2, 116.6, 10.9.

HRMS (ESI) m/z calculated for $C_{23}H_{15}O_2S$ [M−H]$^-$ 355.0787, found 355.0779.

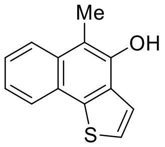

5-Methylnaphtho[1,2-*b*]thiophen-4-ol. (**31a**) The title compound was prepared following the general procedure A from 1-(2-bromothiophen-3-yl)ethan-1-one (102.5 mg, 0.5 mmol) and (2-formylphenyl)boronic acid (73.4 mg, 0.55 mmol). Purification was carried out by silica gel column chromatography (petroleum ether/ethyl acetate = 20/1) to afford the product as a yellow semisolid (72.9 mg, 68% yield over two steps).

^{1}H NMR (400 MHz, $CDCl_3$) δ = 8.32 – 8.27 (m, 1H), 8.01 – 7.94 (m, 2H), 7.58 – 7.52 (m, 2H), 7.49 (ddd, *J* = 8.1 Hz, *J* = 6.9 Hz, *J* = 1.3 Hz, 1H), 5.24 (s, 1H), 2.61 (s, 3H).

^{13}C NMR (100 MHz, $CDCl_3$) δ = 146.3, 141.0, 139.1, 132.6, 129.3, 128.9, 127.4, 126.9, 126.8, 126.6, 125.8, 125.6, 124.1, 123.6, 123.2, 122.9, 120.0, 111.0, 11.3.

HRMS (ESI) m/z calculated for $C_{13}H_9OS$ [M−H]$^-$ 213.0369, found 213.0371.

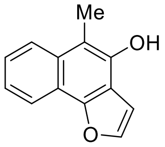

5-Methylnaphtho[1,2-*b*]furan-4-ol. (**32a**) The title compound was prepared following the general procedure A from 1-(2-bromofuran-3-yl)ethan-1-one (94.5 mg, 0.5 mmol) and (2-formylphenyl)boronic acid (73.4 mg, 0.55 mmol). Purification was carried out by silica gel column chromatography (petroleum ether/ethyl acetate = 20/1) to afford the product as a white solid (62.4 mg, 63% yield over two steps, m.p. = 111–112 ^{o}C).

^{1}H NMR (400 MHz, $CDCl_3$) δ = 8.07 (dd, *J* = 7.7 Hz, *J* = 1.1 Hz, 1H), 8.02 – 7.96 (m, 1H), 7.72 (d, *J* = 1.9 Hz, 1H), 7.55 – 7.44 (m, 2H), 7.26 (d, *J* = 1.0 Hz, 1H), 5.48 (s, 1H), 2.63 (s, 3H).

^{13}C NMR (100 MHz, $CDCl_3$) δ = 143.5, 143.4, 138.5, 131.3, 124. 9, 124.2, 123.7, 123.3, 121.7, 112.9, 106.4, 10.42.

HRMS (ESI) m/z calculated for $C_{13}H_9O_2$ [M−H]$^-$ 197.0597, found 197.0590.

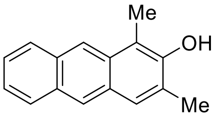

1,3-Dimethylanthracen-2-ol. (**33a**) The title compound was prepared following the procedure E from anthracen-2-ol (97.1 mg, 0.5 mmol). Purification was carried out by silica gel column chromatography (petroleum ether/ethyl acetate = 20/1) to afford the product as a yellow solid (46.7 mg, 42% yield over four steps, m.p. = 78–80 ^{o}C).

^{1}H NMR (400 MHz, DMSO-*d*$_6$) δ = 8.74 (s, 1H), 8.39 (s, 1H), 8.30 (s, 1H), 8.03 (d, *J* = 8.1 Hz, 1H), 7.96 (d, *J* = 8.0 Hz, 1H), 7.67 (s, 1H), 7.45 – 7.35 (m, 2H), 2.60 (s, 3H), 2.40 (s, 3H).

^{13}C NMR (100 MHz, DMSO-*d*$_6$) δ = 150.8, 131.7, 130.9, 129.5, 129.3, 127.9, 127.7, 126.2, 125.2, 124.9, 124.2, 120.4, 113.2, 18.1, 11.5.

HRMS (ESI) m/z calculated for $C_{16}H_{13}O$ [M−H]$^-$ 221.0961, found 221.0949.

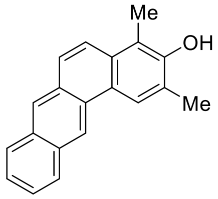

2,4-Dimethyltetraphen-3-ol. (**34a**) The title compound was prepared following the procedure F from tetraphen-3-ol (122.1 mg, 0.5 mmol). Purification was carried out by silica gel column chromatography (petroleum ether/ethyl acetate = 20/1) to afford the product as a pale yellow solid (47.7 mg, 35% yield over four steps, m.p. = 107–110 °C).

^{1}H NMR (400 MHz, Acetone-d_6) δ = 9.29 (s, 1H), 8.69 (s, 1H), 8.44 (s, 1H), 8.23 – 8.19 (m, 1H), 8.10 (d, *J* = 7.3 Hz, 1H), 7.92 – 7.83 (m, 2H), 7.77 (s, 1H), 7.57 (m, 2H), 2.65 (s, 3H), 2.61 (s, 3H).

^{13}C NMR (100 MHz, Acetone-d_6) δ = 154.3, 133.9, 133.1, 131.9, 131.6, 131.4, 131.1, 129. 9, 129.3, 127.9, 127.1, 126.9, 125.6, 124.9, 124.3, 123.7, 122.3, 120.2, 18.6, 12.4.

HRMS (ESI) m/z calculated for $C_{20}H_{15}O$ $[M{-}H]^-$ 271.1117, found 271.1118.

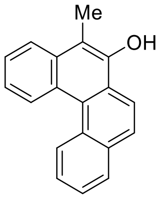

5-Methylbenzo[*c*]phenanthren-6-ol. (**35a**) The title compound was prepared following the general procedure A from 1-(1-bromonaphthalen-2-yl)ethan-1-one (124.6 mg, 0.5 mmol) and (2-formylphenyl)boronic acid (73.4 mg, 0.55 mmol). Purification was carried out by silica gel column chromatography (petroleum ether/ethyl acetate = 20/1) to afford the product as a yellow semisolid (91.7 mg, 71% yield over two steps).

^{1}H NMR (400 MHz, $CDCl_3$) δ = 9.05 (t, *J* = 7.9 Hz, 2H), 8.28 (d, *J* = 8.8 Hz, 1H), 8.07 – 8.00 (m, 2H), 7.92 (d, *J* = 8.8 Hz, 1H), 7.66 (dt, *J* = 13.4 Hz, *J* = 7.4 Hz, 3H), 7.56 (t, *J* = 7.6 Hz, 1H), 5.43 (s, 1H), 2.61 (s, 3H).

^{13}C NMR (100 MHz, $CDCl_3$) δ = 146.4, 133.3, 133.1, 129.9, 128.4, 128.3, 128.2, 127.3, 127.2, 126.4, 126.1, 125.9, 125.7, 123.9, 123.3, 119.7, 112.0, 11.3.

HRMS (ESI) m/z calculated for $C_{19}H_{13}O$ $[M{-}H]^-$ 257.0961, found 257.0960.

3.3 Characterization of carbon–nitrogen transmutation products

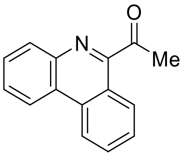

1-(phenanthridin-6-yl)Ethan-1-one. (**1**) The title compound was prepared following the general experimental procedure from 10-methylphenanthren-9-ol (20.8 mg, 0.1 mmol). Purification was carried out by silica gel column chromatography (petroleum ether/ethyl acetate = 30/1) to afford the product as a pale yellow solid (17.7 mg, 80% yield, m.p. = 107–109 °C).

^{1}H NMR (400 MHz, $CDCl_3$) δ = 8.31 (t, *J* = 8.0 Hz, 2H), 8.21 – 8.15 (m, 4H), 8.01 – 7.94 (m, 2H), 7.92 – 7.86 (m, 2H), 7.58 – 7.52 (m, 2H), 2.90 (s, 3H).

^{13}C NMR (100 MHz, $CDCl_3$) δ = 200.6, 153.1, 146.6, 141.1, 137.1, 136.9, 133.6, 132.9, 131.0, 129.9, 129.8, 128.8, 128.3, 127.7, 126.6, 126.5, 126.4, 125.5, 125.3, 118.4, 25.6.

HRMS (ESI) m/z calculated for $C_{15}H_{12}NO$ $[M+H]^+$ 222.0913, found 222.0923.

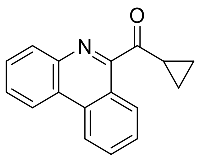

Cyclopropyl(phenanthridin-6-yl)methanone. (**2**) The title compound was prepared following the general experimental procedure from 10-cyclopropylphenanthren-9-ol (23.4 mg, 0.1 mmol). Purification was carried out by silica gel column chromatography (petroleum ether/ethyl acetate = 30/1) to afford the product as a colorless oil (10.4 mg, 42% yield).

^{1}H NMR (400 MHz, $CDCl_3$) δ = 8.73 (d, *J* = 8.4 Hz, 1H), 8.60 (d, *J* = 8.4 Hz, 1H), 8.54 (d, *J* = 7.9 Hz, 1H), 8.26 (d, *J* = 7.9 Hz, 1H), 7.82 – 7.66 (m, 4H), 3.41 – 3.37 (m, 1H), 1.44 – 1.40 (m, 2H), 1.23 – 1.19 (m, 2H).

^{13}C NMR (100 MHz, $CDCl_3$) δ = 203.7, 155.0, 142.5, 133.4, 130.9, 130.8 128.8, 128.5, 127.9, 127.8, 125.0, 122.9, 122.0, 121.9, 19.5, 13.1.

HRMS (ESI) m/z calculated for $C_{13}H_{14}NO$ $[M+H]^+$ 248.1070, found 248.1078.

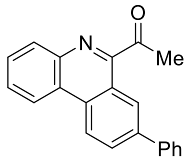

1-(8-phenylphenanthridin-6-yl)Ethan-1-one. (**3**) The title compound was prepared following the general experimental procedure from 10-methyl-7-phenylphenanthren-9-ol (28.4 mg, 0.1 mmol). Purification was carried out by silica gel column chromatography (petroleum ether/ethyl acetate = 30/1) to afford the product as a yellow solid (15.2 mg, 51% yield, m.p. = 113–117 ^{o}C).

^{1}H NMR (400 MHz, $CDCl_3$) δ = 9.16 (d, *J* = 2.0 Hz, 1H), 8.65 (d, *J* = 8.6 Hz, 1H), 8.58 – 8.54 (m, 1H), 8.23 (dd, *J* = 7.8, 1.8 Hz, 1H), 8.09 (dd, *J* = 8.6 Hz, *J* = 2.0 Hz, 1H), 7.76 (m, 4H), 7.54 – 7.45 (m, 2H), 7.45 – 7.40 (m, 1H), 2.98 (s, 3H).

^{13}C NMR (100 MHz, $CDCl_3$) δ = 202.8, 153.8, 142.3, 140.7, 140.1, 132.5, 130.9, 129.9, 128.9, 129.0, 128.8, 127.9, 127.5, 125.8, 125.2, 123.4, 122.5, 122.1, 28.6.

HRMS (ESI) m/z calculated for $C_{21}H_{16}NO$ $[M+H]^+$ 298.1226, found 298.1224.

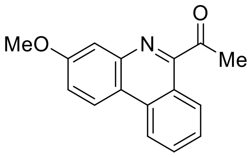

1-(3-methoxyphenanthridin-6-yl)Ethan-1-one. (**4**) The title compound was prepared following the general experimental procedure from 3-methoxy-10-methylphenanthren-9-ol (23.8 mg, 0.1 mmol). Purification was carried out by silica gel column chromatography (petroleum ether/ethyl acetate = 20/1) to afford the product as a white solid (10.1 mg, 40% yield, m.p. = 121–126 ^{o}C).

^{1}H NMR (400 MHz, $CDCl_3$) δ = 8.82 (d, *J* = 8.3 Hz, 1H), 8.50 (d, *J* = 8.3 Hz, 1H), 8.42 (d, *J* = 9.1 Hz, 1H), 7.82 – 7.76 (m, 1H), 7.66 – 7.59 (m, 1H), 7.57 (d, *J* = 2.6 Hz, 1H), 7.35 (dd, *J* = 9.1 Hz, *J* = 2.7 Hz, 1H), 4.00 (s, 3H), 2.94 (s, 3H).

^{13}C NMR (100 MHz, $CDCl_3$) δ = 202.7, 160.1, 154.3, 144.0, 133.7, 130.7, 127.7, 126.9, 123.2, 122.0, 121.4, 120.1, 119.4, 110.1, 55.6, 28.6.

HRMS (ESI) m/z calculated for $C_{16}H_{13}NO_2Na$ $[M+Na]^+$ 274.0839, found 274.0839.

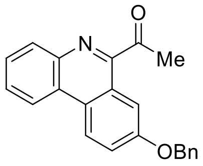

1-(8-(benzyloxy)Phenanthridin-6-yl)ethan-1-one. (**5**) The title compound was prepared following the general experimental procedure from 7-(benzyloxy)-10-methylphenanthren-9-ol (31.4 mg, 0.1 mmol). Purification was carried out by silica gel column chromatography (petroleum ether/ethyl acetate = 20/1) to afford the product as a yellow solid (13.4 mg, 41% yield, m.p. = 117–119 °C).

^{1}H NMR (400 MHz, $CDCl_3$) δ = 8.58 (d, *J* = 2.6 Hz, 1H), 8.55 (d, *J* = 9.1 Hz, 1H), 8.48 (dd, *J* = 6.4 Hz, *J* = 3.2 Hz, 1H), 8.22 – 8.19 (m, 1H), 7.73 – 7.71 (m, 2H), 7.57 – 7.52 (m, 3H), 7.44 (t, *J* = 7.4 Hz, 2H), 7.37 (t, *J* = 7.2 Hz, 1H), 5.25 (s, 2H), 2.96 (s, 3H).

^{13}C NMR (100 MHz, $CDCl_3$) δ = 203.2, 158.4, 152.0, 141.7, 136.4, 131.0, 128.9, 128.6, 128.2, 128.1, 127.9, 127.8, 125.6, 124.5, 123.6, 122.4, 121.6, 108.3, 70.2, 28.6.

HRMS (ESI) m/z calculated for $C_{22}H_{17}NO_2Na$ $[M+Na]^+$ 350.1152, found 350.1160.

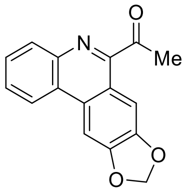

1-([1,3]dioxolo[4,5-*j*]phenanthridin-6-yl)Ethan-1-one. (**6**) The title compound was prepared following the general experimental procedure from 5-methylphenanthro[2,3-*d*][1,3]dioxol-6-ol (25.2 mg, 0.1 mmol). Purification was carried out by silica gel column chromatography (petroleum ether/ethyl acetate = 20/1) to afford the product as a yellow solid (12.7 mg, 48% yield, m.p. = 117–120 °C).

^{1}H NMR (400 MHz, $CDCl_3$) δ = 8.37 – 8.34 (m, 2H), 8.18 – 8.15 (m, 1H), 7.91 (s, 1H), 7.72 – 7.68 (m, 2H), 6.16 (s, 2H), 2.93 (s, 3H).

^{13}C NMR (100 MHz, $CDCl_3$) δ = 203.2, 151.9, 150.9, 148.8, 142.2, 131.7, 130.9, 128.4, 128.2, 125.5, 121.9, 119.8, 105.0, 101.9, 99.7, 28.5.

HRMS (ESI) m/z calculated for $C_{16}H_{11}NO_3Na$ $[M+Na]^+$ 288.0631, found 288.0616.

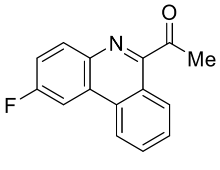

1-(2-fluorophenanthridin-6-yl)Ethan-1-one. (**7**) The title compound was prepared following the general experimental procedure from 3-fluoro-10-methylphenanthren-9-ol (22.6 mg, 0.1 mmol). Purification was carried out by silica gel column chromatography (petroleum ether/ethyl acetate = 30/1) to afford the product as a white solid (14.4 mg, 60% yield, m.p. = 114–116 °C).

^{1}H NMR (400 MHz, $CDCl_3$) δ = 8.93 (ddd, *J* = 8.4 Hz, *J* = 1.4 Hz, *J* = 0.7 Hz, 1H), 8.52 – 8.47 (m, 1H), 8.21 (dd, *J* = 9.0 Hz, *J* = 5.8 Hz, 1H), 8.16 (dd, *J* = 10.1 Hz, *J* = 2.7 Hz, 1H), 7.86 (ddd, *J* = 8.3 Hz, *J* = 7.0 Hz, *J* = 1.4 Hz, 1H), 7.75 (ddd, *J* = 8.3 Hz, *J* = 7.0 Hz, *J* = 1.2 Hz, 1H), 7.51 (ddd, *J* = 9.0 Hz, *J* = 7.9 Hz, *J* = 2.7 Hz, 1H), 2.93 (s, 3H).

^{13}C NMR (100 MHz, $CDCl_3$) δ = 202.5, 163.8 (d, *J* = 248.6 Hz), 139.2, 133.4 (d, *J* = 9.2 Hz), 132.9, 130.8, 128.8, 127.9, 126.9 (d, *J* = 9.6 Hz), 123.0, 122.1, 118.0 (d, *J* = 24.6 Hz), 107.1 (d, *J* = 23.5 Hz), 28.5.

^{19}**F NMR** (376 MHz, $CDCl_3$) δ = −109.29 (s, 1F).

HRMS (ESI) m/z calculated for $C_{15}H_{10}NOFNa$ $[M+Na]^+$ 262.0639, found 262.0639.

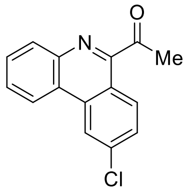

1-(9-chlorophenanthridin-6-yl)Ethan-1-one. (**8**) The title compound was prepared following the general experimental procedure from 6-chloro-10-methylphenanthren-9-ol (24.3 mg, 0.1 mmol). Purification was carried out by silica gel column chromatography (petroleum ether/ethyl acetate = 30/1) to afford the product as a yellow solid (13.3 mg, 52% yield, m.p. = 107–110 °C).

^{1}**H NMR** (400 MHz, $CDCl_3$) δ = 8.89 (d, *J* = 9.0 Hz, 1H), 8.51 (d, *J* = 1.6 Hz, 1H), 8.42 (d, *J* = 7.9 Hz, 1H), 8.19 (d, *J* = 8.0 Hz, 1H), 7.82 – 7.71 (m, 2H), 7.61 (dd, *J* = 8.9 Hz, *J* = 1.7 Hz, 1H), 2.93 (s, 3H).

^{13}**C NMR** (100 MHz, $CDCl_3$) δ = 202.4, 152.7, 142.7, 137.3, 134.8, 131.1, 129.6, 129.5, 129.1, 128.6, 124.2, 122.0, 121.6, 121.3, 28.4.

HRMS (ESI) m/z calculated for $C_{15}H_{10}NOClNa$ $[M+Na]^+$ 278.0343, found 278.0348.

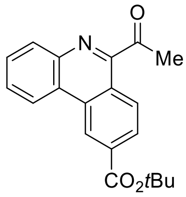

***tert*-Butyl 6-acetylphenanthridine-9-carboxylate**. (**9**) The title compound was prepared following the general experimental procedure from *tert*-butyl 10-hydroxy-9-methylphenanthrene-3-carboxylate (30.8 mg, 0.1 mmol). Purification was carried out by silica gel column chromatography (petroleum ether/ethyl acetate = 20/1) to afford the product as a yellow solid (23.1 mg, 72% yield, m.p. = 101–103 °C).

^{1}**H NMR** (400 MHz, $CDCl_3$) δ = 9.29 (d, *J* = 1.7 Hz, 1H), 8.98 (d, *J* = 8.7 Hz, 1H), 8.69 – 8.64 (m, 1H), 8.26 – 8.23 (m, 2H), 7.83 – 7.80 (m, 2H), 2.96 (s, 3H), 1.69 (s, 9H).

^{13}**C NMR** (100 MHz, $CDCl_3$) δ = 202.5, 165.1, 153.1, 142.6, 133.3, 133.1, 131.2, 129.4, 129.3, 127.9, 127.8, 125.4, 124.9, 123.8, 122.2, 82.1, 28.5, 28.2.

HRMS (ESI) m/z calculated for $C_{20}H_{20}NO_3$ $[M+H]^+$ 322.1438, found 322.1438.

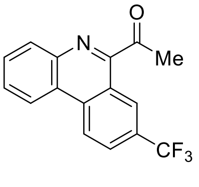

1-(8-(trifluoromethyl)phenanthridin-6-yl)Ethan-1-one. (**10**) The title compound was prepared following the general experimental procedure from 10-methyl-7-(trifluoromethyl)phenanthren-9-ol (27.6 mg, 0.1 mmol). Purification was carried out by silica gel column chromatography (petroleum ether/ethyl acetate = 30/1) to afford the product as a white solid (20.2 mg, 70% yield, m.p. = 111–113 °C).

^{1}**H NMR** (400 MHz, $CDCl_3$) δ = 9.33 (s, 1H), 8.74 (d, *J* = 8.7 Hz, 1H), 8.59 (d, *J* = 7.9 Hz, 1H), 8.27 (dd, *J* = 7.9 Hz, *J* = 1.6 Hz, 1H), 8.04 (dd, *J* = 8.6 Hz, *J* = 2.0 Hz, 1H), 7.90 – 7.80 (m, 2H), 2.97 (s, 3H).

^{13}**C NMR** (100 MHz, $CDCl_3$) δ = 202.3, 152.8 143.0, 135.5, 131.3, 130.1, 130.0 (q, *J* = 46.0 Hz), 129.6, 126.6, 126.5,

125.8, 125.7, 124.5, 123.0, 122.4, 28.3.

^{19}F NMR (376 MHz, $CDCl_3$) δ = −62.17 (s, 3F).

HRMS (ESI) m/z calculated for $C_{16}H_{10}NOF_3$ $[M+Na]^+$ 312.0607, found 312.0606.

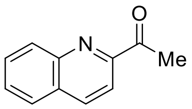

1-(quinolin-2-yl)Ethan-1-one. (**11**) The title compound was prepared following the general experimental procedure from 1-methylnaphthalen-2-ol (15.8 mg, 0.1 mmol). Purification was carried out by silica gel column chromatography (petroleum ether/ethyl acetate = 30/1) to afford the product as a white solid (10.3 mg, 60% yield, m.p. = 43–46 °C).

^{1}H NMR (400 MHz, $CDCl_3$) δ = 8.25 (d, *J* = 8.5 Hz, 1H), 8.20 (dd, *J* = 8.5 Hz, *J* = 1.1 Hz, 1H), 8.12 (d, *J* = 8.5 Hz, 1H), 7.86 (dd, *J* = 8.3Hz, *J* = 1.5 Hz, 1H), 7.80 – 7.76 (m, 1H), 7.66 – 7.62 (m, 1H), 2.87 (s, 3H).

^{13}C NMR (100 MHz, $CDCl_3$) δ = 200.7, 153.2, 147.2, 136.8, 130.5, 129.9, 129.5, 128.5, 127.6, 117.9, 25.5.

HRMS (ESI) m/z calculated for $C_{11}H_{10}NO$ $[M+H]^+$ 172.0757, found 172.0758.

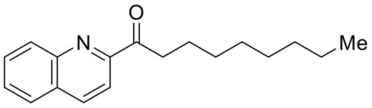

1-(quinolin-2-yl)Nonan-1-one. (**12**) The title compound was prepared following the general experimental procedure from 1-octylnaphthalen-2-ol (25.6 mg, 0.1 mmol). Purification was carried out by silica gel column chromatography (petroleum ether/ethyl acetate = 30/1) to afford the product as a colorless oil (14.0 mg, 52% yield).

^{1}H NMR (400 MHz, $CDCl_3$) δ = 8.25 (d, *J* = 8.5 Hz, 1H), 8.19 (d, *J* = 8.5 Hz, 1H), 8.12 (d, *J* = 8.6 Hz, 1H), 7.86 (d, *J* = 8.1 Hz, 1H), 7.79 – 7.76 (m, 1H), 7.64 (t, *J* = 7.5 Hz, 1H), 3.39 (t, *J* = 7.5 Hz, 2H), 1.83 – 1.75 (m, 2H), 1.47 – 1.40 (m, 2H), 1.37 – 1.25 (m, 8H), 0.88 (t, *J* = 6.8 Hz, 3H).

^{13}C NMR (100 MHz, $CDCl_3$) δ = 202.8, 153.1, 147.2, 136.8, 130.5, 129.9, 129.5, 128.4, 127.6, 118.2, 37.43 31.9, 29.5, 29.4, 29.2, 24.2, 22.7, 14.1.

HRMS (ESI) m/z calculated for $C_{18}H_{24}NO$ $[M+H]^+$ 270.1852, found 270.1853.

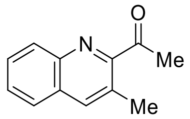

1-(3-methylquinolin-2-yl)Ethan-1-one. (**13**) The title compound was prepared following the general experimental procedure from 1,3-dimethylnaphthalen-2-ol (17.2 mg, 0.1 mmol). Purification was carried out by silica gel column chromatography (petroleum ether/ethyl acetate = 30/1) to afford the product as a yellow oil (13.0 mg, 70% yield).

^{1}H NMR (400 MHz, $CDCl_3$) δ = 8.10 (d, *J* = 8.2 Hz, 1H), 7.95 (s, 1H), 7.75 (dd, *J* = 8.1 Hz, *J* = 1.5 Hz, 1H), 7.71 – 7.67 (m, 1H), 7.59 – 7.56 (m, 1H), 2.84 (s, 3H), 2.69 (s, 3H).

^{13}C NMR (100 MHz, $CDCl_3$) δ = 202.7, 153.0, 145.5, 138.1, 130.2, 129.8, 129.1, 128.9, 128.3, 126.6, 28.1, 20.4.

HRMS (ESI) m/z calculated for $C_{12}H_{11}NONa$ $[M+Na]^+$ 208.0733, found 208.0737.

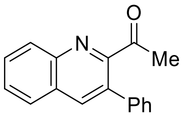

1-(3-phenylquinolin-2-yl)Ethan-1-one. (**14**) The title compound was prepared following the general experimental procedure from 1-methyl-3-phenylnaphthalen-2-ol (23.4 mg, 0.1 mmol). Purification was carried out by silica gel column chromatography (petroleum ether/ethyl acetate = 30/1) to afford the product as a yellow solid (12.9 mg, 52% yield, m.p. = 85–87 ºC).

^{1}H NMR (400 MHz, $CDCl_3$) δ = 8.19 (d, *J* = 8.5 Hz, 1H), 8.15 (s, 1H), 7.87 (d, *J* = 8.4 Hz, 1H), 7.80 – 7.76 (m, 1H), 7.66 – 7.63 (m, 1H), 7.46 – 7.41 (m, 3H), 7.39 – 7.37 (m, 2H), 2.69 (s, 3H).

^{13}C NMR (100 MHz, $CDCl_3$) δ = 202.2, 155.1, 146.0, 138.7, 137.9, 133.3, 130.0, 129.8, 128.7, 128.5, 128.4, 127.7, 127.6, 28.7.

HRMS (ESI) m/z calculated for $C_{17}H_{14}NO$ $[M+H]^+$ 248.1070, found 248.1070.

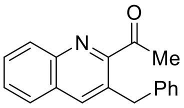

1-(3-benzylquinolin-2-yl)Ethan-1-one. (**15**) The title compound was prepared following the general experimental procedure from 3-benzyl-1-methylnaphthalen-2-ol (24.8 mg, 0.1 mmol). Purification was carried out by silica gel column chromatography (petroleum ether/ethyl acetate = 30/1) to afford the product as a white solid (16.2 mg, 62% yield, m.p. = 66–68 ºC).

^{1}H NMR (400 MHz, $CDCl_3$) δ = 8.12 (d, *J* = 8.4 Hz, 1H), 7.91 (s, 1H), 7.78 – 7.68 (m, 2H), 7.59 (t, *J* = 7.5 Hz, 1H), 7.29 – 7.26 (m, 2H), 7.23 (d, *J* = 8.1 Hz, 1H), 7.17 (d, *J* = 7.4 Hz, 2H), 4.48 (s, 2H), 2.69 (s, 3H).

^{13}C NMR (100 MHz, $CDCl_3$) δ = 202.9, 153.7, 145.5, 139.9, 137.9, 132.9, 129.7, 129.4, 129.3, 128.9, 128.5, 128.3, 127.09, 126.3, 38.3, 28.3.

HRMS (ESI) m/z calculated for $C_{18}H_{16}NO$ $[M+H]^+$ 262.1226, found 262.1219.

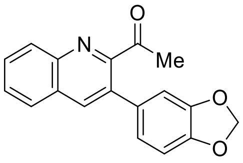

1-(3-(benzo[*d*][1,3]dioxol-5-yl)quinolin-2-yl)Ethan-1-one. (**16**) The title compound was prepared following the general experimental procedure from 3-(dibenzo[b,d]furan-3-yl)-1-methylnaphthalen-2-ol (27.8 mg, 0.1 mmol). Purification was carried out by silica gel column chromatography (petroleum ether/ethyl acetate = 20/1) to afford the product as a yellow oil (16.6 mg, 57% yield).

^{1}H NMR (400 MHz, $CDCl_3$) δ = 8.17 (d, *J* = 8.7 Hz, 1H), 8.11 (s, 1H), 7.85 (d, *J* = 8.2 Hz, 1H), 7.81 – 7.73 (m, 1H), 7.68 – 7.59 (m, 1H), 6.91 – 6.79 (m, 3H), 6.03 (s, 2H), 2.68 (s, 3H).

^{13}C NMR (100 MHz, $CDCl_3$) δ = 202.2, 155.3, 147.8, 147.4, 145.9, 137.6, 132.8, 132.4, 132.3, 129.9, 129.7, 128.3, 127.5, 122.4, 109.3, 108.4, 101.3, 28.8.

HRMS (ESI) m/z calculated for $C_{18}H_{14}NO_3$ $[M+H]^+$ 292.0968, found 292.0980.

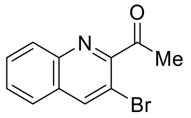

1-(3-bromoquinolin-2-yl)Ethan-1-one. (**17**) The title compound was prepared following the general experimental procedure from 3-bromo-1-methylnaphthalen-2-ol (23.7 mg, 0.1 mmol). Purification was carried out by silica gel column chromatography (petroleum ether/ethyl acetate = 30/1) to afford the product as a pale yellow solid (13.0 mg, 52% yield, m.p. = 57–60 °C).

^{1}H NMR (400 MHz, $CDCl_3$) δ = 8.40 (s, 1H), 8.07 (d, *J* = 8.4 Hz, 1H), 7.78 – 7.71 (m, 2H), 7.63 – 7.59 (m, 1H), 2.79 (s, 3H).

^{13}C NMR (100 MHz, $CDCl_3$) δ = 199.9, 152.7, 145.2, 140.8, 130.4, 129.7, 129.4, 129.0, 126.5, 112.3, 28.2.

HRMS (ESI) m/z calculated for $C_{11}H_9NOBr$ $[M+H]^+$ 249.9862, found 249.9857.

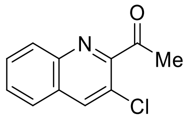

1-(3-chloroquinolin-2-yl)Ethan-1-one. (**18**) The title compound was prepared following the general experimental procedure from 3-chloro-1-methylnaphthalen-2-ol (19.2 mg, 0.1 mmol). Purification was carried out by silica gel column chromatography (petroleum ether/ethyl acetate = 30/1) to afford the product as a white solid (10.3 mg, 41% yield, m.p. = 41–42 °C).

^{1}H NMR (400 MHz, $CDCl_3$) δ = 8.24 – 8.22 (m, 1H), 8.12 (m, 1H), 7.79 – 7.74 (m, 2H), 7.67 – 7.62 (m, 1H), 2.81 (s, 3H).

^{13}C NMR (100 MHz, $CDCl_3$) δ = 199.5, 151.9, 145.1, 137.3, 136.3, 130.3, 129.9, 129.1, 126.6, 125.1, 28.4.

HRMS (ESI) m/z calculated for $C_{11}H_8NOClNa$ $[M+Na]^+$ 228.0187, found 228.0171.

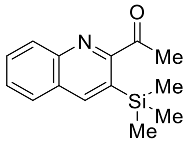

1-(3-(trimethylsilyl)quinolin-2-yl)Ethan-1-one. (**19**) The title compound was prepared following the general experimental procedure from 1-methyl-3-(trimethylsilyl)naphthalen-2-ol (23.0 mg, 0.1 mmol). Purification was carried out by silica gel column chromatography (petroleum ether/ethyl acetate = 50/1) to afford the product as a white solid (12.7 mg, 52% yield, m.p. = 51–52 °C).

^{1}H NMR (400 MHz, $CDCl_3$) δ = 8.46 (s, 1H), 8.16 (d, *J* = 8.4 Hz, 1H), 7.86 (d, *J* = 7.6 Hz, 1H), 7.77– 7.75 (m, 1H), 7.66 – 7.60 (m, 1H), 2.87 (s, 3H), 0.38 (s, 9H).

^{13}C NMR (100 MHz, $CDCl_3$) δ = 202.4, 156.8, 146.9, 145.1, 131.7, 130.2, 129.9, 128.5, 128.4, 127.7, 26.4, 0.2.

HRMS (ESI) m/z calculated for $C_{11}H_{17}NOSi$ $[M+H]^+$ 244.1152, found 244.1146.

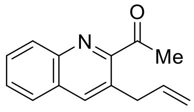

1-(3-allylquinolin-2-yl)Ethan-1-one. (**20**) The title compound was prepared following the general experimental procedure from 3-allyl-1-methylnaphthalen-2-ol (19.8 mg, 0.1 mmol). Purification was carried out by silica gel column chromatography (petroleum ether/ethyl acetate = 30/1) to afford the product as a pale yellow solid (16.1 mg, 76% yield, m.p. = 61–63 °C).

^{1}H NMR (400 MHz, $CDCl_3$) δ = 8.13 – 8.08 (m, 1H), 8.00 (s, 1H), 7.78 (d, *J* = 8.1 Hz, 1H), 7.70 – 7.69 (m, 1H), 7.63 – 7.58 (m, 1H), 6.11 – 6.00 (m, 1H), 5.12 (dd, *J* = 10.1 Hz, *J* = 1.6 Hz, 1H), 5.05 (dd, *J* = 17.2 Hz, *J* = 1.8 Hz, 1H), 3.86 (d, *J* = 6.6 Hz, 2H), 2.82 (s, 3H).

^{13}C NMR (100 MHz, $CDCl_3$) δ = 202.8, 153.4, 145.6, 137.5, 136.7, 131.9, 129.8, 129.3, 129.0, 128.3, 126.9, 116.6, 36.7, 28.3.

HRMS (ESI) m/z calculated for $C_{14}H_{13}NONa$ $[M+Na]^+$ 234.0889, found 234.0881.

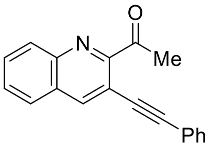

1-(3-(phenylethynyl)quinolin-2-yl)Ethan-1-one. (**21**) The title compound was prepared following the general experimental procedure from 1-methyl-3-(phenylethynyl)naphthalen-2-ol (25.8 mg, 0.1 mmol). Purification was carried out by silica gel column chromatography (petroleum ether/ethyl acetate = 30/1) to afford the product as a yellow solid (10.3 mg, 38% yield, m.p. = 56–68 °C).

^{1}H NMR (400 MHz, $CDCl_3$) δ = 8.45 (s, 1H), 8.15 (d, *J* = 8.5 Hz, 1H), 7.83 (d, *J* = 8.1 Hz, 1H), 7.78 (t, *J* = 7.7 Hz, 1H), 7.68 – 7.61 (m, 3H), 7.38 – 7.37 (m, 3H), 2.87 (s, 3H).

^{13}C NMR (100 MHz, $CDCl_3$) δ = 199.8, 153.9, 145.4, 141.5, 131.9, 130.6, 130.1, 128.9, 128.7, 128.4, 128.0, 127.1, 122.9, 114.8, 95.1, 86.4, 27.5.

HRMS (ESI) m/z calculated for $C_{19}H_{13}NONa$ $[M+Na]^+$ 294.0889, found 294.0889.

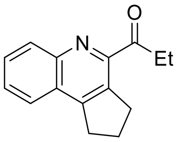

1-(2,3-dihydro-1*H*-cyclopenta[*c*]quinolin-4-yl)Propan-1-one. (**22**) The title compound was prepared following the general experimental procedure from 5-ethyl-2,3-dihydro-1*H*-cyclopenta[*a*]naphthalen-4-ol (21.2 mg, 0.1 mmol). Purification was carried out by silica gel column chromatography (petroleum ether/ethyl acetate = 30/1) to afford the product as a pale yellow solid (11.7 mg, 52% yield, m.p. = 95–97 °C).

^{1}H NMR (400 MHz, $CDCl_3$) δ = 8.18 (d, *J* = 8.4 Hz, 1H), 7.82 (d, *J* = 8.1 Hz, 1H), 7.70 (t, *J* = 7.6 Hz, 1H), 7.61 (t, *J* = 7.4 Hz, 1H), 3.49 – 3.39 (m, 4H), 3.26 (t, *J* = 7.7 Hz, 2H), 2.31 – 2.24 (m, 2H), 1.26 (t, *J* = 6.4 Hz, 3H).

^{13}C NMR (100 MHz, $CDCl_3$) δ = 204.8, 152.5, 149.8, 146.2, 136.2, 130.63 128.8, 128.1, 127.2, 124.1, 33.2, 31.8, 30.6, 24.4, 8.0.

HRMS (ESI) m/z calculated for $C_{15}H_{16}NO$ $[M+H]^+$ 226.1226, found 226.1213.

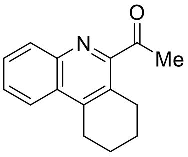

1-(7,8,9,10-tetrahydrophenanthridin-6-yl)Ethan-1-one. (**23**) The title compound was prepared following the general experimental procedure from 10-methyl-5,6,7,8-tetrahydrophenanthren-9-ol (21.2 mg, 0.1 mmol). Purification was carried out by silica gel column chromatography (petroleum ether/ethyl acetate = 30/1) to afford the product as a pale yellow solid (11.5 mg, 51% yield, m.p. = 85–86 °C).

^{1}H NMR (400 MHz, $CDCl_3$) δ = 8.06 (d, *J* = 8.3 Hz, 1H), 7.91 (d, *J* = 8.4 Hz, 1H), 7.65 (m, 1H), 7.57 (m, 1H), 3.11 (m,

4H), 2.80 (s, 3H), 1.93 (m, 2H), 1.82 (m, 2H).

^{13}C NMR (100 MHz, $CDCl_3$) δ = 203.4, 154.1, 144.5, 143.2, 130.5, 128.6, 128.5, 128.4, 127.9, 122.5, 28.7, 26.8, 25.9, 22.4, 21.8.

HRMS (ESI) m/z calculated for $C_{15}H_{16}NO$ $[M+H]^+$ 226.1226, found 226.1226.

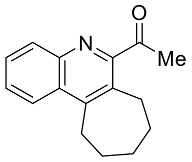

1-(8,9,10,11-tetrahydro-7*H*-cyclohepta[*c*]quinolin-6-yl)Ethan-1-one. (**24**) The title compound was prepared following the general experimental procedure from 5-methyl-8,9,10,11-tetrahydro-7*H*-cyclohepta[*a*]naphthalen-6-ol (22.6 mg, 0.1 mmol). Purification was carried out by silica gel column chromatography (petroleum ether/ethyl acetate = 30/1) to afford the product as a pale yellow solid (12.0 mg, 50% yield, m.p. = 72–73 ^{o}C).

^{1}H NMR (400 MHz, $CDCl_3$) δ = 8.08 (dt, *J* = 8.3 Hz, *J* = 1.4 Hz, 2H), 7.71 – 7.62 (m, 1H), 7.60 – 7.54 (m, 1H), 3.31 – 3.26 (m, 2H), 3.13 – 3.08 (m, 2H), 2.79 (s, 3H), 1.89 (m, 2H), 1.70 (m, 4H).

^{13}C NMR (100 MHz, $CDCl_3$) δ = 204.0, 155.2, 150.7, 145.4, 133.2, 130.5, 128.6, 127.5, 127.3, 123.0, 31.7, 29.2, 29.0, 27.7, 26.6, 25.9.

HRMS (ESI) m/z calculated for $C_{16}H_{18}NO$ $[M+H]^+$ 240.1383, found 240.1388.

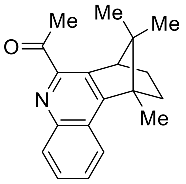

1-(10,11,11-trimethyl-7,8,9,10-tetrahydro-7,10-methanophenanthridin-6-yl)Ethan-1-one. (**25**) The title compound was prepared following the general experimental procedure from 4,9,11,11-tetramethyl-1,2,3,4-tetrahydro-1,4-methanophenanthren-10-ol (26.6 mg, 0.1 mmol). Purification was carried out by silica gel column chromatography (petroleum ether/ethyl acetate = 20/1) to afford the product as a yellow oil (17.6 mg, 63% yield).

^{1}H NMR (400 MHz, $CDCl_3$) δ = 8.28 – 8.25 (m, 1H), 8.18 (dd, *J* = 8.9 Hz, *J* = 1.0 Hz, 1H), 7.65 (m, 1H), 7.54 (m, 1H), 3.86 (d, *J* = 4.0 Hz, 1H), 2.87 (s, 3H), 2.27 – 2.20 (m, 1H), 1.99 – 1.93 (m, 1H), 1.74 (s, 3H), 1.23 – 1.17 (m, 1H), 1.13 – 1.07 (m, 1H), 1.01 (s, 3H), 0.55 (s, 3H).

^{13}C NMR (100 MHz, $CDCl_3$) δ = 202.6, 156.3, 147.4, 146.8, 139.6, 131.6, 128.1, 127.4, 126.2, 123.3, 58.7, 55.9, 51.3, 32.6, 27.0, 25.5, 19.9, 19.1, 14.7.

HRMS (ESI) m/z calculated for $C_{19}H_{22}NO$ $[M+H]^+$ 280.1696, found 280.1699.

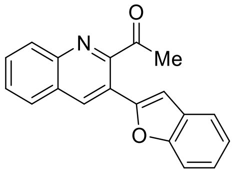

1-(3-(benzofuran-2-yl)quinolin-2-yl)Ethan-1-one. (**26**) The title compound was prepared following the general experimental procedure from 3-(benzofuran-2-yl)-1-methylnaphthalen-2-ol (27.4 mg, 0.1 mmol). Purification was carried out by silica gel column chromatography (petroleum ether/ethyl acetate = 20/1) to afford the product as a yellow solid (14.1 mg, 49% yield, m.p. = 94–96 ^{o}C).

¹H NMR (400 MHz, $CDCl_3$) δ = 8.56 (s, 1H), 8.14 (d, *J* = 8.5 Hz, 1H), 7.88 (d, *J* = 8.1 Hz, 1H), 7.77 (m, 1H), 7.62 (m, 2H), 7.51 (d, *J* = 8.1 Hz, 1H), 7.32 (tt, *J* = 8.4 Hz, *J* = 1.5 Hz, 1H), 7.28 – 7.23 (m, 1H), 7.05 (s, 1H), 2.83 (s, 3H).

¹³C NMR (100 MHz, $CDCl_3$) δ = 202.1, 155.1, 154.6, 152.2, 146.2, 136.2, 130.7, 129.6, 128.8, 128.4, 127.9, 127.9, 124.9, 123.1, 121.4, 121.2, 111.2, 105.8, 28.6.

HRMS (ESI) m/z calculated for $C_{19}H_{14}N_2O_2$ $[M+H]^+$ 288.1019, found 288.1035.

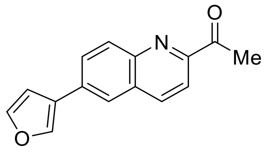

1-(6-(furan-3-yl)quinolin-2-yl)Ethan-1-one. (**27**) The title compound was prepared following the general experimental procedure from 6-(furan-3-yl)-1-methylnaphthalen-2-ol (22.4 mg, 0.1 mmol). Purification was carried out by silica gel column chromatography (petroleum ether/ethyl acetate = 20/1) to afford the product as a yellow solid (12.3 mg, 52% yield, m.p. = 85–87 °C).

¹H NMR (400 MHz, $CDCl_3$) δ = 8.23 (d, *J* = 8.5 Hz, 1H), 8.18 (d, *J* = 9.3 Hz, 1H), 8.12 (d, *J* = 8.5 Hz, 1H), 7.94 – 7.89 (m, 3H), 7.56 (s, 1H), 6.85 (s, 1H), 2.87 (s, 3H).

¹³C NMR (100 MHz, $CDCl_3$) δ = 200.5, 152.8, 146.4, 144.3, 139.7, 136.5, 132.7, 131.0, 129.9, 128.6, 125.7, 123.2, 118.5, 108.7, 25.6.

HRMS (ESI) m/z calculated for $C_{15}H_{11}NO_2Na$ $[M+Na]^+$ 260.0682, found 260.0688.

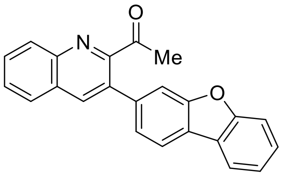

1-(3-(dibenzo[b,d]furan-3-yl)quinolin-2-yl)Ethan-1-one. (**28**) The title compound was prepared following the general experimental procedure from 3-(dibenzo[b,d]furan-3-yl)-1-methylnaphthalen-2-ol (32.4 mg, 0.1 mmol). Purification was carried out by silica gel column chromatography (petroleum ether/ethyl acetate = 20/1) to afford the product as a yellow solid (15.5 mg, 46% yield, m.p. = 113–114 °C).

¹H NMR (400 MHz, $CDCl_3$) δ = 8.24 (s, 2H), 7.99 (t, *J* = 7.0 Hz, 2H), 7.85 (dd, *J* = 31.8 Hz, *J* = 6.9 Hz, 2H), 7.72 – 7.46 (m, 4H), 7.42 – 7.32 (m, 2H), 2.74 (s, 3H).

¹³C NMR (100 MHz, $CDCl_3$) δ = 201.9, 156.6, 156.2, 154.9, 146.0, 142.7, 138.2, 137.9, 133.2, 130.2, 129.8, 128.5, 128.3, 127.6, 127.4, 123.8, 123.8, 122.9, 120.7, 120.6, 111.9, 111.7, 28.8.

HRMS (ESI) m/z calculated for $C_{23}H_{15}NO_2Na$ $[M+Na]^+$ 360.0995, found 360.0990.

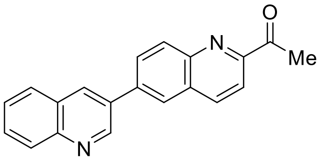

1-([3,6'-biquinolin]-2'-yl)Ethan-1-one. (**29**) The title compound was prepared following the general experimental procedure from 1-methyl-6-(quinolin-3-yl)naphthalen-2-ol (28.5 mg, 0.1 mmol). Purification was carried out by silica gel column chromatography (petroleum ether/ethyl acetate = 10/1) to afford the product as a yellow solid (9.5 mg, 32% yield, m.p. = 102–103 °C).

¹H NMR (400 MHz, $CDCl_3$) δ = 9.30 (d, *J* = 2.3 Hz, 1H), 8.46 (d, *J* = 2.0 Hz, 1H), 8.33 (dd, *J* = 8.4 Hz, *J* = 2.1 Hz, 2H),

8.22 – 8.09 (m, 4H), 7.92 (d, *J* = 8.0 Hz, 1H), 7.77 (ddd, *J* = 8.4 Hz, *J* = 7.0 Hz, *J* = 1.3 Hz, 1H), 7.62 (ddd, *J* = 7.9 Hz, *J* = 6.8 Hz, *J* = 0.9 Hz, 1H), 2.88 (s, 3H).

^{13}C NMR (100 MHz, $CDCl_3$) δ = 200.3, 153.5, 149.3, 147.2, 146.8, 137.7, 137.0, 134.2, 132.6, 131.6, 130.1, 129.8, 129.3, 129.0, 128.1, 127.9, 127. 5, 125.8, 118.7, 25.5.

HRMS (ESI) m/z calculated for $C_{20}H_{14}N_2ONa$ $[M+Na]^+$ 321.0998, found 321.1005.

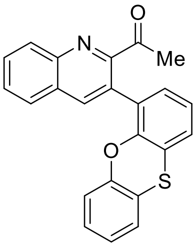

1-(3-(phenoxathiin-4-yl)quinolin-2-yl)Ethan-1-one. (**30**) The title compound was prepared following the general experimental procedure from 1-methyl-3-(phenoxathiin-4-yl)naphthalen-2-ol (35.6 mg, 0.1 mmol). Purification was carried out by silica gel column chromatography (petroleum ether/ethyl acetate = 15/1) to afford the product as a yellow oil (19.6 mg, 53% yield).

^{1}H NMR (400 MHz, $CDCl_3$) δ = 8.25 (dq, *J* = 8.5 Hz, *J* = 0.9 Hz, 1H), 8.09 (d, *J* = 1.0 Hz, 1H), 7.87 (dd, *J* = 8.1 Hz, *J* = 1.4 Hz, 1H), 7.82 (m, 1H), 7.66 (m, 1H), 7.20 (m, 1H), 7.15 (d, *J* = 1.1 Hz, 1H), 7.14 (d, *J* = 4.5 Hz, 1H), 7.09 – 7.06 (m, 1H), 6.99 – 6.95 (m, 2H), 6.56 – 6.53 (m, 1H), 2.83 (s, 3H).

^{13}C NMR (100 MHz, $CDCl_3$) δ = 200.6, 154.4, 151.6, 148.1, 146.1, 138.5, 130.1, 129.9, 129.7, 129.3, 128.7, 128.5, 128.4, 127. 6, 127.4, 126.7, 126.5, 124.6, 124.5, 120.3, 120.1, 117.3, 27.2.

HRMS (ESI) m/z calculated for $C_{23}H_{16}NO_2S$ $[M+H]^+$ 370.0896, found 370.0892.

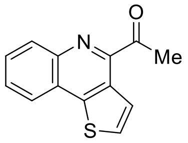

1-(thieno[3,2-*c*]quinolin-4-yl)Ethan-1-one. (**31**) The title compound was prepared following the general experimental procedure from 5-methylnaphtho[1,2-*b*]thiophen-4-ol (21.4 mg, 0.1 mmol). Purification was carried out by silica gel column chromatography (petroleum ether/ethyl acetate = 20/1) to afford the product as a white solid (14.3 mg, 63% yield, m.p. = 97–98 ^{o}C).

^{1}H NMR (400 MHz, $CDCl_3$) δ = 8.33 (dt, *J* = 3.0 Hz, *J* = 1.1 Hz, 1H), 8.31 (dd, *J* = 2.8 Hz, *J* = 1.6 Hz, 1H), 7.98 (s, 2H), 7.80 – 7.71 (m, 2H), 2.98 (s, 3H).

^{13}C NMR (100 MHz, $CDCl_3$) δ = 200.9, 147.0, 143.9, 143.6, 143.3, 135.7, 131.0, 128.9, 128.4, 125.5, 123.4, 120.2, 25.2.

HRMS (ESI) m/z calculated for $C_{13}H_9NOSNa$ $[M+Na]^+$ 250.0297, found 250.0308.

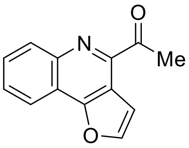

1-(furo[3,2-*c*]quinolin-4-yl)Ethan-1-one. (**32**) The title compound was prepared following the general experimental procedure from 5-methylnaphtho[1,2-*b*]furan-4-ol (19.8 mg, 0.1 mmol). Purification was carried out by silica gel column chromatography (petroleum ether/ethyl acetate = 20/1) to afford the product as a yellow solid (6.6 mg,

31% yield, m.p. = 109–111 °C).

^{1}H NMR (400 MHz, $CDCl_3$) δ = 8.33 – 8.28 (m, 1H), 8.17 – 8.12 (m, 1H), 8.03 – 8.00 (m, 1H), 7.74 (m, 2H), 7.33 – 7.30 (m, 1H), 2.95 (s, 3H).

^{13}C NMR (100 MHz, $CDCl_3$) δ = 199.7, 148.5, 146.7, 142.9, 139.9, 132.4, 131.1, 128.8, 128.1, 125.1, 123.4, 104.9, 26.8.

HRMS (ESI) m/z calculated for $C_{13}H_{10}NO_2$ $[M+H]^+$ 212.0706, found 212.0715.

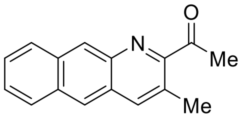

1-(3-methylbenzo[*g*]quinolin-2-yl)Ethan-1-one. (**33**) The title compound was prepared following the general experimental procedure from 5-methylbenzo[*c*]phenanthren-6-ol (22.2 mg, 0.1 mmol). Purification was carried out by silica gel column chromatography (petroleum ether/ethyl acetate = 15/1) to afford the product as a orange solid (14.6 mg, 62% yield, m.p. = 116–118 °C).

^{1}H NMR (400 MHz, $CDCl_3$) δ = 8.63 (s, 1H), 8.18 (s, 1H), 7.98 (d, *J* = 9.6 Hz, 2H), 7.95 – 7.89 (m, 1H), 7.47 – 7.40 (m, 2H), 2.80 (s, 3H), 2.62 (s, 3H).

^{13}C NMR (100 MHz, $CDCl_3$) δ = 202.6, 154.5, 142.3, 137.6, 133.6, 132.9, 129.1, 128.7, 128.4, 128.0, 126.9, 126.7, 126.1, 125.1, 28.1, 20.6.

HRMS (ESI) m/z calculated for $C_{16}H_{14}NO$ $[M+H]^+$ 236.1070, found 236.1068.

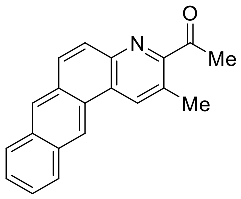

1-(2-methylnaphtho[2,3-*f*]quinolin-3-yl)Ethan-1-one. (**34**) The title compound was prepared following the general experimental procedure from 2,4-dimethyltetraphen-3-ol (27.2 mg, 0.1 mmol). Purification was carried out by silica gel column chromatography (petroleum ether/ethyl acetate = 15/1) to afford the product as a yellow solid (5.1 mg, 18% yield, m.p. = 120–121 °C).

^{1}H NMR (400 MHz, $CDCl_3$) δ = 9.08 (s, 1H), 8.85 (s, 1H), 8.38 (s, 1H), 8.12 (dt, *J* = 7.1 Hz, *J* = 3.5 Hz, 1H), 8.07 (dd, *J* = 6.2 Hz, *J* = 3.4 Hz, 1H), 7.99 (d, *J* = 9.3 Hz, 1H), 7.86 (d, *J* = 9.3 Hz, 1H), 7.61 (dt, *J* = 6.4 Hz, *J* = 3.3 Hz, 2H), 2.89 (s, 3H), 2.84 (s, 3H).

^{13}C NMR (100 MHz, $CDCl_3$) δ = 202.7, 151.4, 145.9, 133.8, 132.6, 131.9, 131.6, 131.1, 130.4, 128.4, 128.0, 127.9, 127.5, 127.3, 127.0, 126.7, 126.4, 122.7, 28.2, 20.9.

HRMS (ESI) m/z calculated for $C_{20}H_{16}NO$ $[M+H]^+$ 286.1226, found 286.1233.

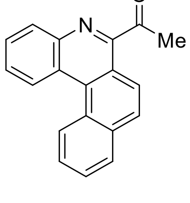

1-(benzo[*k*]phenanthridin-6-yl)Ethan-1-one. (**35**) The title compound was prepared following the general experimental procedure from 5-methylbenzo[*c*]phenanthren-6-ol (25.8 mg, 0.1 mmol). Purification was carried out by silica gel column chromatography (petroleum ether/ethyl acetate = 20/1) to afford the product as a pale yellow solid

(20.6 mg, 76% yield, m.p. = 105–106 ºC).

^{1}H NMR (400 MHz, $CDCl_3$) δ = 9.07 – 9.03 (m, 1H), 9.01 – 8.97 (m, 1H), 8.70 (d, *J* = 9.0 Hz, 1H), 8.35 – 8.29 (m, 1H), 8.05 – 8.01 (m, 1H), 7.97 (d, *J* = 9.0 Hz, 1H), 7.84 – 7.75 (m, 2H), 7.74 – 7.71 (m, 2H), 2.99 (s, 3H).

^{13}C NMR (100 MHz, $CDCl_3$) δ = 203.1, 153.3, 144.4, 134.6, 132.8, 130.7, 128.9, 128.7, 128.5, 128.5, 128.4, 128.3, 128.1, 127.2, 126.8, 125.4, 123.2, 122.0, 28.9.

HRMS (ESI) m/z calculated for $C_{19}H_{13}NONa$ $[M+Na]^+$ 294.0889, found 294.0889.

3.4 Characterization of synthetic application products

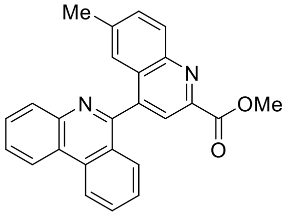

Methyl 6-methyl-4-(phenanthridin-6-yl)quinoline-2-carboxylate (36). The title compound was prepared following the general procedure G from 1-(phenanthridin-6-yl)ethan-1-one (0.1 mmol). Purification was carried out by silica gel column chromatography (petroleum ether/ethyl acetate = 10/1) to afford the product as a white solid (18.9 mg, 50% yield, m.p. = 230–232 ºC).

^{1}H NMR (400 MHz, $CDCl_3$) δ = 8.78 (d, *J* = 8.4 Hz, 1H), 8.74 – 8.70 (m, 1H), 8.36 – 8.31 (m, 2H), 8.26 (m, 1H), 7.90 (ddd, *J* = 8.4 Hz, *J* = 5.9 Hz, *J* = 2.4 Hz, 1H), 7.81 (m, 2H), 7.64 (dd, *J* = 8.7 Hz, *J* = 2.0 Hz, 1H), 7.57 – 7.51 (m, 2H), 7.28 (d, *J* = 1.3 Hz, 1H), 4.09 (s, 3H), 2.33 (s, 3H).

^{13}C NMR (100 MHz, $CDCl_3$) δ = 165.8, 157.9, 146.7, 146.5, 146.0, 143. 6, 139.7, 133.1, 132.9, 131.3, 130.8, 130.4, 129.2, 128.4, 128.2, 127.8, 127.7, 125.5, 124.4, 124.1, 122.4, 122.2, 121.9, 53.2, 21.9

HRMS (ESI) m/z calculated for $C_{25}H_{19}N_2O_2$ $[M+H]^+$ 379.1441, found 379.1437.

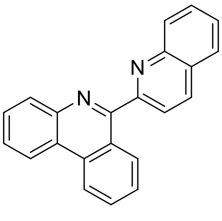

6-(quinolin-2-yl)Phenanthridine (37). The title compound was prepared following the general procedure H from 1-(phenanthridin-6-yl)ethan-1-one (0.1 mmol). as a pale yellow solid (25.4 mg, 83% yield, m.p. = 138–140 ºC).

^{1}H NMR (400 MHz, $CDCl_3$) δ = 8.71 (t, *J* = 9.3 Hz, 2H), 8.65 (d, *J* = 8.0 Hz, 1H), 8.40 (d, *J* = 8.5 Hz, 1H), 8.29 (dd, *J* = 11.8 Hz, *J* = 8.5 Hz, 2H), 8.18 (d, *J* = 8.4 Hz, 1H), 7.94 (d, *J* = 8.1 Hz, 1H), 7.88 (t, *J* = 7.5 Hz, 1H), 7.79 (q, *J* = 6.9 Hz, 2H), 7.75 – 7.70 (m, 1H), 7.65 (dt, *J* = 14.3 Hz, *J* = 7.6 Hz, 2H).

^{13}C NMR (100 MHz, $CDCl_3$) δ = 158.3, 158.1, 147.3, 143.6, 136.9, 133.7, 130.7, 130.5, 129.8, 129.8, 129.1, 128.8, 127.7, 127.6, 127.4, 127.3, 127.1, 124.9, 124.3, 122.7, 122.1, 122.0.

HRMS (ESI) m/z calculated for $C_{22}H_{15}N_2$ $[M+H]^+$ 307.1230, found 307.1215.

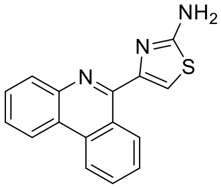

4-(phenanthridin-6-yl)Thiazol-2-amine (38). The title compound was prepared following the general procedure I from 1-(phenanthridin-6-yl)ethan-1-one (0.1 mmol). Purification was carried out by silica gel column chromatography (petroleum ether/ethyl acetate = 1/1) to afford the product as a brown solid (15.3 mg, 55% yield, m.p. = 110–112 ^{o}C).

^{1}H NMR (400 MHz, DMSO-d_6) δ = 9.08 (d, *J* = 8.3 Hz, 1H), 8.90 (d, *J* = 8.4 Hz, 1H), 8.83 (d, *J* = 8.1 Hz, 1H), 8.15 – 8.09 (m, 1H), 7.97 (t, *J* = 7.6 Hz, 1H), 7.84 – 7.74 (m, 3H), 7.32 (s, 1H), 7.30 (s, 2H).

^{13}C NMR (100 MHz, DMSO-d_6) δ = 168.3, 153.8, 150.4, 143.3, 132.9, 131.0, 129.7, 129.2, 129.1, 127.4, 127.2, 124.5, 123.5, 122.7, 122.5, 109.6.

HRMS (ESI) m/z calculated for $C_{16}H_{10}N_3S$ [M−H]$^-$ 276.0590, found 276.0577.

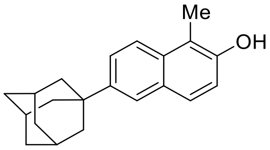

6-((3*r*,5*r*,7*r*)-adamantan-1-yl)-1-methylnaphthalen-2-ol (40). The title compound was prepared following the general procedure K from 1-methylnaphthalen-2-ol (**11a**) (1.0 mmol). Purification was carried out by silica gel column chromatography (petroleum ether/ethyl acetate = 15/1) to afford the product as a white solid (263.2 mg, 90% yield, m.p. = 209–211 ^{o}C).

^{1}H NMR (400 MHz, CDCl$_3$) δ = 7.90 (d, *J* = 9.0 Hz, 1H), 7.68 (d, *J* = 2.1 Hz, 1H), 7.62 (dd, *J* = 8.9 Hz, *J* = 2.1 Hz, 2H), 7.05 (d, *J* = 8.7 Hz, 1H), 4.96 (s, 1H), 2.55 (s, 3H), 2.20 – 2.14 (m, 3H), 2.04 (d, *J* = 3.0 Hz, 6H), 1.88 – 1.80 (m, 6H).

^{13}C NMR (100 MHz, CDCl$_3$) δ = 149.9, 145.9, 131.9, 129.2, 127.3, 124.5, 123.4, 122.9, 117.4, 114.9, 43.1, 36.8, 35.9, 28.9, 26.9, 10.4.

HRMS (ESI) m/z calculated for $C_{21}H_{23}O$ [M−H]$^-$ 291.1743, found 291.1743.

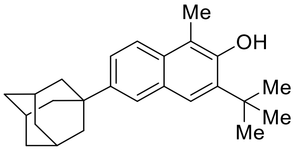

6-((3*r*,5*r*,7*r*)-adamantan-1-yl)-3-(*tert*-butyl)-1-methylnaphthalen-2-ol (41). The title compound was prepared following the general procedure L from 6-((3*r*,5*r*,7*r*)-adamantan-1-yl)-1-methylnaphthalen-2-ol (0.1 mmol). Purification was carried out by silica gel column chromatography (petroleum ether/ethyl acetate = 15/1) to afford the product as a pale yellow solid (19.5 mg, 56% yield, m.p. = 215–217 ^{o}C).

^{1}H NMR (400 MHz, CDCl$_3$) δ = 7.82 (d, *J* = 8.9 Hz, 1H), 7.68 (d, *J* = 2.1 Hz, 1H), 7.64 (s, 1H), 7.56 (dd, *J* = 8.9 Hz, *J* = 2.1 Hz, 1H), 5.06 (s, 1H), 2.52 (s, 3H), 2.18 – 2.12 (m, 3H), 2.03 (d, *J* = 3.0 Hz, 6H), 1.83 (q, *J* = 3.3 Hz, 6H), 1.54 (s, 9H).

^{13}C NMR (100 MHz, CDCl$_3$) δ = 150.4, 145.9, 137.7, 130.2, 128.8, 123.9, 123.8, 123.5, 122.2, 114.2, 43.2, 36.9, 35.9, 35.1, 29.9, 28.9, 10.5

HRMS (ESI) m/z calculated for $C_{25}H_{22}O$ [M−H]$^-$ 347.2369, found 347.2354.

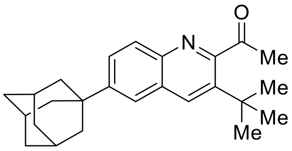

1-(6-((3*r*,5*r*,7*r*)-adamantan-1-yl)-3-(tert-butyl)quinolin-2-yl)ethan-1-one (42). The title compound was prepared following the general experimental procedure from 6-((3*r*,5*r*,7*r*)-adamantan-1-yl)-3-(tert-butyl)-1-methylnaphthalen-2-ol (0.1 mmol). Purification was carried out by silica gel column chromatography (petroleum ether/ethyl acetate = 20/1) to afford the product as a semisolid (18.1 mg, 50% yield).

^{1}H NMR (400 MHz, $CDCl_3$) δ = 8.16 (s, 1H), 7.96 (d, *J* = 8.9 Hz, 1H), 7.78 (dd, *J* = 8.9 Hz, *J* = 2.2 Hz, 1H), 7.67 (d, *J* = 2.1 Hz, 1H), 2.76 (s, 3H), 2.14 (dd, *J* = 6.0 Hz, *J* = 3.2 Hz, 3H), 2.01 (d, *J* = 2.9 Hz, 6H), 1.85 – 1.76 (m, 6H), 1.47 (s, 9H).

^{13}C NMR (100 MHz, $CDCl_3$) δ = 204.9, 157.7, 150.5, 143.3, 140.1, 134.1, 128.1, 127.8, 127.7, 122.5, 42.9, 36.7, 36.5, 34.5, 31.4, 30.3, 28.8.

HRMS (ESI) m/z calculated for $C_{25}H_{32}NO$ $[M+H]^+$ 362.2478, found 362.2465.

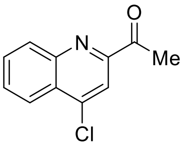

1-(4-chloroquinolin-2-yl)Ethan-1-one. (**43a**) The title compound was prepared following the general procedure M from 1-(quinolin-2-yl)ethan-1-one (0.5 mmol). Purification was carried out by silica gel column chromatography (petroleum ether/ethyl acetate = 30/1) to afford the product as a white solid (51.4 mg, 50% yield, m.p. = 90–92 °C).

^{1}H NMR (400 MHz, $CDCl_3$) δ = 8.27 (dd, *J* = 8.5 Hz, *J* = 1.4 Hz, 1H), 8.21 (d, *J* = 8.7 Hz, 1H), 8.19 (s, 1H), 7.83 (m, 1H), 7.74 (m, 1H), 2.85 (s, 3H).

^{13}C NMR (100 MHz, $CDCl_3$) δ = 199.5, 152.9, 148.0, 143.7, 130.9, 130.8, 129.6, 127.6, 124.2, 118.2, 25.4.

HRMS (ESI) m/z calculated for $C_{11}H_8NOClNa$ $[M+Na]^+$ 228.0187, found 228.0188.

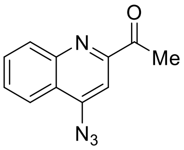

1-(4-azidoquinolin-2-yl)Ethan-1-one. (**43b**) The title compound was prepared following the general procedure M from 1-(4-chloroquinolin-2-yl)ethan-1-one (0.5 mmol). Purification was carried out by silica gel column chromatography (petroleum ether/ethyl acetate = 20/1) to afford the product as a pale yellow solid (87.0 mg, 82% yield, m.p. = 103–105 °C).

^{1}H NMR (400 MHz, $CDCl_3$) δ = 8.14 (dd, *J* = 8.5 Hz, *J* = 1.3 Hz, 1H), 8.06 (dd, *J* = 8.4 Hz, *J* = 1.5 Hz, 1H), 7.85 (d, *J* = 0.8 Hz, 1H), 7.82 – 7.76 (m, 1H), 7.62 (ddd, *J* = 8.1 Hz, *J* = 7.1 Hz, *J* = 1.0 Hz, 1H), 2.85 (s, 3H).

^{13}C NMR (100 MHz, $CDCl_3$) δ = 200.0, 153.3, 148.1, 147.2, 130.9, 130.3, 128.4, 122.3, 105.4, 25.4.

HRMS (ESI) m/z calculated for $C_{11}H_8N_4ONa$ $[M+Na]^+$ 235.0590, found 235.0577.

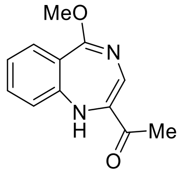

1-(5-methoxy-1*H*-benzo[*e*][1,4]diazepin-2-yl)Ethan-1-one. (**43**) The title compound was prepared following the general procedure M from 1-(4-azidoquinolin-2-yl)ethan-1-one (0.5 mmol). Purification was carried out by silica gel column chromatography (petroleum ether/ethyl acetate = 10/1) to afford the product as a orange solid (41.1 mg, 38% yield, m.p. = 96–100 ºC).

^{1}H NMR (400 MHz, $CDCl_3$) δ = 7.33 (dd, *J* = 7.8 Hz, *J* = 1.6 Hz, 1H), 7.25 – 7.21 (m, 1H), 7.03 (s, 1H), 6.87 (td, *J* = 7.6 Hz, *J* = 1.1 Hz, 1H), 6.56 (dd, *J* = 8.0 Hz, *J* = 1.1 Hz, 1H), 5.79 (s, 1H), 3.92 (s, 3H), 2.31 (s, 3H).

^{13}C NMR (100 MHz, $CDCl_3$) δ = 191.7, 166.9, 154.5, 135.5, 133.6, 132.7, 129.8, 124.3, 122.1, 119.6, 54.6, 24.1

HRMS (ESI) m/z calculated for $C_{12}H_{13}N_2O_2$ $[M+H]^+$ 217.0972, found 217.0961.

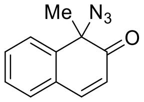

1-Azido-1-methylnaphthalen-2(1*H*)-one. (**44**) The title compound was prepared following the control experimental procedure from 1-methylnaphthalen-2-ol (0.1 mmol). Purification was carried out by silica gel column chromatography (petroleum ether/ethyl acetate = 50/1) to afford the product as a yellow oil (14.5 mg, 73% yield).

^{1}H NMR (400 MHz, $CDCl_3$) δ = 7.59 (dd, *J* = 7.4 Hz, *J* = 0.9 Hz, 1H), 7.46 – 7.41 (m, 2H), 7.36 – 7.29 (m, 2H), 6.16 (d, *J* = 9.9 Hz, 1H), 1.67 (s, 3H).

^{13}C NMR (100 MHz, $CDCl_3$) δ = 199.6, 145.4, 141.8, 130.6, 129.6, 128.6, 128.4, 126.9, 123.2, 67.3, 28.1.

HRMS (ESI) m/z calculated for $C_{11}H_9N_3ONa$ $[M+Na]^+$ 222.0638, found 222.0623.

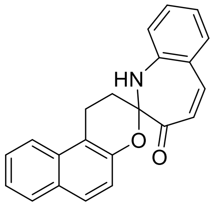

1',2'-dihydrospiro[benzo[*b*]azepine-2,3'-benzo[*f*]chromen]-3(1*H*)-one. **(45)** The title compound was prepared following the control experimental procedure from 1-azido-1-methylnaphthalen-2(1*H*)-one (0.1 mmol). Purification was carried out by silica gel column chromatography (petroleum ether/ethyl acetate = 5/1) to afford the product as a pale yellow solid (6.9 mg, 42% yield).

^{1}H NMR (400 MHz, $CDCl_3$) δ = 7.87 (dd, *J* = 8.5 Hz, *J* = 1.1 Hz, 1H), 7.72 (dd, *J* = 8.1 Hz, *J* = 1.3 Hz, 1H), 7.54 (ddd, *J* = 8.4 Hz, *J* = 6.9 Hz, *J* = 1.4 Hz, 1H), 7.46 (d, *J* = 8.9 Hz, 1H), 7.43 (dd, *J* = 7.8 Hz, *J* = 1.5 Hz, 1H), 7.41 – 7.35 (m, 2H), 7.11 – 7.05 (m, 1H), 6.95 (td, *J* = 7.5 Hz, *J* = 1.2 Hz, 1H), 6.64 (d, *J* = 8.9 Hz, 1H), 6.57 (d, *J* = 7.9 Hz, 1H), 6.47 (d, *J* = 12.4 Hz, 1H), 5.31 (s, 1H), 3.45 (ddd, *J* = 17.6 Hz, *J* = 7.2 Hz, *J* = 3.5 Hz, 1H), 3.19 (m, 1H), 2.76 (ddd, *J* = 14.3 Hz, *J* = 10.6 Hz, *J* = 7.2 Hz, 1H), 2.41 (ddd, *J* = 14.3 Hz, *J* = 7.1 Hz, *J* = 3.5 Hz, 1H)

^{13}C NMR (100 MHz, $CDCl_3$) δ = 187.9, 148.7, 142.8, 141.1, 133.8, 132.3, 131.4, 129.4, 128.4, 128.1, 126.4, 124.5, 123.9, 123.7, 121.9, 120.8, 119.2, 119.0, 112.8, 83.4, 25.6, 18.2.

HRMS (ESI) m/z calculated for $C_{28}H_{18}NO_2Na$ $[M+H]^+$ 328.1332, found 328.1338.

3.5 Characterization of unsuccessful example of naphthol

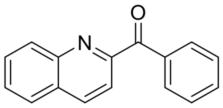

Phenyl(quinolin-2-yl)methanone. The title compound was prepared following the general experimental procedure from 1-phenylnaphthalen-2-ol. Purification was carried out by silica gel column chromatography (petroleum ether/ethyl acetate = 30/1) to afford the product as a pale yellow solid (1.6 mg, 7% yield).

^{1}H NMR (400 MHz, $CDCl_3$) δ = 8.36 (dd, *J* = 8.5, 0.9 Hz, 1H), 8.25 – 8.20 (m, 3H), 8.11 (d, *J* = 8.5 Hz, 1H), 7.92 (dd, *J* = 8.2 Hz, *J* =1.5 Hz, 1H), 7.81 – 7.77 (m, 1H), 7.70 – 7.60 (m, 2H), 7.56 – 7.49 (m, 2H).

^{13}C NMR (100 MHz, $CDCl_3$) δ = 193.8, 154.7, 146.7, 137.1, 136.1, 133.1, 131.5, 130.5, 130.1, 128.9, 128.4, 128.1, 127.6, 120.8.

Spectra data in accordance with the reported literature.[15]

3.6 NMR Spectra

Copies of ^{1}H and ^{13}C spectra

10-Methylphenanthren-9-ol (1a)

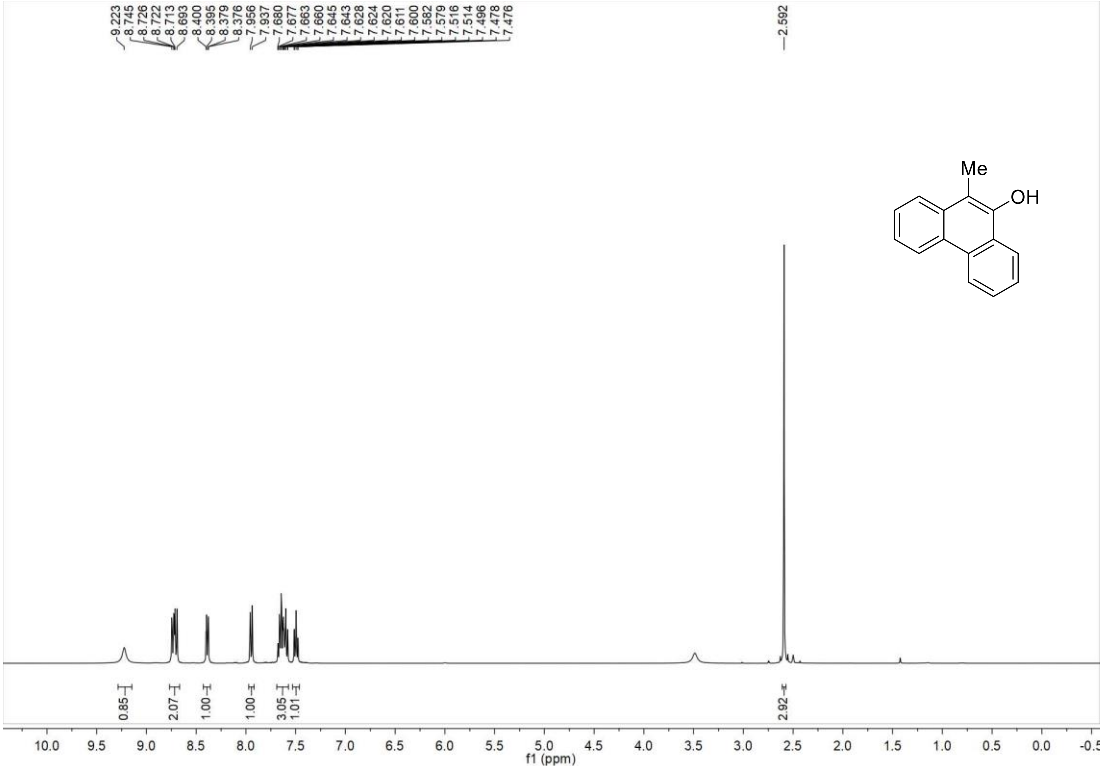

Supplementary Figure 3. ^{1}H-NMR of compound **1a**, recorded at 400 MHz and 25 °C in DMSO-d_6.

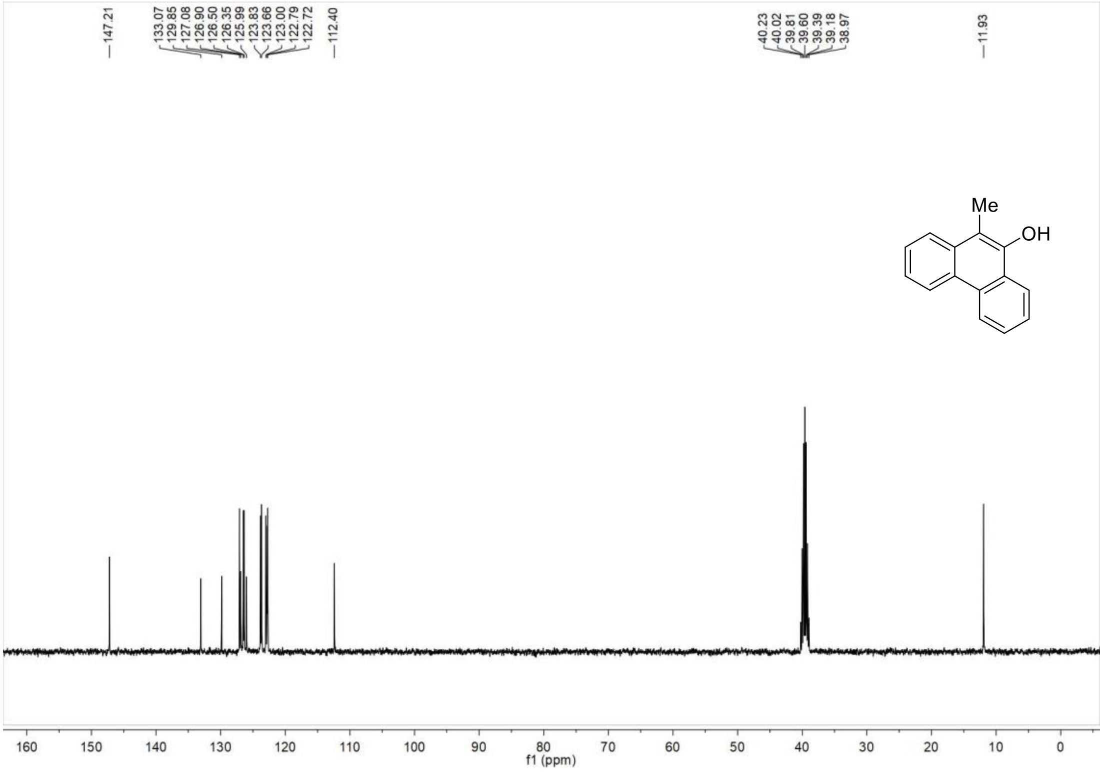

Supplementary Figure 4. ^{13}C-NMR of compound **1a**, recorded at 400 MHz and 25 °C in DMSO-d_6

10-Cyclopropylphenanthren-9-ol (2a)

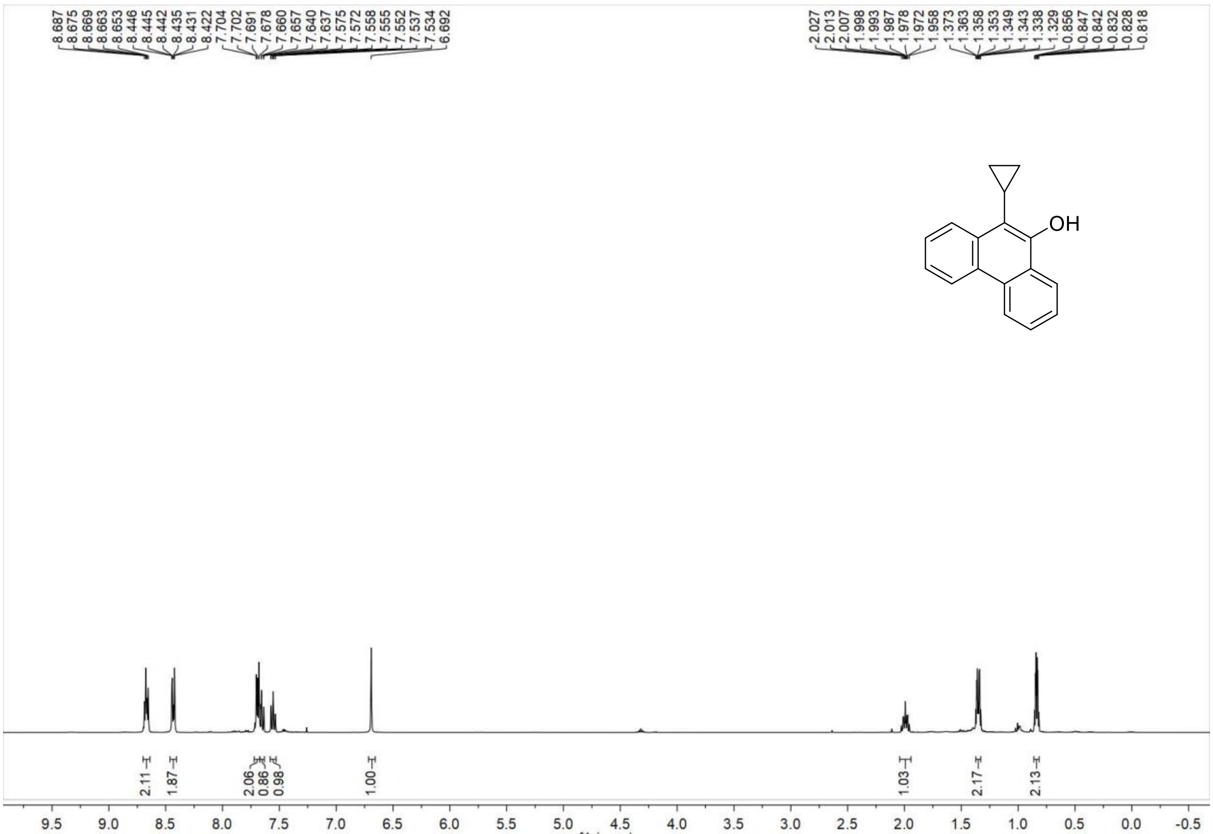

Supplementary Figure 5. ^{1}H-NMR of compound **2a**, recorded at 400 MHz and 25 °C in $CDCl_3$.

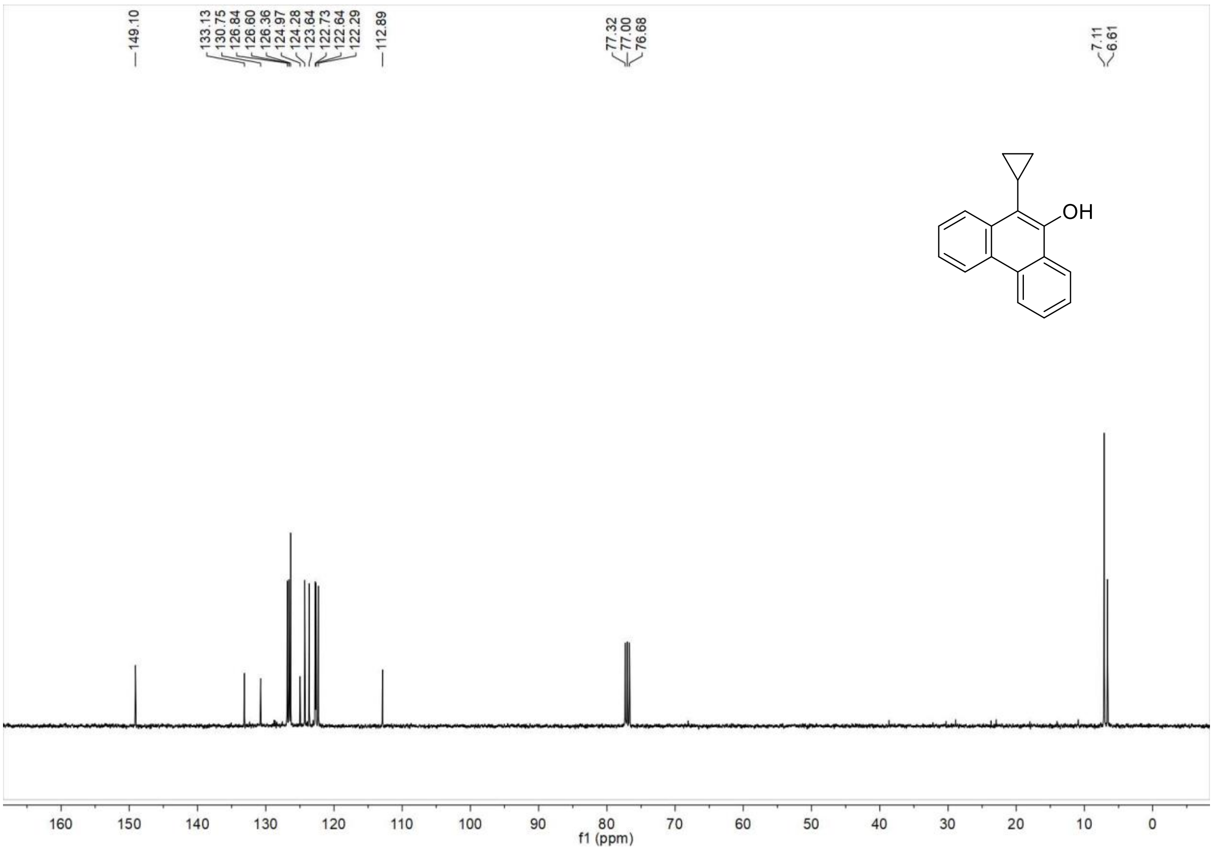

Supplementary Figure 6. ^{13}C-NMR of compound **2a**, recorded at 400 MHz and 25 °C in $CDCl_3$

10-Methyl-7-phenylphenanthren-9-ol (3a)

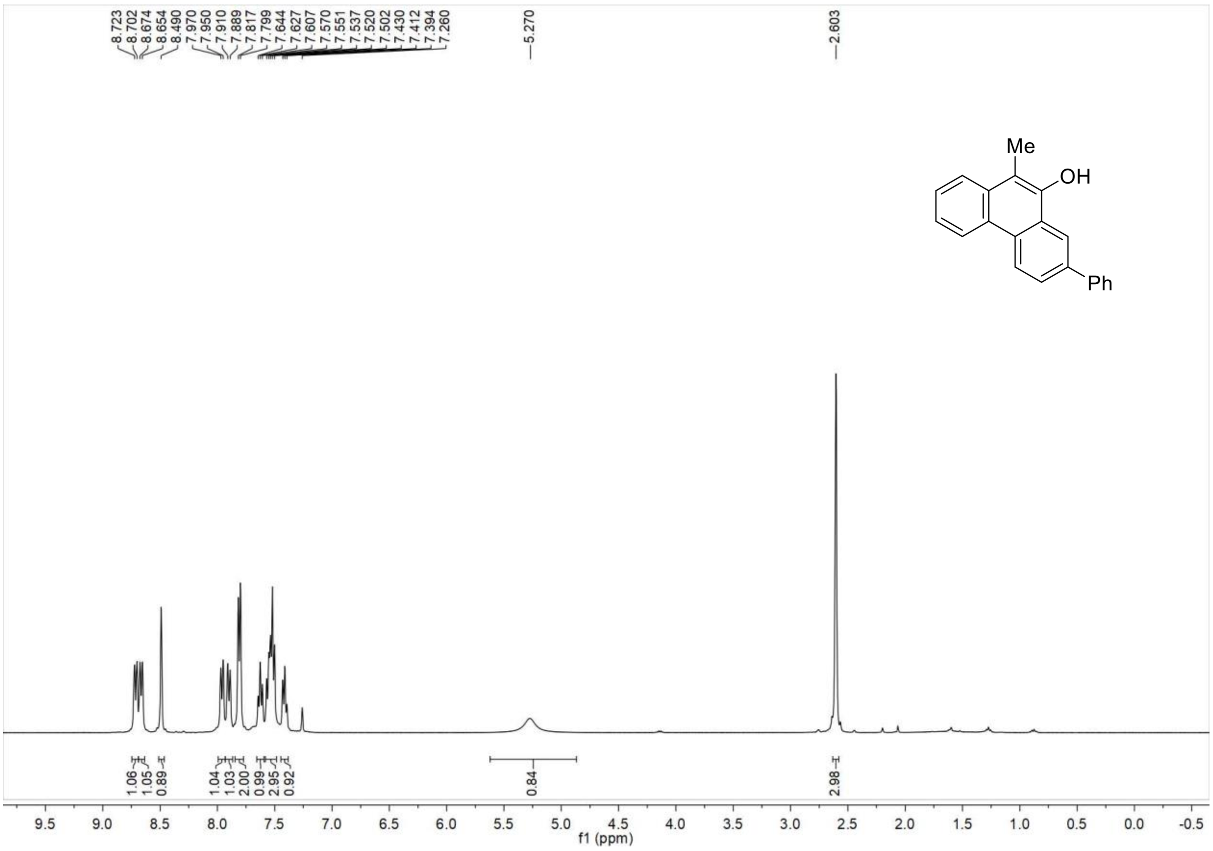

Supplementary Figure 7. ^{1}H-NMR of compound **3a**, recorded at 400 MHz and 25 °C in $CDCl_3$

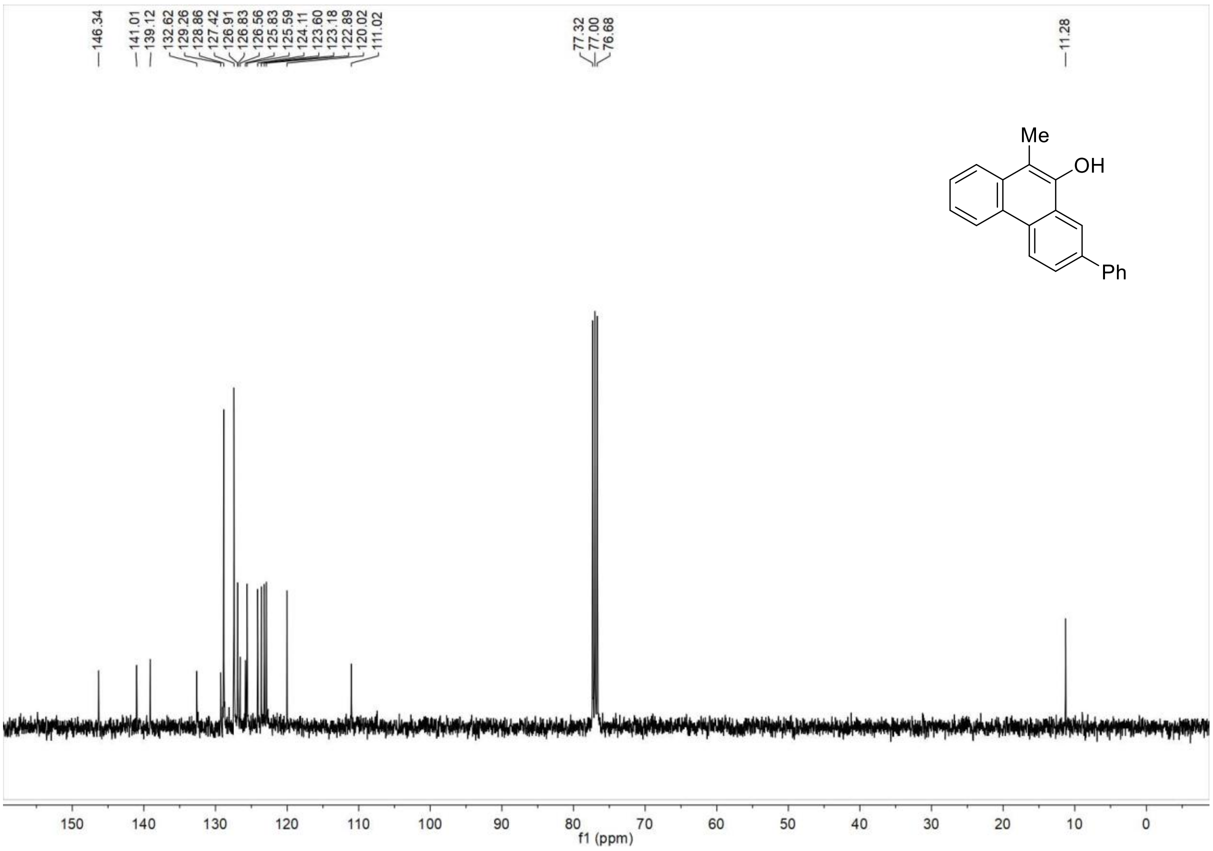

Supplementary Figure 8. ^{13}C-NMR of compound **3a**, recorded at 400 MHz and 25 °C in $CDCl_3$

2-Methoxy-10-methylphenanthren-9-ol (4a)

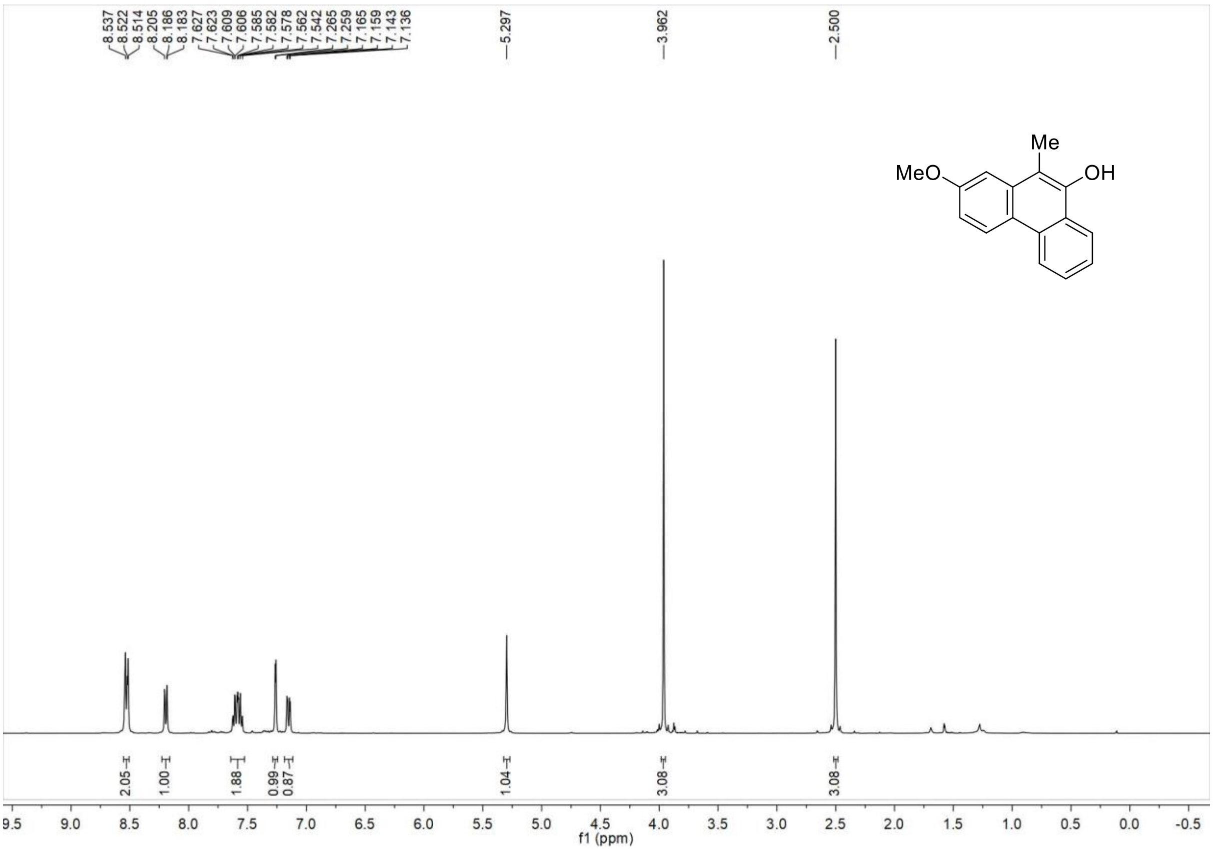

Supplementary Figure 9. ^{1}H-NMR of compound **4a**, recorded at 400 MHz and 25 °C in $CDCl_3$

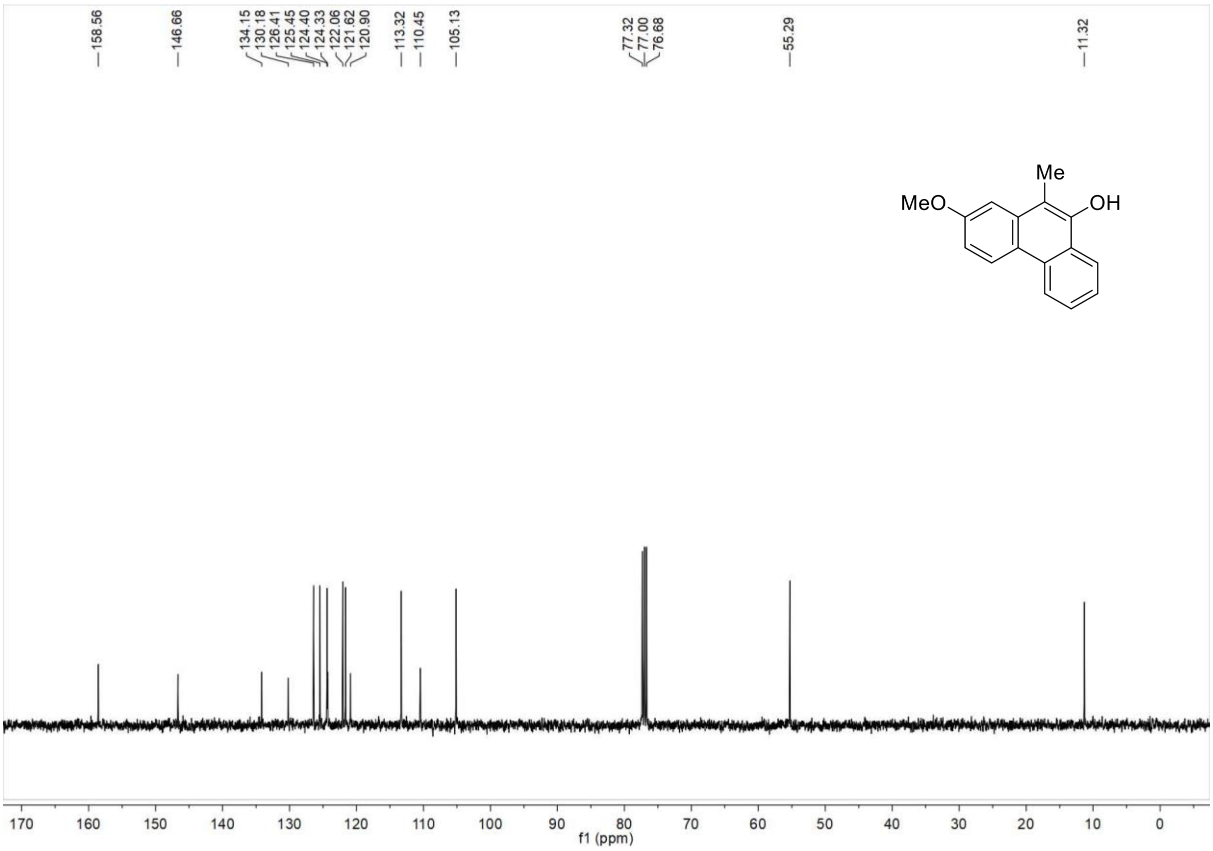

Supplementary Figure 10. ^{13}C-NMR of compound **4a**, recorded at 400 MHz and 25 °C in $CDCl_3$

7-(benzyloxy)-10-Methylphenanthren-9-ol (**5a**)

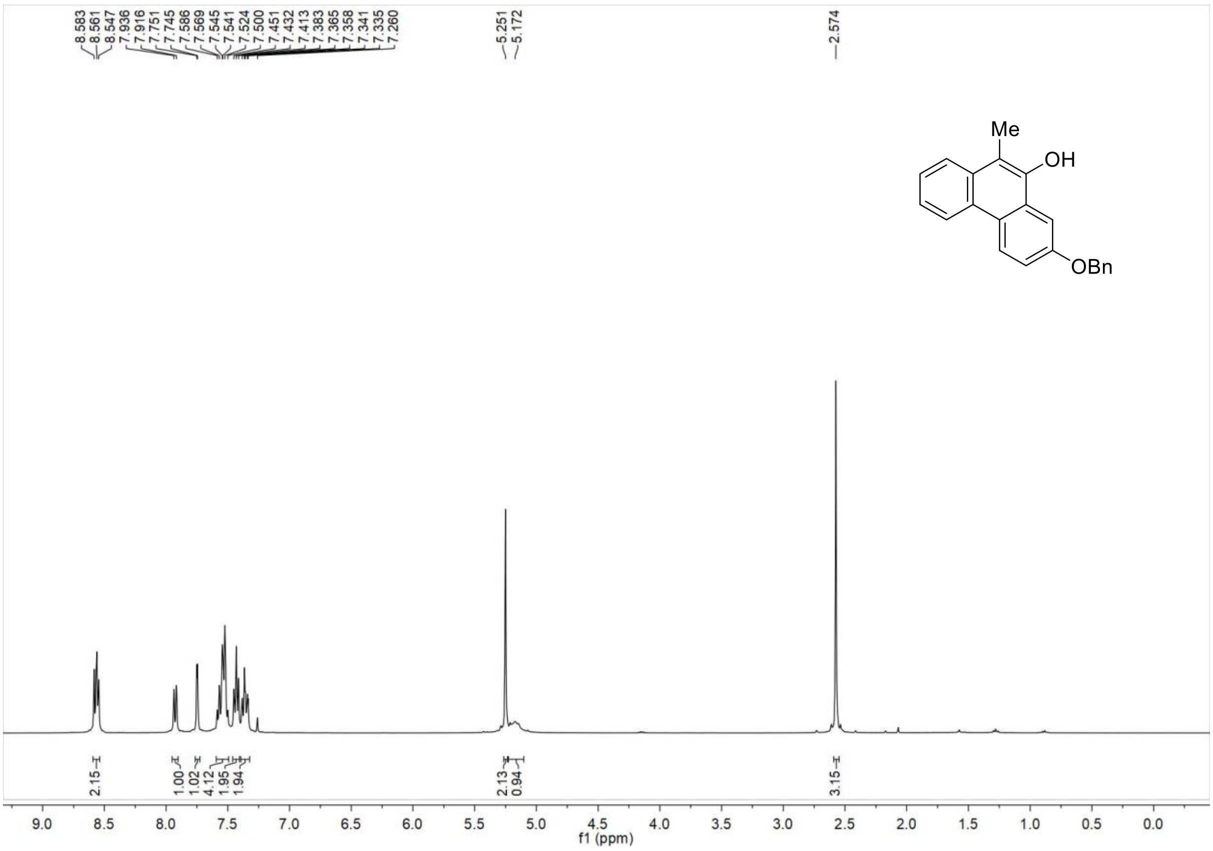

Supplementary Figure 11. ^{1}H-NMR of compound **5a**, recorded at 400 MHz and 25 °C in $CDCl_3$

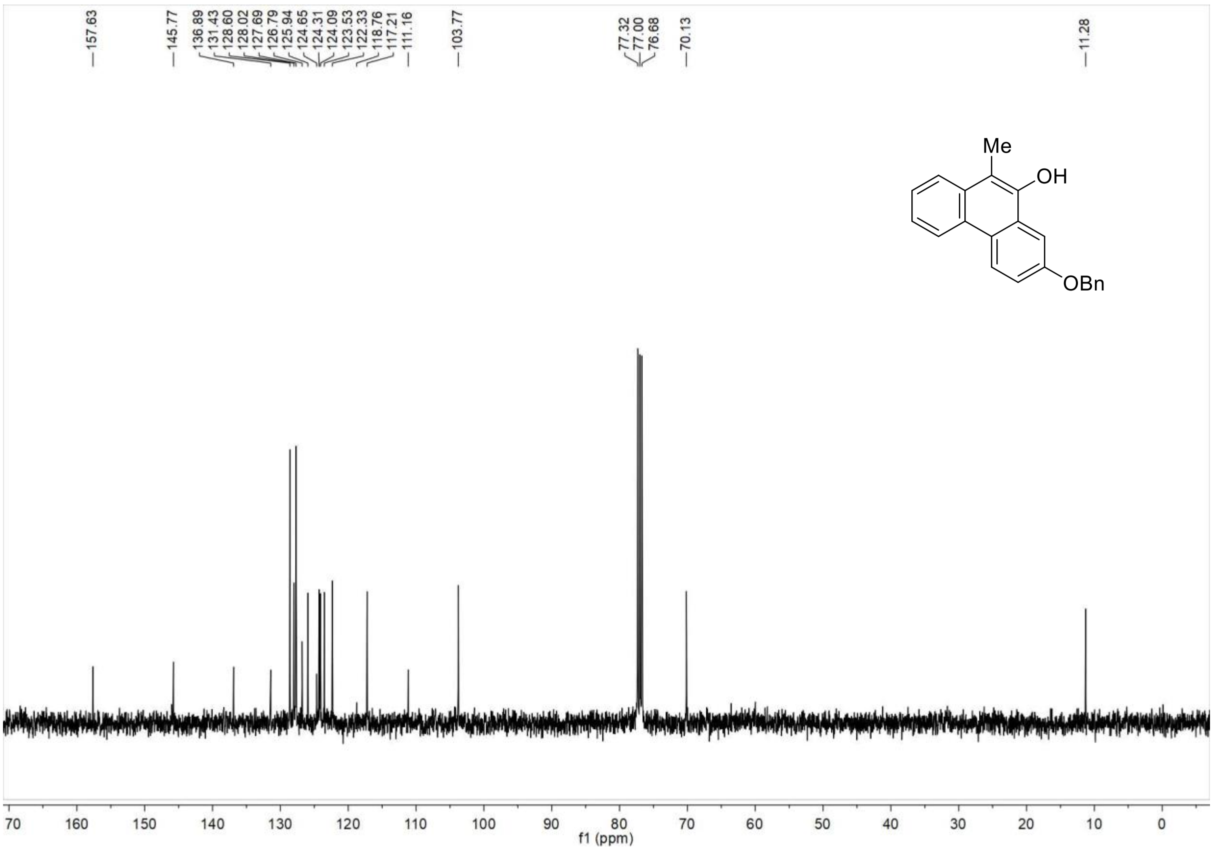

Supplementary Figure 12. ^{13}C-NMR of compound **5a**, recorded at 400 MHz and 25 °C in $CDCl_3$

5-Methylphenanthro[2,3-*d*][1,3]dioxol-6-ol (6a)

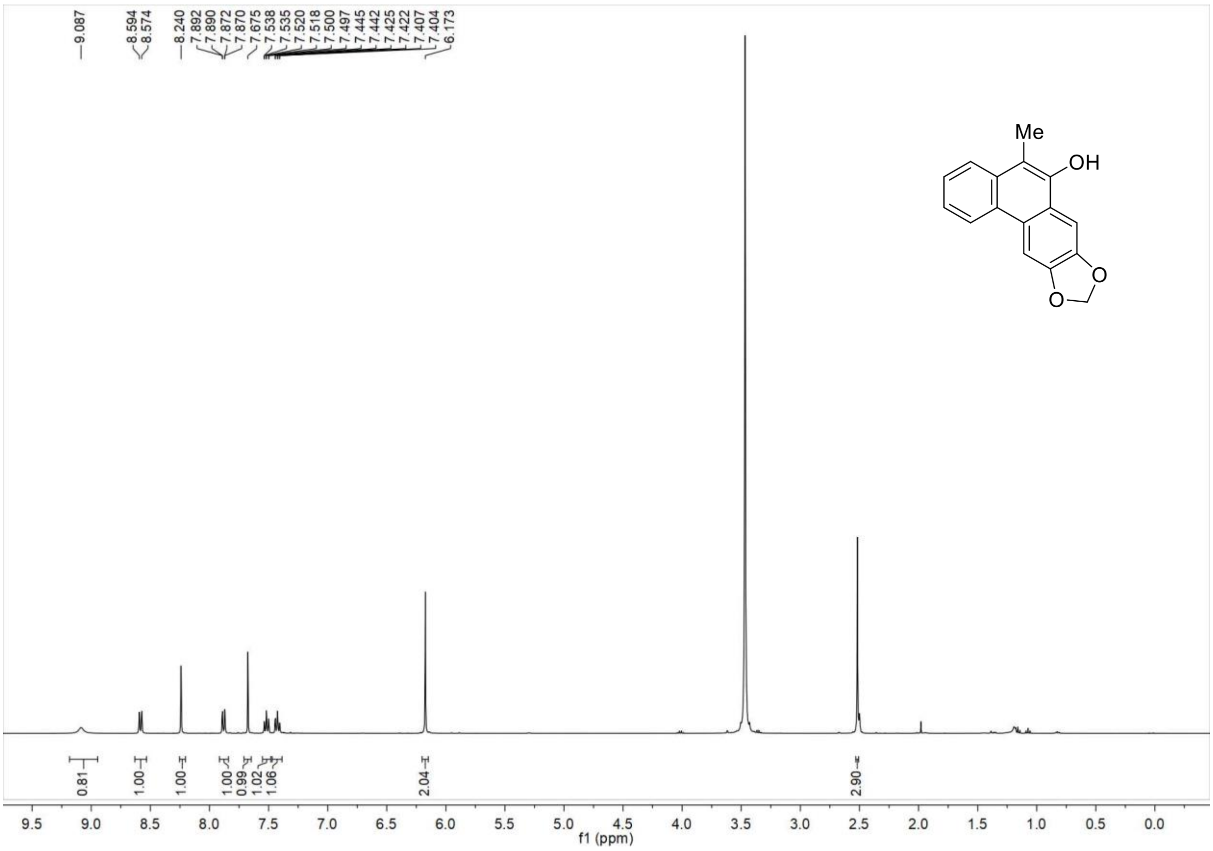

Supplementary Figure 13. ^{1}H-NMR of compound **6a**, recorded at 400 MHz and 25 °C in DMSO-d_6

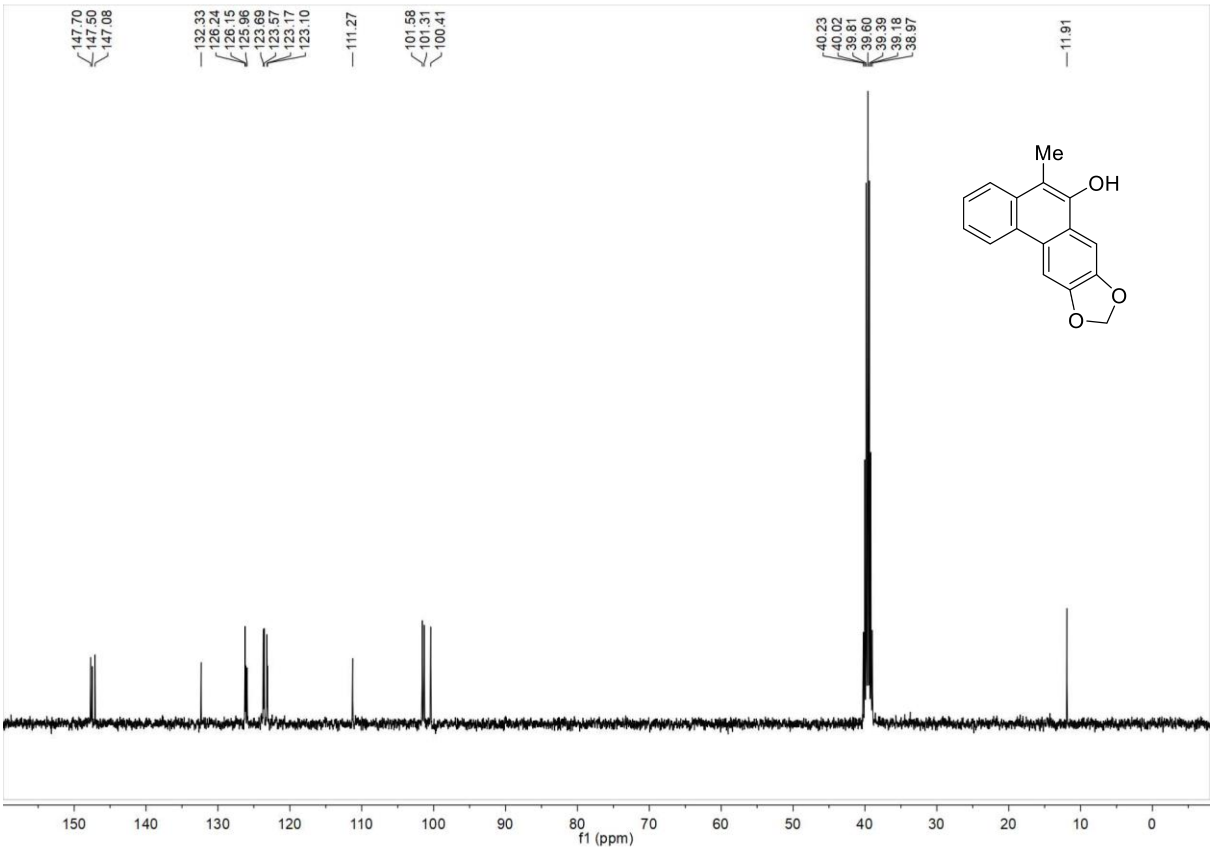

Supplementary Figure 14. ^{13}C-NMR of compound **6a**, recorded at 400 MHz and 25 °C in DMSO-d_6

3-Fluoro-10-methylphenanthren-9-ol (**7a**)

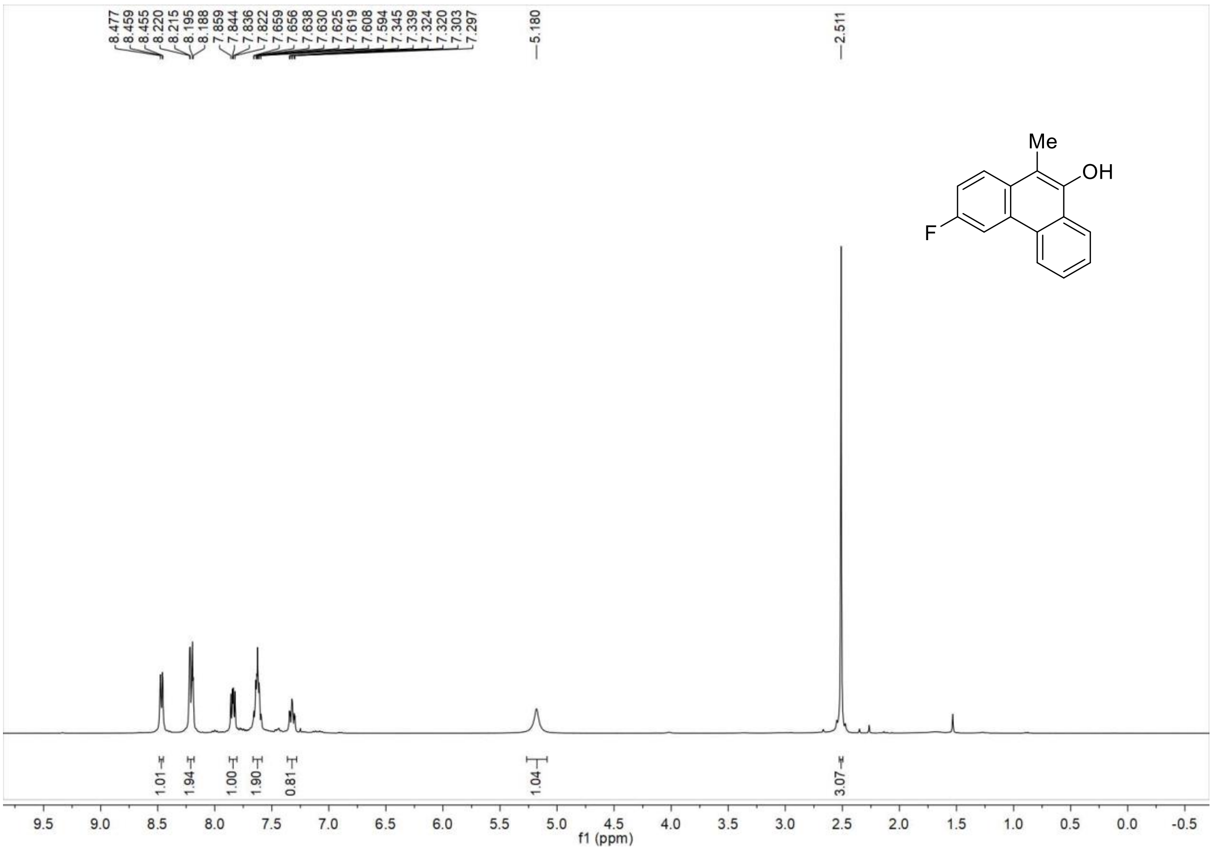

Supplementary Figure 15. ^{1}H-NMR of compound **7a**, recorded at 400 MHz and 25 °C in $CDCl_3$

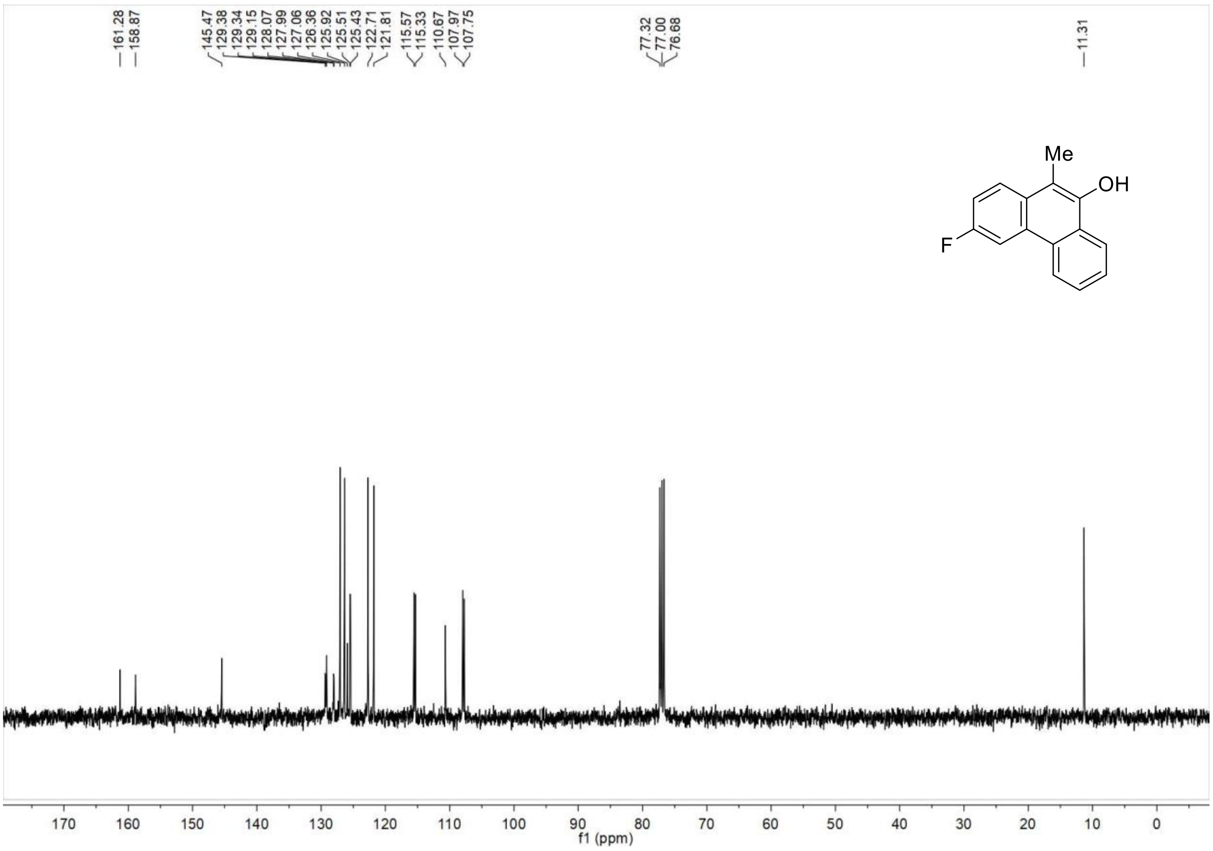

Supplementary Figure 16. ^{13}C-NMR of compound **7a**, recorded at 400 MHz and 25 °C in $CDCl_3$

6-Chloro-10-methylphenanthren-9-ol (8a)

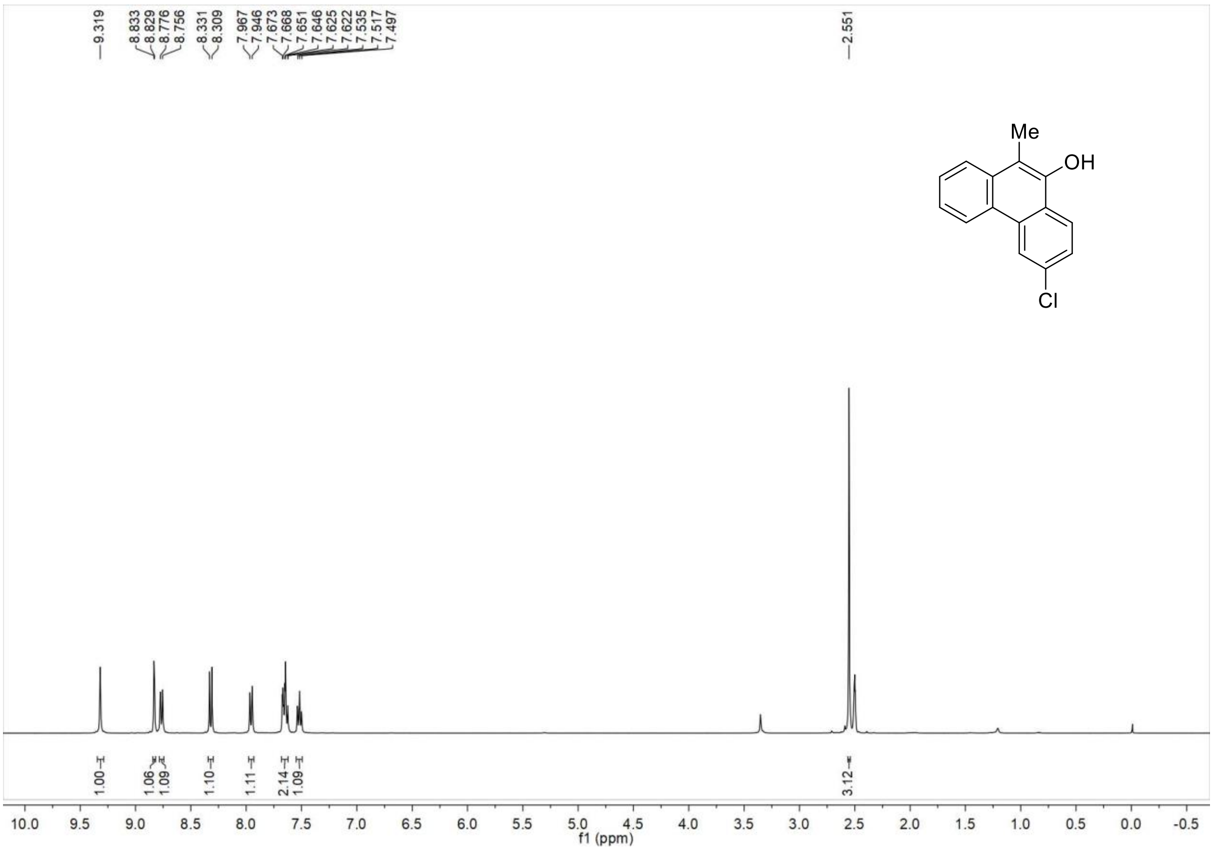

Supplementary Figure 17. ^{1}H-NMR of compound **8a**, recorded at 400 MHz and 25 °C in DMSO-*d*6

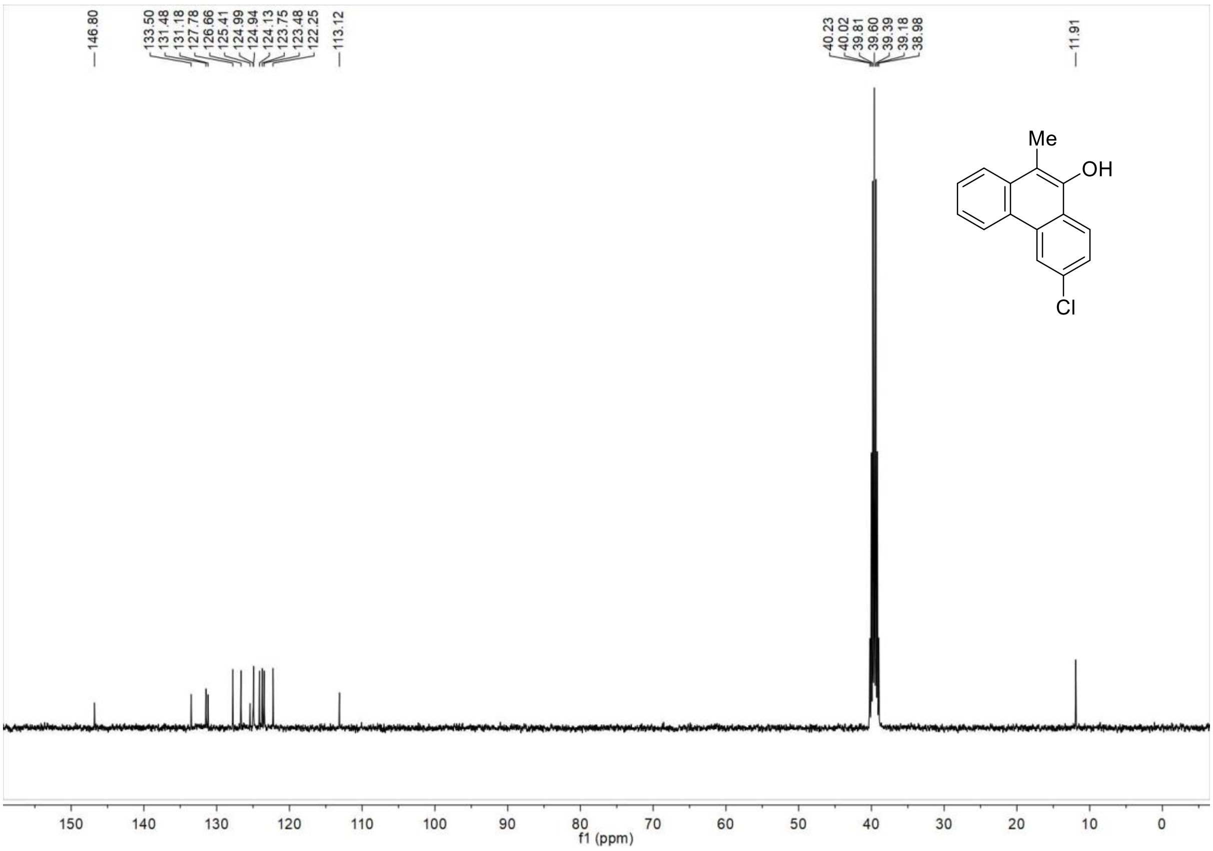

Supplementary Figure 18. ^{13}C-NMR of compound **8a**, recorded at 400 MHz and 25 °C in DMSO-*d*6

***tert*-Butyl 10-hydroxy-9-methylphenanthrene-3-carboxylate (9a)**

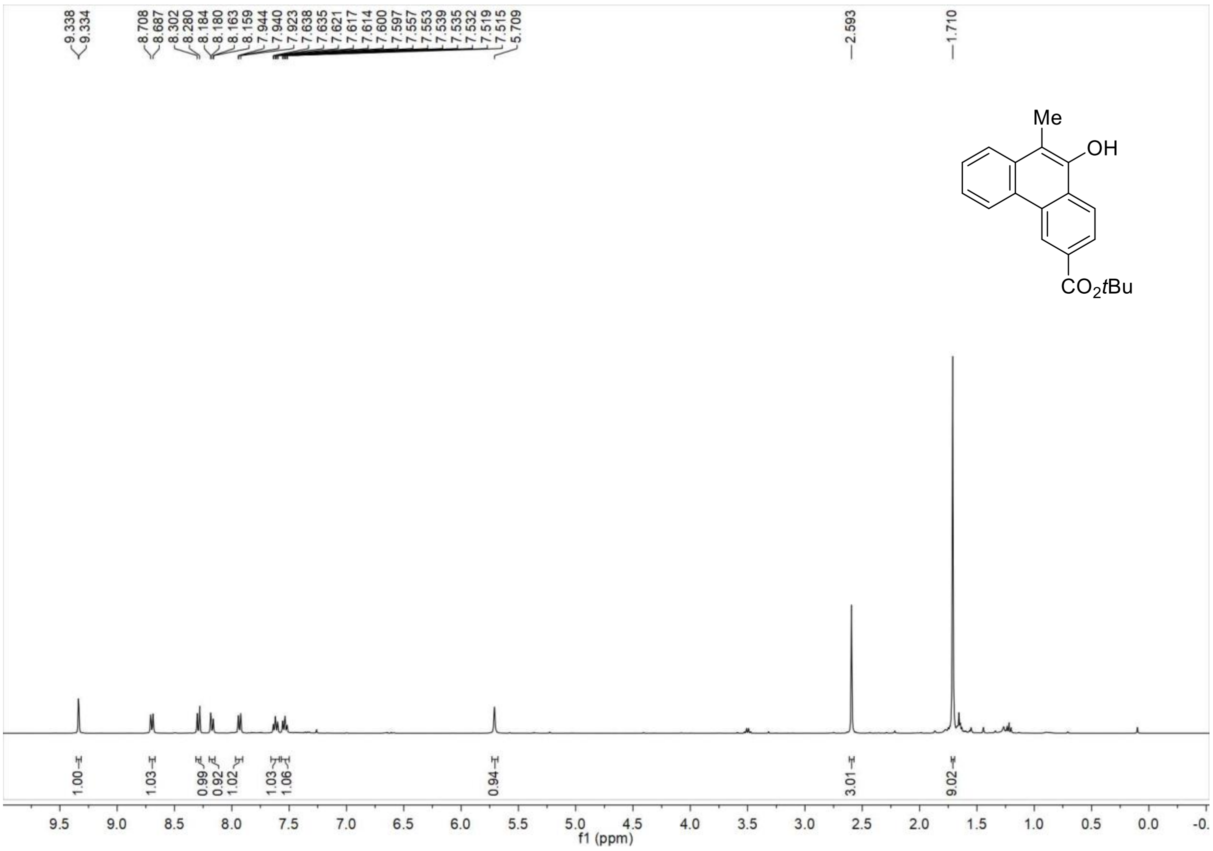

Supplementary Figure 19. ^{1}H-NMR of compound **9a**, recorded at 400 MHz and 25 °C in $CDCl_3$

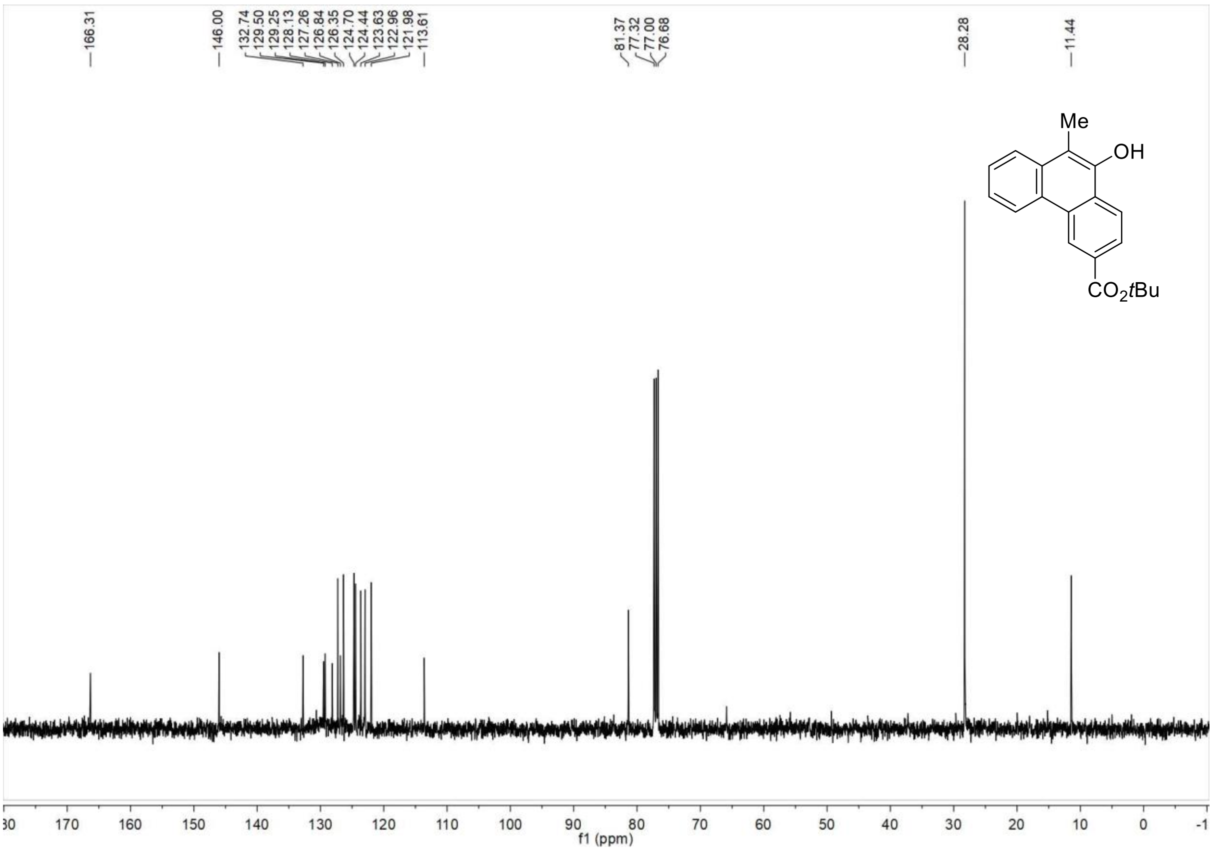

Supplementary Figure 20. ^{13}C-NMR of compound **9a**, recorded at 400 MHz and 25 °C in $CDCl_3$

10-Methyl-7-(trifluoromethyl)phenanthren-9-ol (10a)

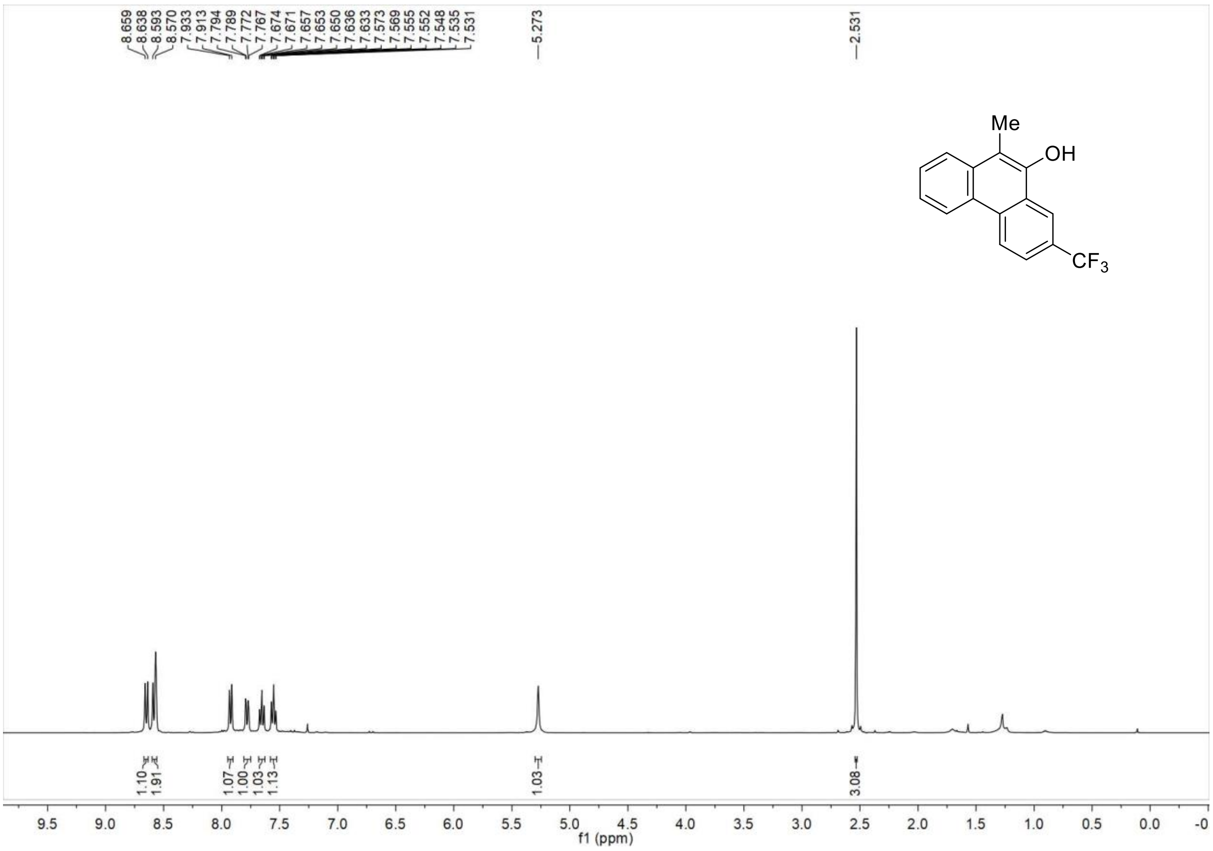

Supplementary Figure 21. ^{1}H-NMR of compound **10a**, recorded at 400 MHz and 25 °C in $CDCl_3$

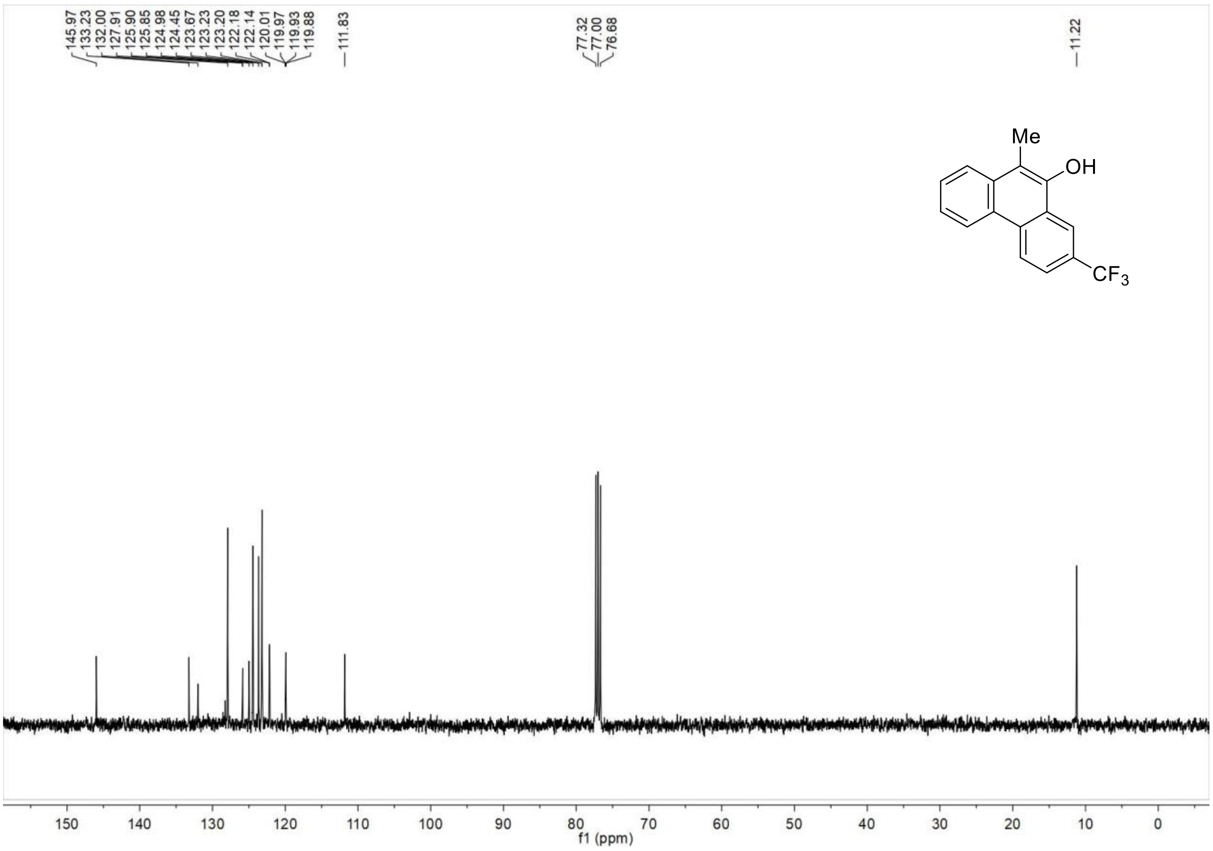

Supplementary Figure 22. ^{13}C-NMR of compound **10a**, recorded at 400 MHz and 25 °C in $CDCl_3$

1-Methylnaphthalen-2-ol (11a)

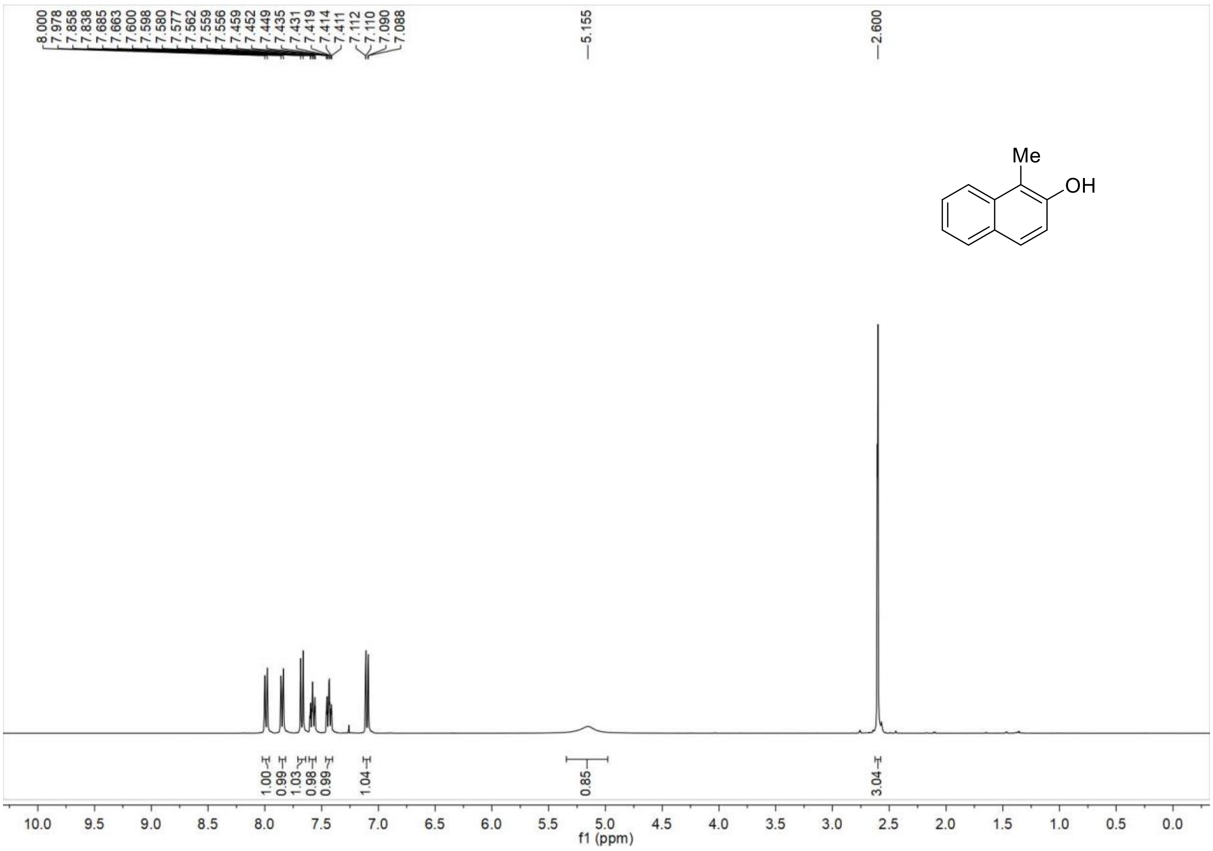

Supplementary Figure 23. ^{1}H-NMR of compound **11a**, recorded at 400 MHz and 25 °C in $CDCl_3$

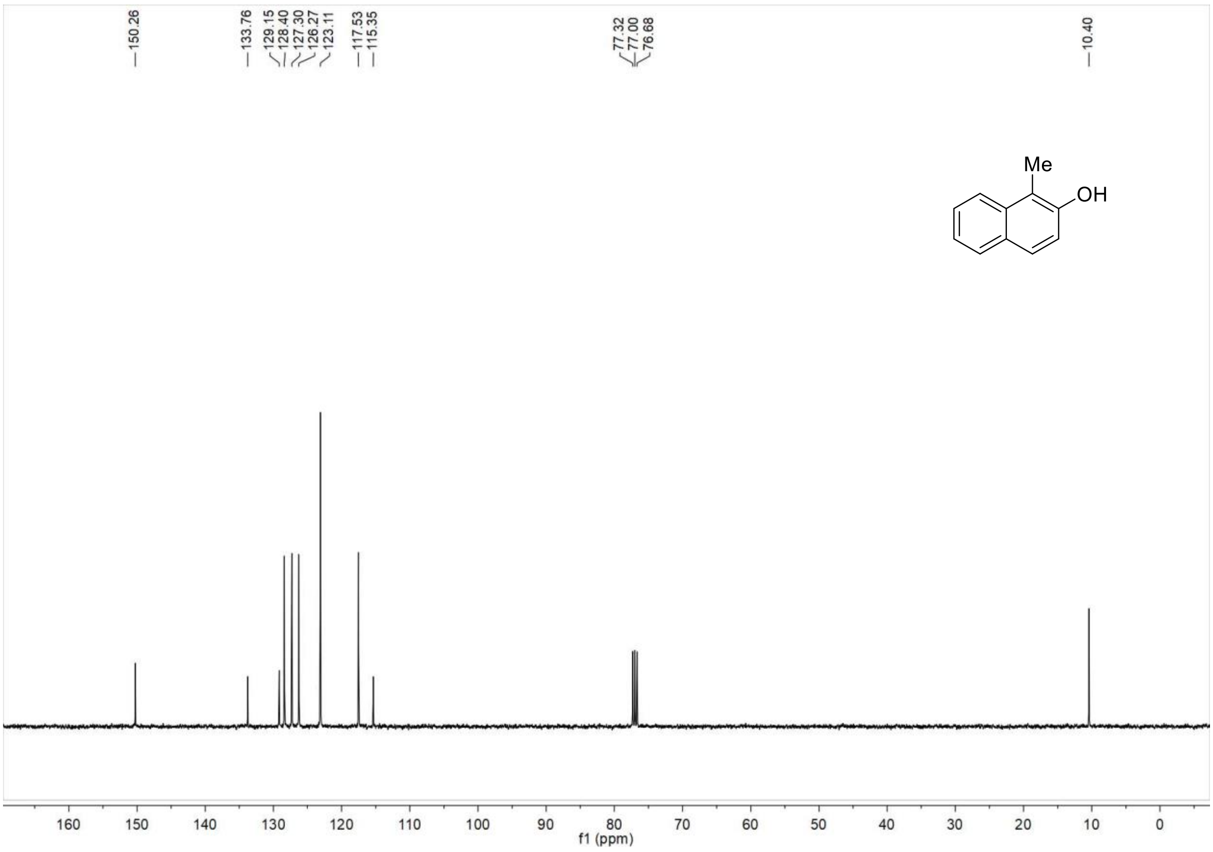

Supplementary Figure 24. ^{13}C-NMR of compound **11a**, recorded at 400 MHz and 25 °C in $CDCl_3$

1-Octylnaphthalen-2-ol (12a)

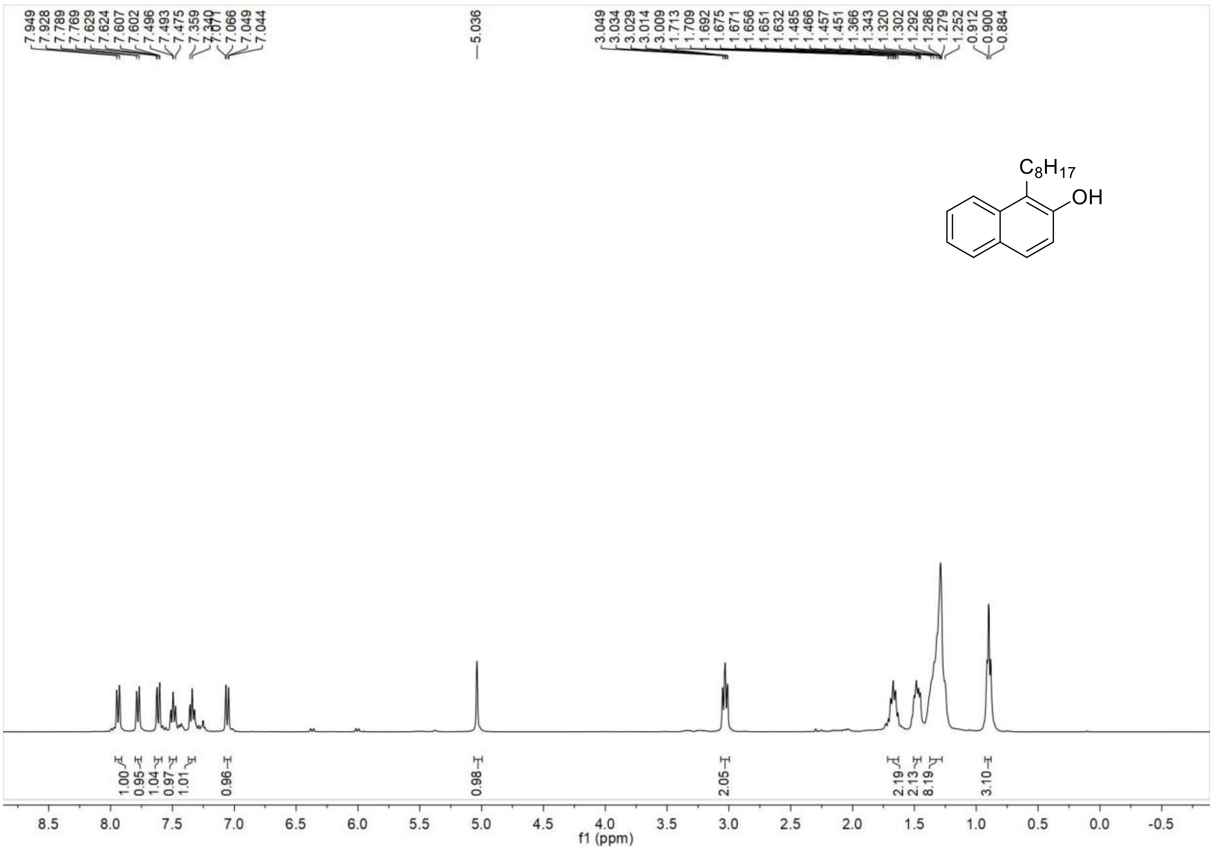

Supplementary Figure 25. ^{1}H-NMR of compound **12a**, recorded at 400 MHz and 25 °C in $CDCl_3$

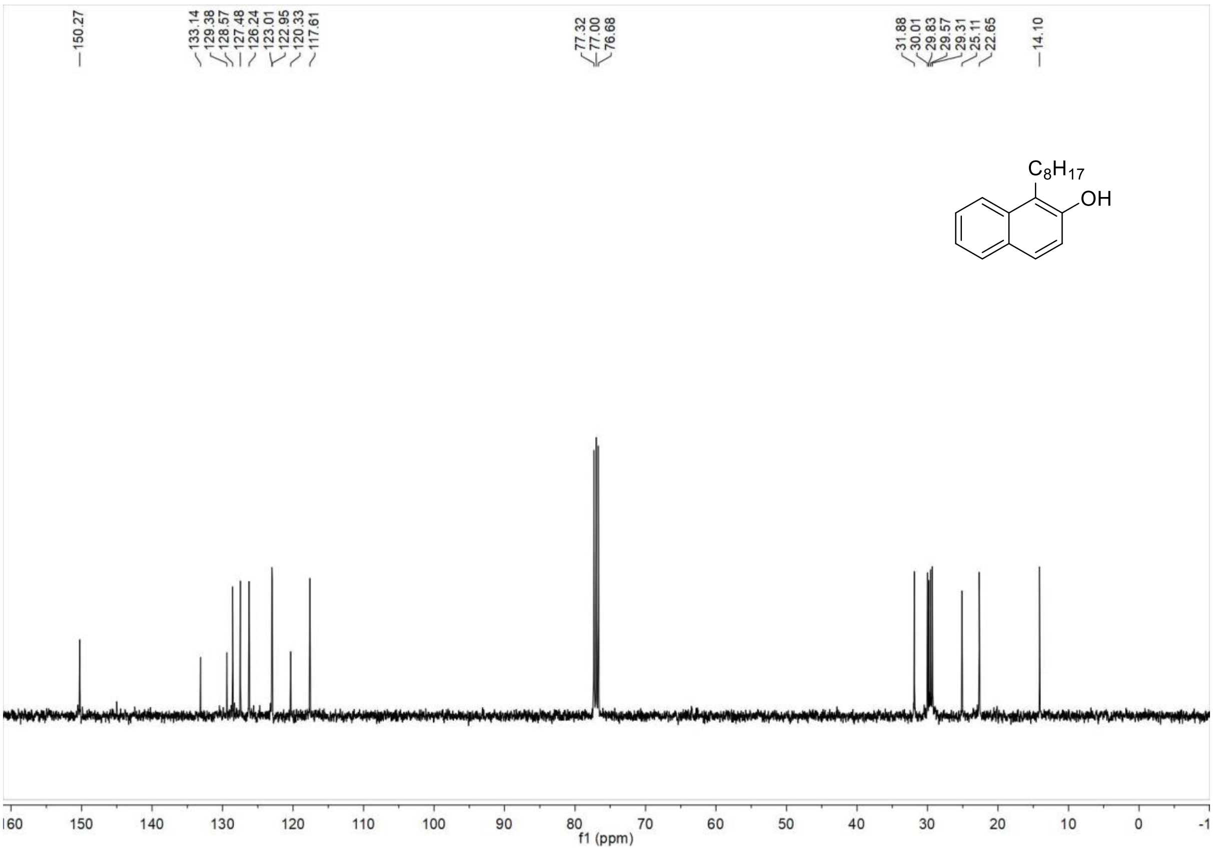

Supplementary Figure 26. ^{13}C-NMR of compound **12a**, recorded at 400 MHz and 25 °C in $CDCl_3$

1,3-Dimethylnaphthalen-2-ol (13a)

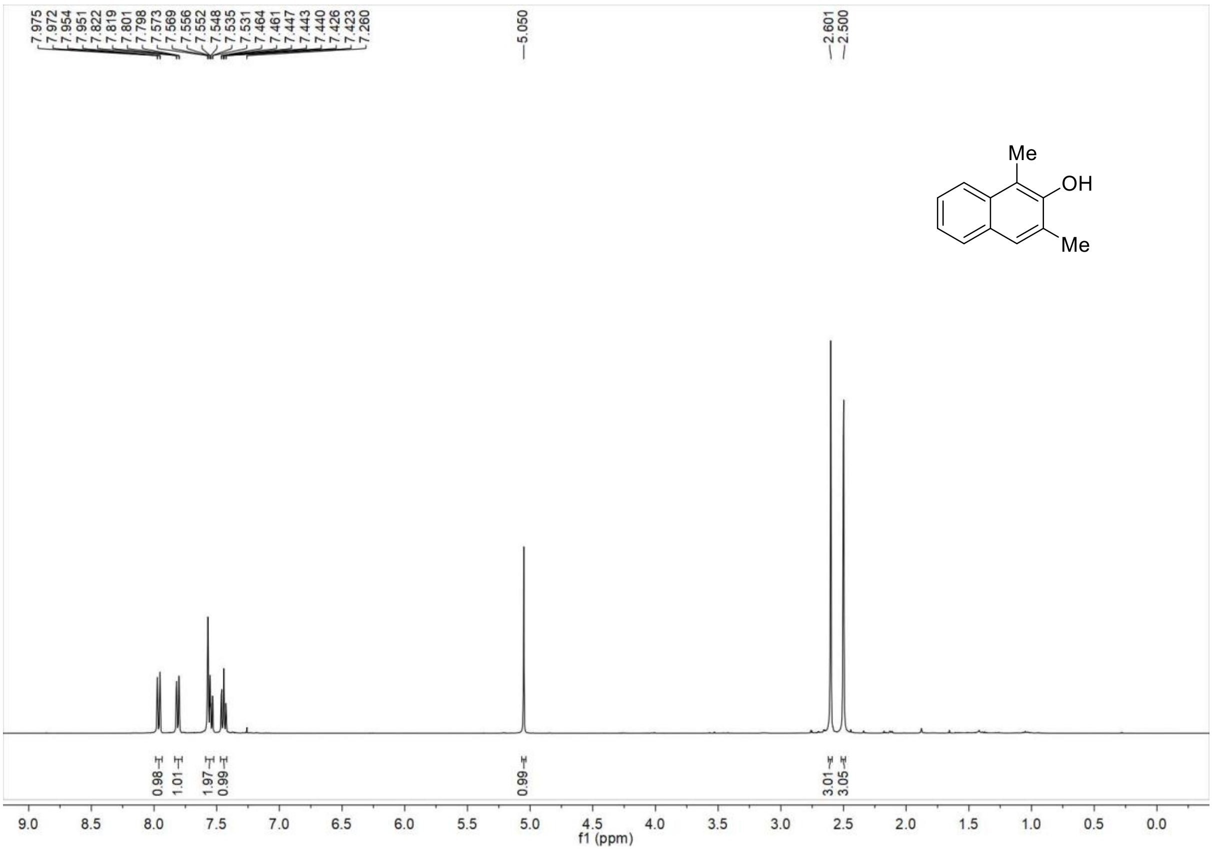

Supplementary Figure 27. ^{1}H-NMR of compound **13a**, recorded at 400 MHz and 25 °C in $CDCl_3$

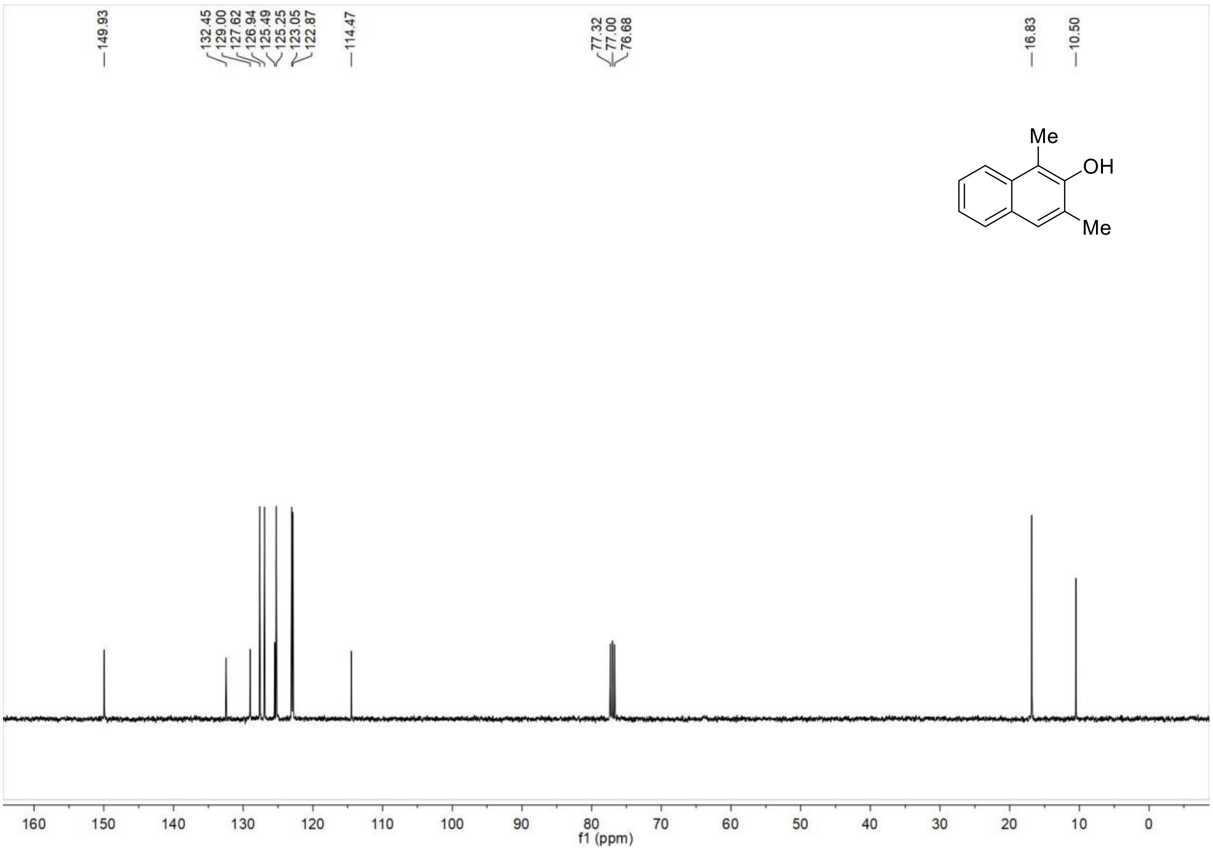

Supplementary Figure 28. ^{13}C-NMR of compound **13a**, recorded at 400 MHz and 25 °C in $CDCl_3$

1-Methyl-3-phenylnaphthalen-2-ol (14a)

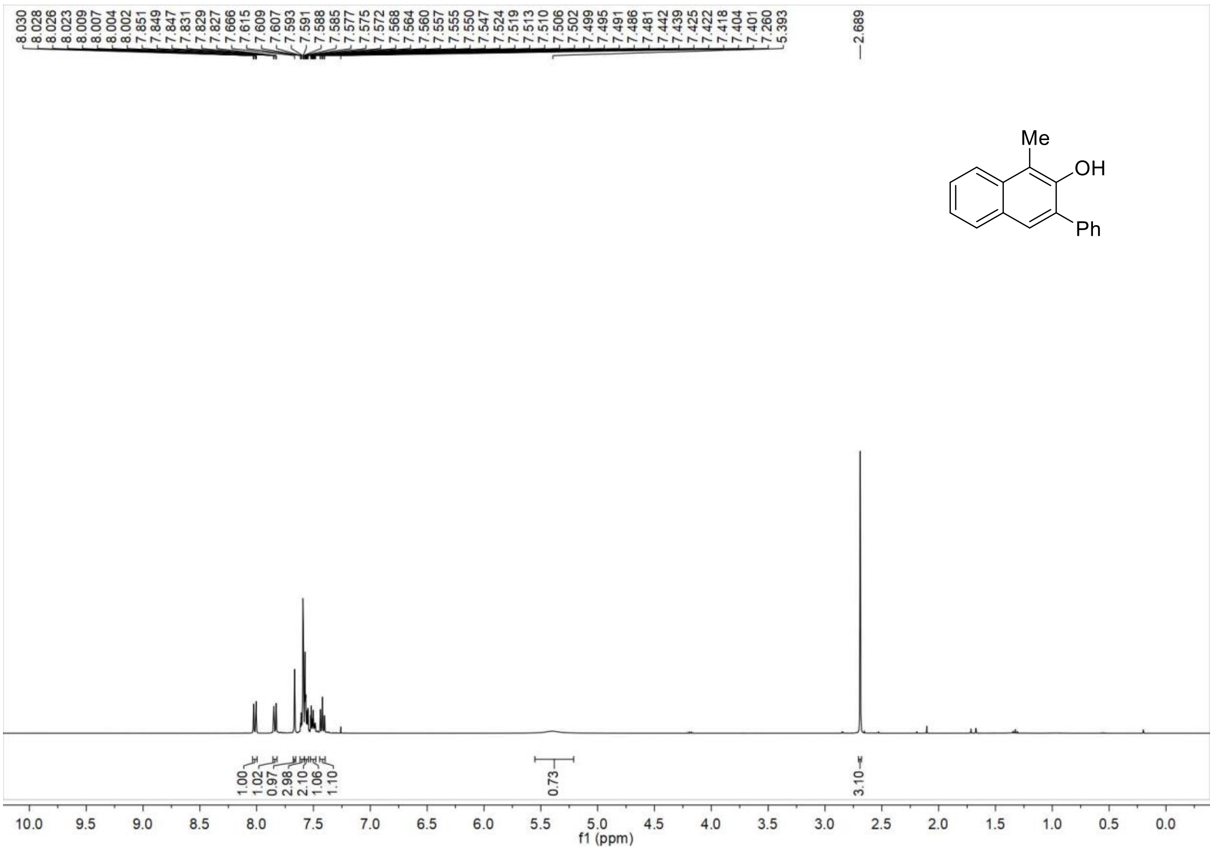

Supplementary Figure 29. ^{1}H-NMR of compound **14a**, recorded at 400 MHz and 25 °C in $CDCl_3$

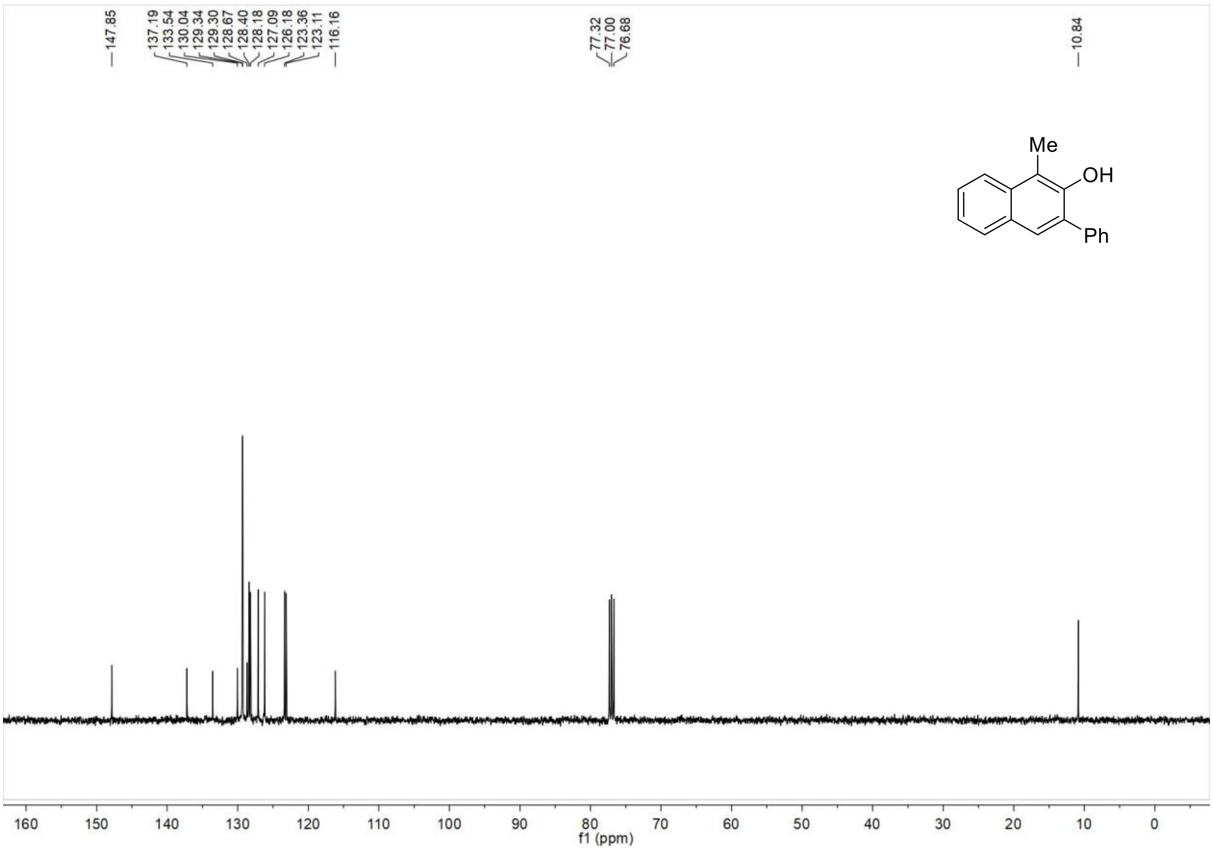

Supplementary Figure 30. ^{13}C-NMR of compound **14a**, recorded at 400 MHz and 25 °C in $CDCl_3$

3-Benzyl-1-methylnaphthalen-2-ol (15a)

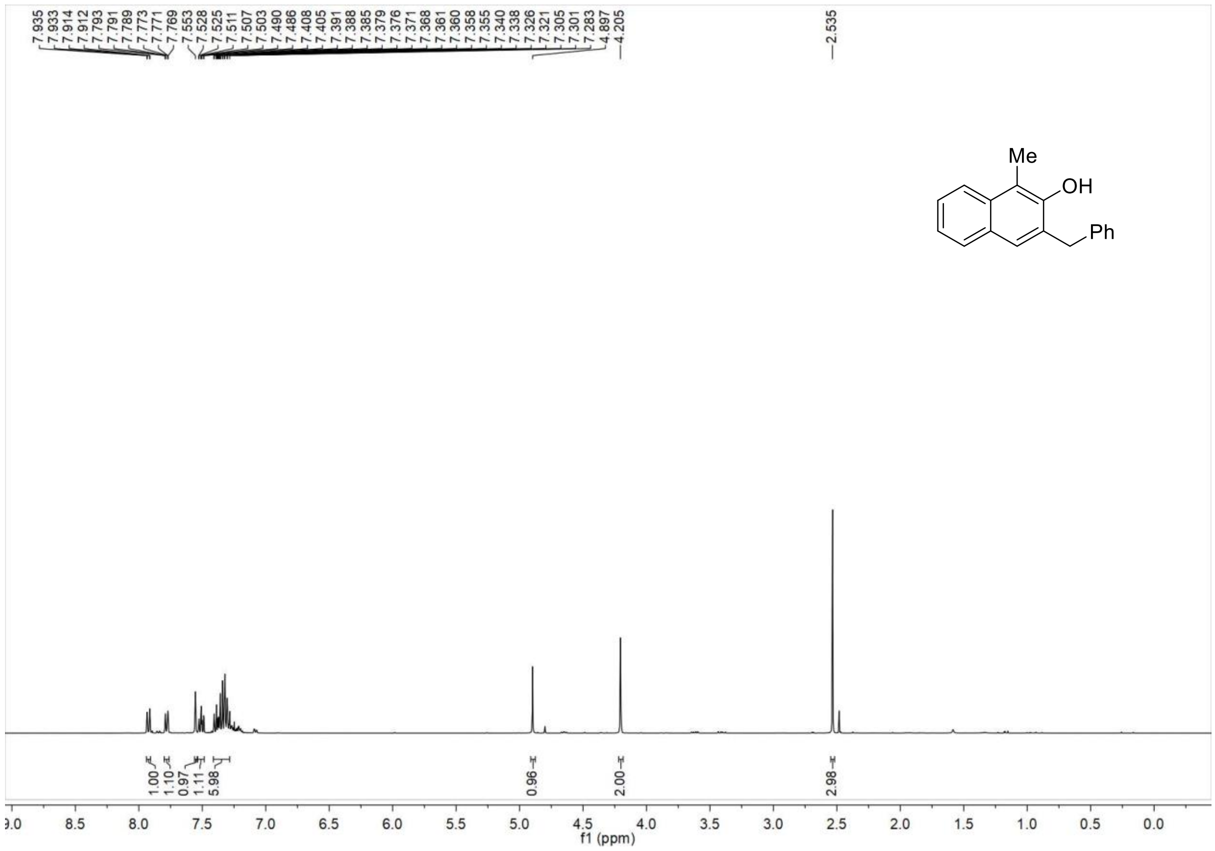

Supplementary Figure 31. ^{1}H-NMR of compound **15a**, recorded at 400 MHz and 25 °C in $CDCl_3$

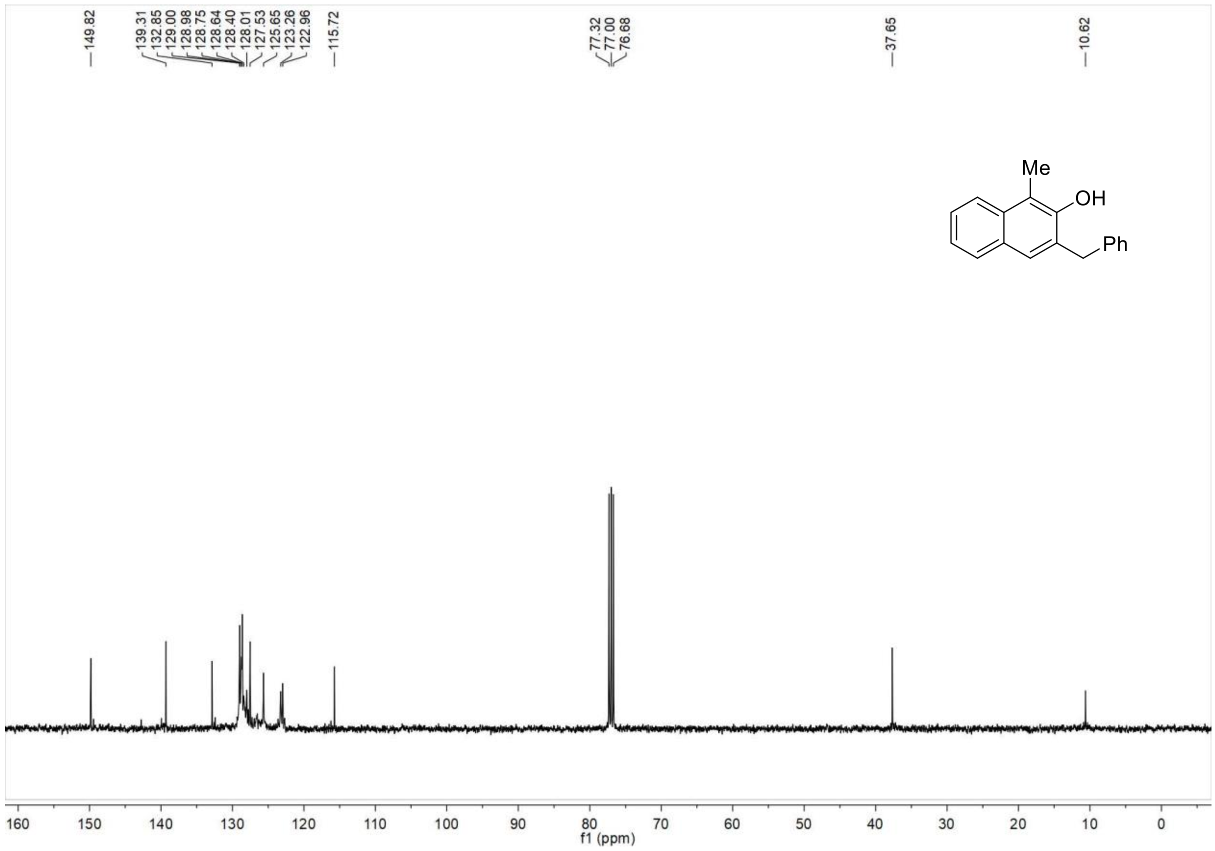

Supplementary Figure 32. ^{13}C-NMR of compound **15a**, recorded at 400 MHz and 25 °C in $CDCl_3$

3-(benzo[*d*][1,3]dioxol-5-yl)-1-Methylnaphthalen-2-ol (16a)

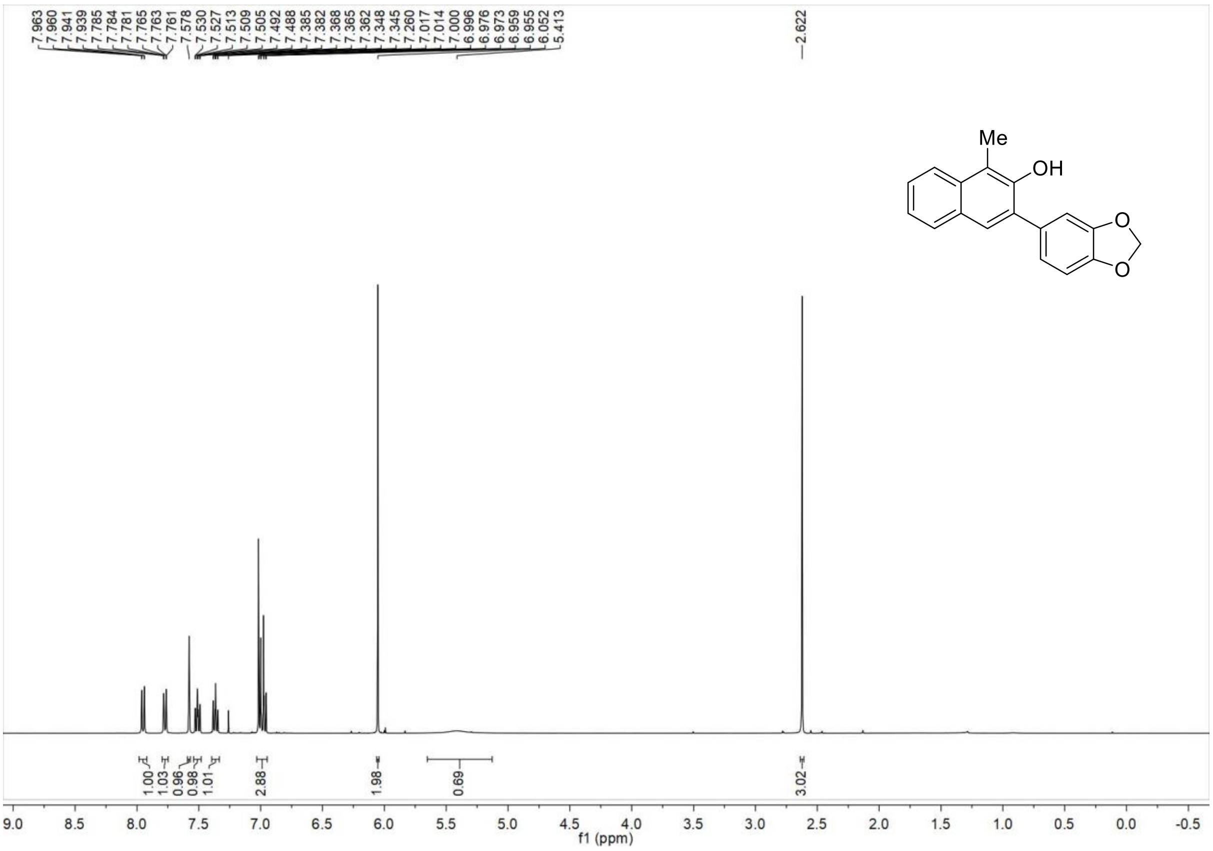

Supplementary Figure 33. ^{1}H-NMR of compound **16a**, recorded at 400 MHz and 25 °C in $CDCl_3$

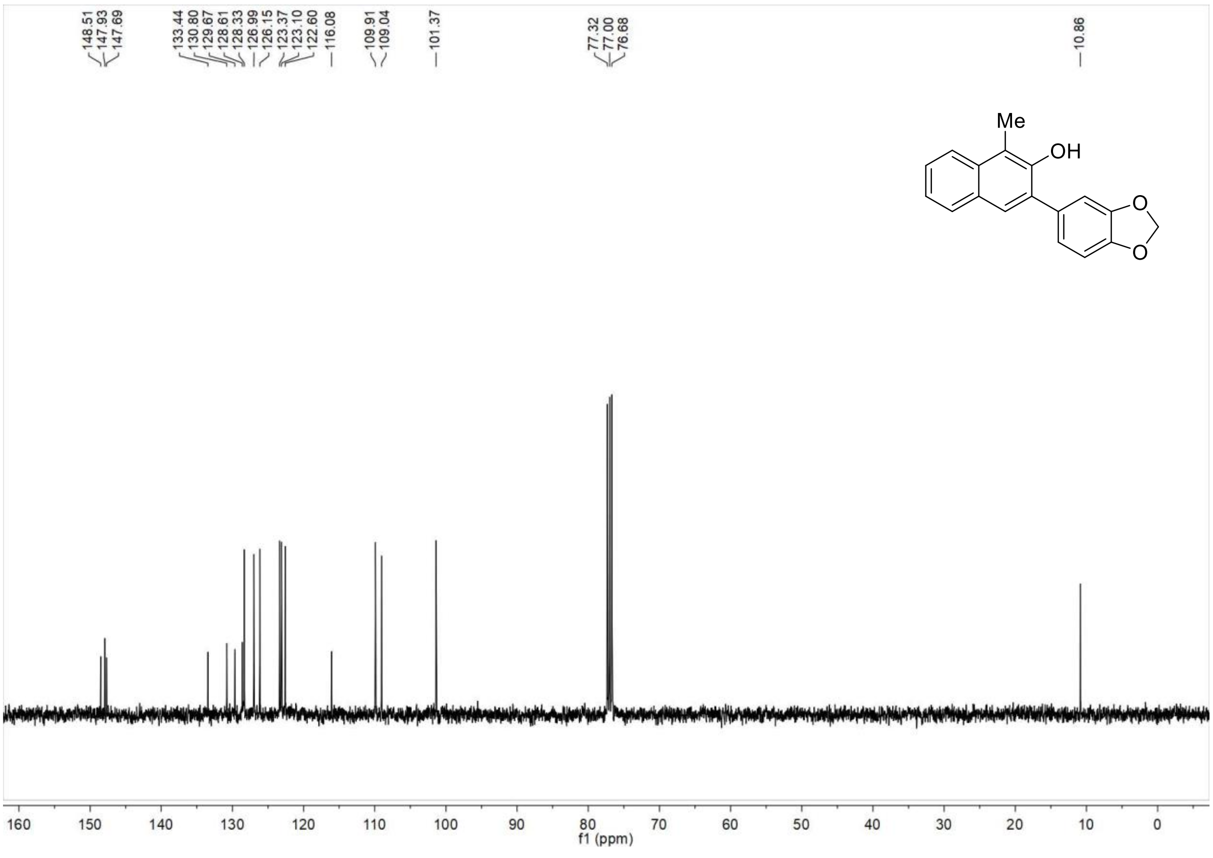

Supplementary Figure 34. ^{13}C-NMR of compound **16a**, recorded at 400 MHz and 25 °C in $CDCl_3$

3-Bromo-1-methylnaphthalen-2-ol (17a)

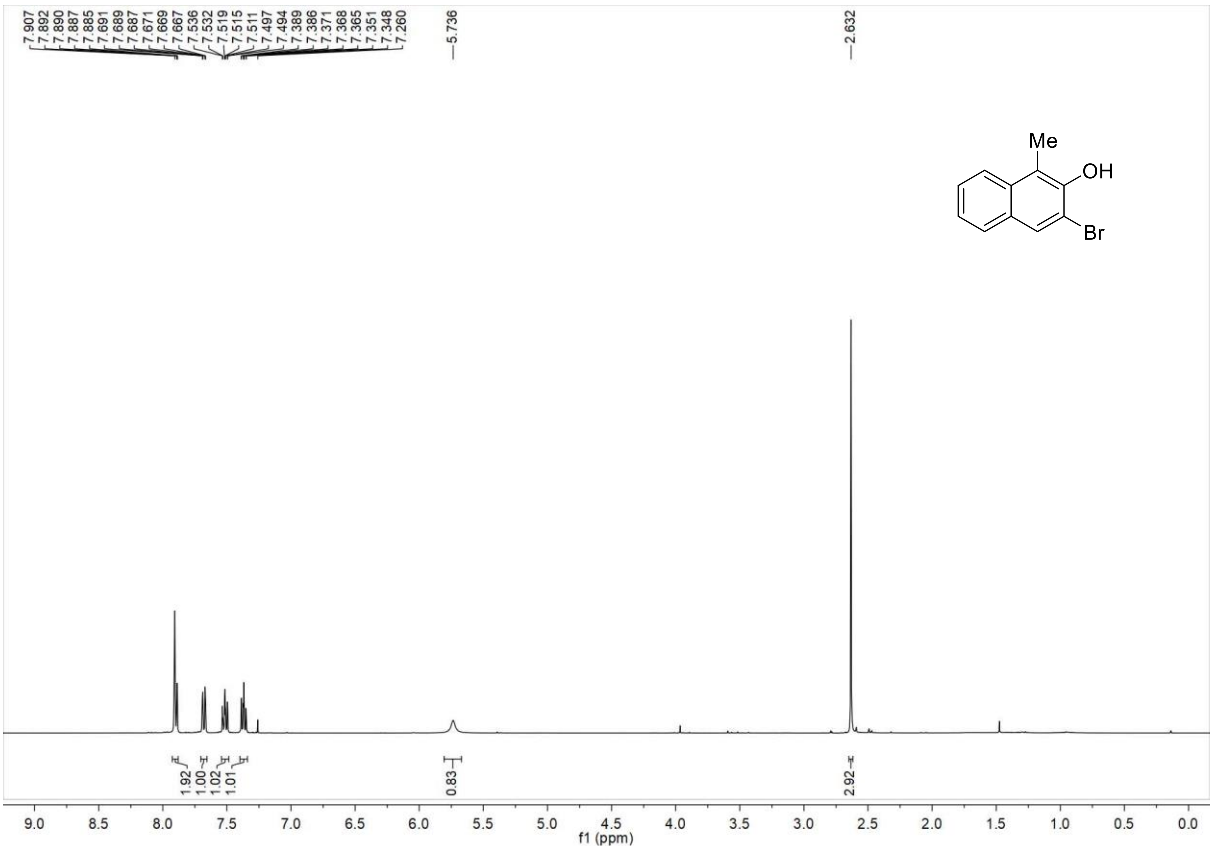

Supplementary Figure 35. ^{1}H-NMR of compound **17a**, recorded at 400 MHz and 25 °C in $CDCl_3$

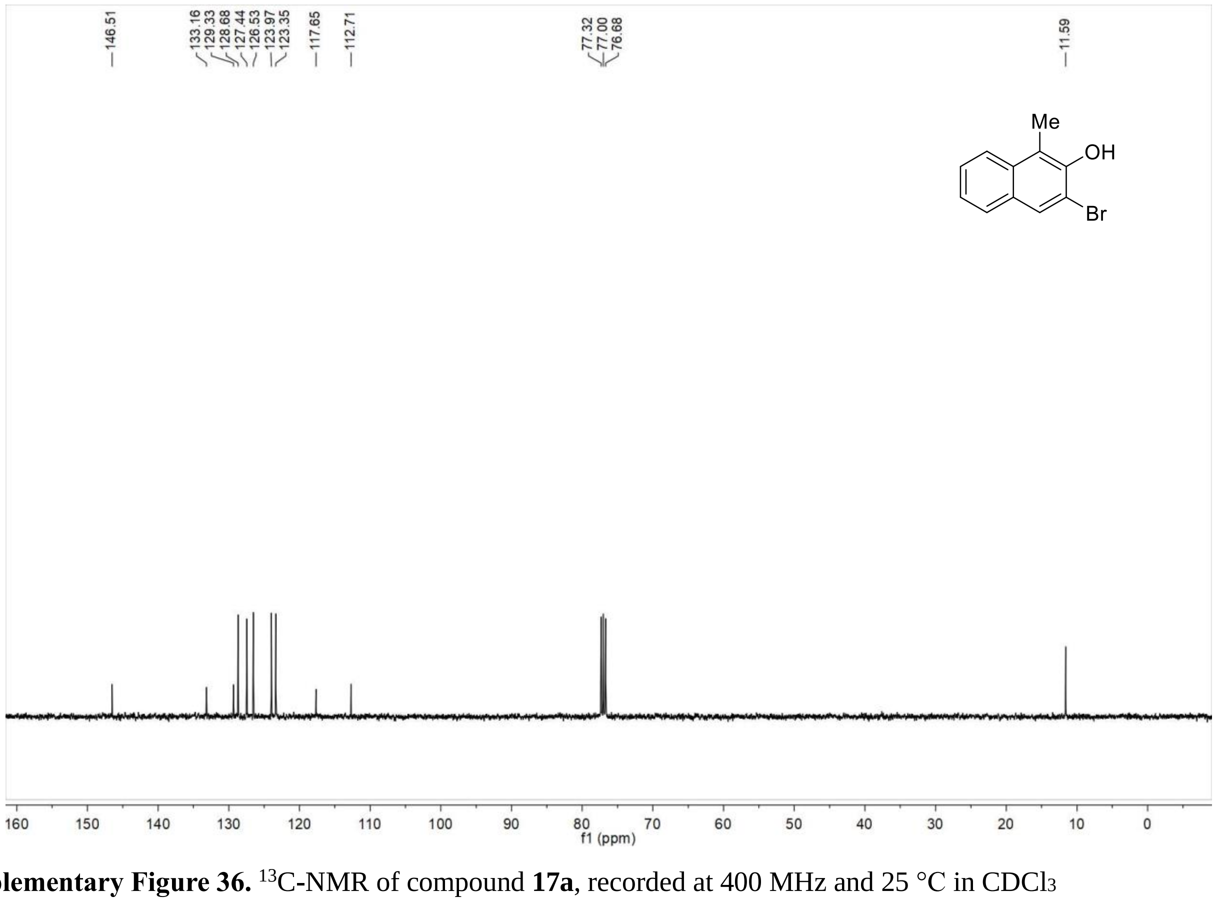

Supplementary Figure 36. ^{13}C-NMR of compound **17a**, recorded at 400 MHz and 25 °C in $CDCl_3$

3-Chloro-1-methylnaphthalen-2-ol (18a)

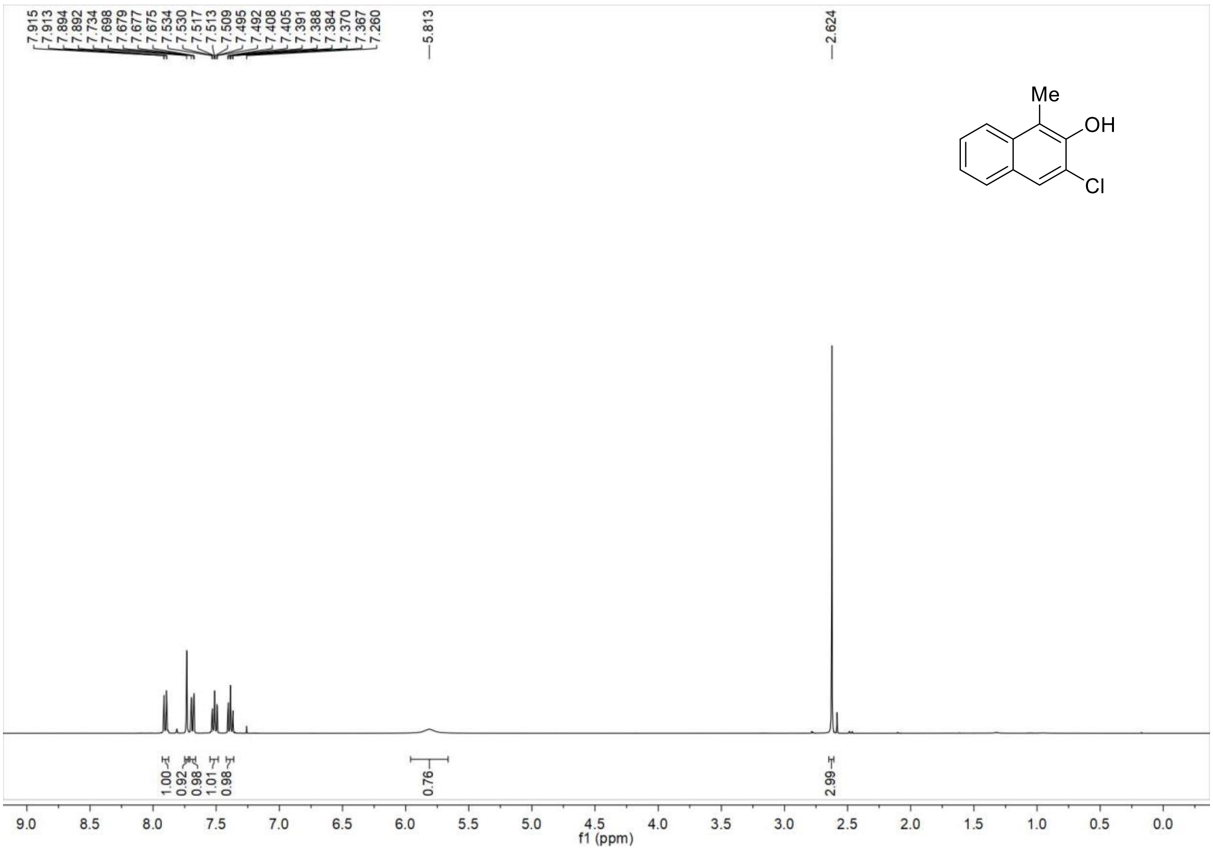

Supplementary Figure 37. ^{1}H-NMR of compound **18a**, recorded at 400 MHz and 25 °C in $CDCl_3$

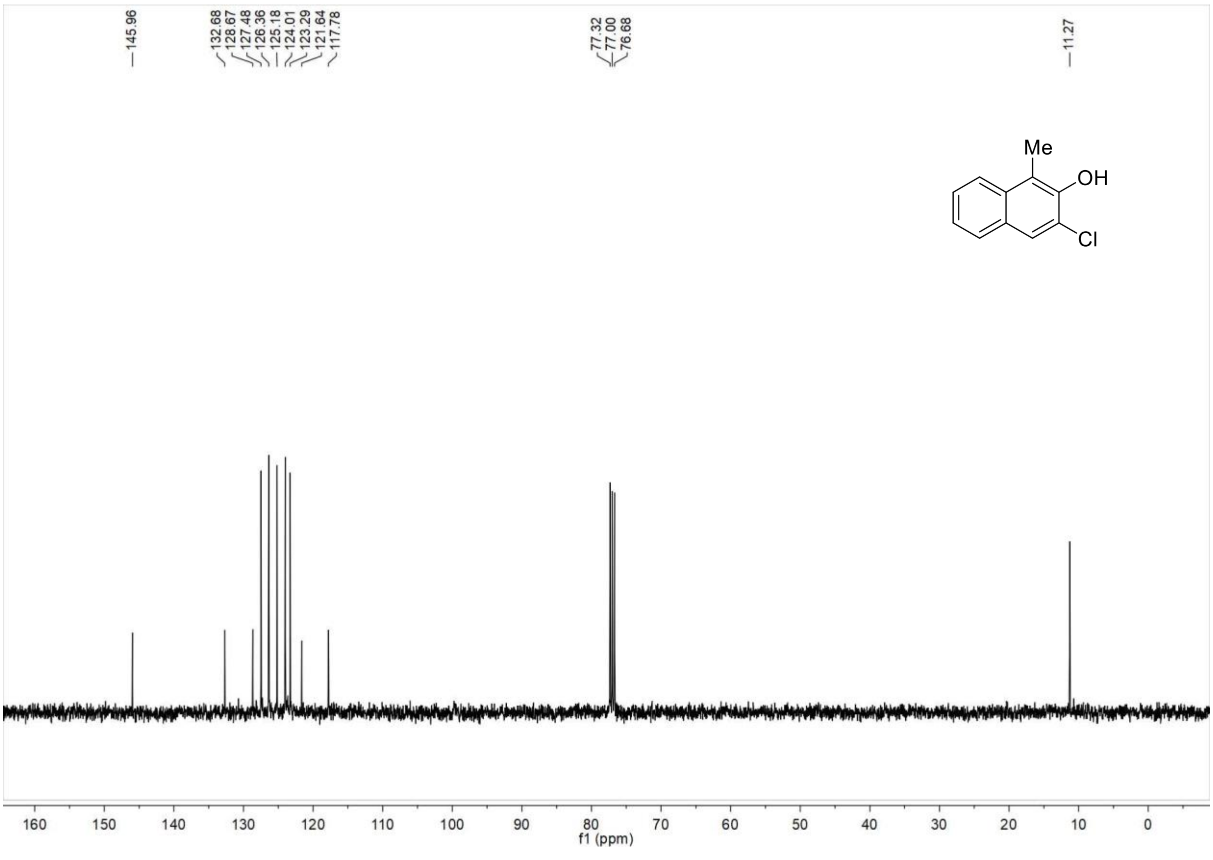

Supplementary Figure 38. ^{13}C-NMR of compound **18a**, recorded at 400 MHz and 25 °C in $CDCl_3$

1-Methyl-3-(trimethylsilyl)naphthalen-2-ol (19a)

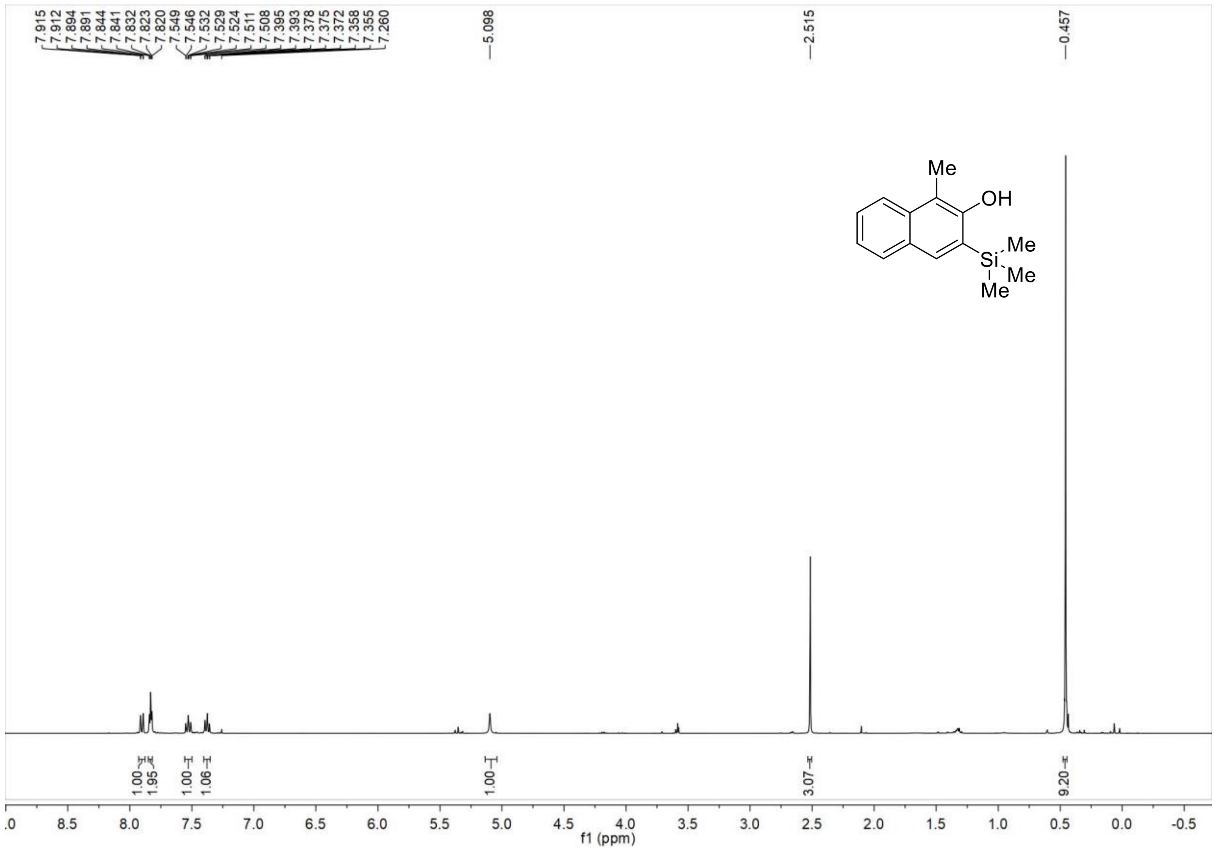

Supplementary Figure 39. ^{1}H-NMR of compound **19a**, recorded at 400 MHz and 25 °C in $CDCl_3$

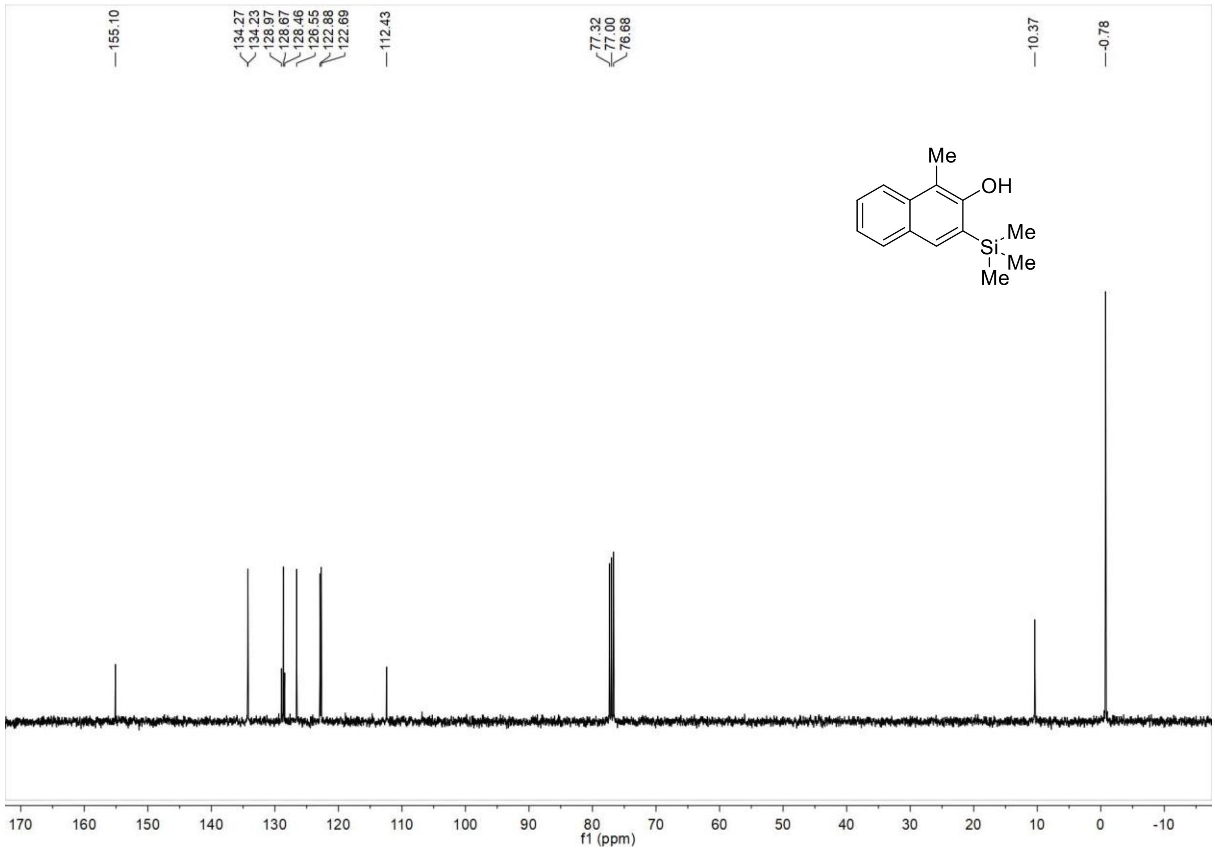

Supplementary Figure 40. ^{13}C-NMR of compound **19a**, recorded at 400 MHz and 25 °C in $CDCl_3$

3-Allyl-1-methylnaphthalen-2-ol (20a)

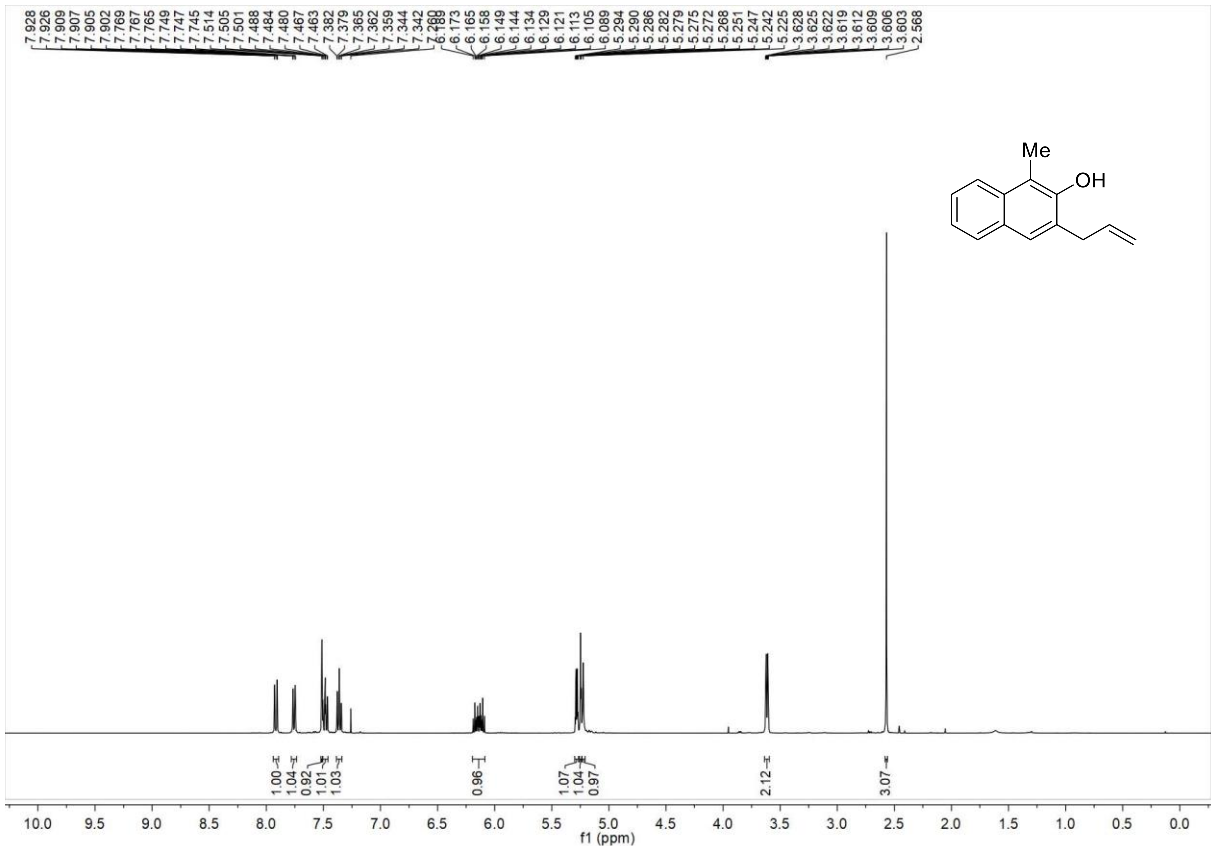

Supplementary Figure 41. ^{1}H-NMR of compound **20a**, recorded at 400 MHz and 25 °C in $CDCl_3$

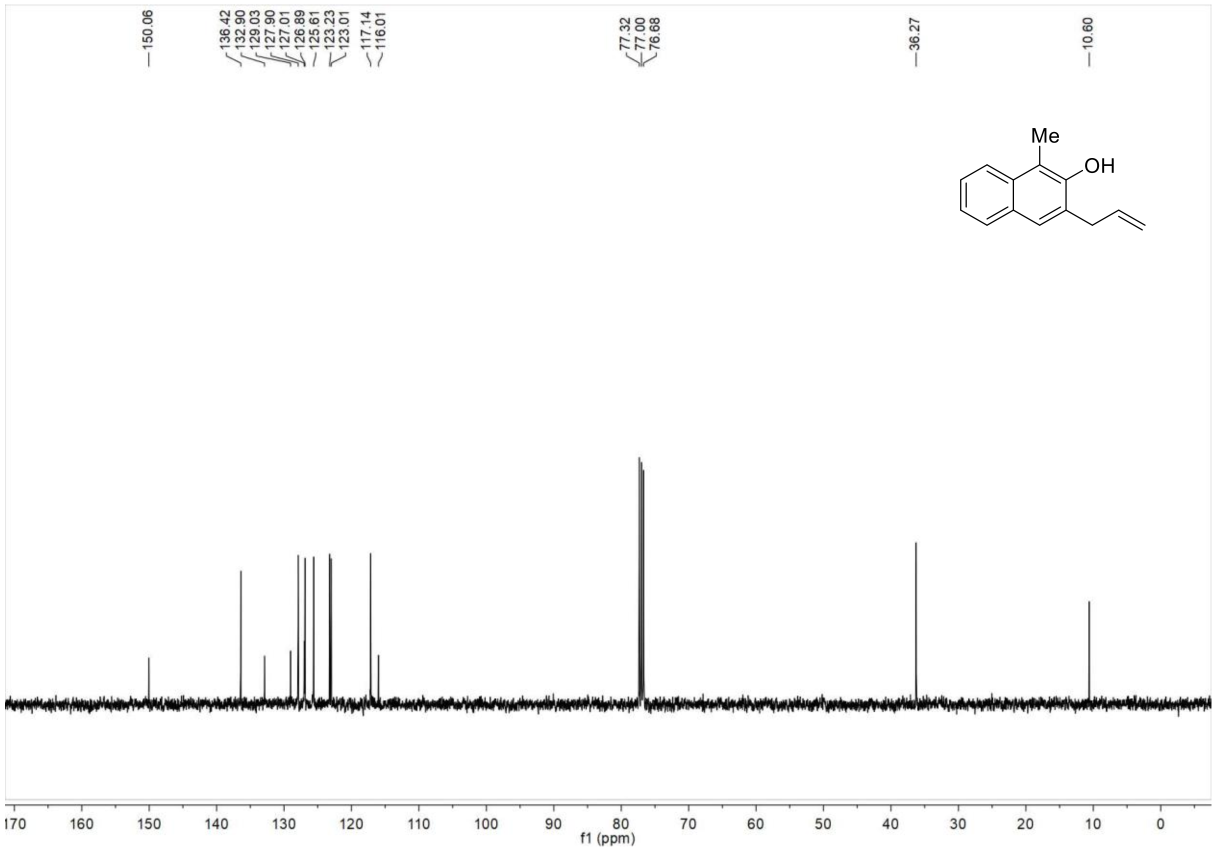

Supplementary Figure 42. ^{13}C-NMR of compound **20a**, recorded at 400 MHz and 25 °C in $CDCl_3$

1-Methyl-3-(phenylethynyl)naphthalen-2-ol (21a)

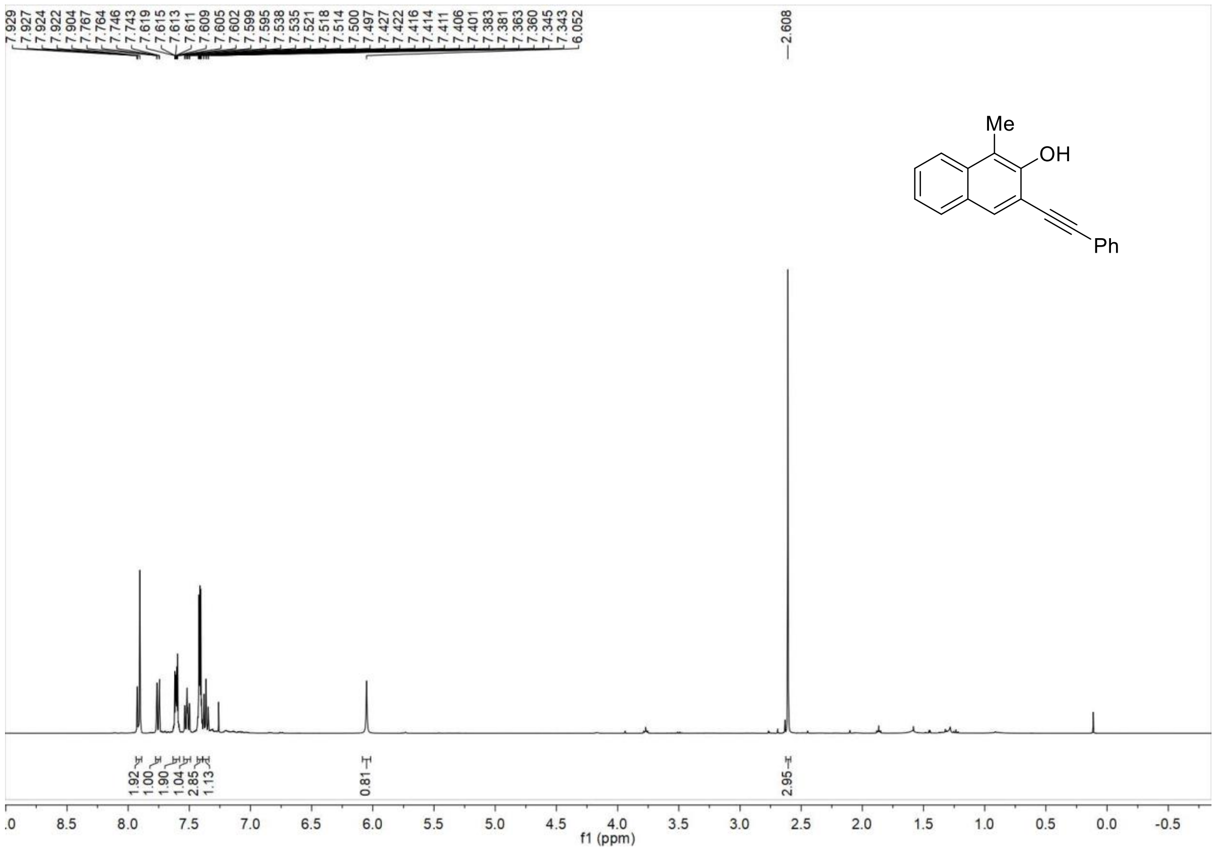

Supplementary Figure 43. ^{1}H-NMR of compound **21a**, recorded at 400 MHz and 25 °C in $CDCl_3$

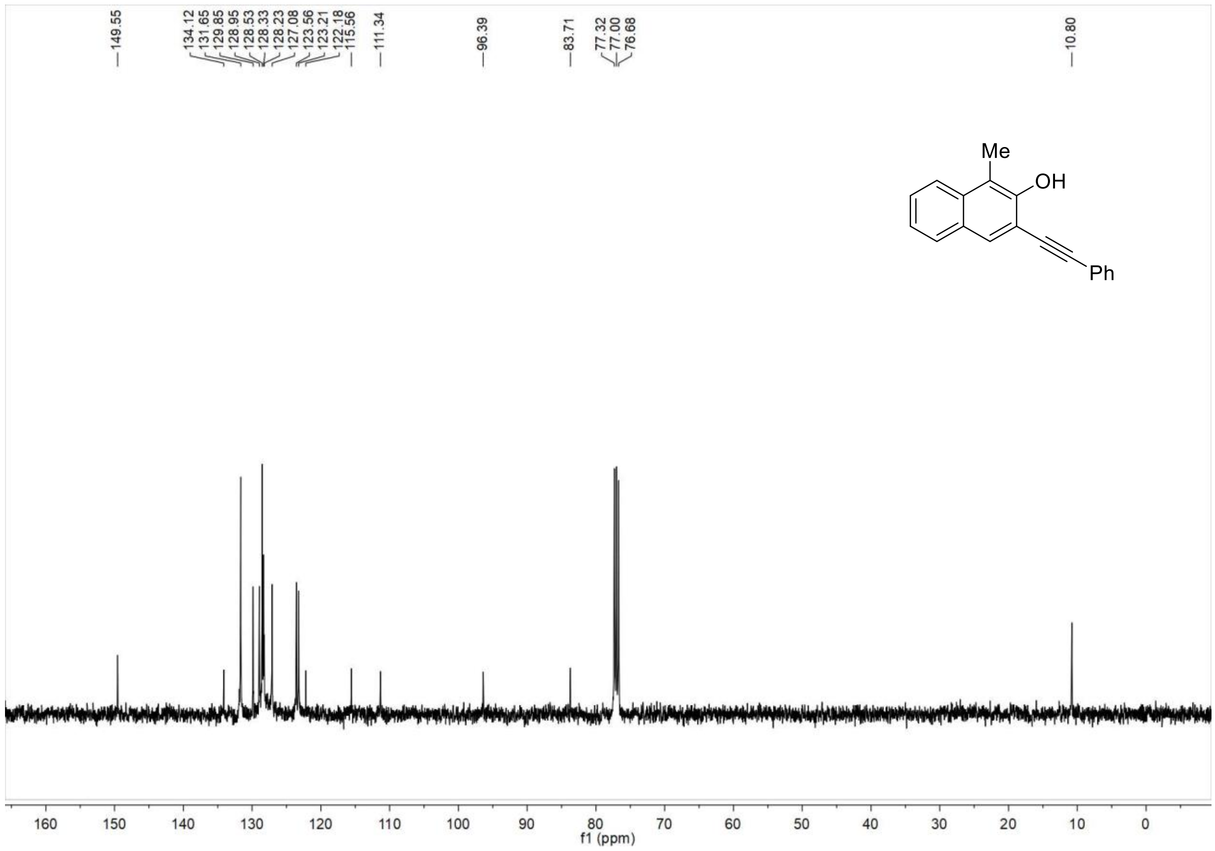

Supplementary Figure 44. ^{13}C-NMR of compound **21a**, recorded at 400 MHz and 25 °C in $CDCl_3$

5-Ethyl-2,3-dihydro-1*H*-cyclopenta[*a*]naphthalen-4-ol (22a)

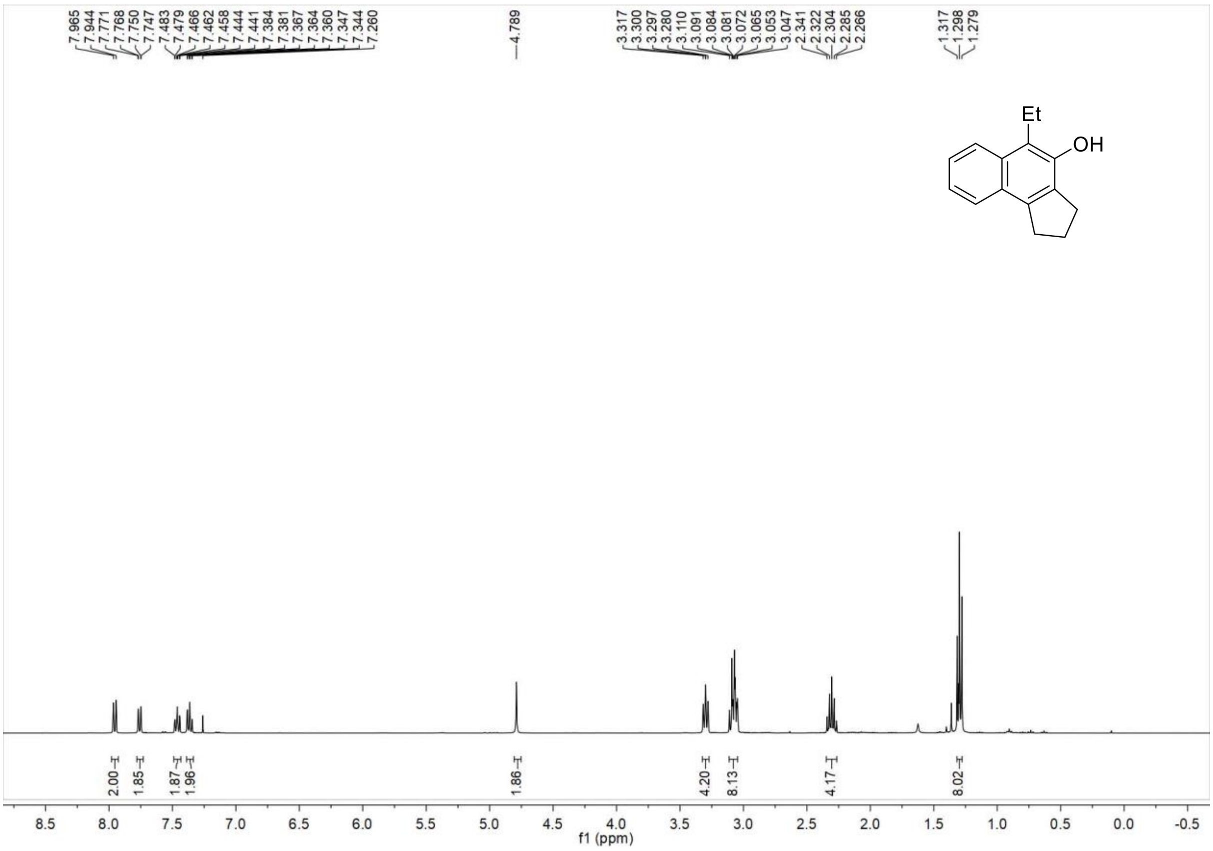

Supplementary Figure 45. ^{1}H-NMR of compound **22a**, recorded at 400 MHz and 25 °C in $CDCl_3$

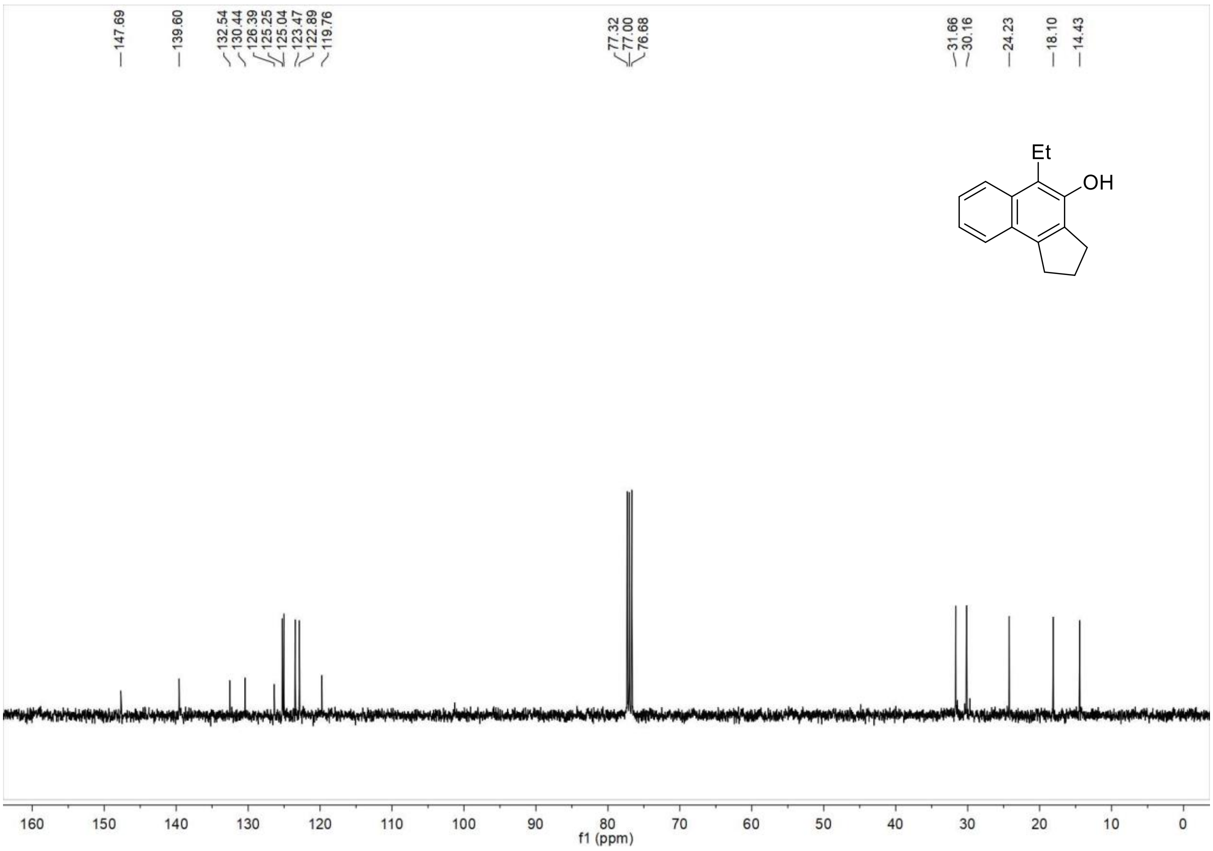

Supplementary Figure 46. ^{13}C-NMR of compound **22a**, recorded at 400 MHz and 25 °C in $CDCl_3$

10-Methyl-5,6,7,8-tetrahydrophenanthren-9-ol (23a)

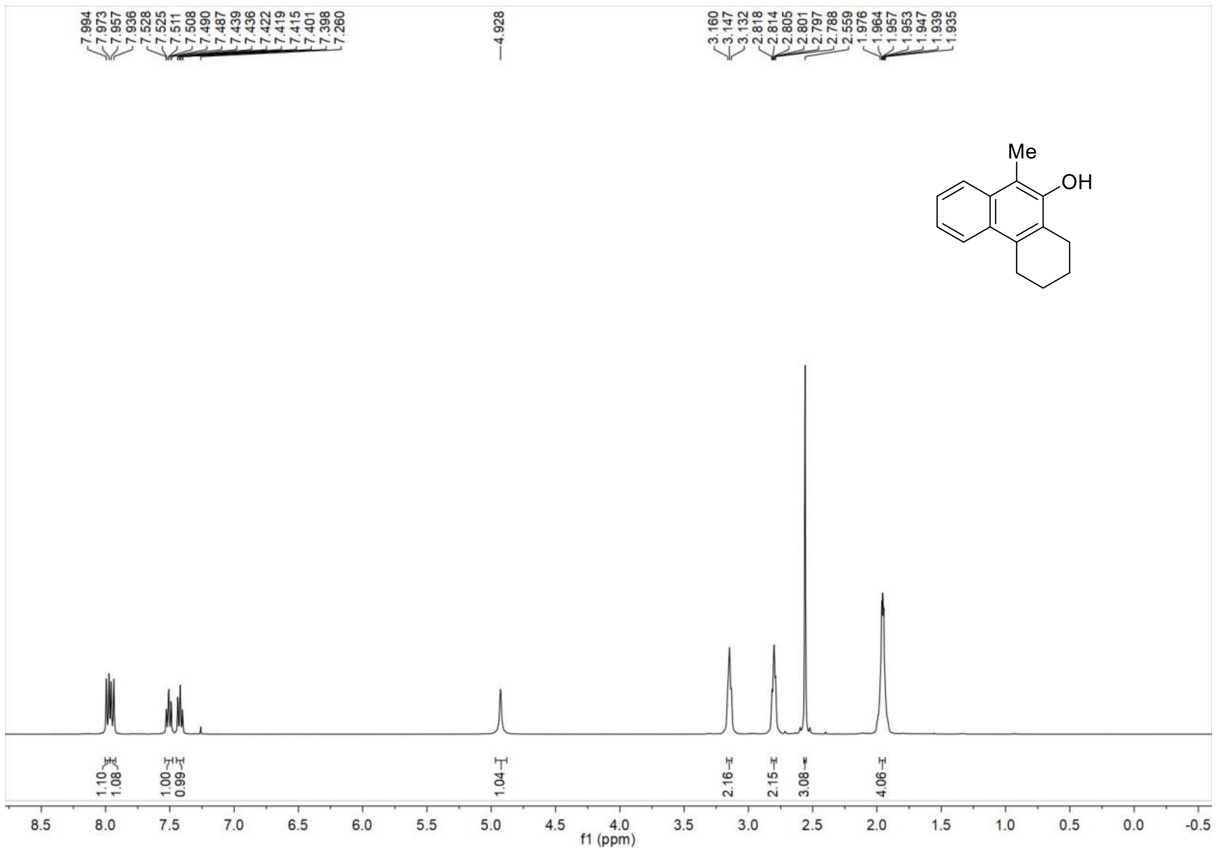

Supplementary Figure 47. ^{1}H-NMR of compound **23a**, recorded at 400 MHz and 25 °C in $CDCl_3$

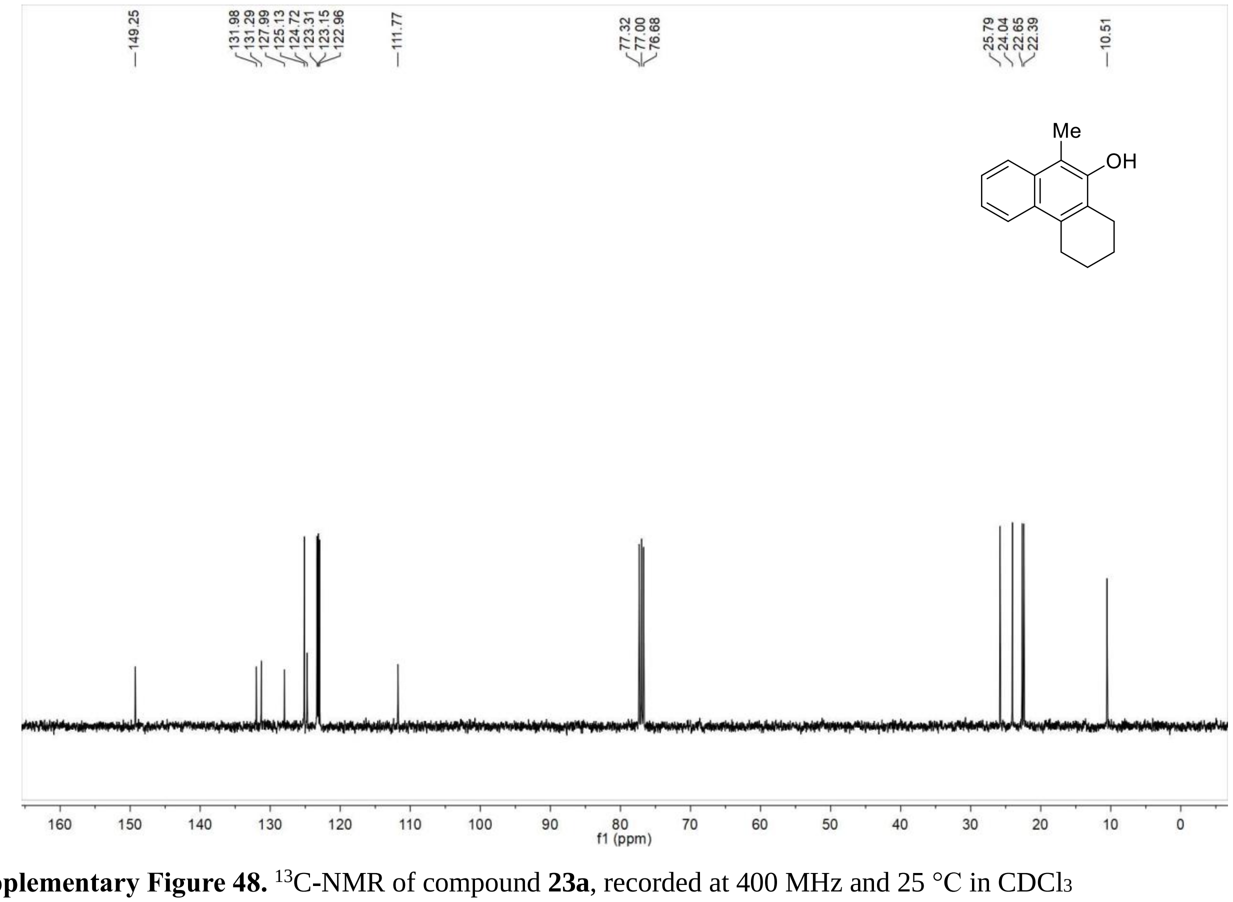

Supplementary Figure 48. ^{13}C-NMR of compound **23a**, recorded at 400 MHz and 25 °C in $CDCl_3$

5-Methyl-8,9,10,11-tetrahydro-7*H*-cyclohepta[*a*]naphthalen-6-ol (24a)

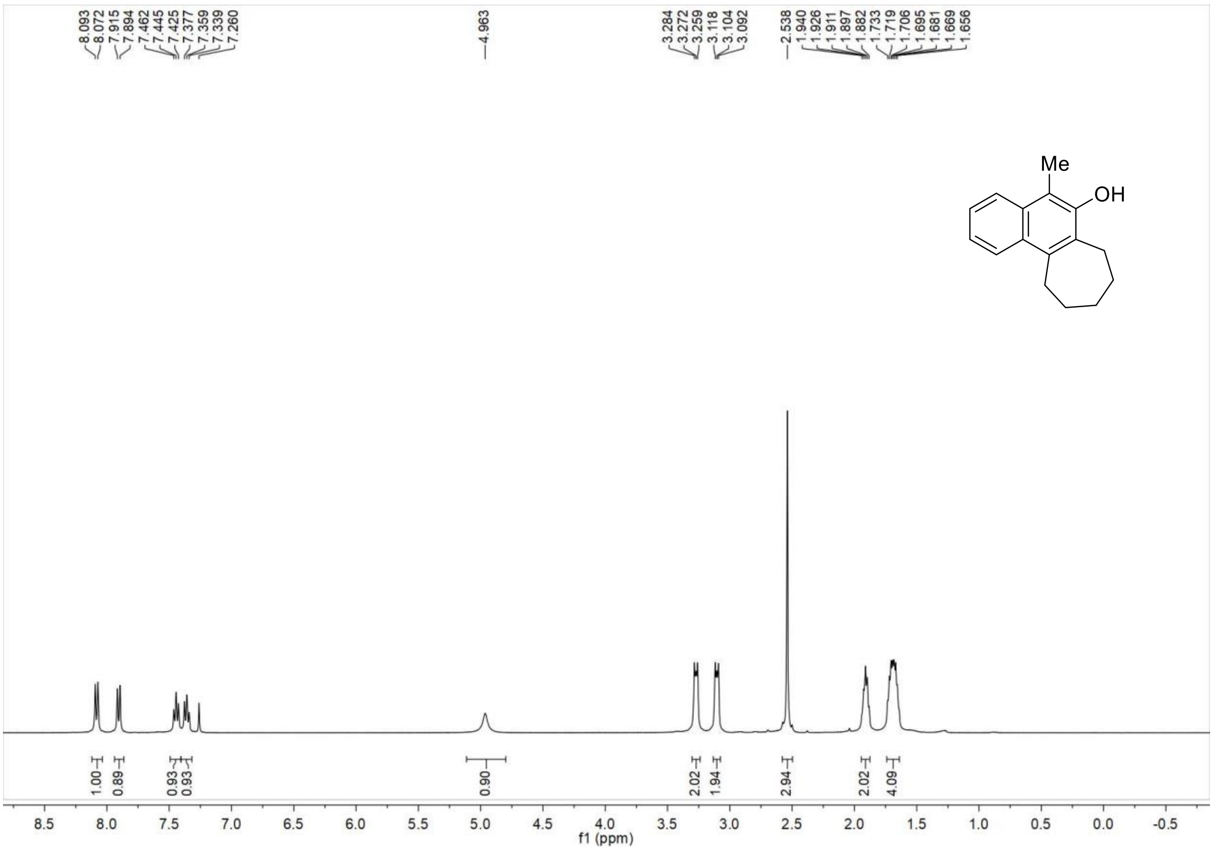

Supplementary Figure 49. ^{1}H-NMR of compound **24a**, recorded at 400 MHz and 25 °C in $CDCl_3$

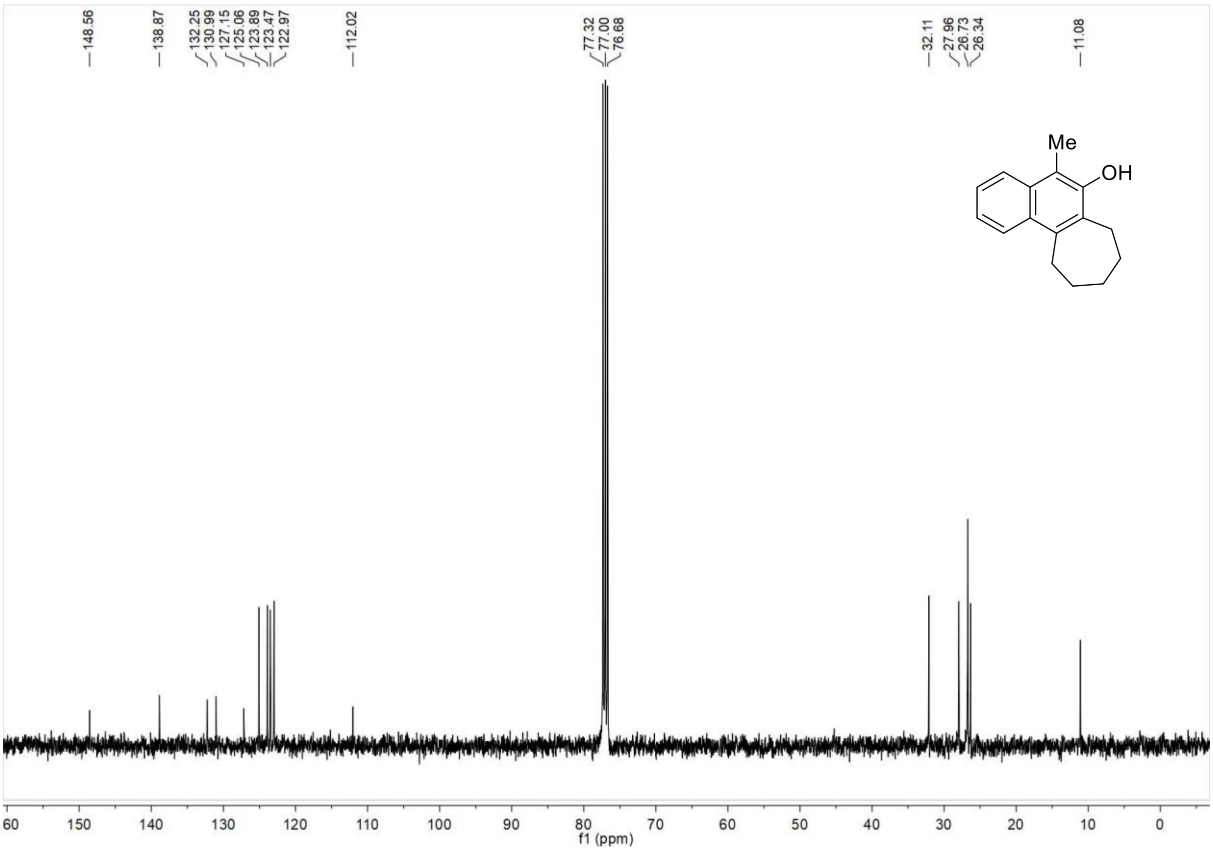

Supplementary Figure 50. ^{13}C-NMR of compound **24a**, recorded at 400 MHz and 25 °C in $CDCl_3$

4,9,11,11-Tetramethyl-1,2,3,4-tetrahydro-1,4-methanophenanthren-10-ol (25a)

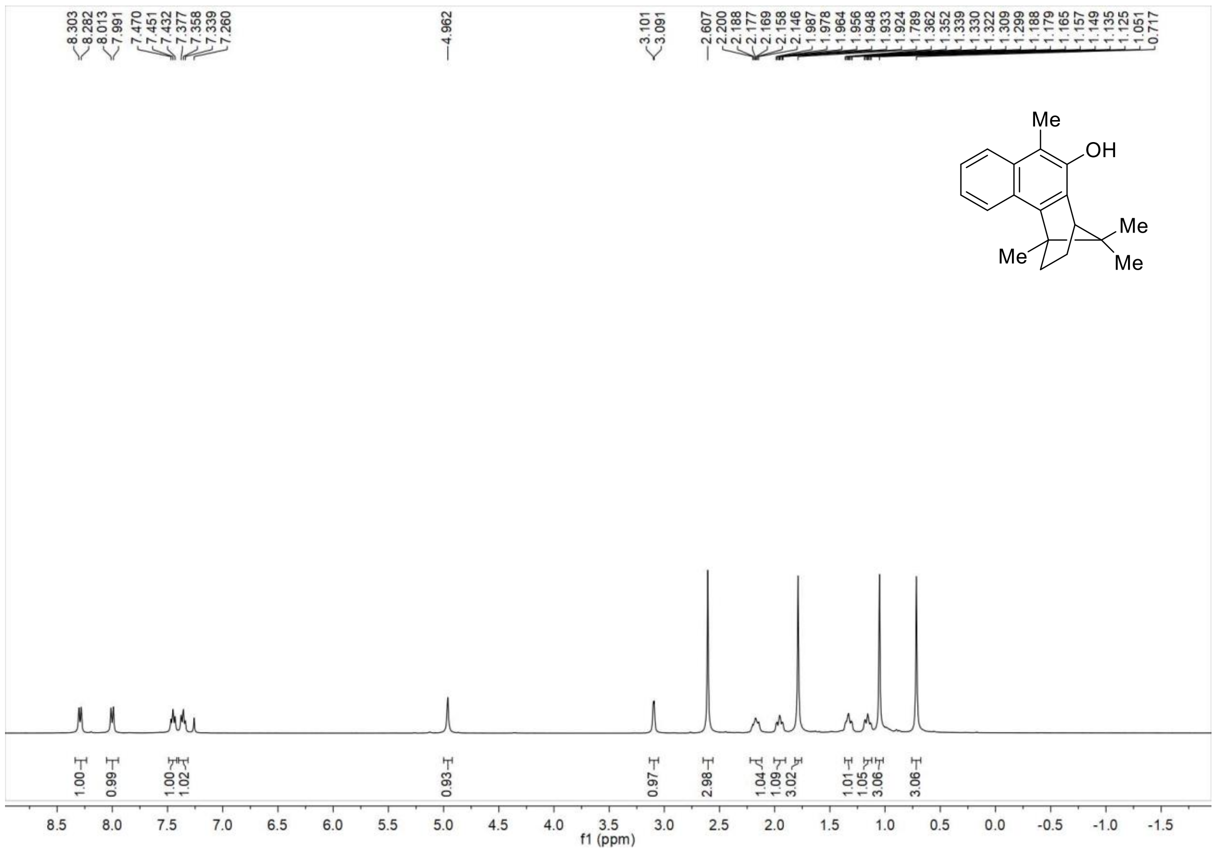

Supplementary Figure 51. ^{1}H-NMR of compound **25a**, recorded at 400 MHz and 25 °C in CDCl3

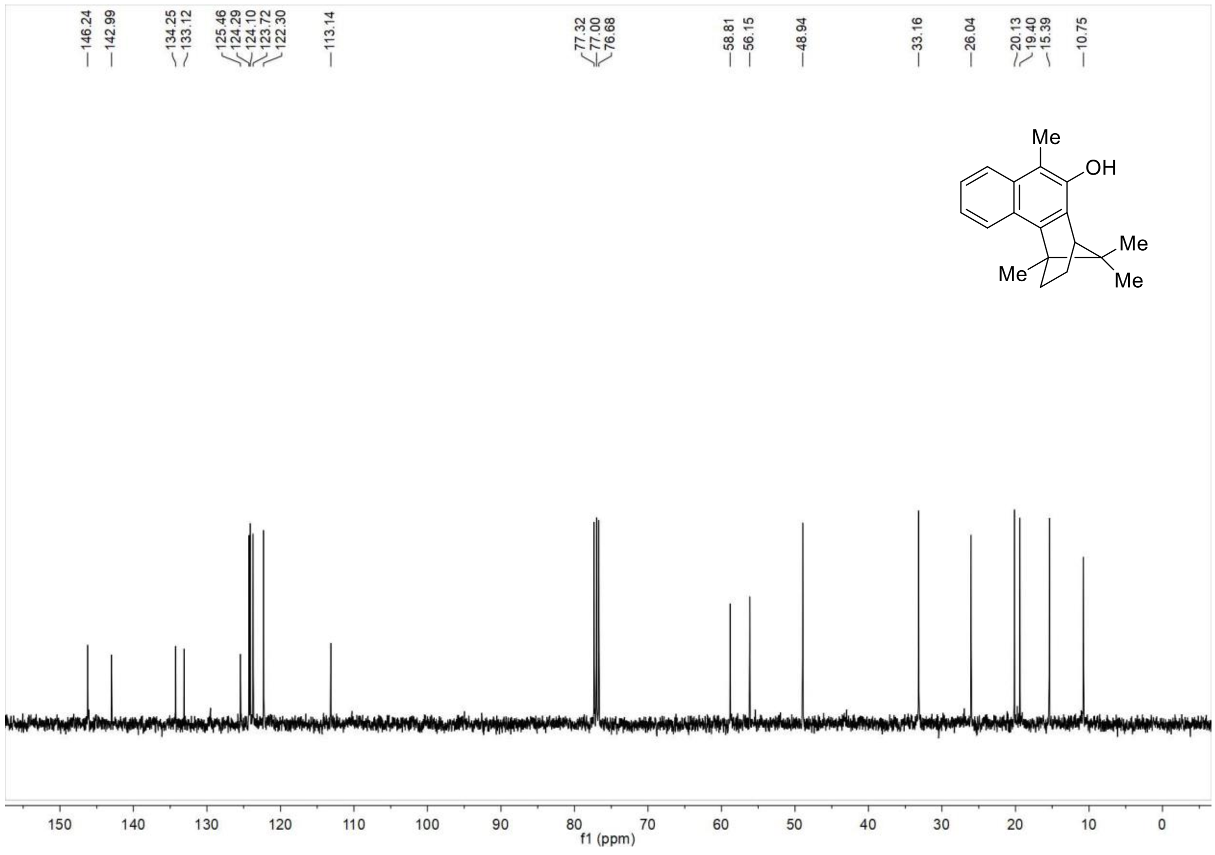

Supplementary Figure 52. ^{13}C-NMR of compound **25a**, recorded at 400 MHz and 25 °C in CDCl3

3-(benzofuran-2-yl)-1-Methylnaphthalen-2-ol (26a)

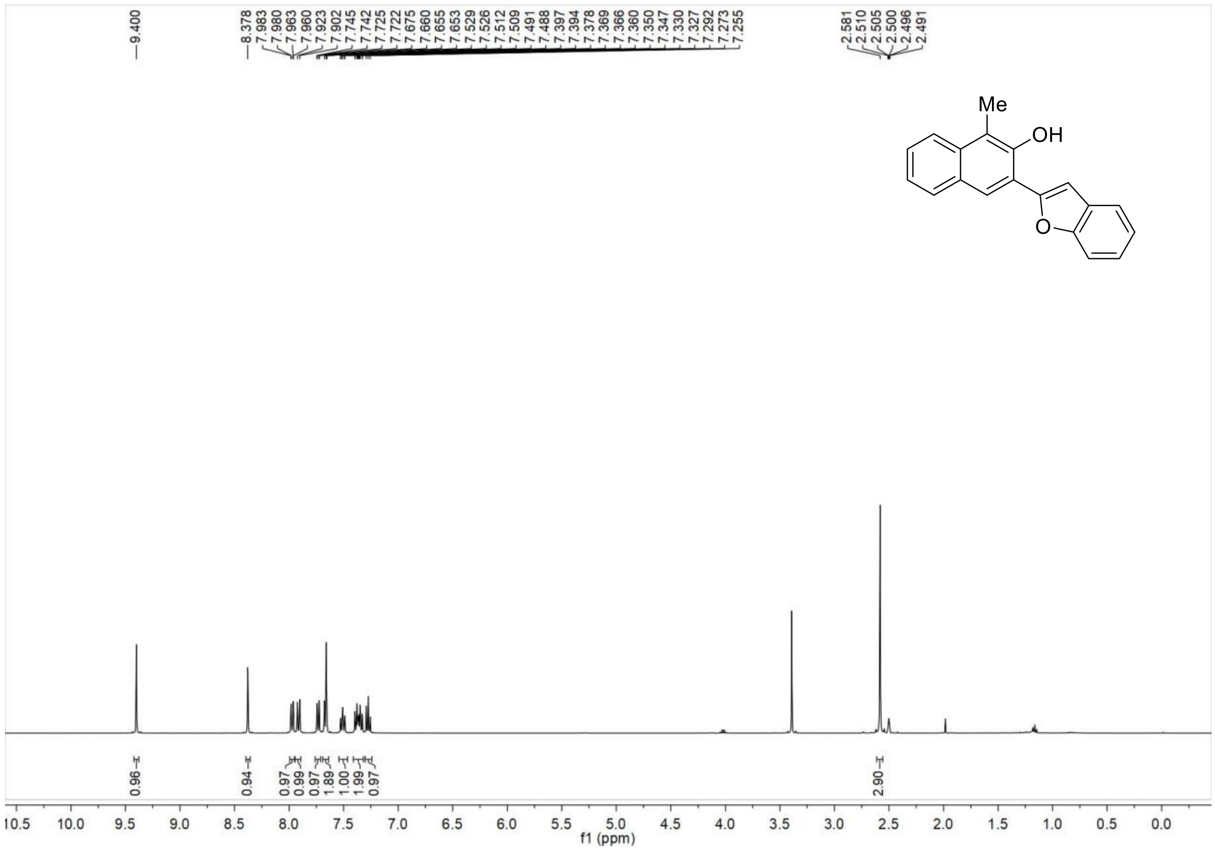

Supplementary Figure 53. ^{1}H-NMR of compound **26a**, recorded at 400 MHz and 25 °C in DMSO-d_6

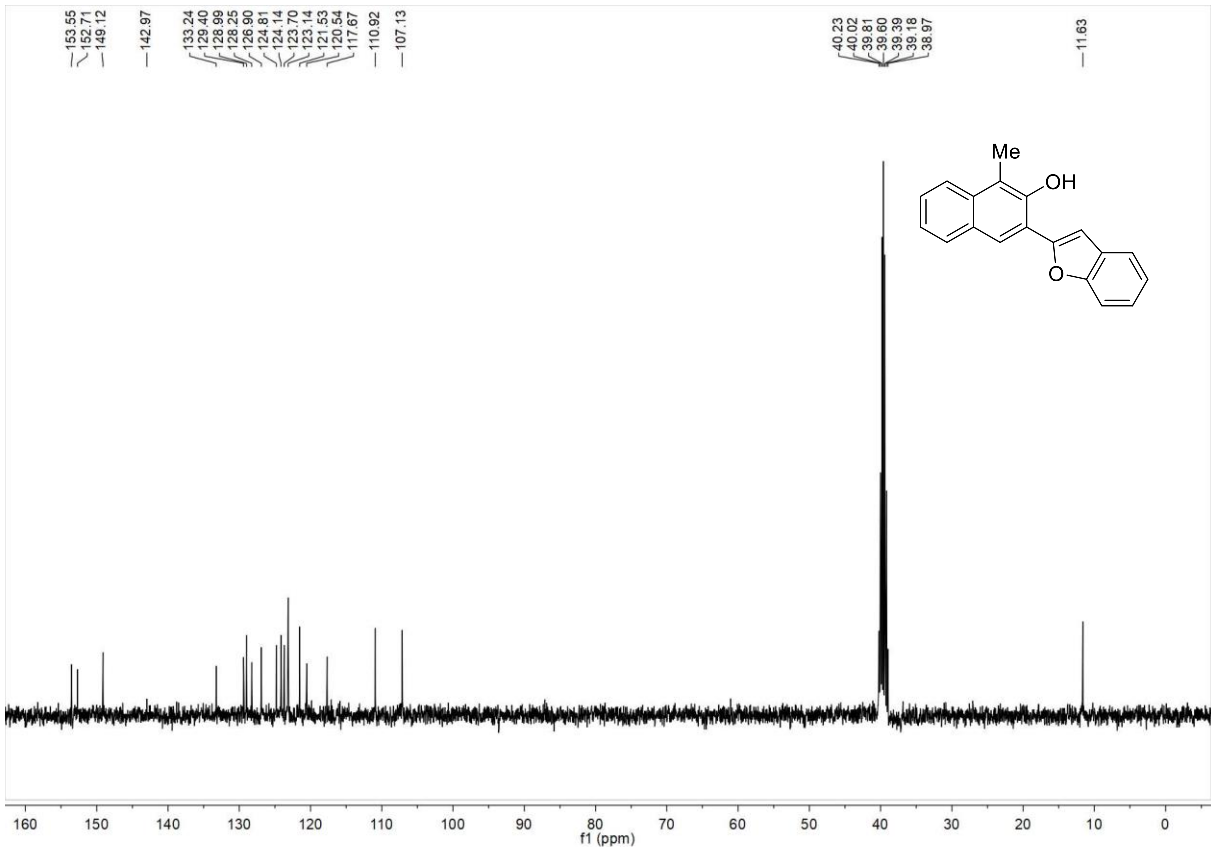

Supplementary Figure 54. ^{13}C-NMR of compound **26a**, recorded at 400 MHz and 25 °C in DMSO-d_6

6-(furan-3-yl)-1-Methylnaphthalen-2-ol (27a)

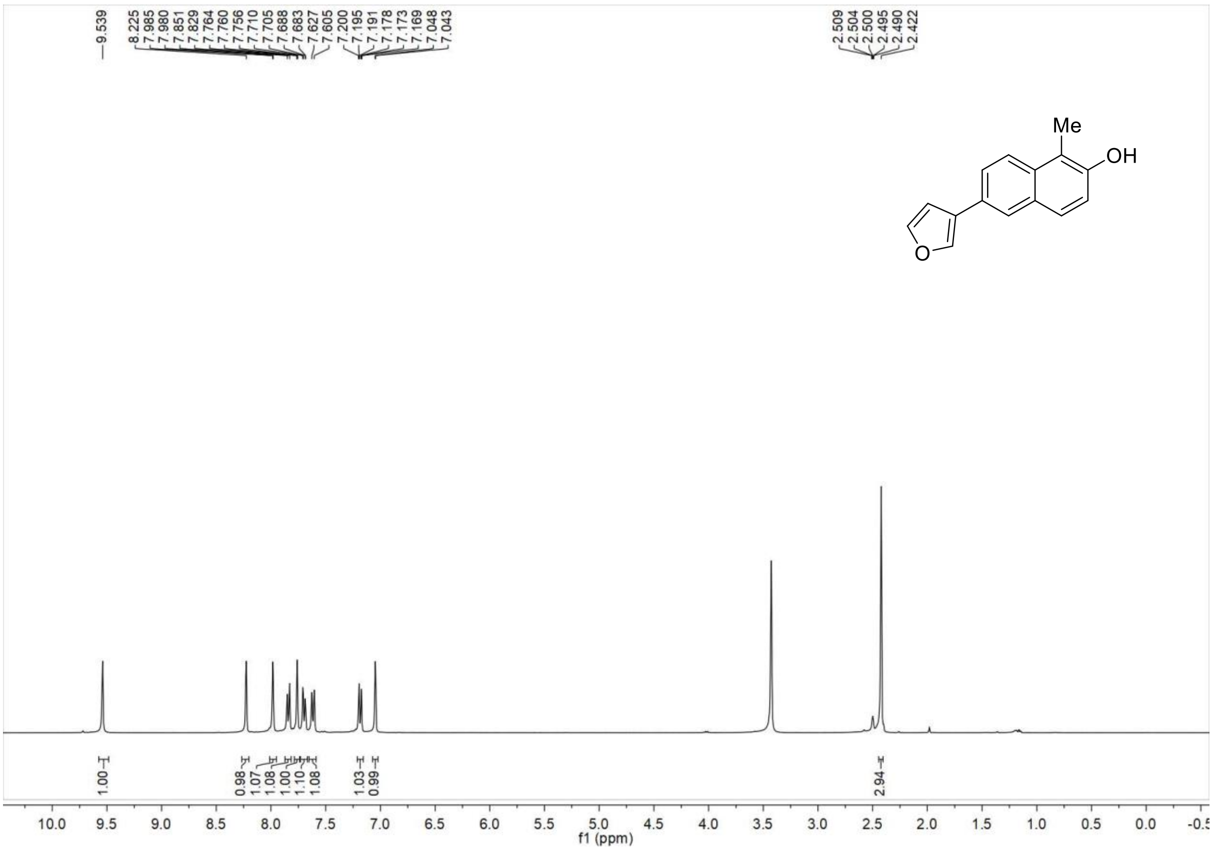

Supplementary Figure 55. ^{1}H-NMR of compound **27a**, recorded at 400 MHz and 25 °C in $CDCl_3$

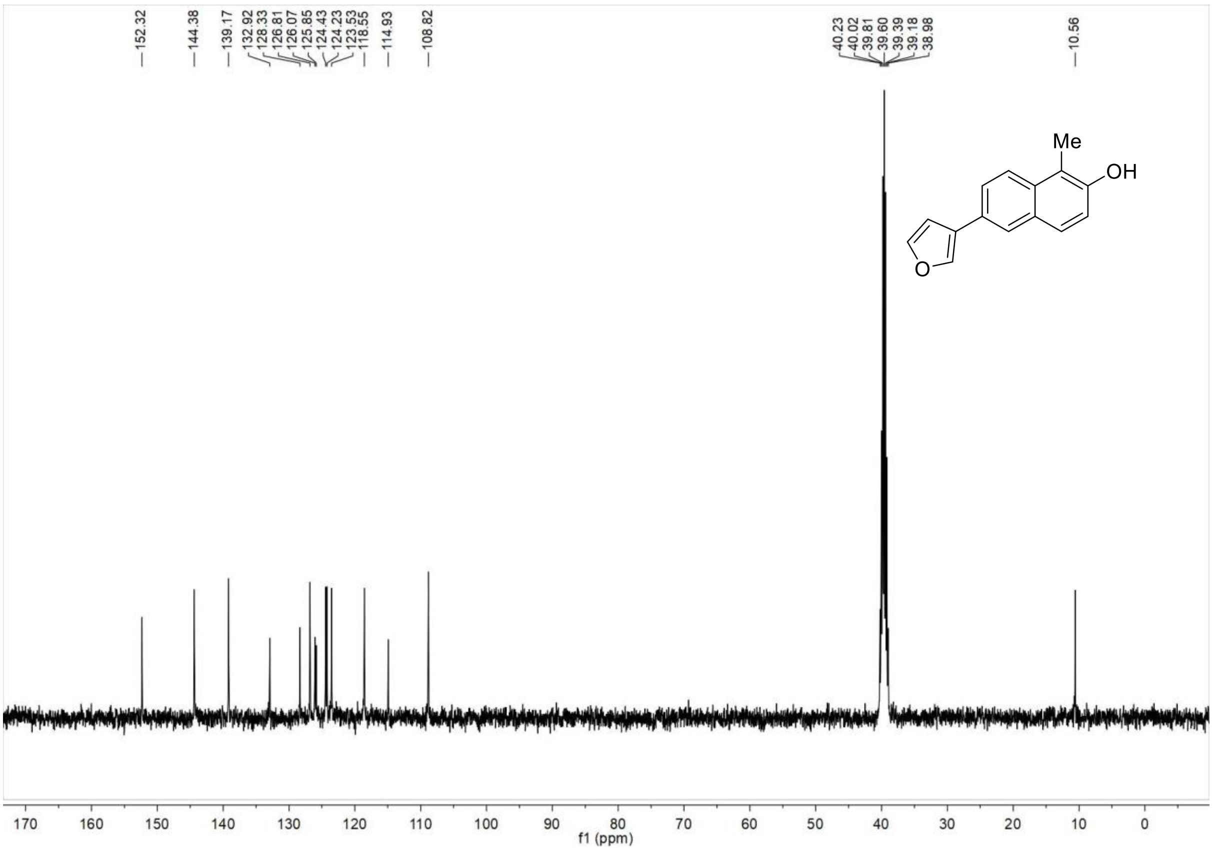

Supplementary Figure 56. ^{13}C-NMR of compound **27a**, recorded at 400 MHz and 25 °C in DMSO-d_6

3-(dibenzo[*b*,*d*]furan-3-yl)-1-Methylnaphthalen-2-ol (**28a**)

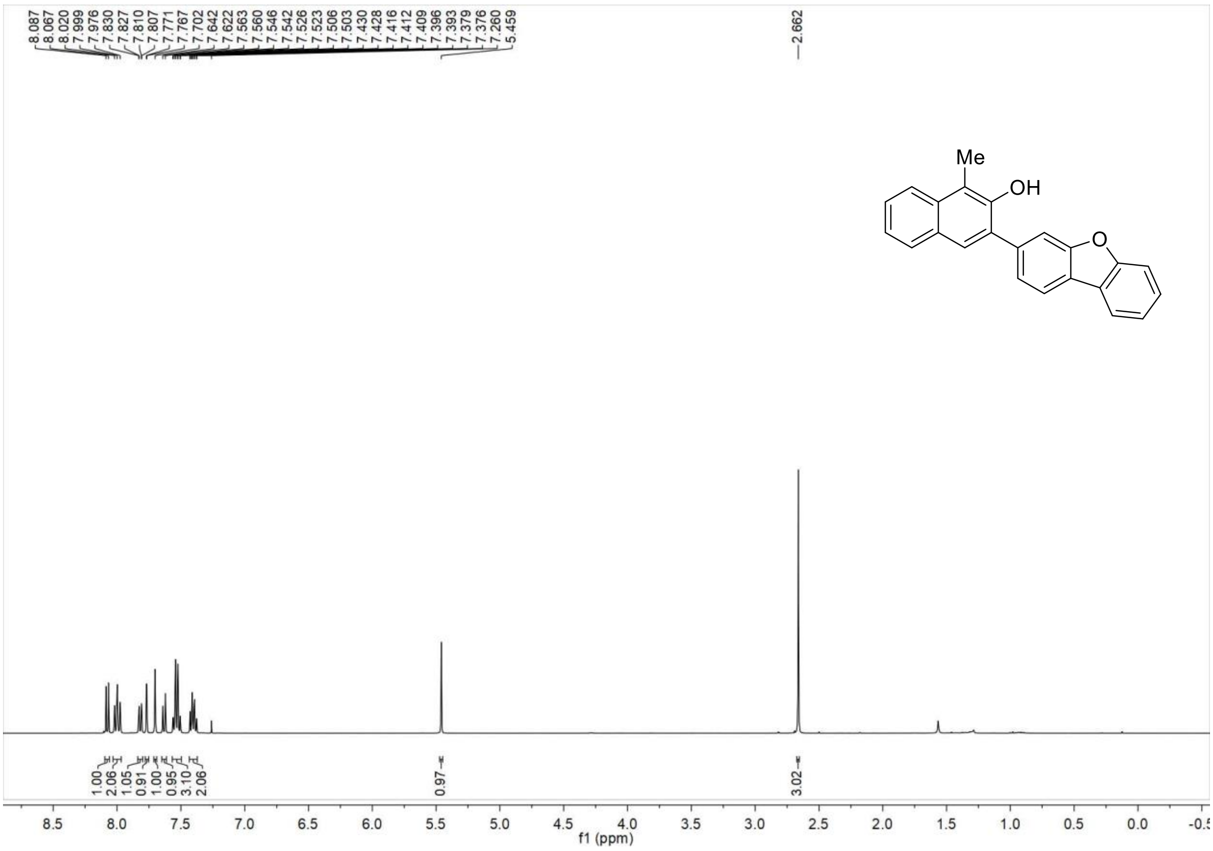

Supplementary Figure 57. ^{1}H-NMR of compound **28a**, recorded at 400 MHz and 25 °C in $CDCl_3$

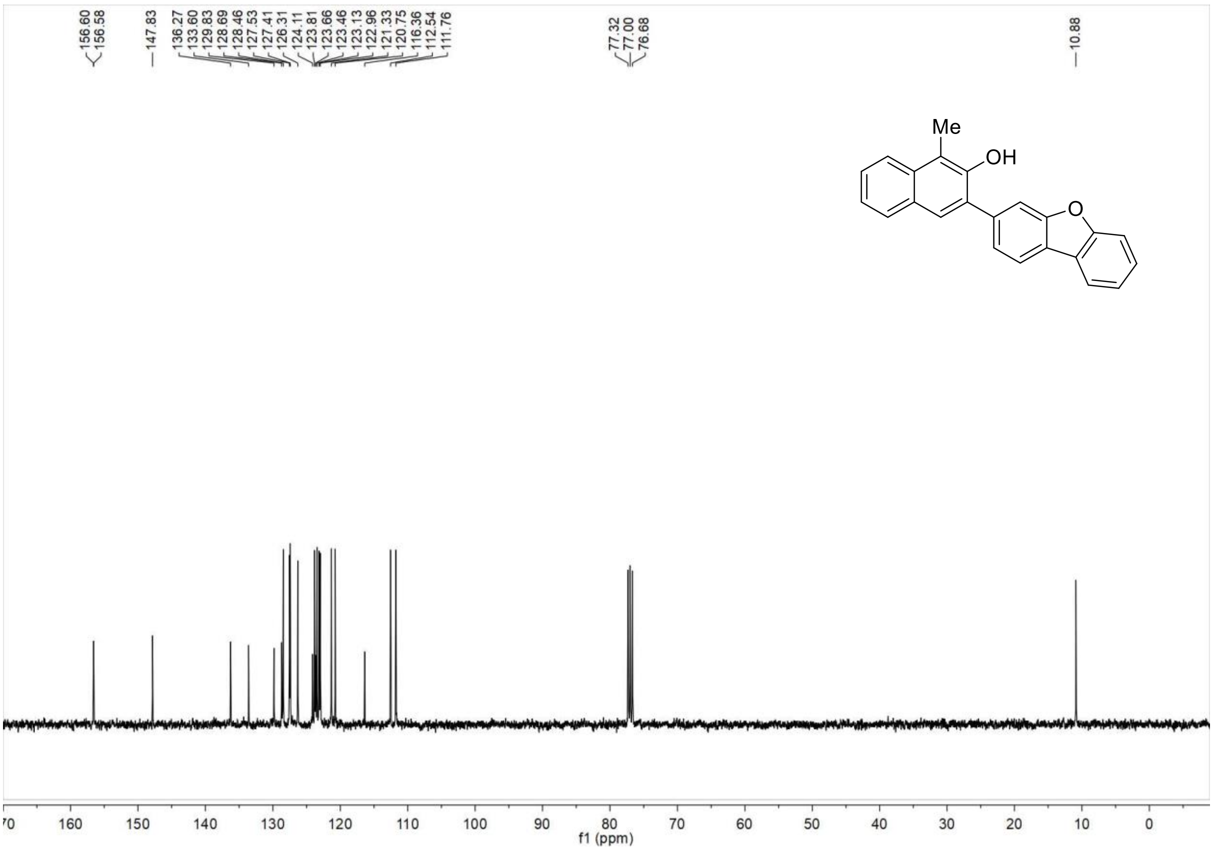

Supplementary Figure 58. ^{13}C-NMR of compound **28a**, recorded at 400 MHz and 25 °C in $CDCl_3$

1-Methyl-6-(quinolin-3-yl)naphthalen-2-ol (**29a**)

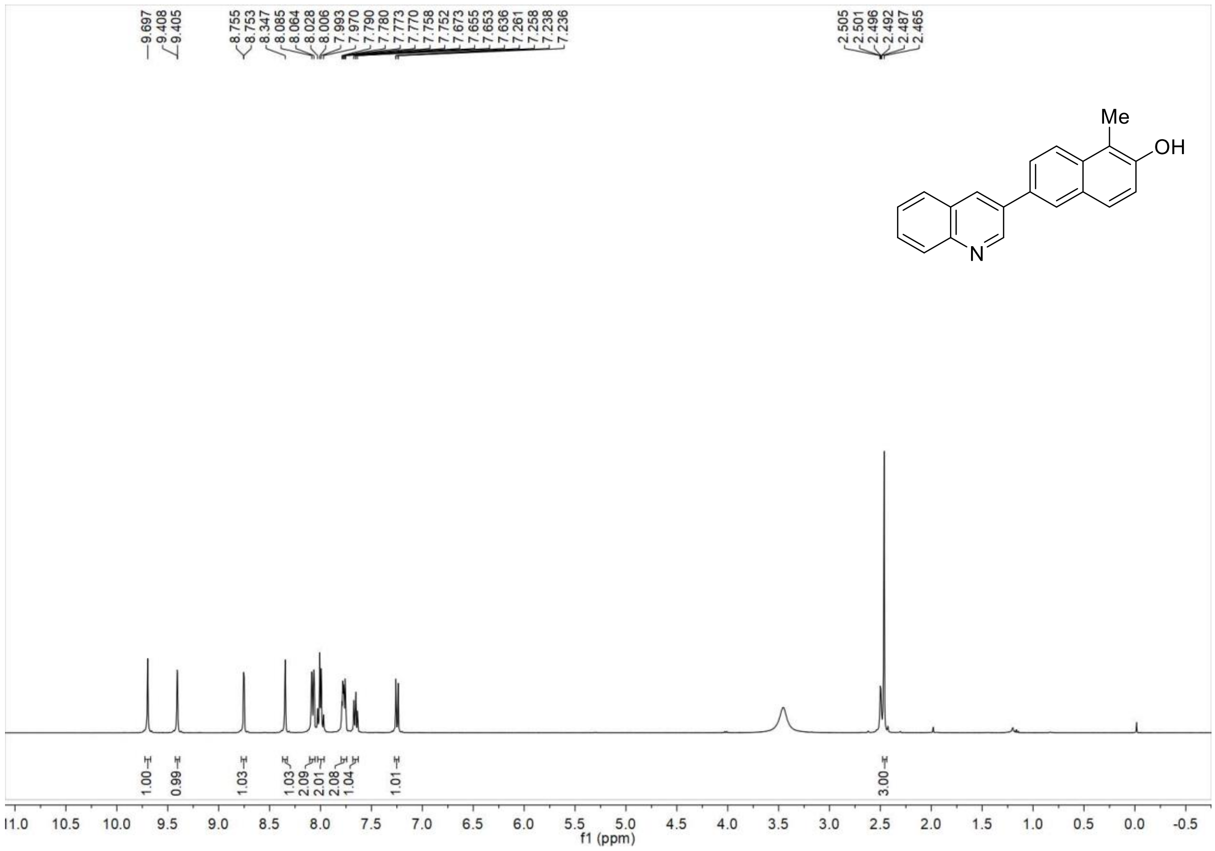

Supplementary Figure 59. ^{1}H-NMR of compound **29a**, recorded at 400 MHz and 25 °C in DMSO-d_6

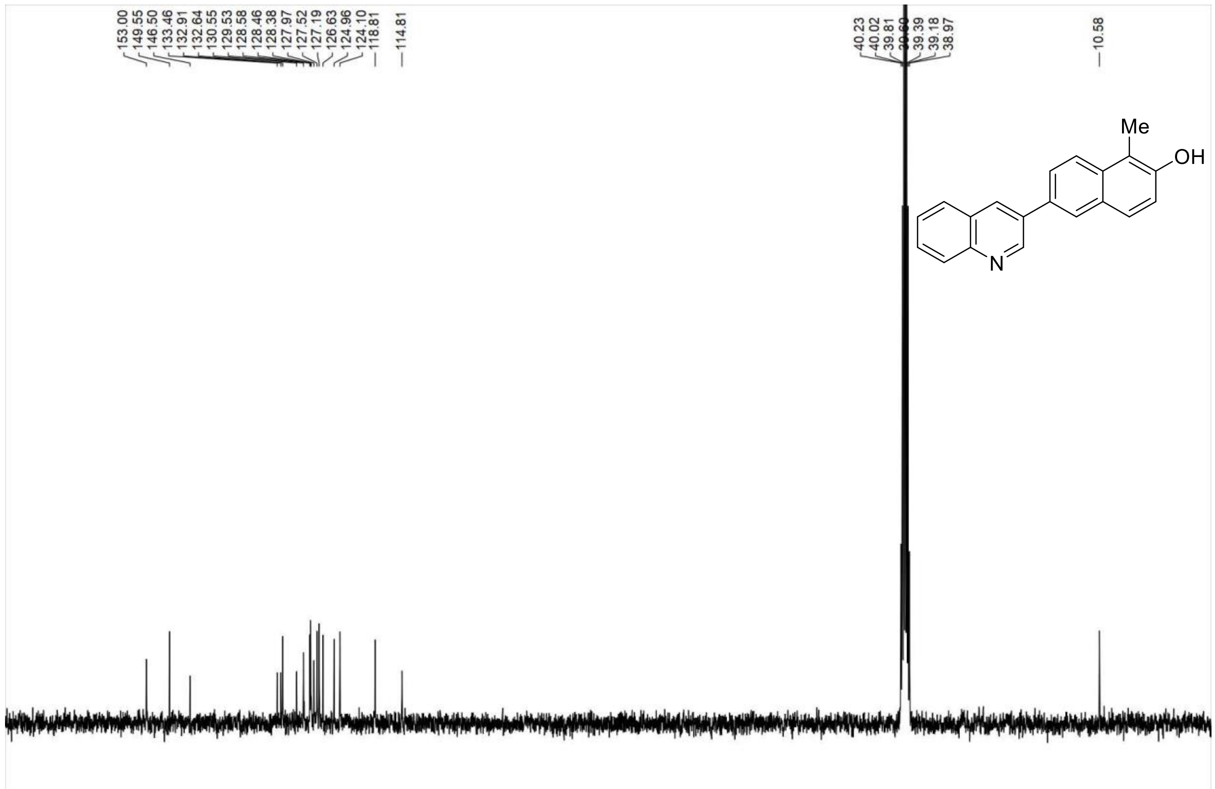

Supplementary Figure 60. ^{13}C-NMR of compound **29a**, recorded at 400 MHz and 25 °C in DMSO-d_6

1-Methyl-3-(phenoxathiin-4-yl)naphthalen-2-ol (**30a**)

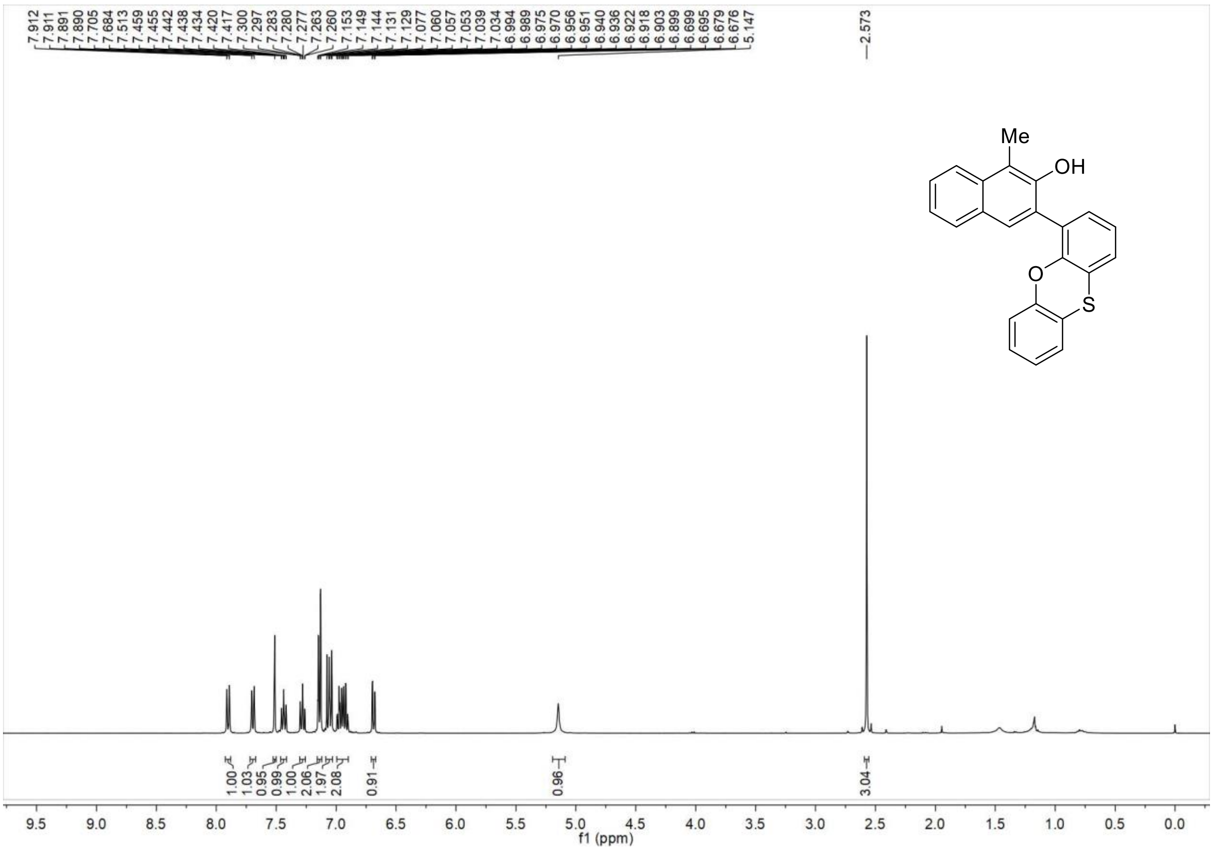

Supplementary Figure 61. ^{1}H-NMR of compound **30a**, recorded at 400 MHz and 25 °C in $CDCl_3$

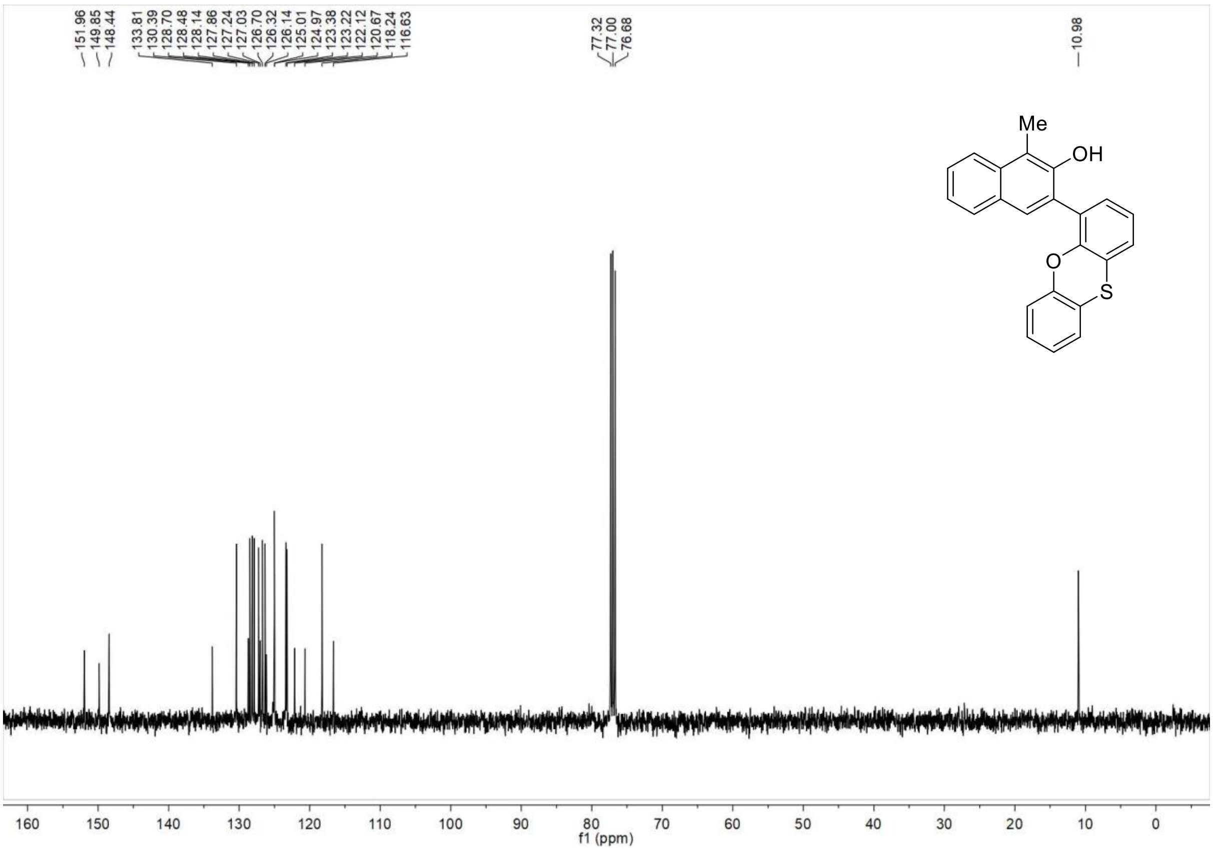

Supplementary Figure 62. ^{13}C-NMR of compound **30a**, recorded at 400 MHz and 25 °C in $CDCl_3$

5-Methylnaphtho[1,2-*b*]thiophen-4-ol (31a)

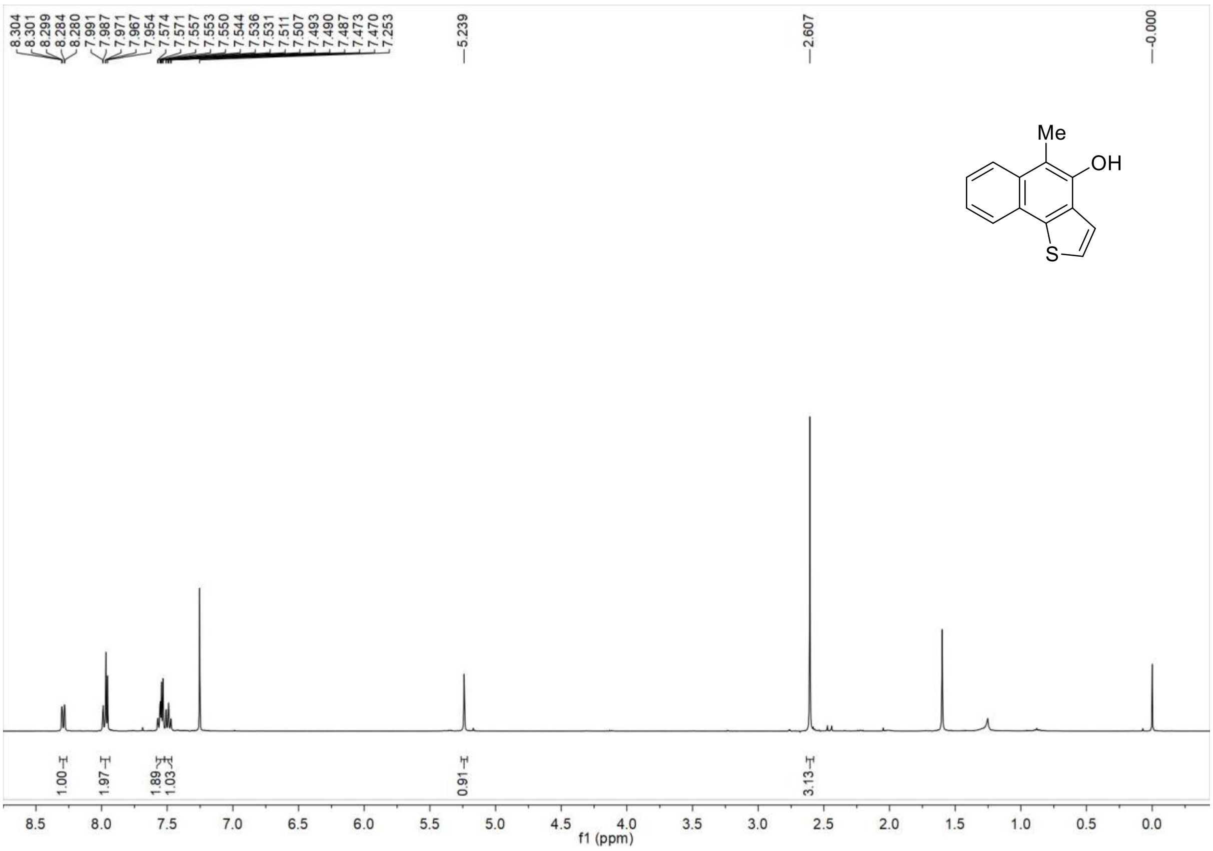

Supplementary Figure 63. ^{1}H-NMR of compound **31a**, recorded at 400 MHz and 25 °C in CDCl3

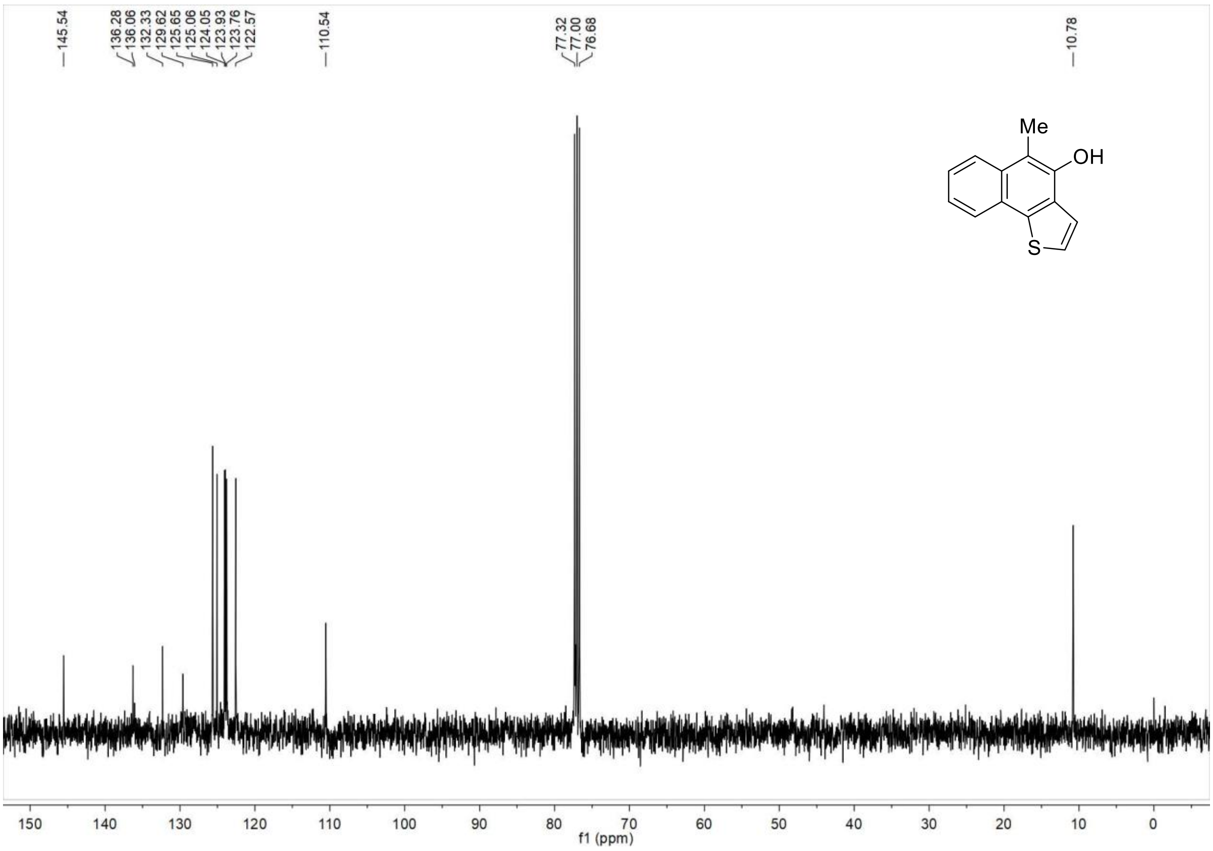

Supplementary Figure 64. ^{13}C-NMR of compound **31a**, recorded at 400 MHz and 25 °C in CDCl3

5-Methylnaphtho[1,2-*b*]furan-4-ol (32a)

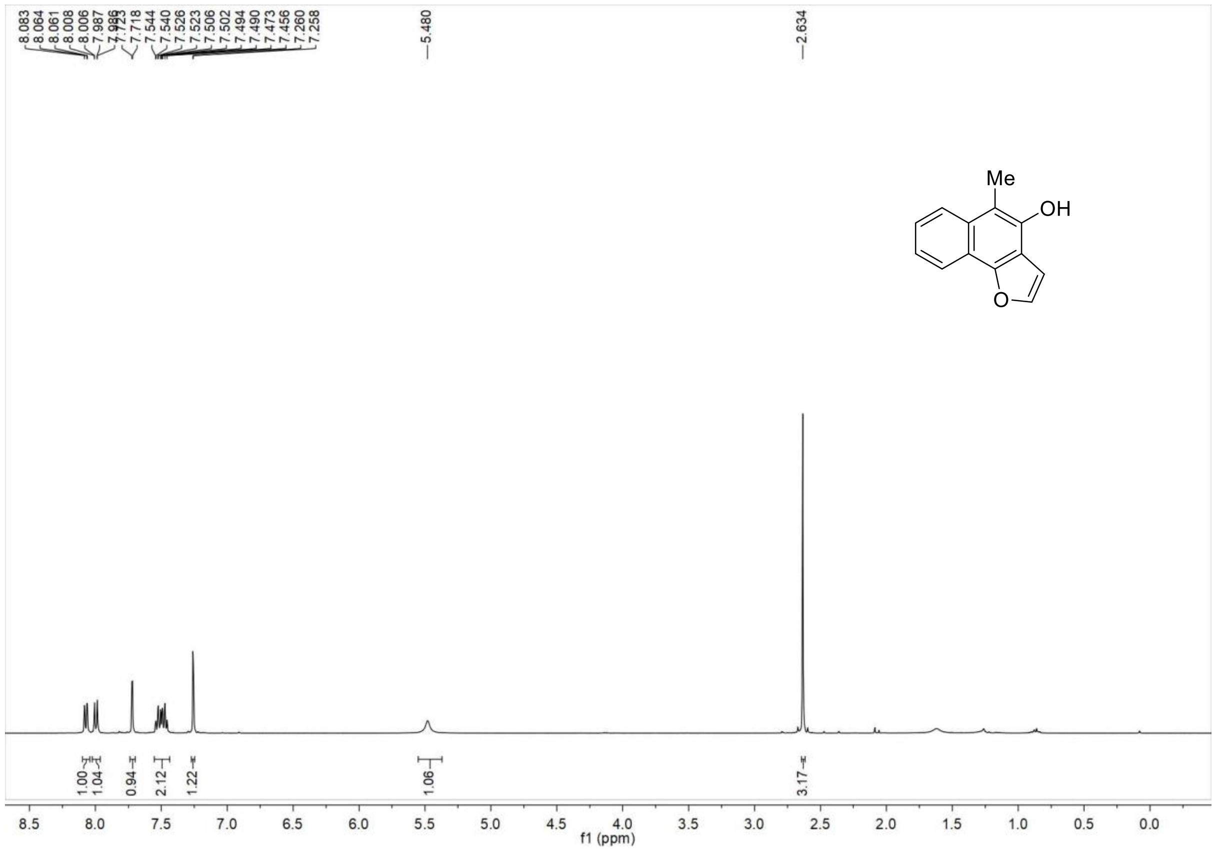

Supplementary Figure 65. ^{1}H-NMR of compound **32a**, recorded at 400 MHz and 25 °C in $CDCl_3$

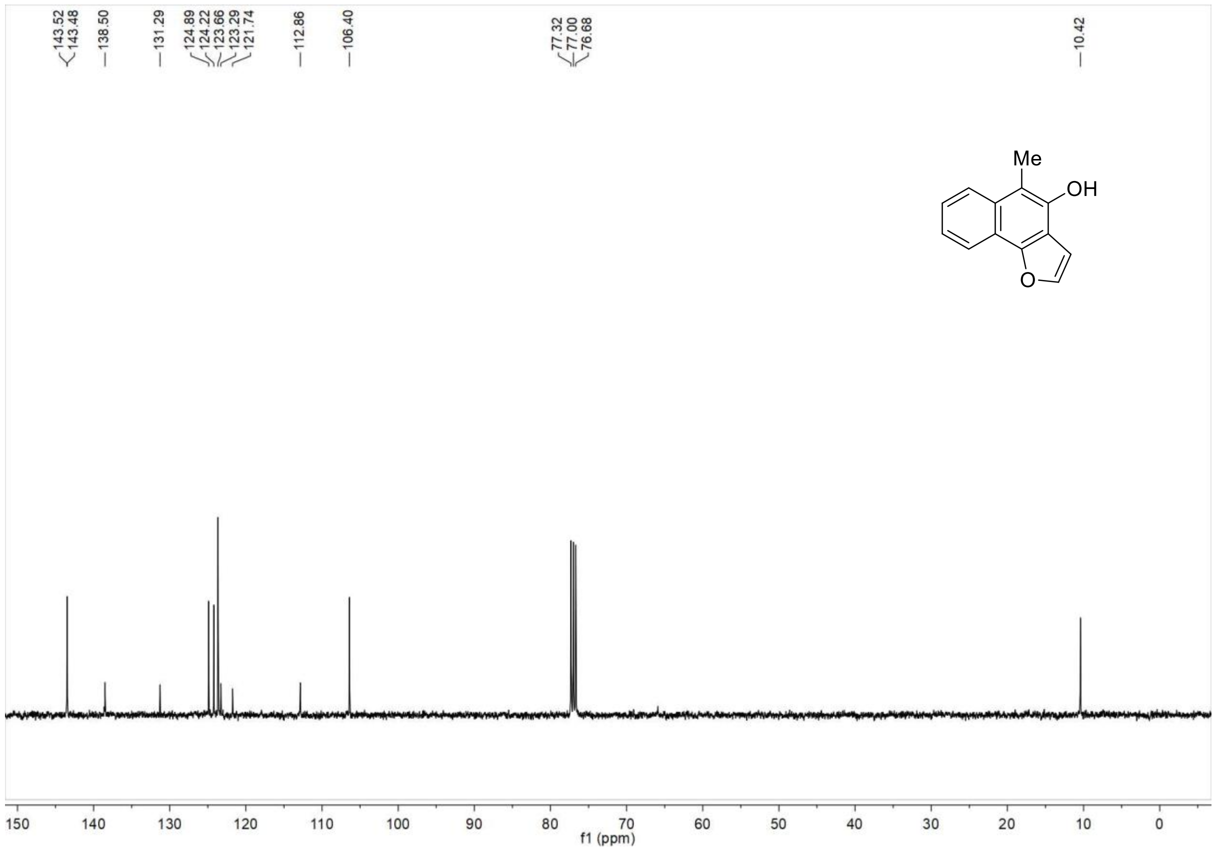

Supplementary Figure 66. ^{13}C-NMR of compound **32a**, recorded at 400 MHz and 25 °C in $CDCl_3$

1,3-Dimethylanthracen-2-ol (33a)

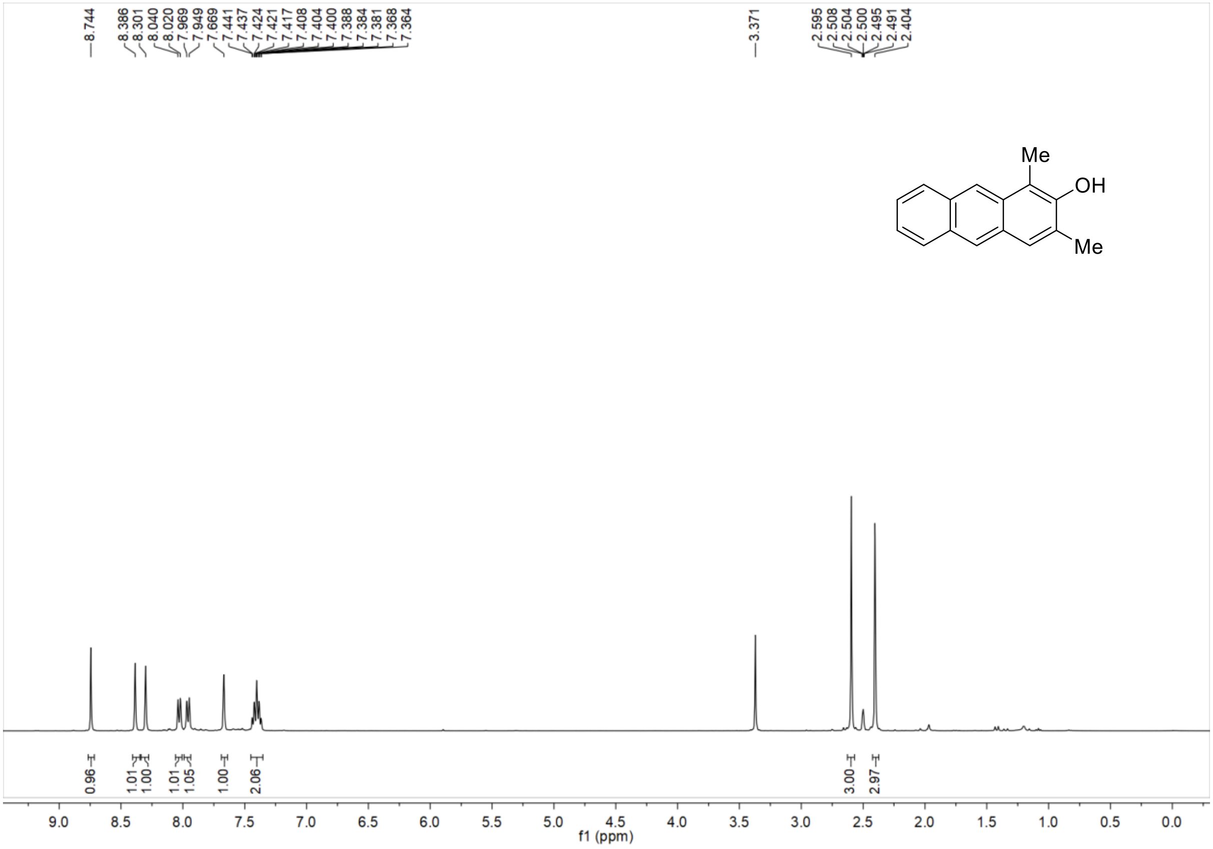

Supplementary Figure 67. ^{1}H-NMR of compound **33a**, recorded at 400 MHz and 25 °C in DMSO-d_6

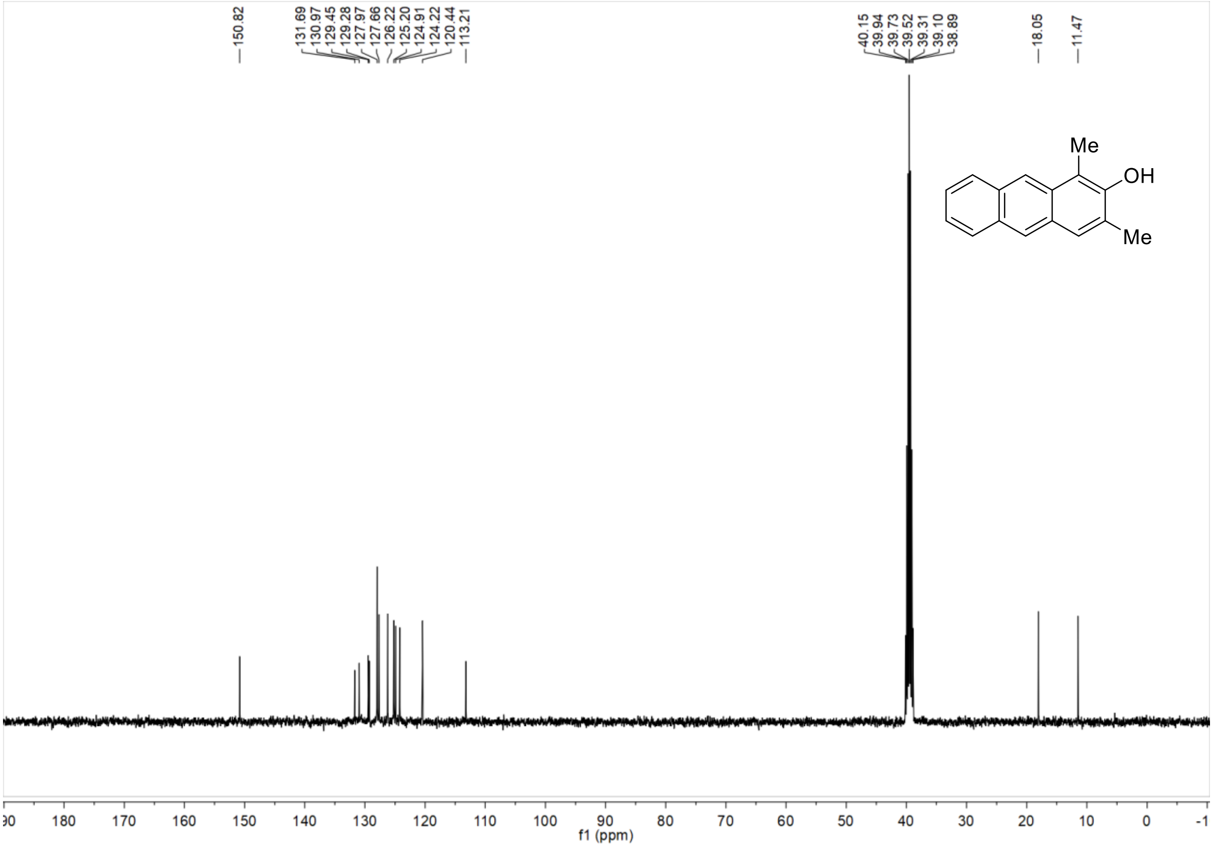

Supplementary Figure 68. ^{13}C-NMR of compound **33a**, recorded at 400 MHz and 25 °C in DMSO-d_6

2,4-Dimethyltetraphen-3-ol (34a)

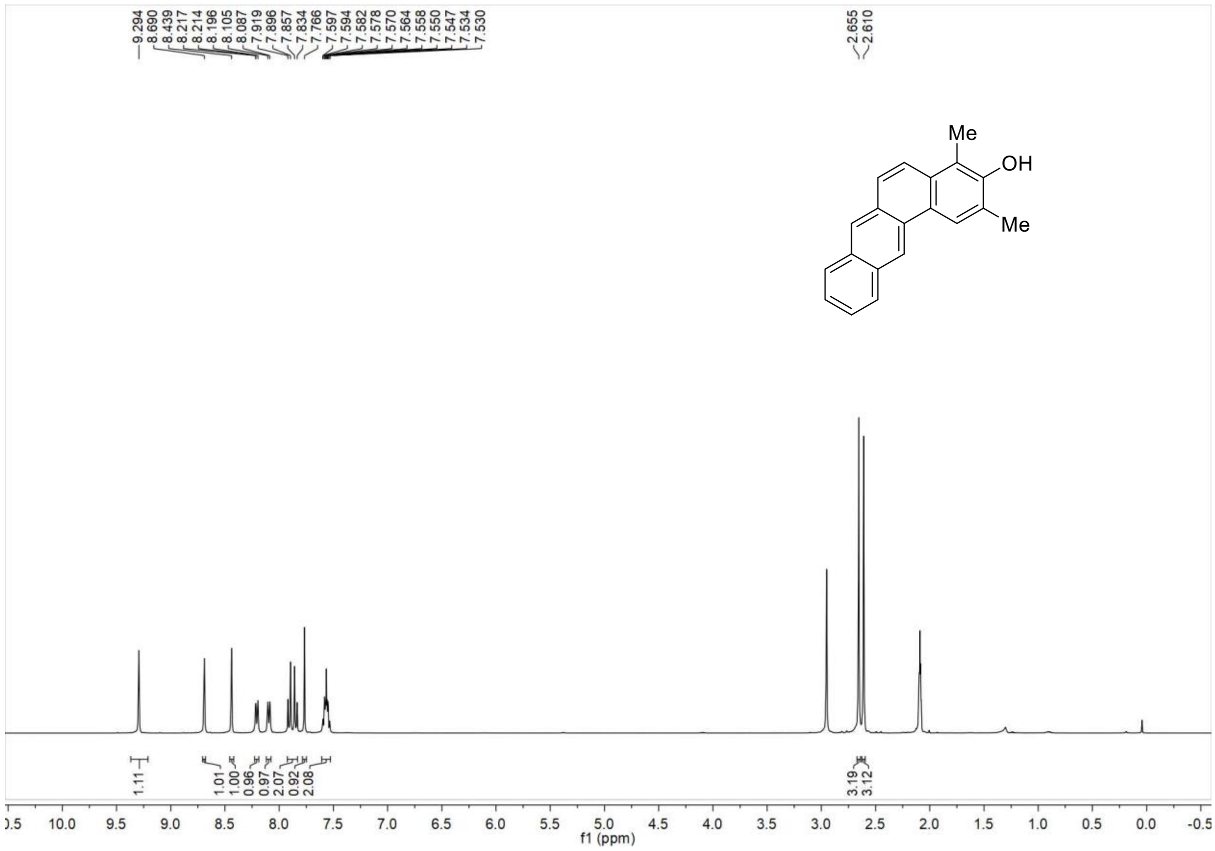

Supplementary Figure 69. ^{1}H-NMR of compound **34a**, recorded at 400 MHz and 25 °C in Acetone-d_6

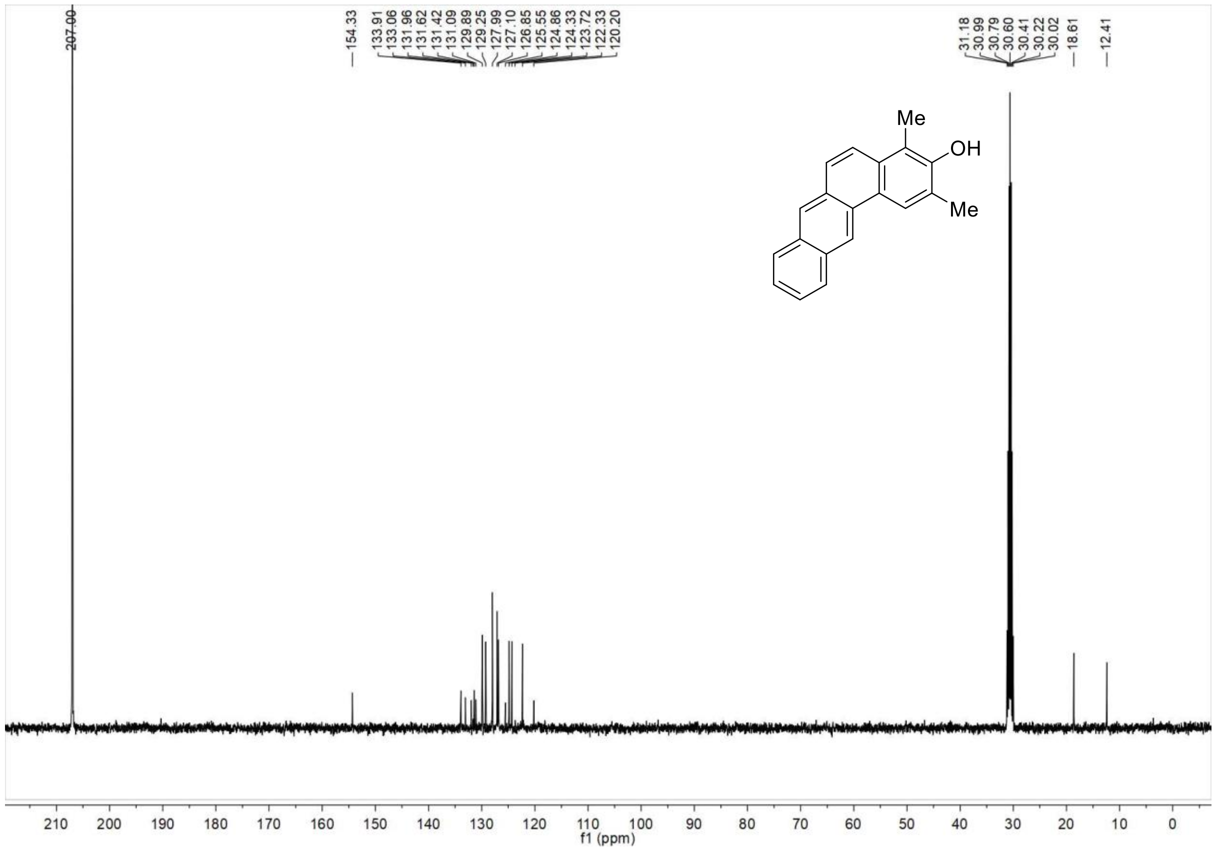

Supplementary Figure 70. ^{13}C-NMR of compound **34a**, recorded at 400 MHz and 25 °C in Acetone-d_6

5-Methylbenzo[*c*]phenanthren-6-ol (35a)

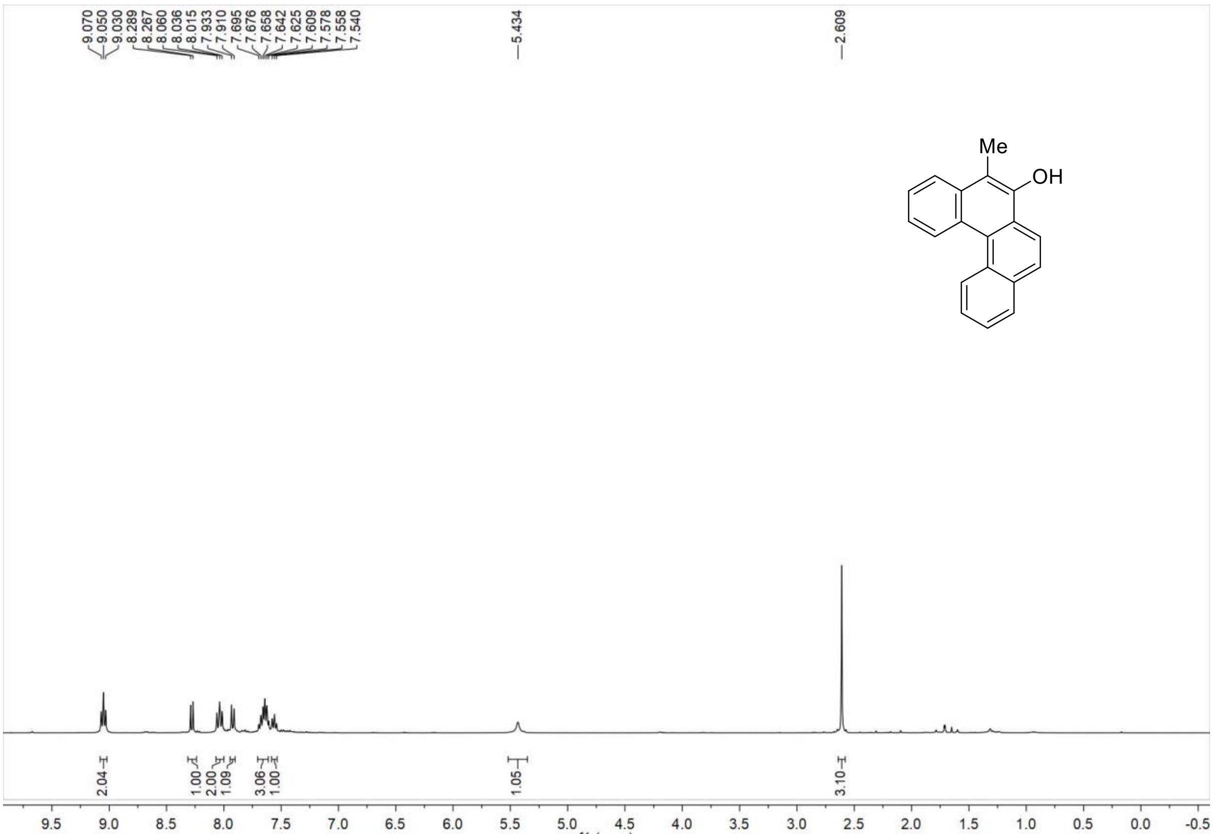

Supplementary Figure 71. ^{1}H-NMR of compound **35a**, recorded at 400 MHz and 25 °C in $CDCl_3$

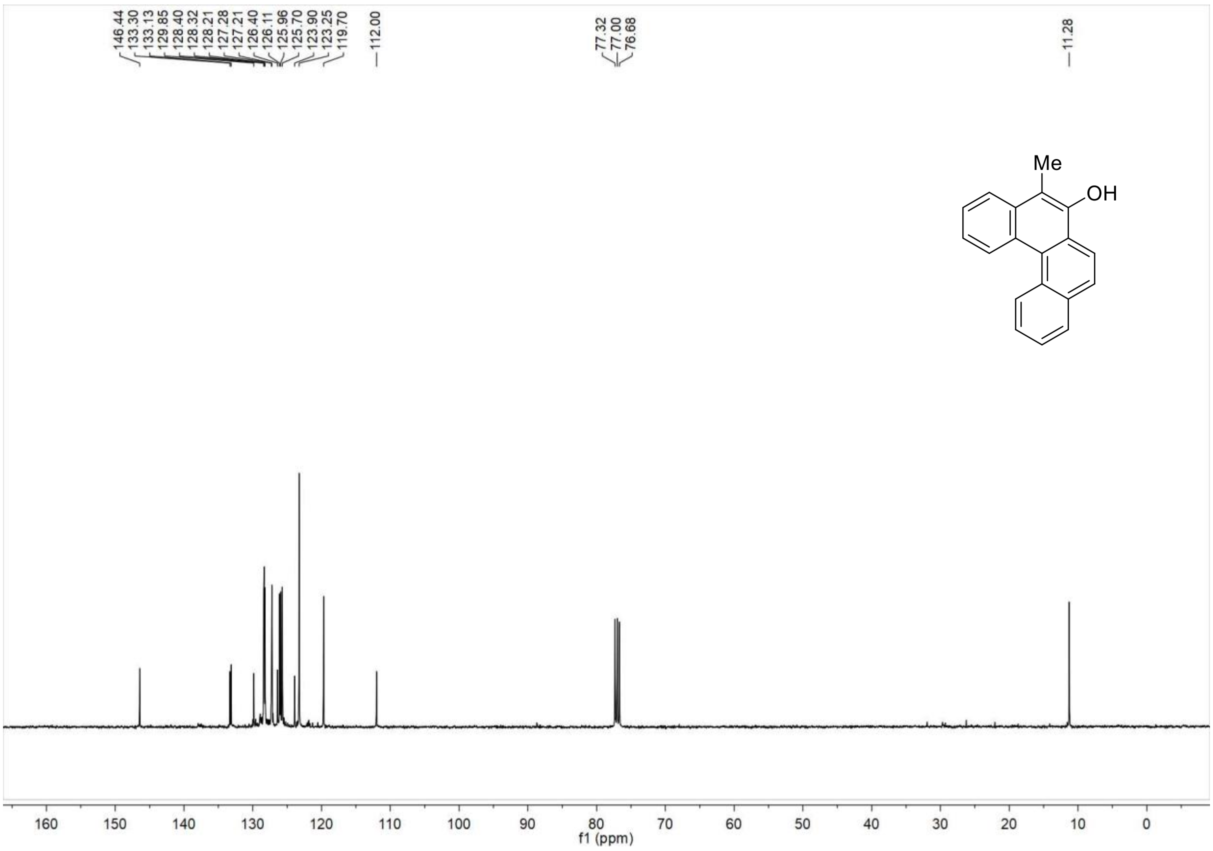

Supplementary Figure 72. ^{13}C-NMR of compound **35a**, recorded at 400 MHz and 25 °C in $CDCl_3$

1-(phenanthridin-6-yl)Ethan-1-one (1)

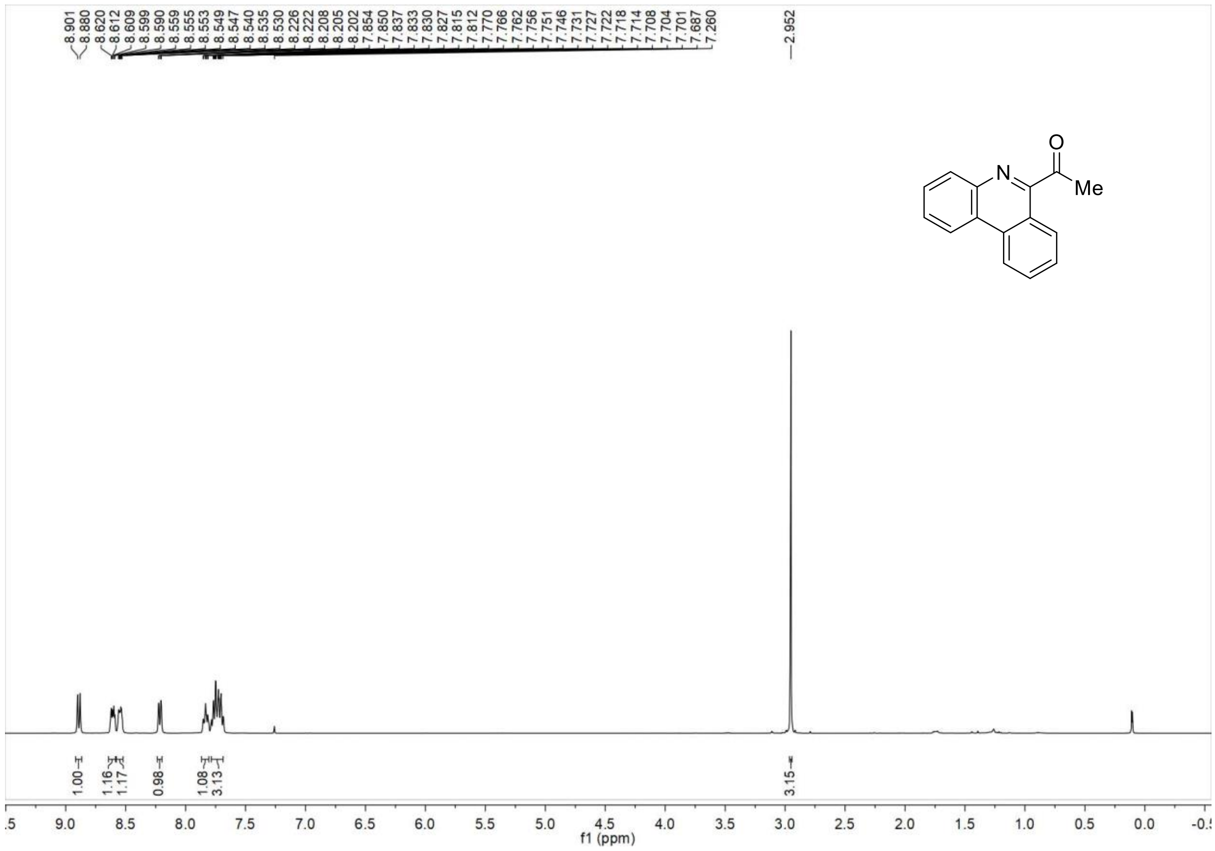

Supplementary Figure 73. ^{1}H-NMR of compound **1**, recorded at 400 MHz and 25 °C in $CDCl_3$

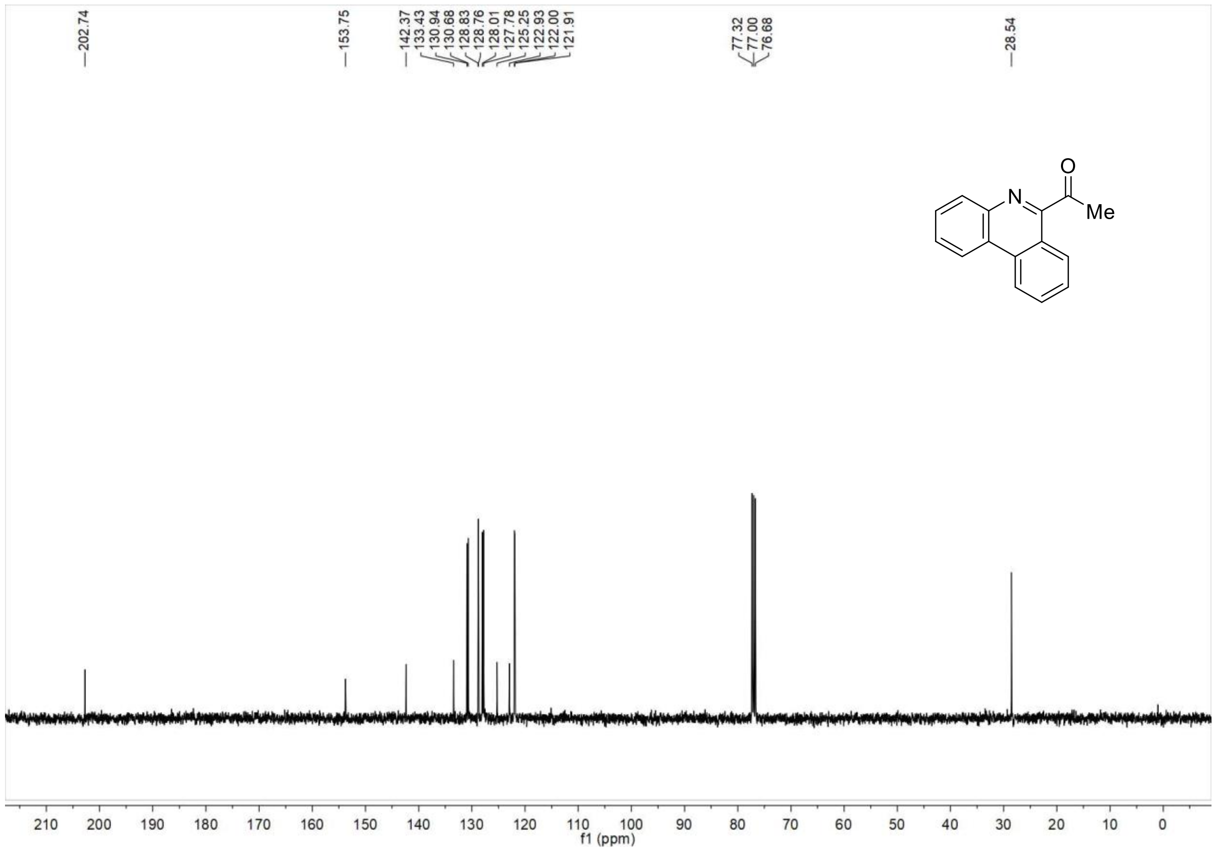

Supplementary Figure 74. ^{13}C-NMR of compound **1**, recorded at 400 MHz and 25 °C in $CDCl_3$

Cyclopropyl(phenanthridin-6-yl)methanone (2)

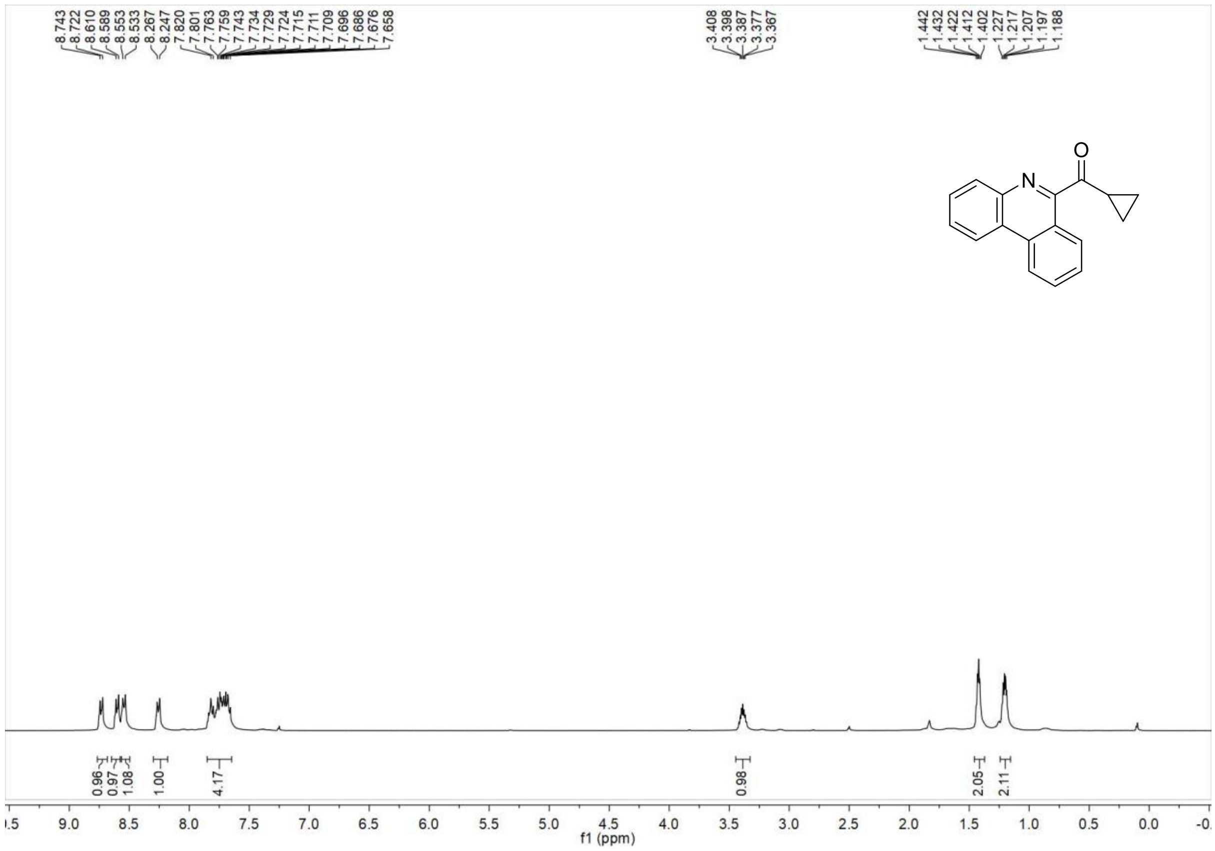

Supplementary Figure 75. ^{1}H-NMR of compound **2**, recorded at 400 MHz and 25 °C in $CDCl_3$

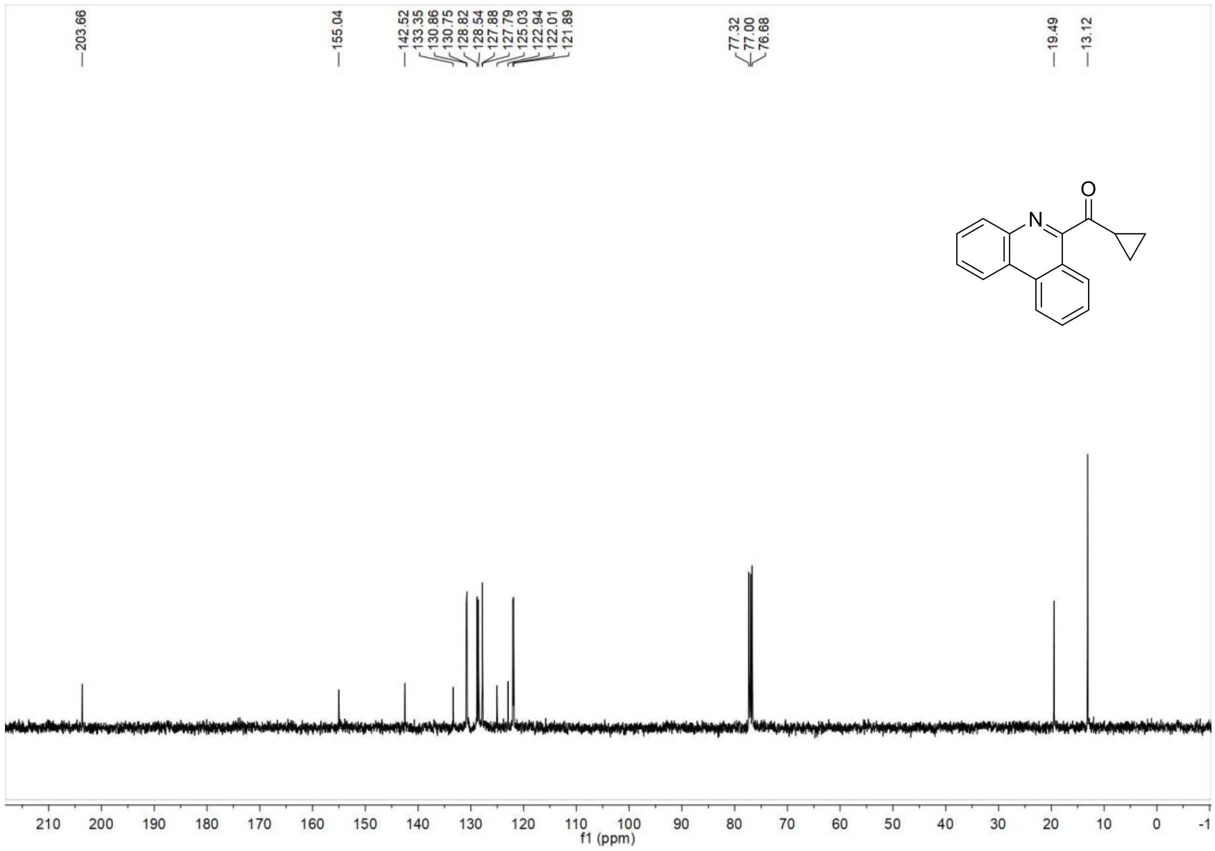

Supplementary Figure 76. ^{13}C-NMR of compound **2**, recorded at 400 MHz and 25 °C in $CDCl_3$

1-(8-phenylphenanthridin-6-yl)Ethan-1-one (3)

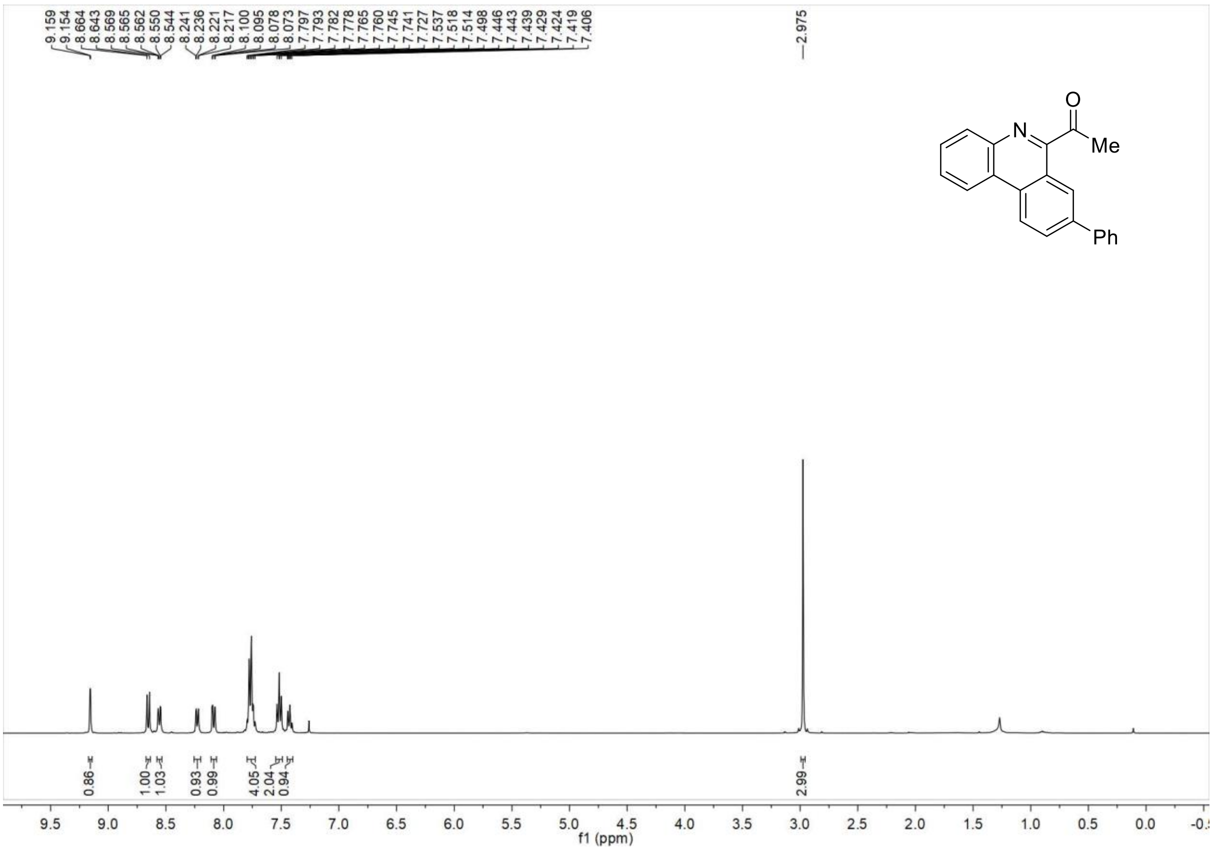

Supplementary Figure 77. ^{1}H-NMR of compound **3**, recorded at 400 MHz and 25 °C in $CDCl_3$

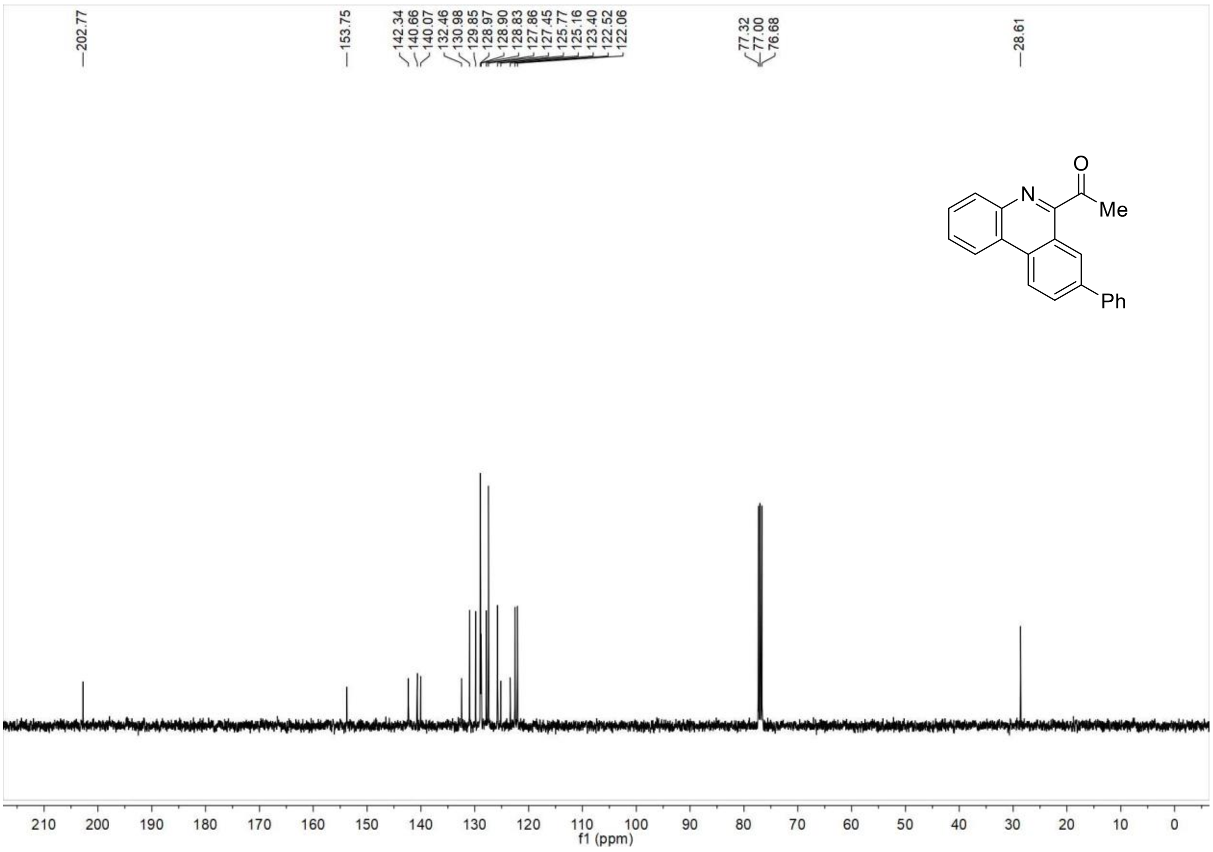

Supplementary Figure 78. ^{13}C-NMR of compound **3**, recorded at 400 MHz and 25 °C in $CDCl_3$

1-(3-methoxyphenanthridin-6-yl)Ethan-1-one (4)

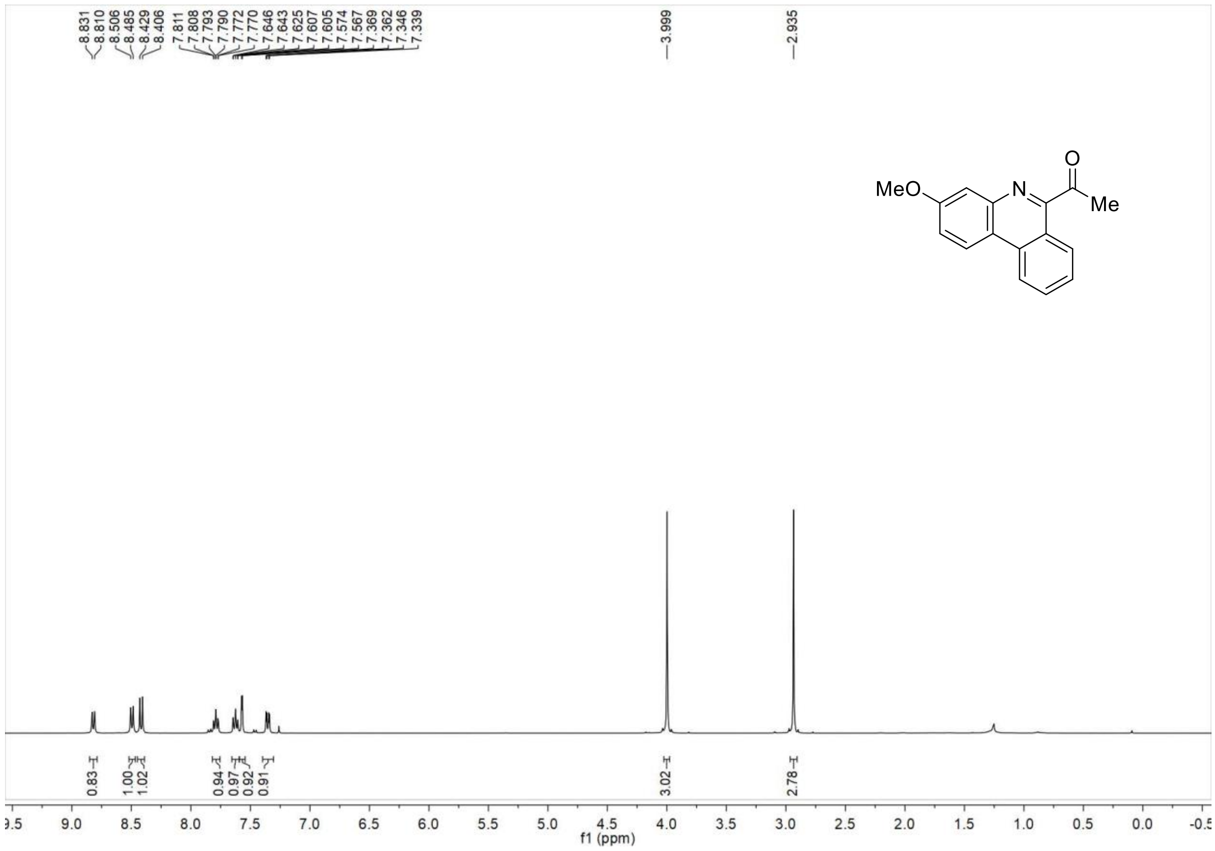

Supplementary Figure 79. ^{1}H-NMR of compound **4**, recorded at 400 MHz and 25 °C in $CDCl_3$

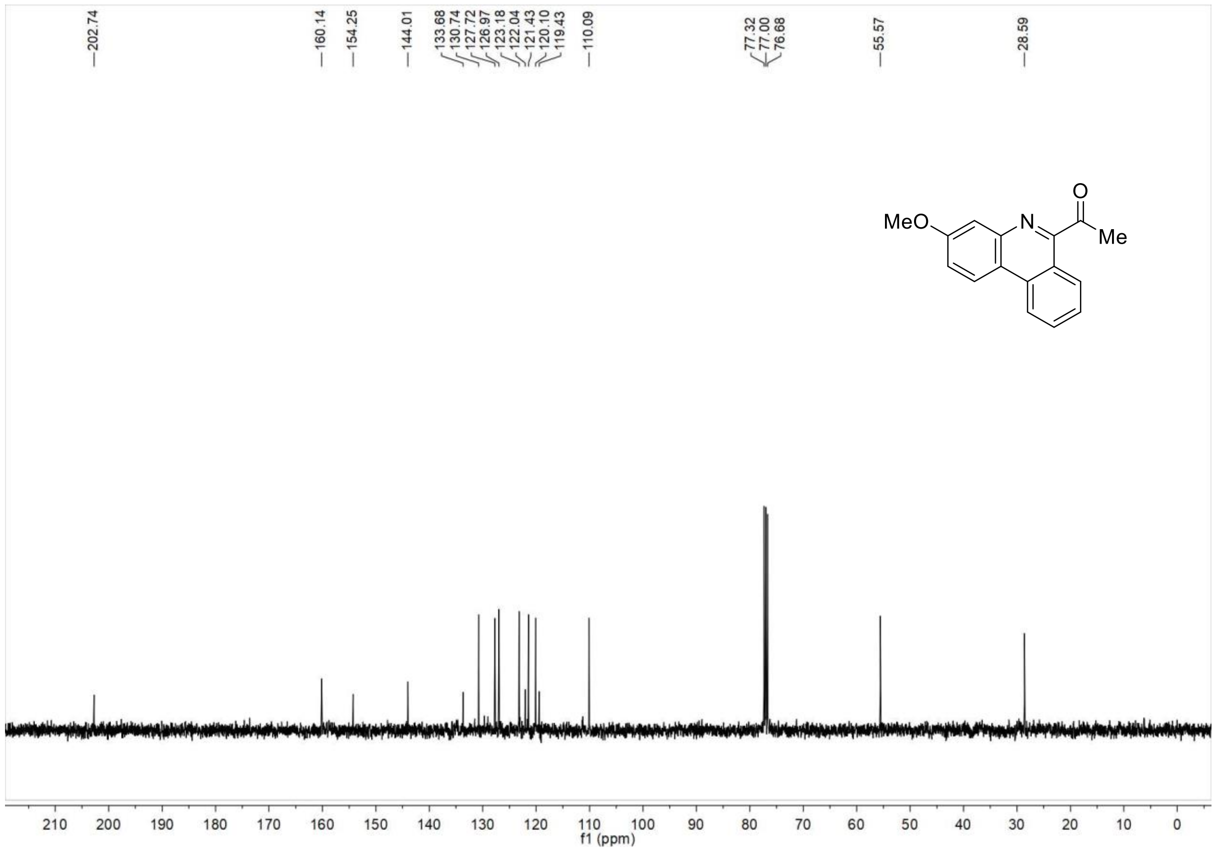

Supplementary Figure 80. ^{13}C-NMR of compound **4**, recorded at 400 MHz and 25 °C in $CDCl_3$

1-(8-(benzyloxy)Phenanthridin-6-yl)ethan-1-one (5)

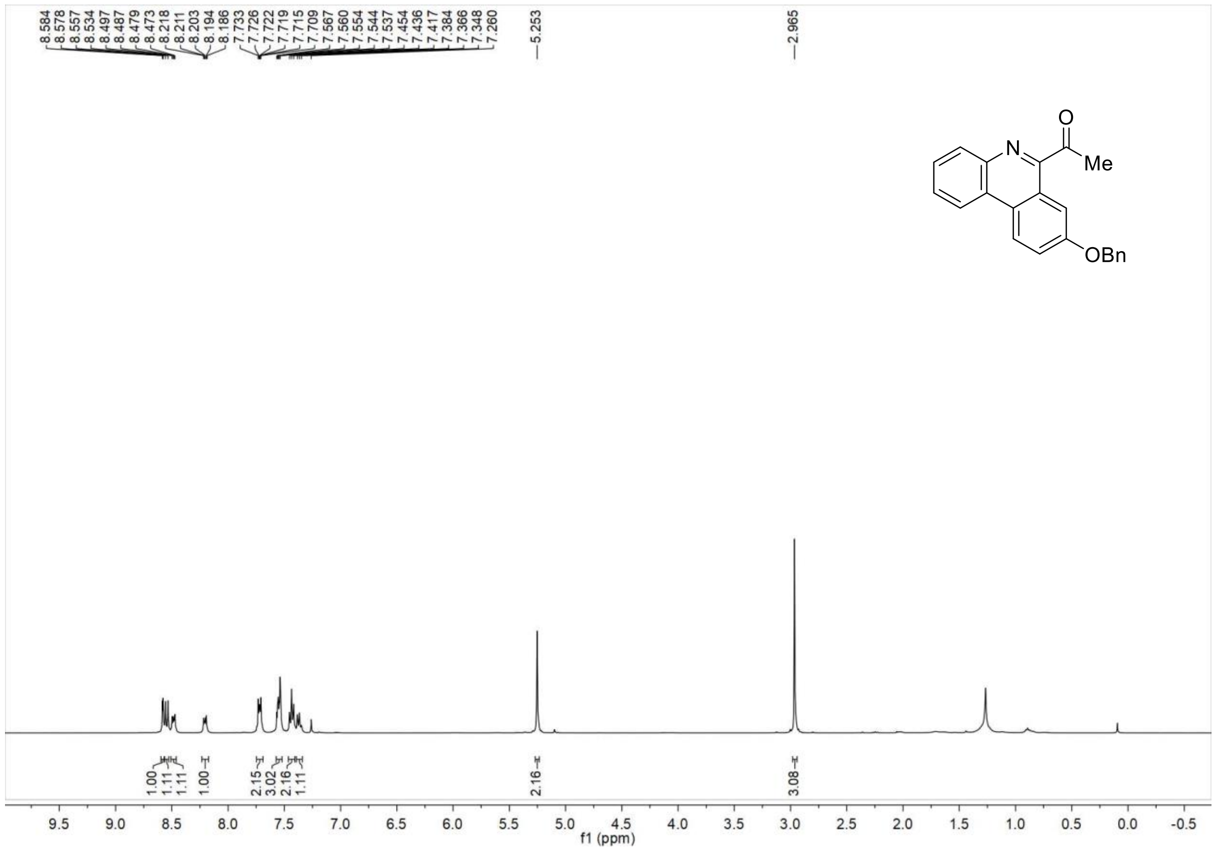

Supplementary Figure 81. ^{1}H-NMR of compound **5**, recorded at 400 MHz and 25 °C in $CDCl_3$

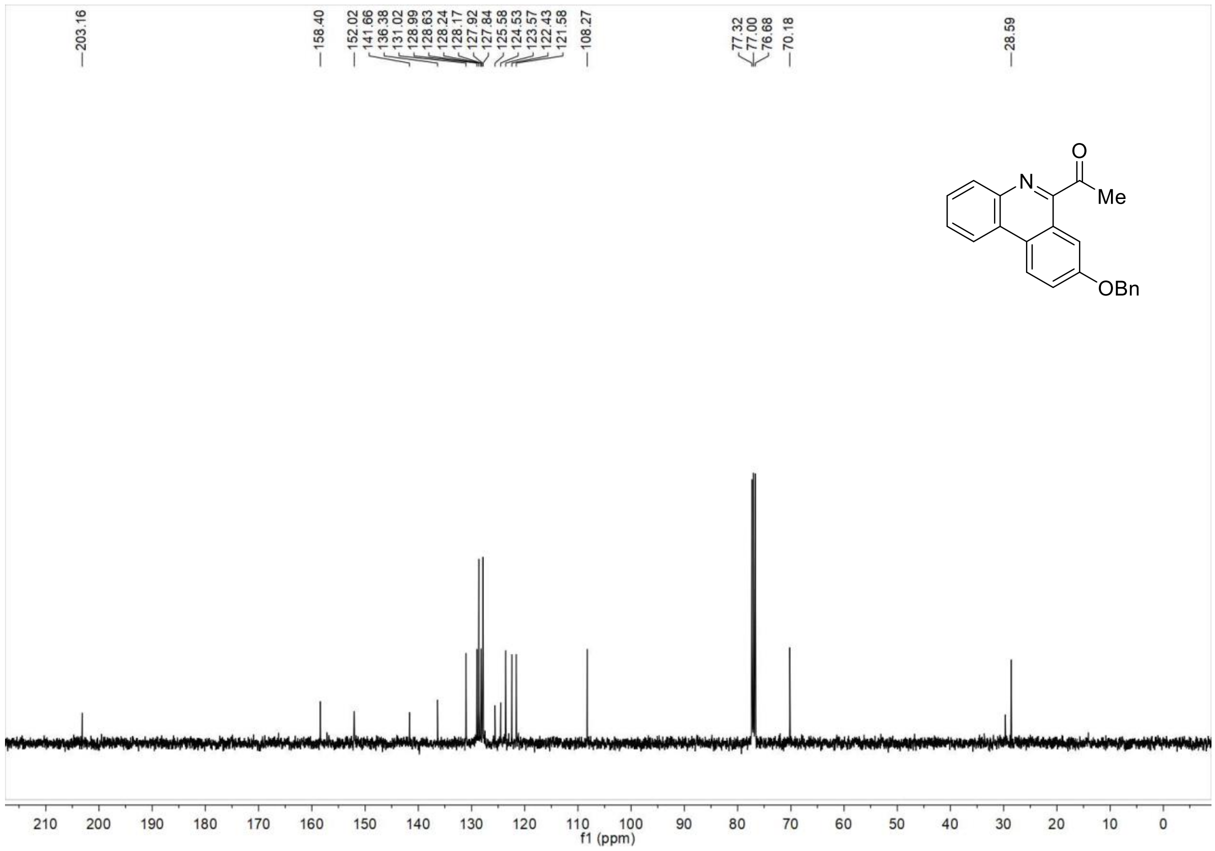

Supplementary Figure 82. ^{13}C-NMR of compound **5**, recorded at 400 MHz and 25 °C in $CDCl_3$

1-([1,3]dioxolo[4,5-*j*]phenanthridin-6-yl)Ethan-1-one (6)

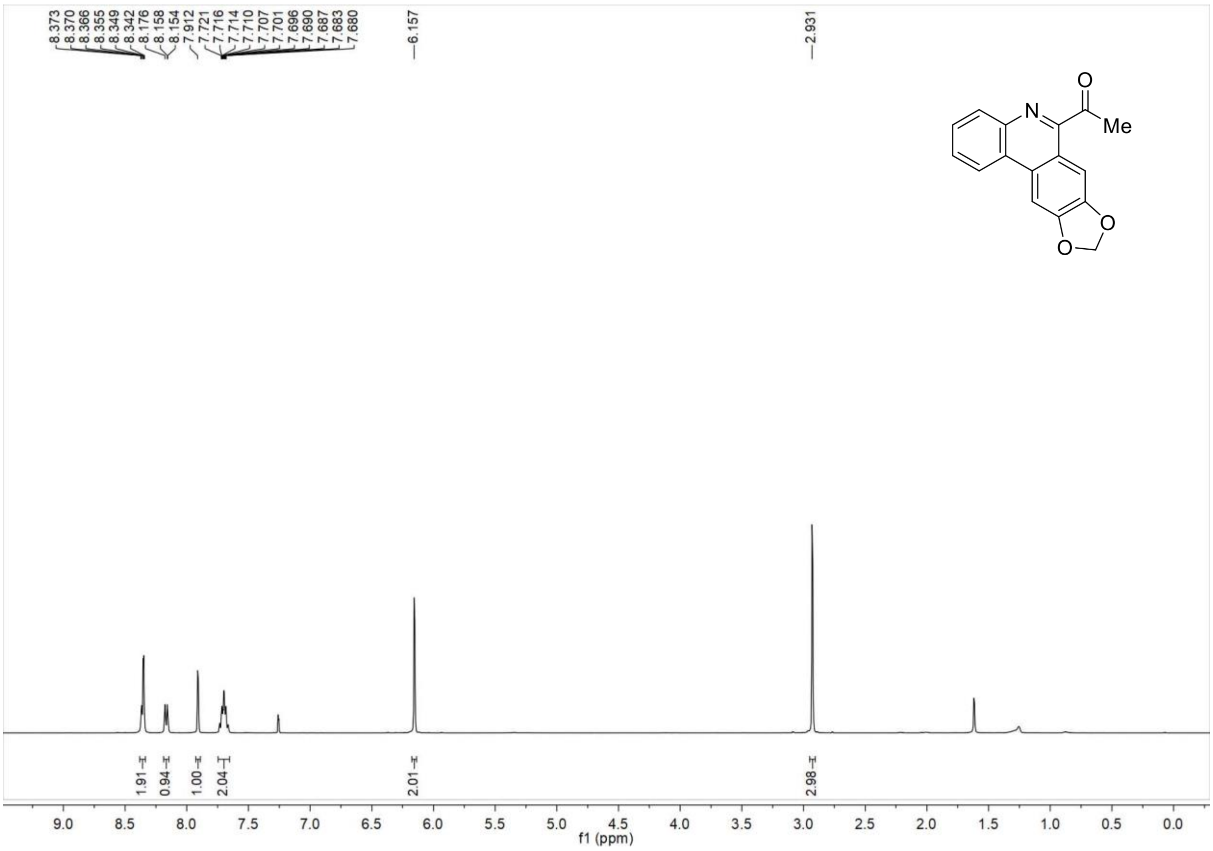

Supplementary Figure 83. ^{1}H-NMR of compound **6**, recorded at 400 MHz and 25 °C in $CDCl_3$

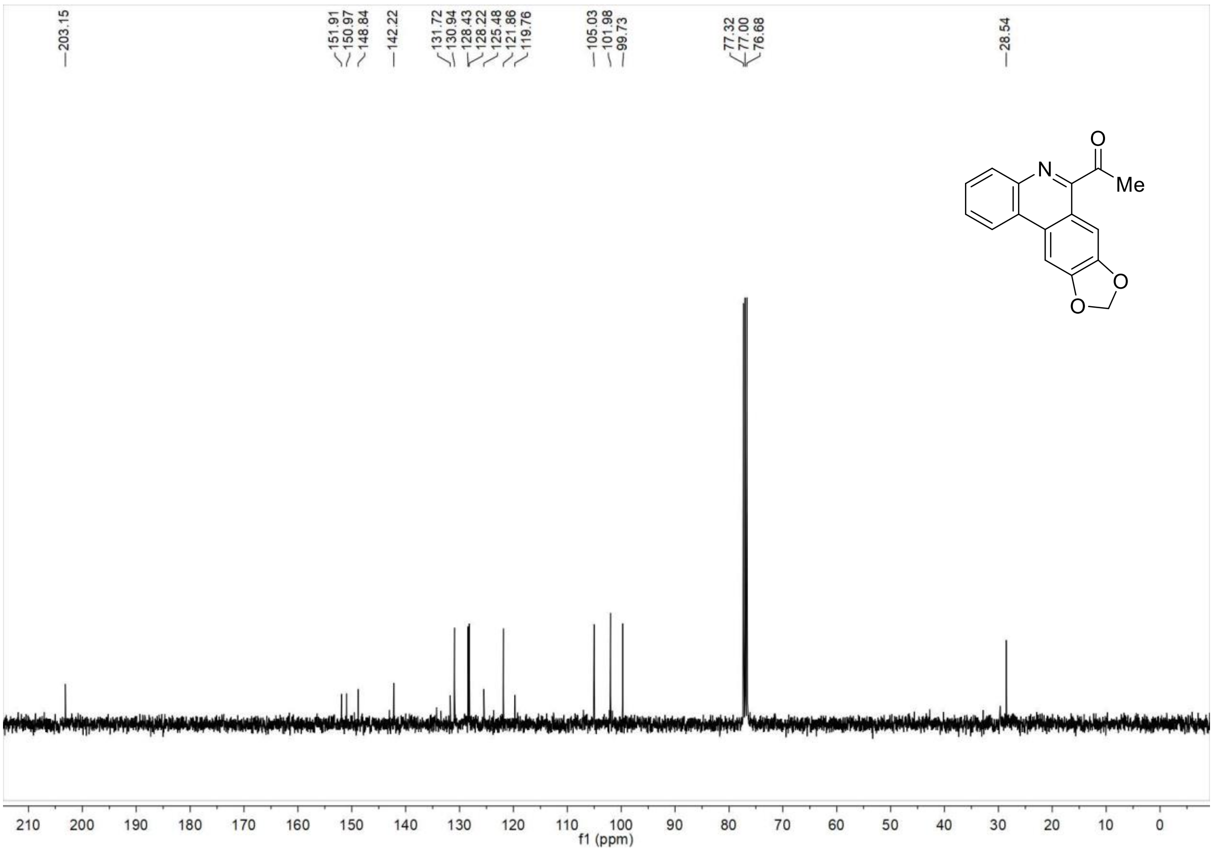

Supplementary Figure 84. ^{13}C-NMR of compound **6**, recorded at 400 MHz and 25 °C in $CDCl_3$

1-(2-fluorophenanthridin-6-yl)Ethan-1-one (7)

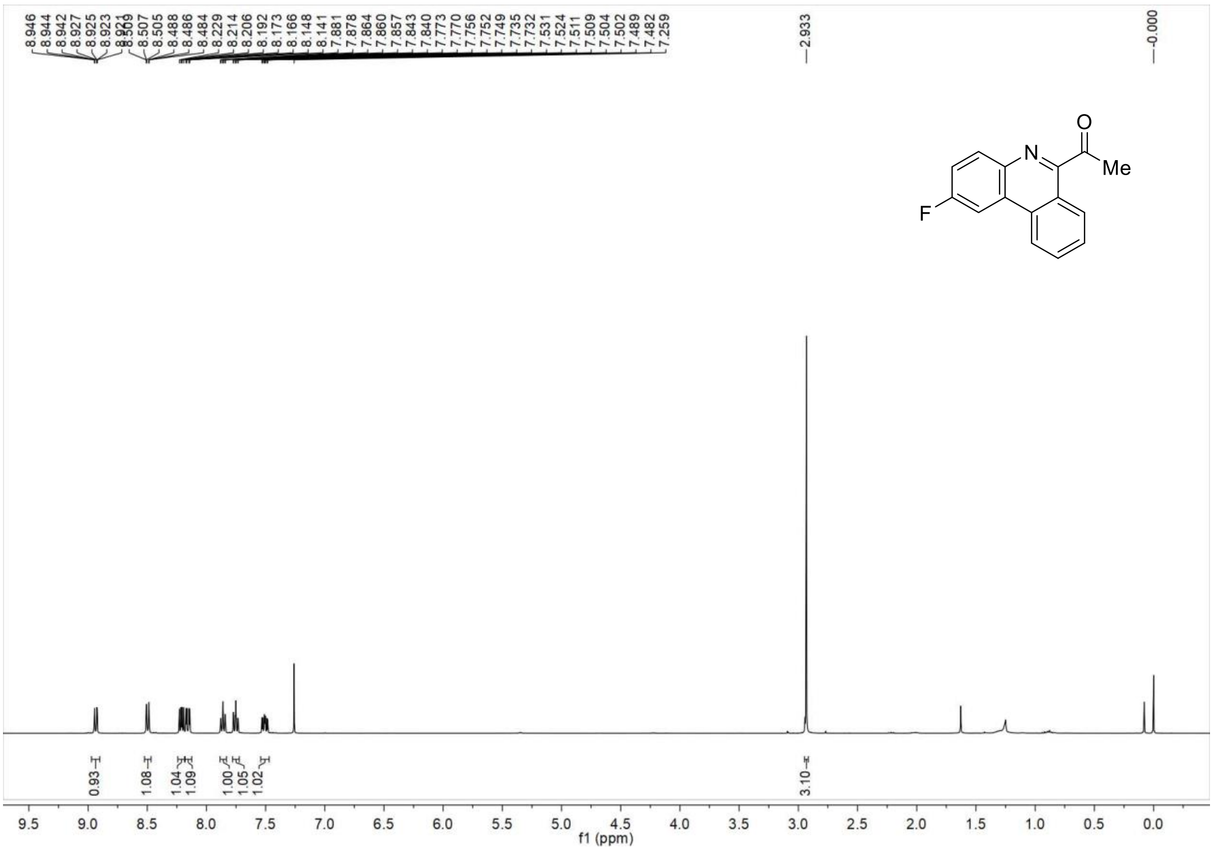

Supplementary Figure 85. ^{1}H-NMR of compound **7**, recorded at 400 MHz and 25 °C in $CDCl_3$

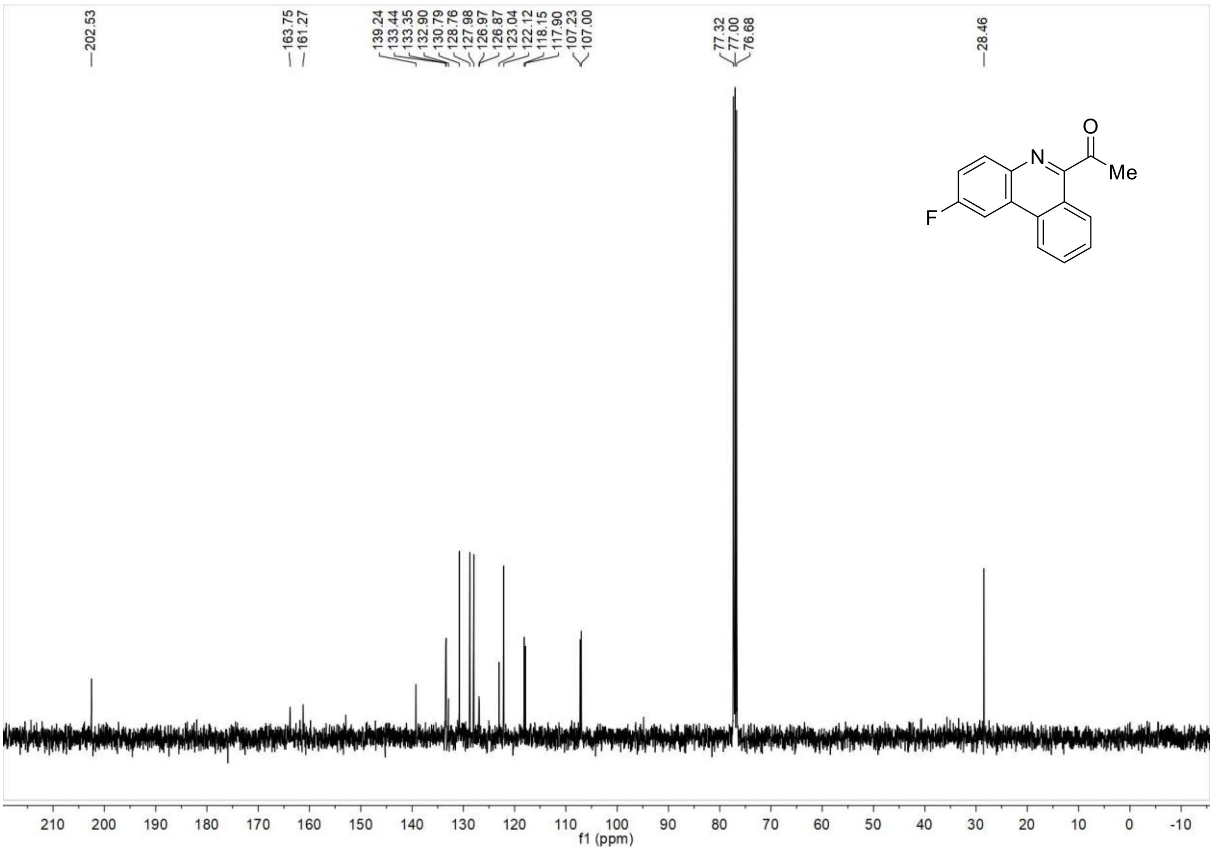

Supplementary Figure 86. ^{13}C-NMR of compound **7**, recorded at 400 MHz and 25 °C in $CDCl_3$

1-(9-chlorophenanthridin-6-yl)Ethan-1-one (8)

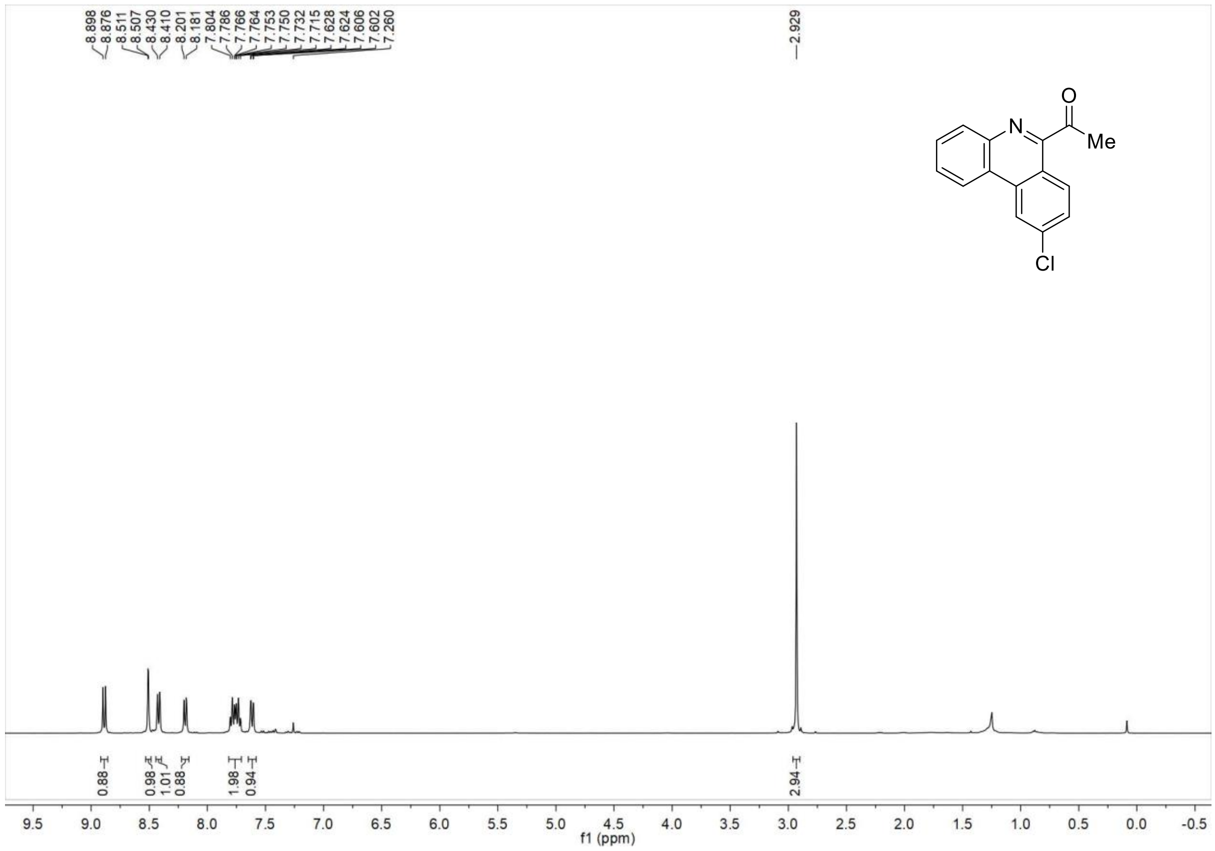

Supplementary Figure 87. ^{1}H-NMR of compound **8**, recorded at 400 MHz and 25 °C in $CDCl_3$

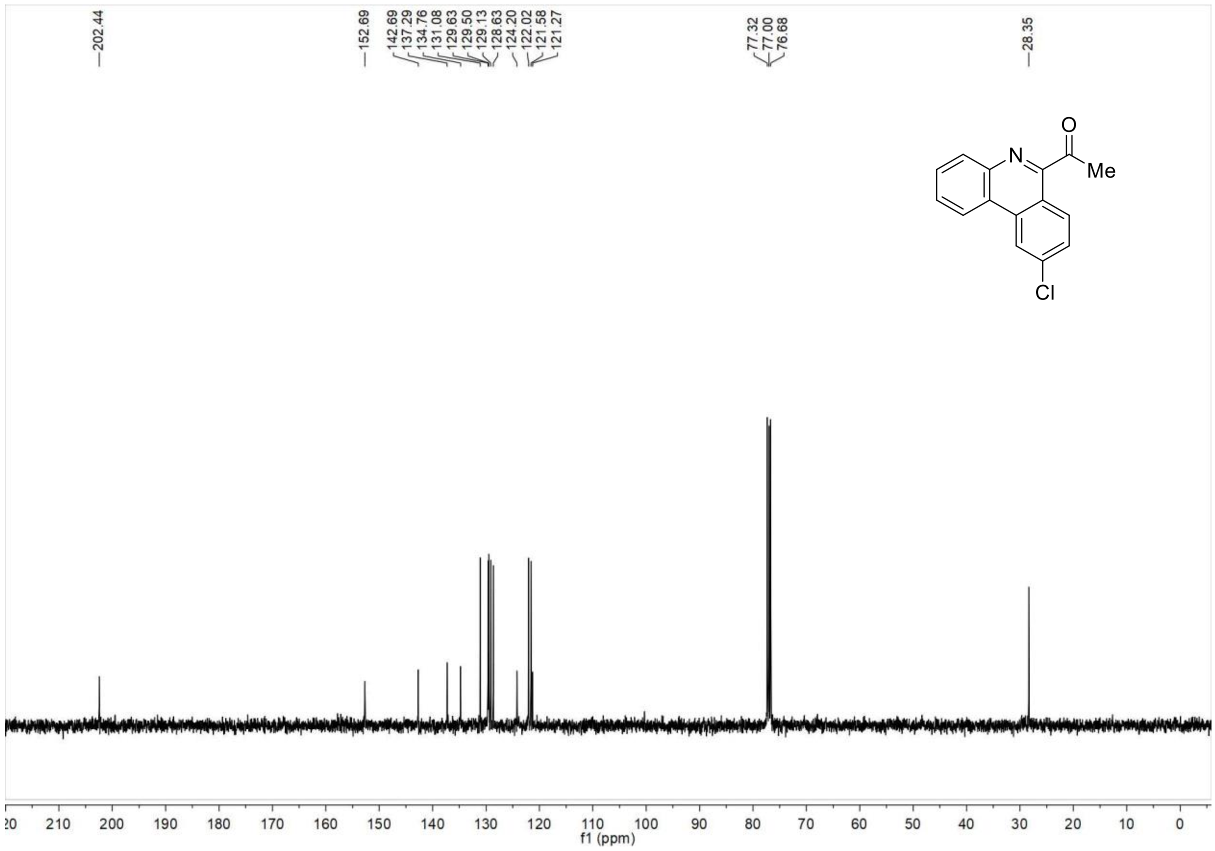

Supplementary Figure 88. ^{13}C-NMR of compound **8**, recorded at 400 MHz and 25 °C in $CDCl_3$

***tert*-Butyl 6-acetylphenanthridine-9-carboxylate (9)**

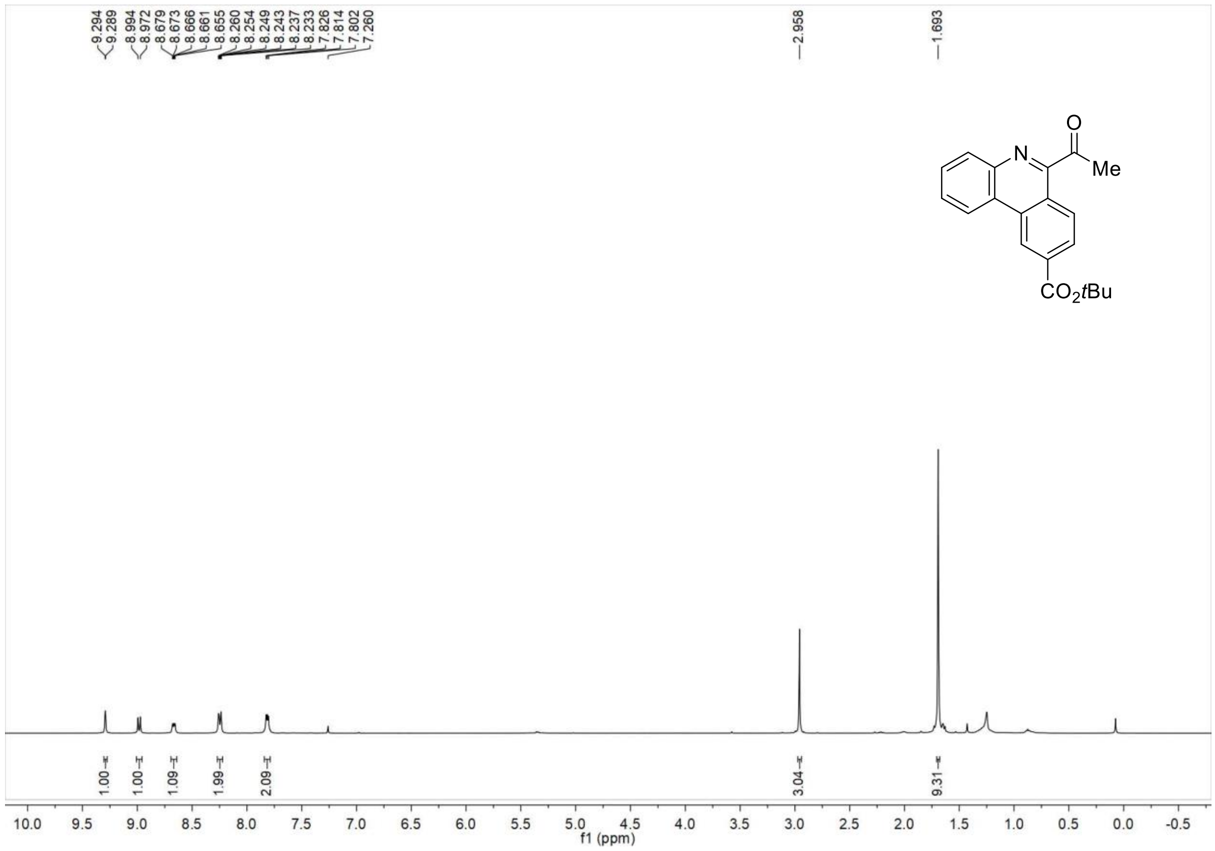

Supplementary Figure 89. ^{1}H-NMR of compound **9**, recorded at 400 MHz and 25 °C in $CDCl_3$

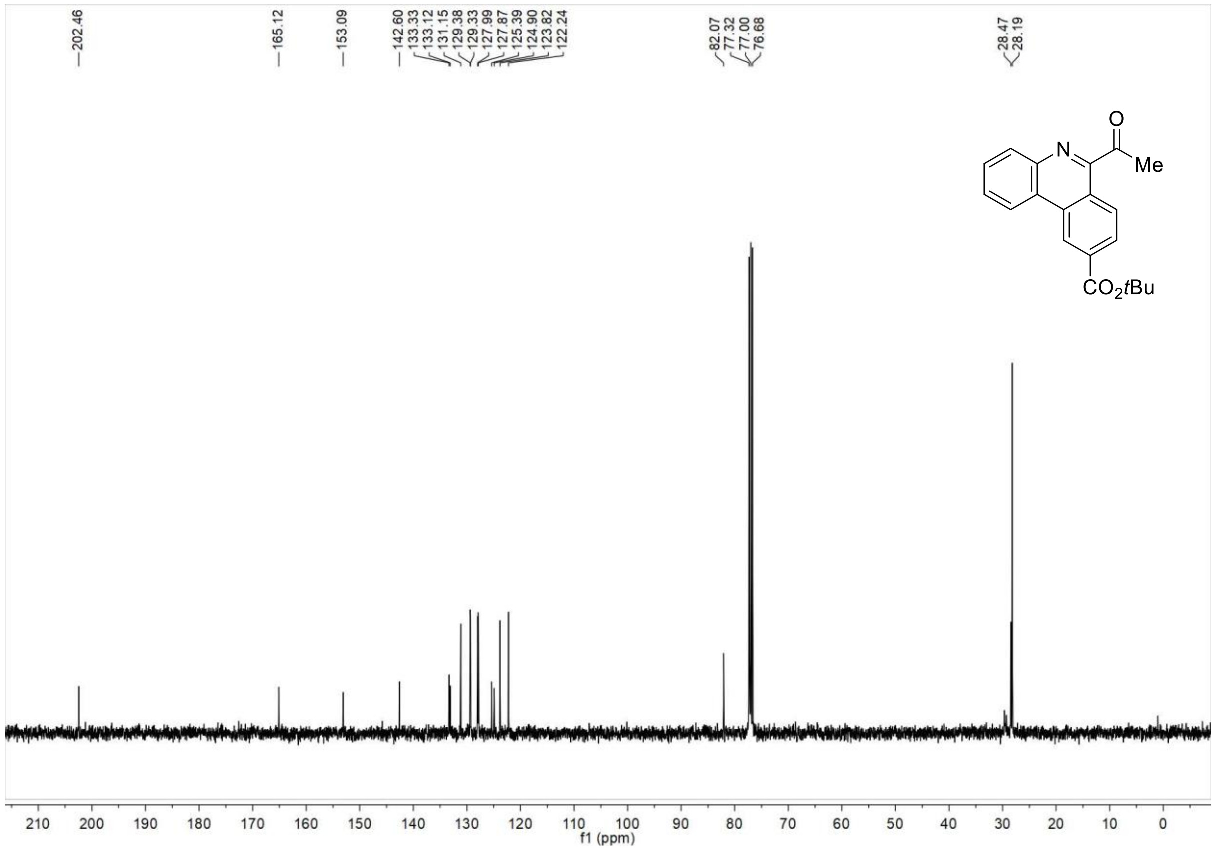

Supplementary Figure 90. ^{13}C-NMR of compound **9**, recorded at 400 MHz and 25 °C in $CDCl_3$

1-(8-(trifluoromethyl)phenanthridin-6-yl)Ethan-1-one (10)

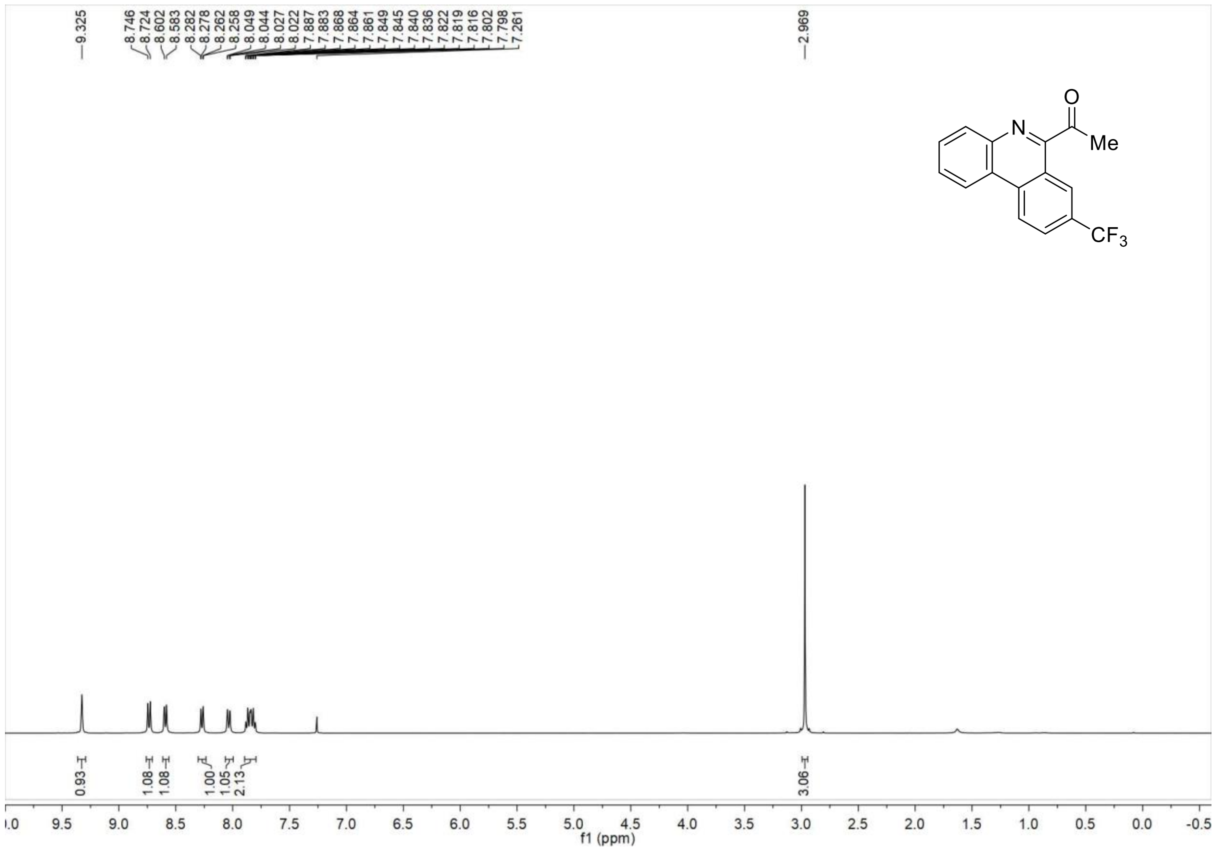

Supplementary Figure 91. ^{1}H-NMR of compound **10**, recorded at 400 MHz and 25 °C in $CDCl_3$

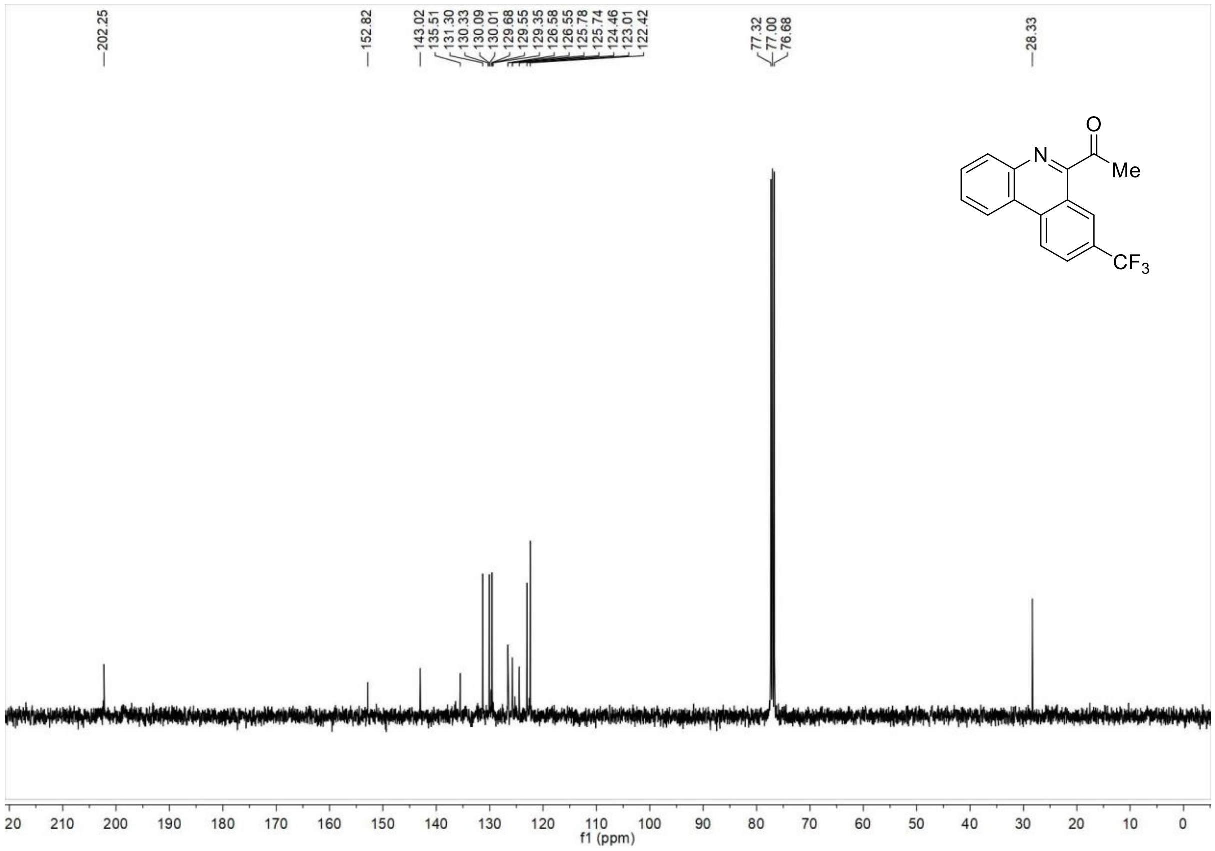

Supplementary Figure 92. ^{13}C-NMR of compound **10**, recorded at 400 MHz and 25 °C in $CDCl_3$

1-(quinolin-2-yl)Ethan-1-one (11)

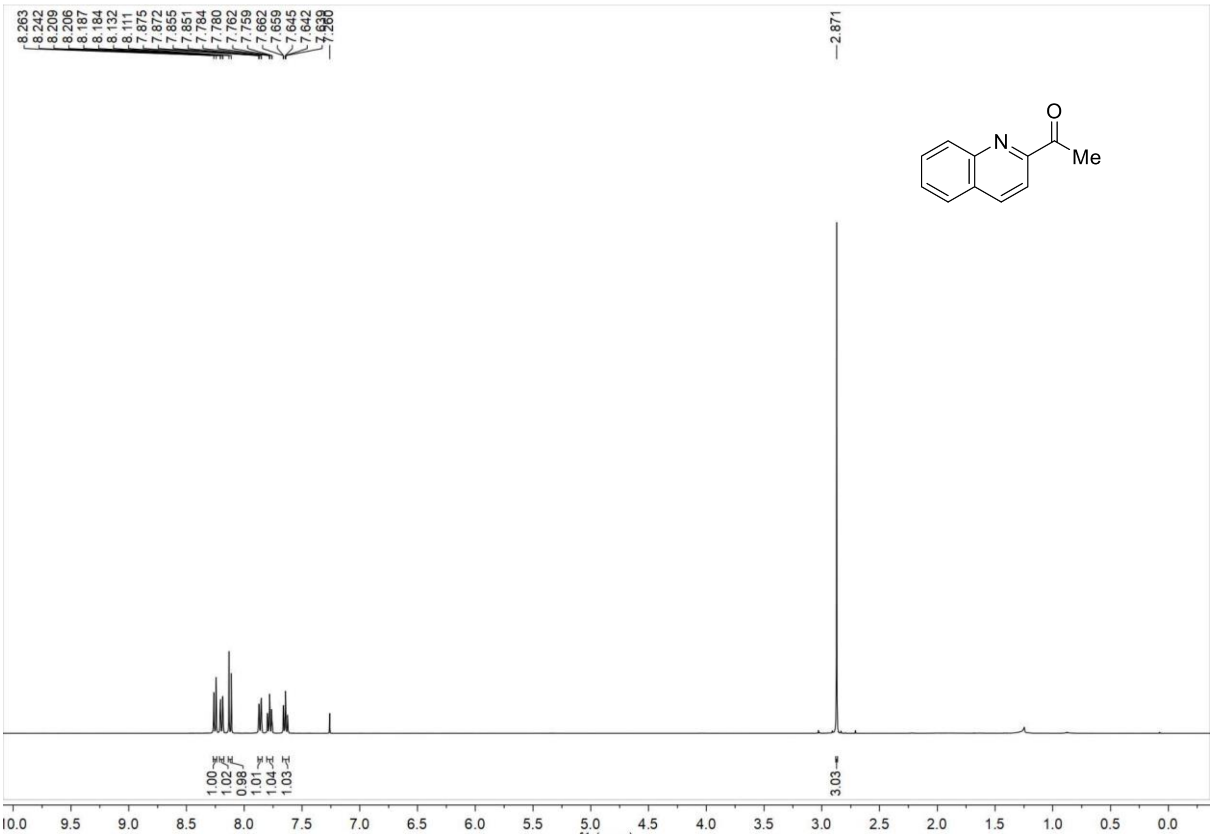

Supplementary Figure 93. ^{1}H-NMR of compound **11**, recorded at 400 MHz and 25 °C in $CDCl_3$

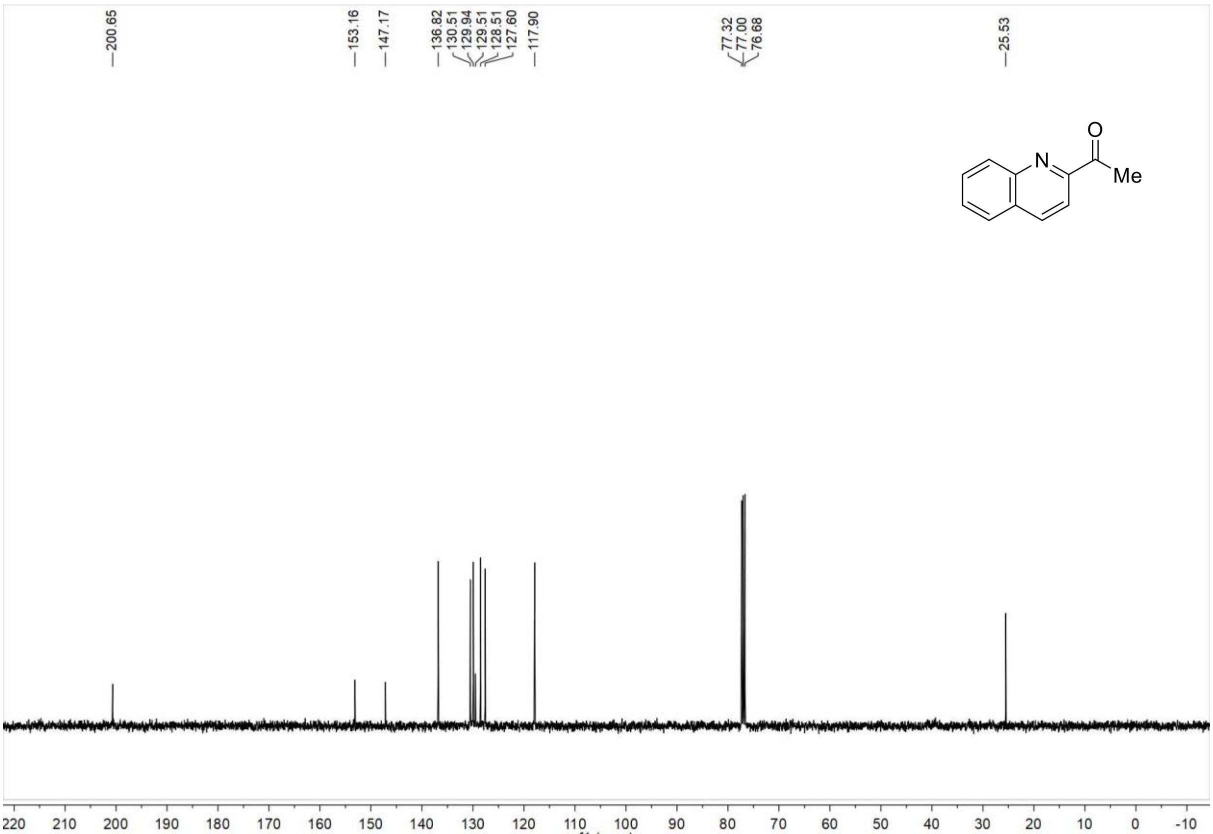

Supplementary Figure 94. ^{13}C-NMR of compound **11**, recorded at 400 MHz and 25 °C in $CDCl_3$

1-(quinolin-2-yl)Nonan-1-one (12)

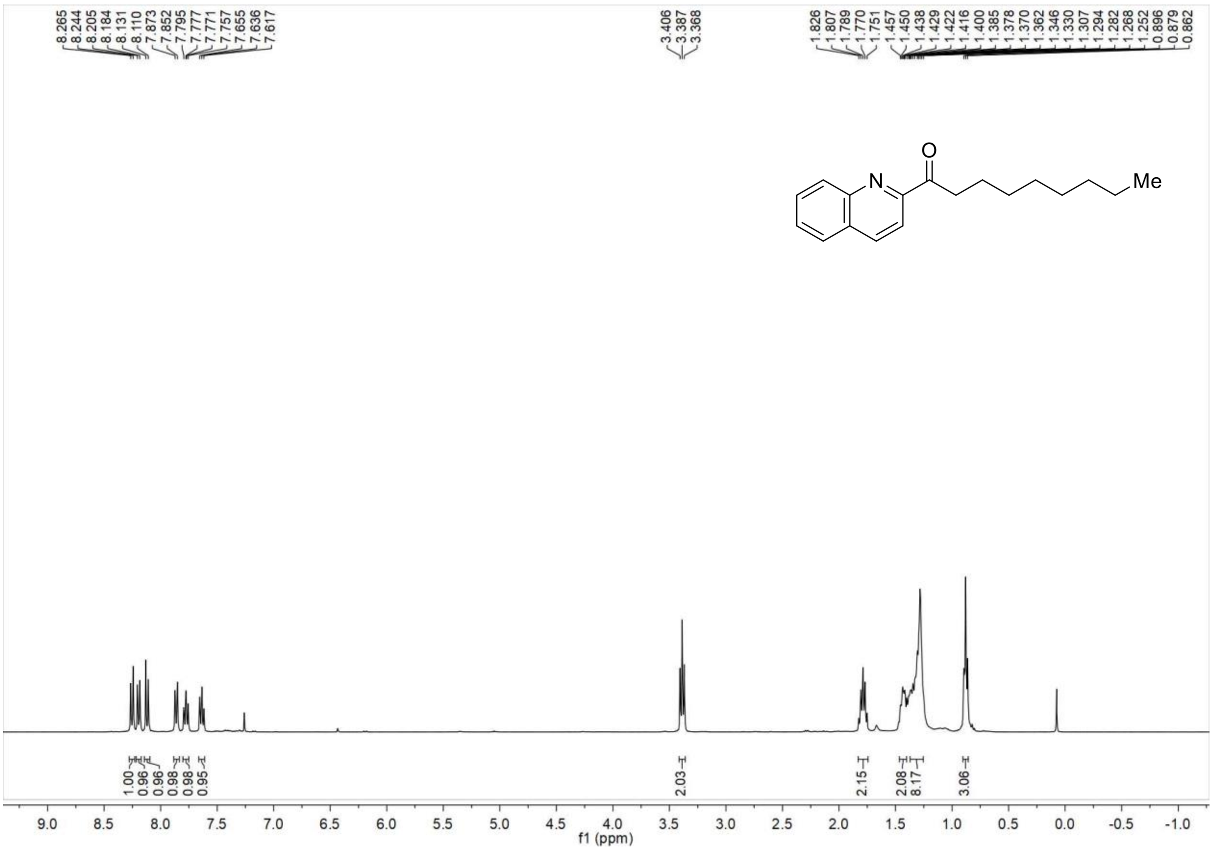

Supplementary Figure 95. ^{1}H-NMR of compound **12**, recorded at 400 MHz and 25 °C in $CDCl_3$

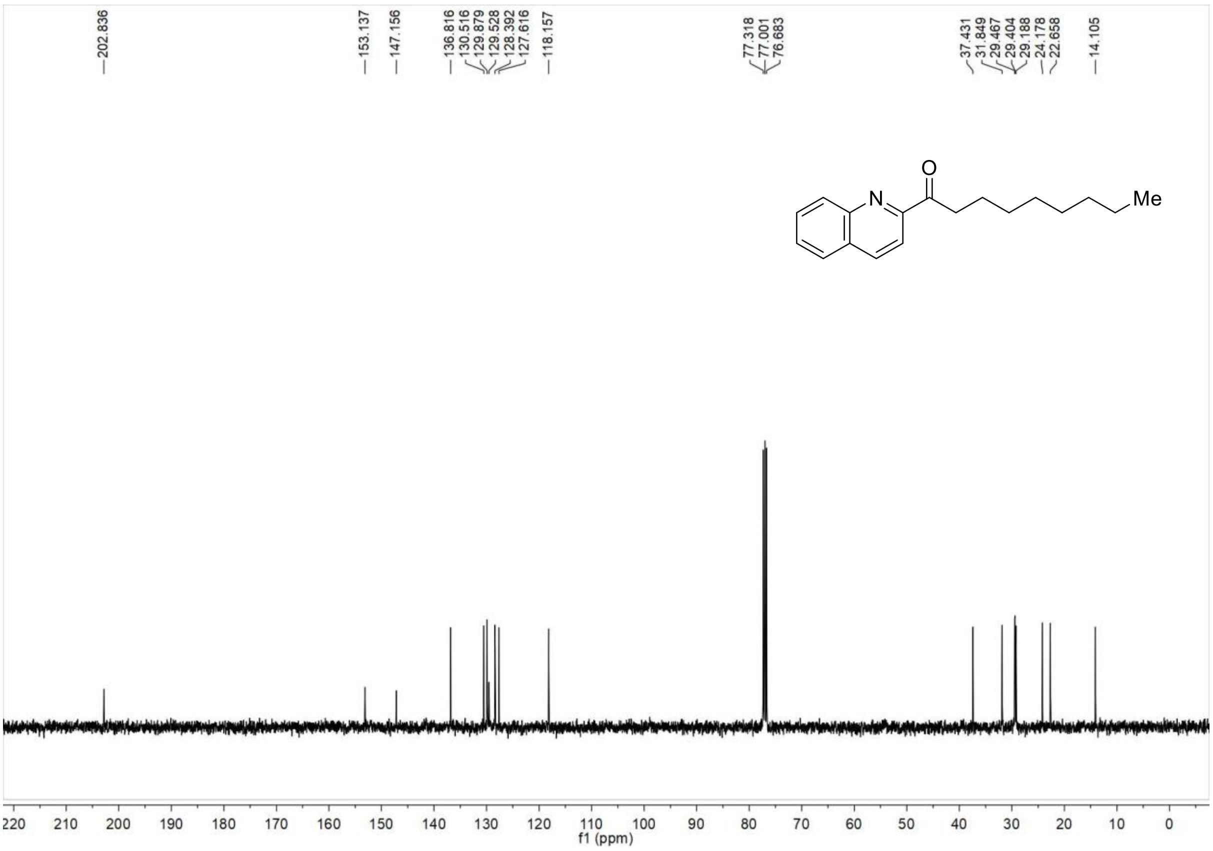

Supplementary Figure 96. ^{13}C-NMR of compound **12**, recorded at 400 MHz and 25 °C in $CDCl_3$

1-(3-methylquinolin-2-yl)Ethan-1-one (13)

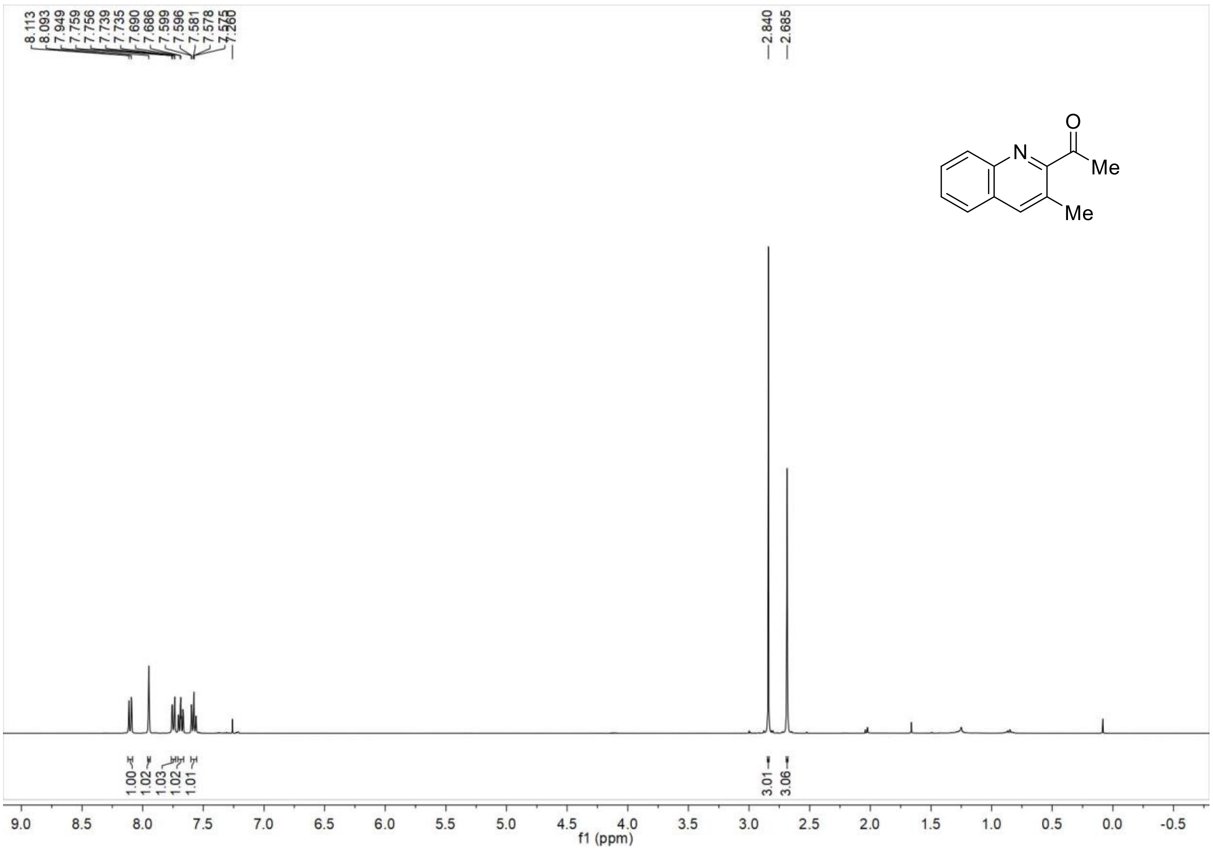

Supplementary Figure 97. ^{1}H-NMR of compound **13**, recorded at 400 MHz and 25 °C in $CDCl_3$

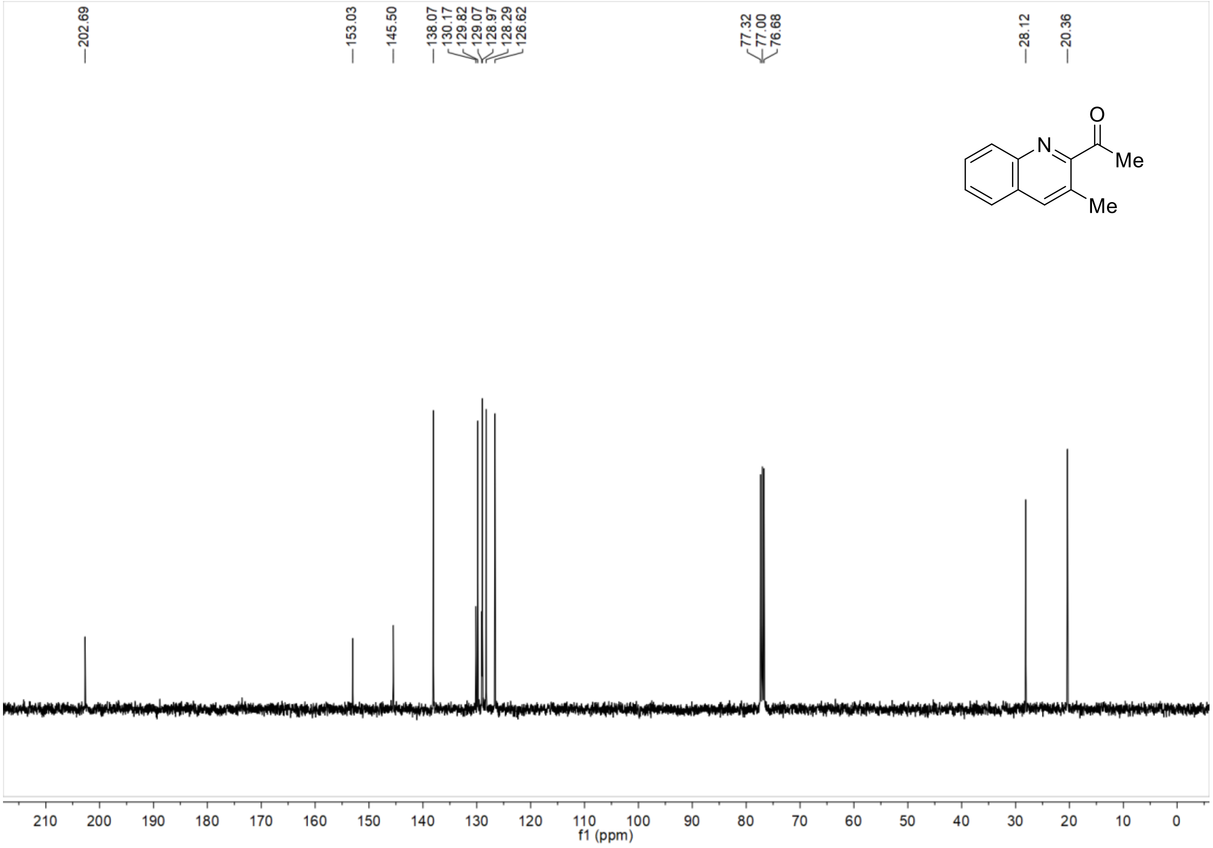

Supplementary Figure 98. ^{13}C-NMR of compound **13**, recorded at 400 MHz and 25 °C in $CDCl_3$

1-(3-phenylquinolin-2-yl)Ethan-1-one (14)

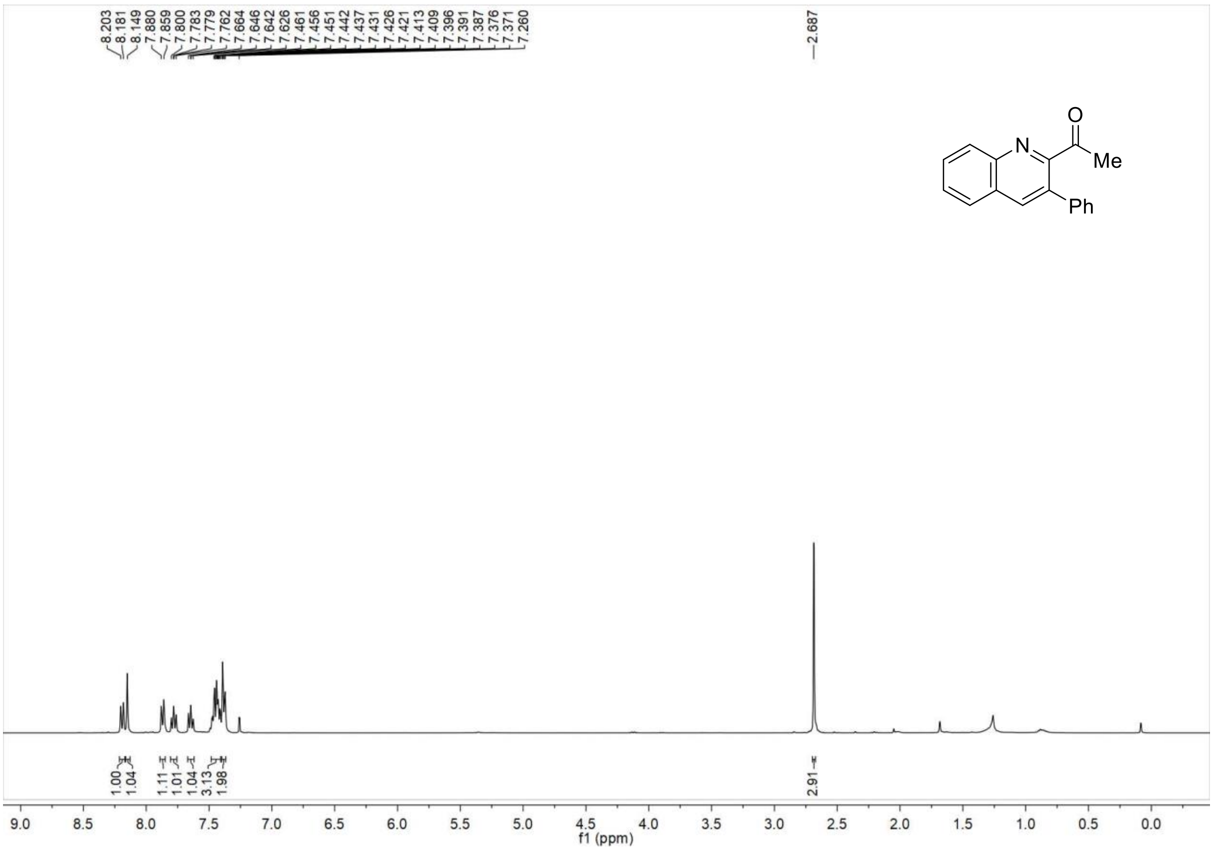

Supplementary Figure 99. ^{1}H-NMR of compound **14**, recorded at 400 MHz and 25 °C in $CDCl_3$

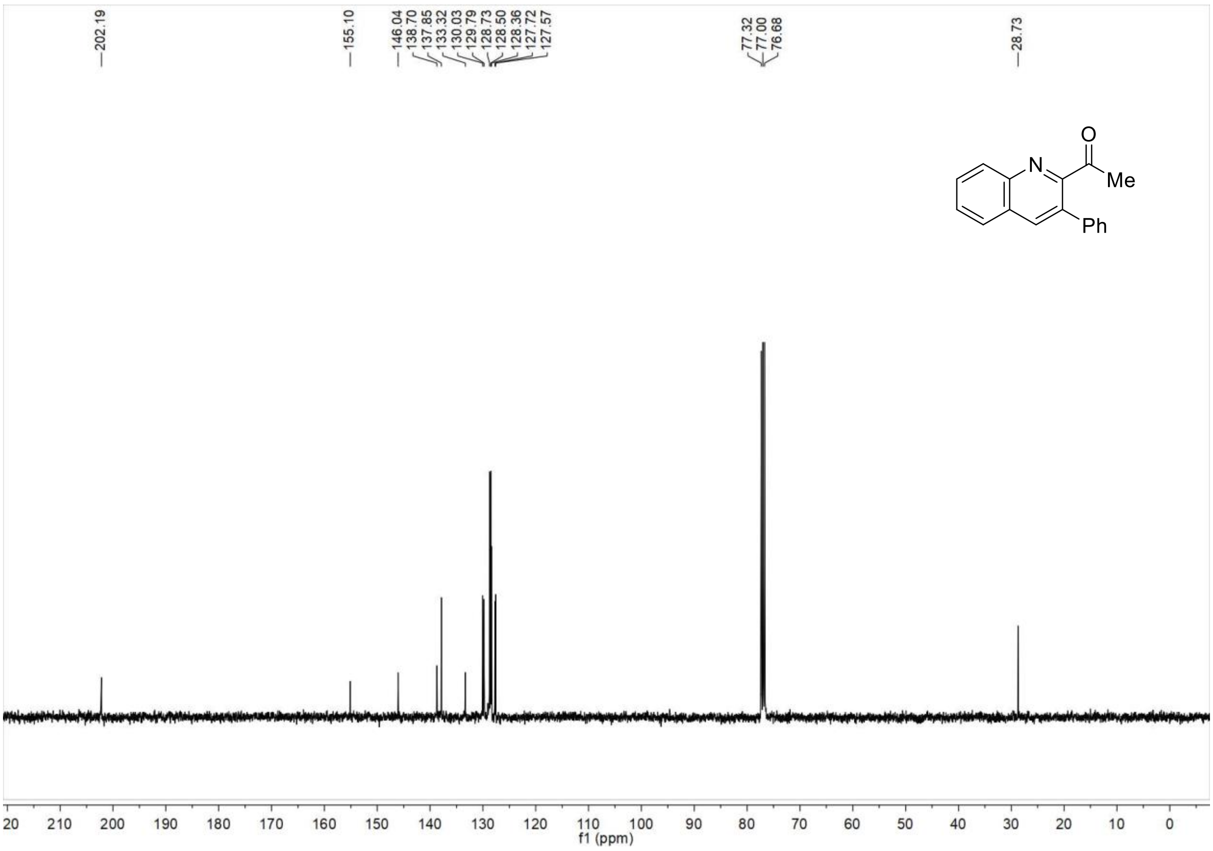

Supplementary Figure 100. ^{13}C-NMR of compound **14**, recorded at 400 MHz and 25 °C in $CDCl_3$

1-(3-benzylquinolin-2-yl)Ethan-1-one (15)

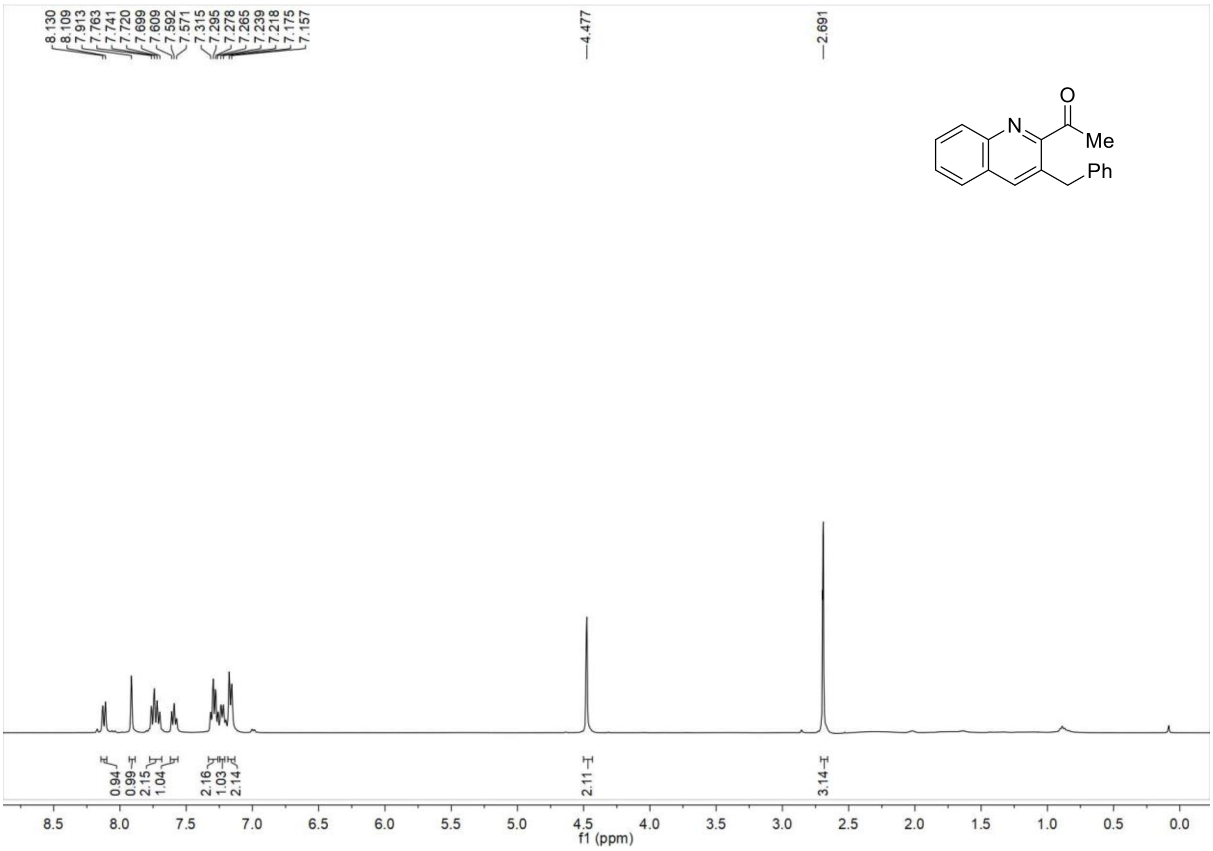

Supplementary Figure 101. ^{1}H-NMR of compound **15**, recorded at 400 MHz and 25 °C in $CDCl_3$

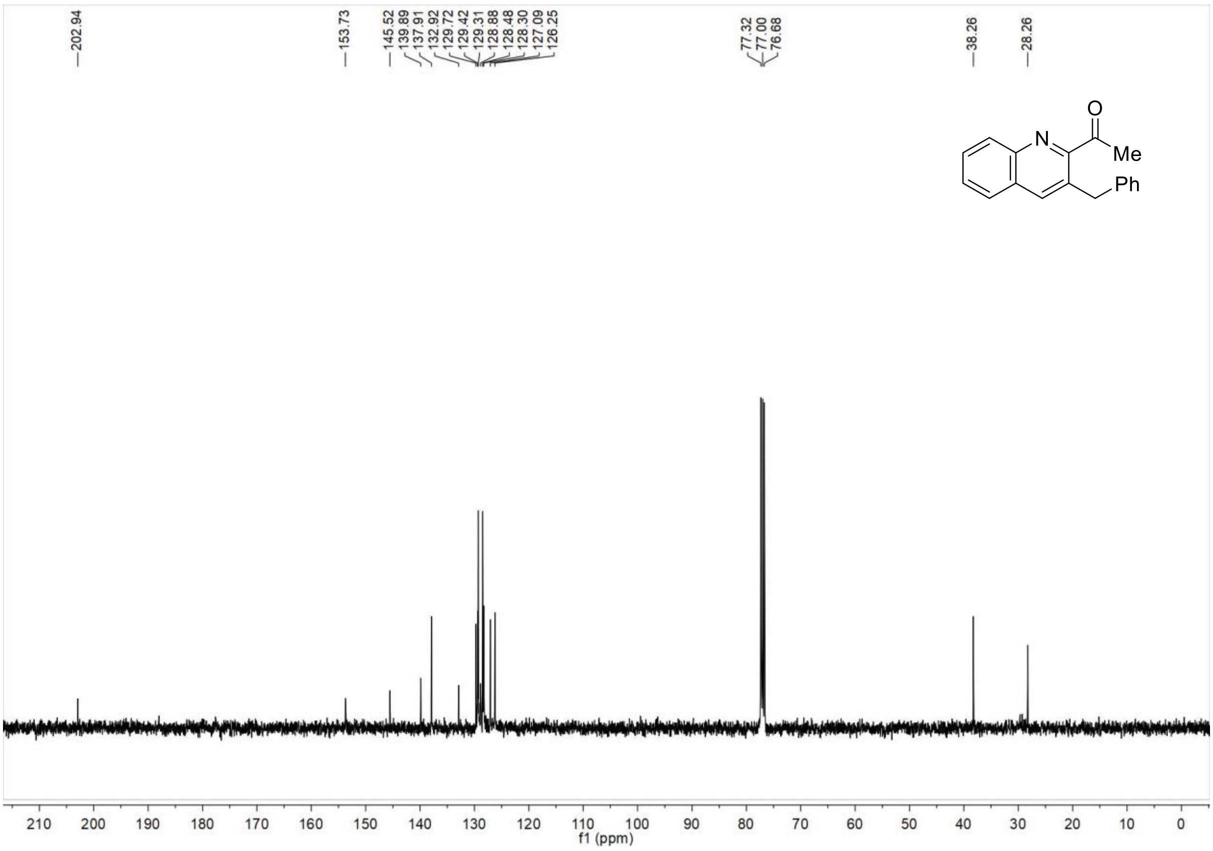

Supplementary Figure 102. ^{13}C-NMR of compound **15**, recorded at 400 MHz and 25 °C in $CDCl_3$

1-(3-(benzo[*d*][1,3]dioxol-5-yl)quinolin-2-yl)Ethan-1-one (16)

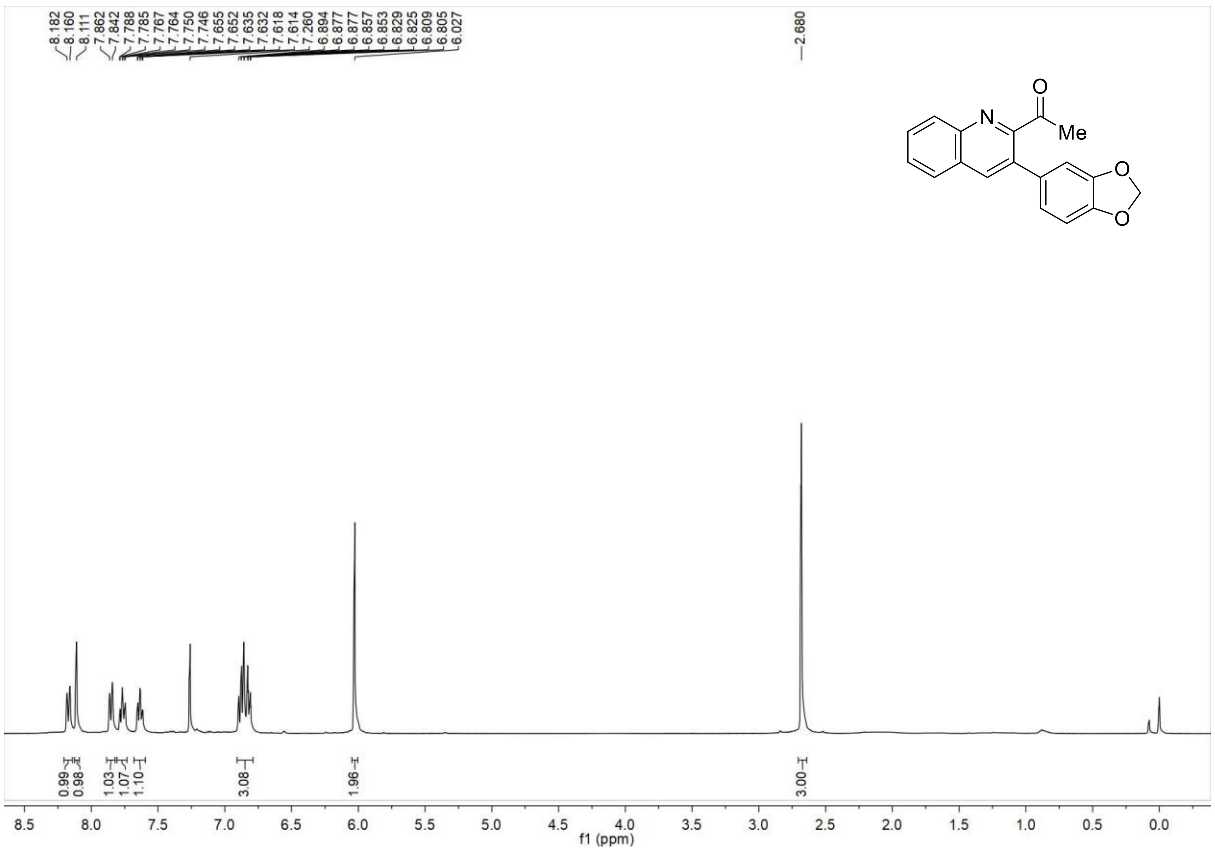

Supplementary Figure 103. ^{1}H-NMR of compound **16**, recorded at 400 MHz and 25 °C in $CDCl_3$

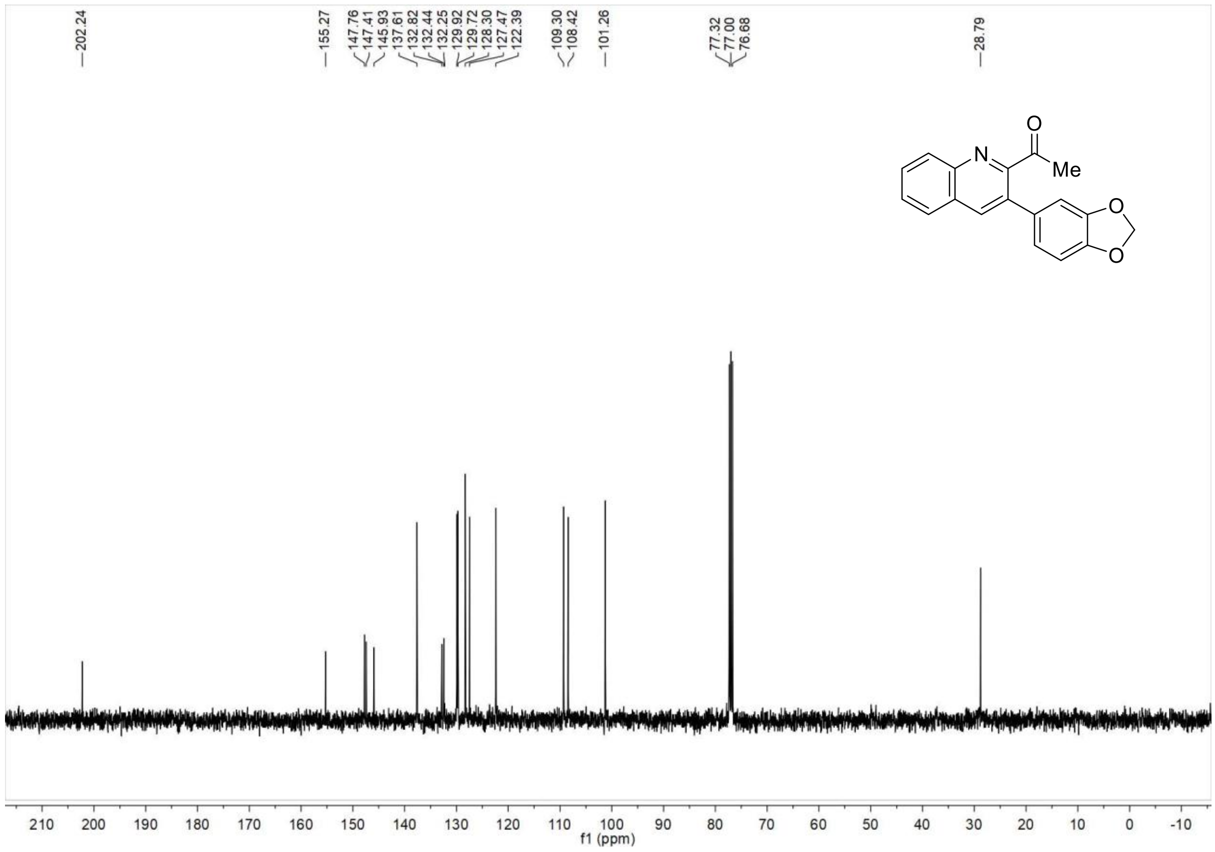

Supplementary Figure 104. ^{13}C-NMR of compound **16**, recorded at 400 MHz and 25 °C in $CDCl_3$

1-(3-bromoquinolin-2-yl)Ethan-1-one (17)

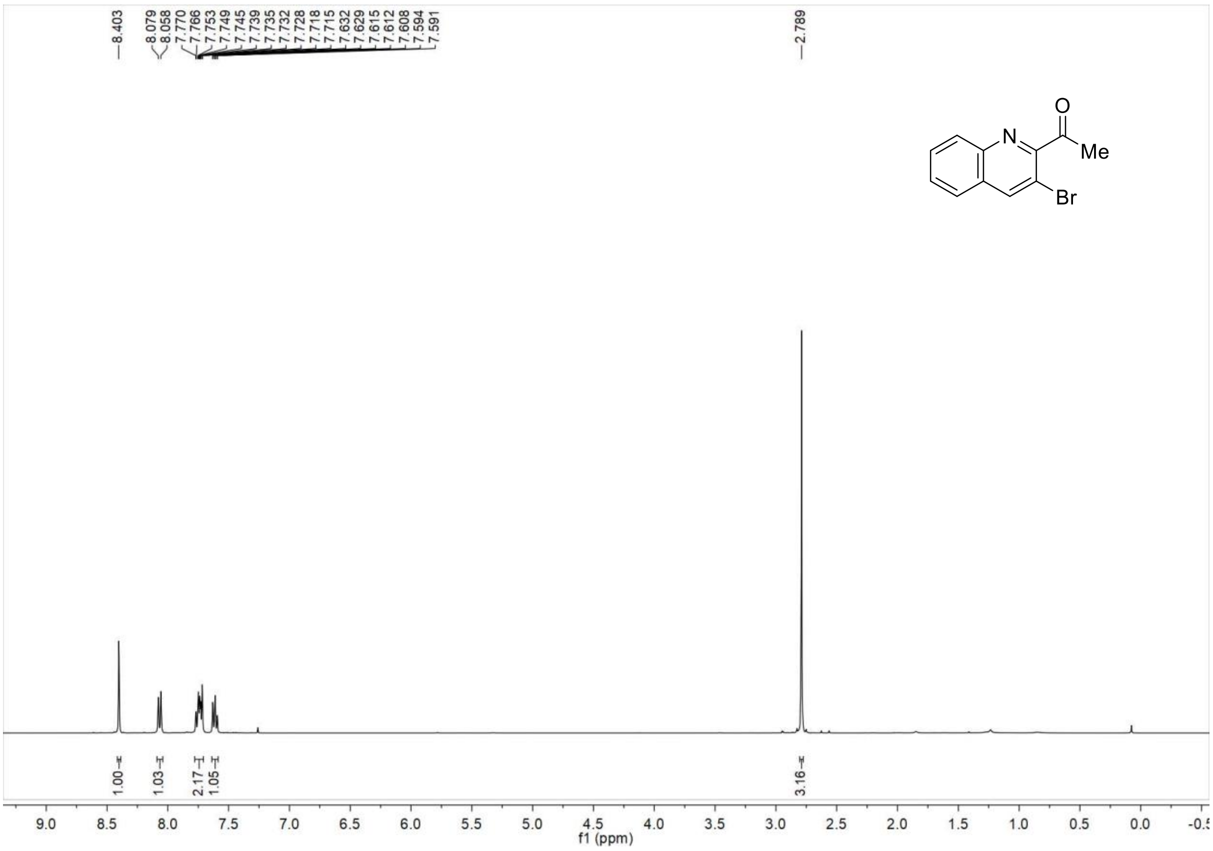

Supplementary Figure 105. ^{1}H-NMR of compound **17**, recorded at 400 MHz and 25 °C in $CDCl_3$

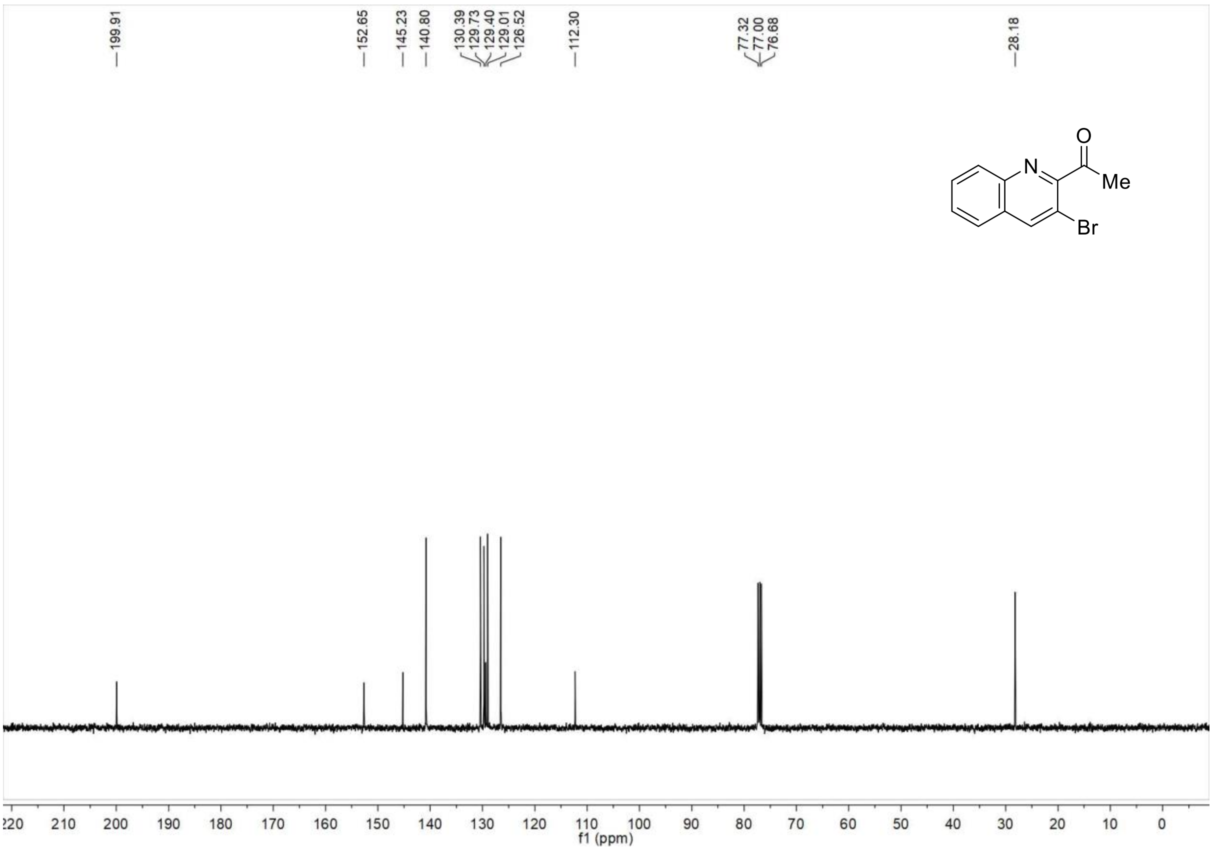

Supplementary Figure 106. ^{13}C-NMR of compound **17**, recorded at 400 MHz and 25 °C in $CDCl_3$

1-(3-chloroquinolin-2-yl)Ethan-1-one (18)

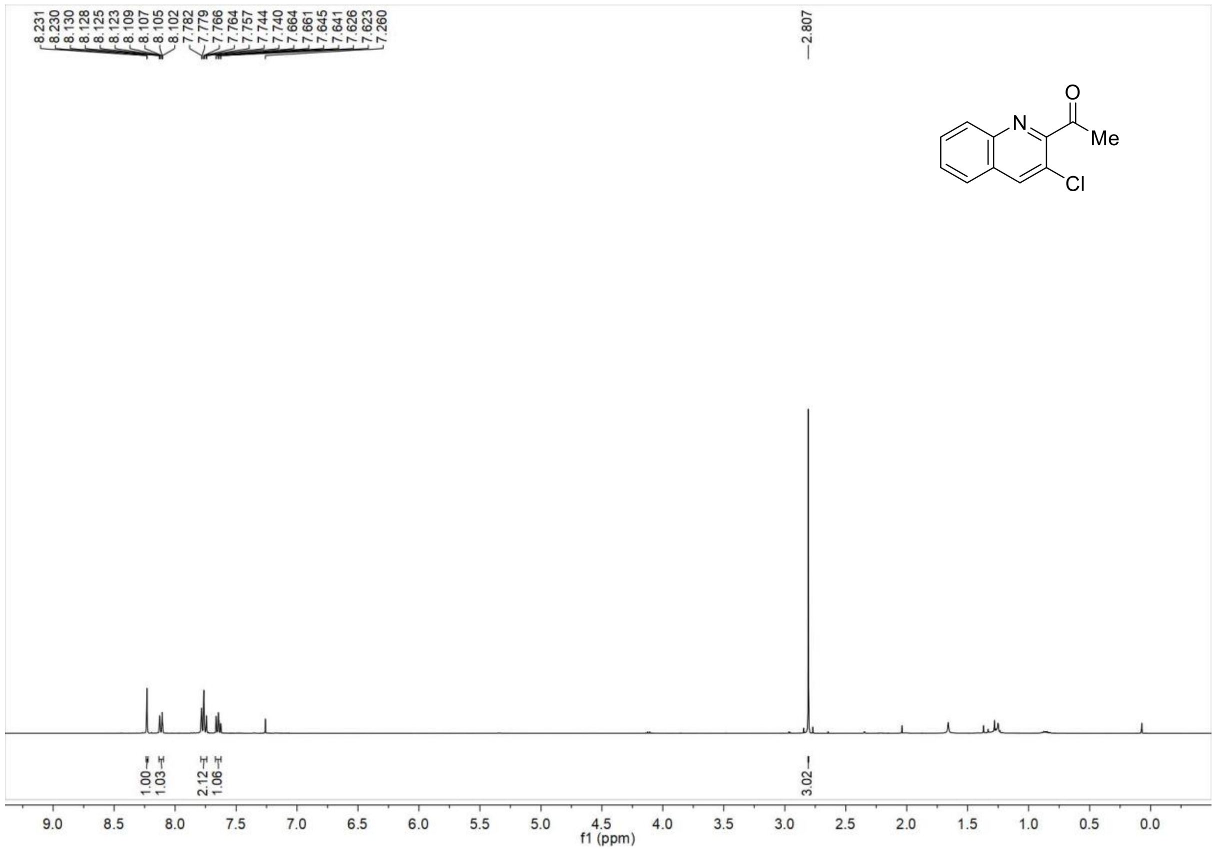

Supplementary Figure 107. ^{1}H-NMR of compound **18**, recorded at 400 MHz and 25 °C in $CDCl_3$

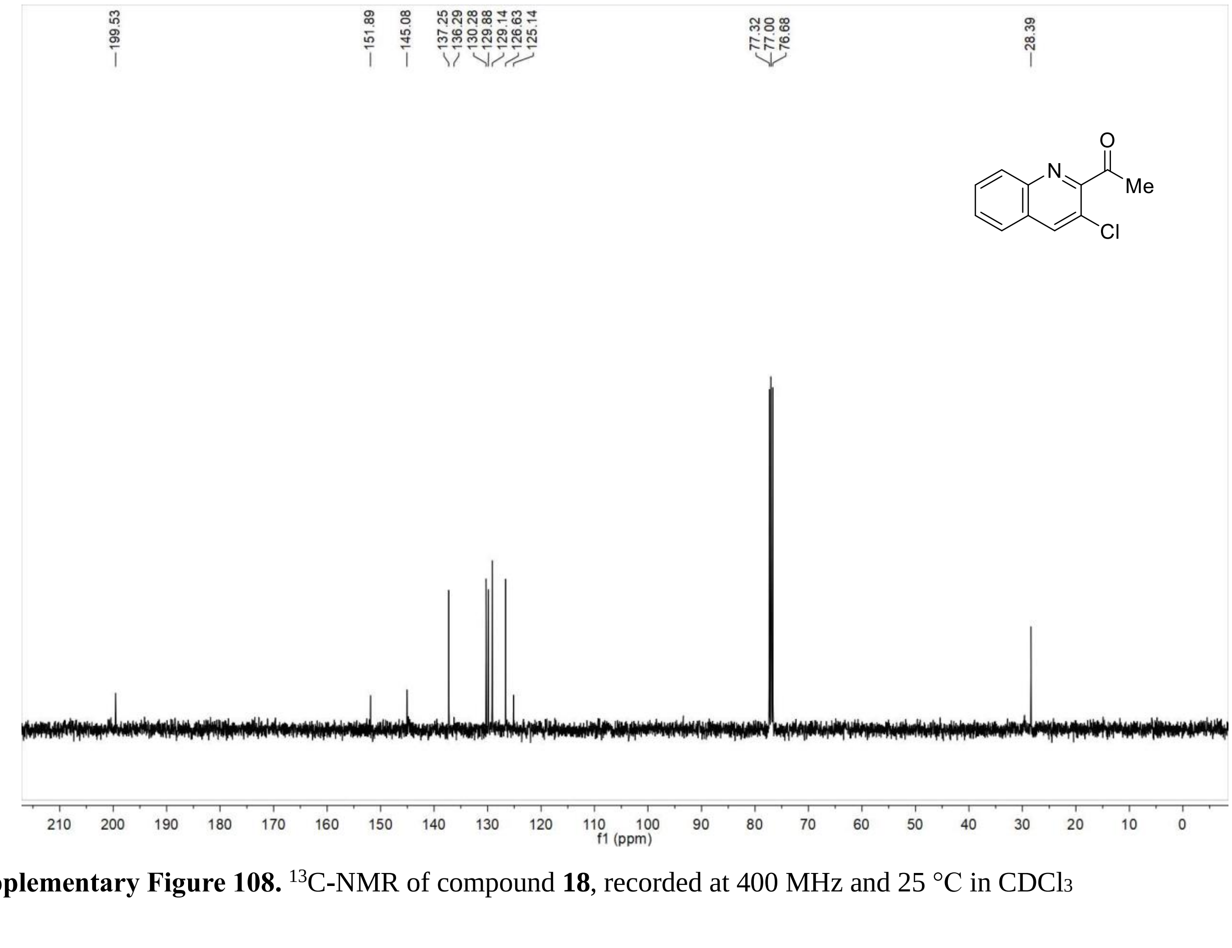

Supplementary Figure 108. ^{13}C-NMR of compound **18**, recorded at 400 MHz and 25 °C in $CDCl_3$

1-(3-(trimethylsilyl)quinolin-2-yl)Ethan-1-one (19)

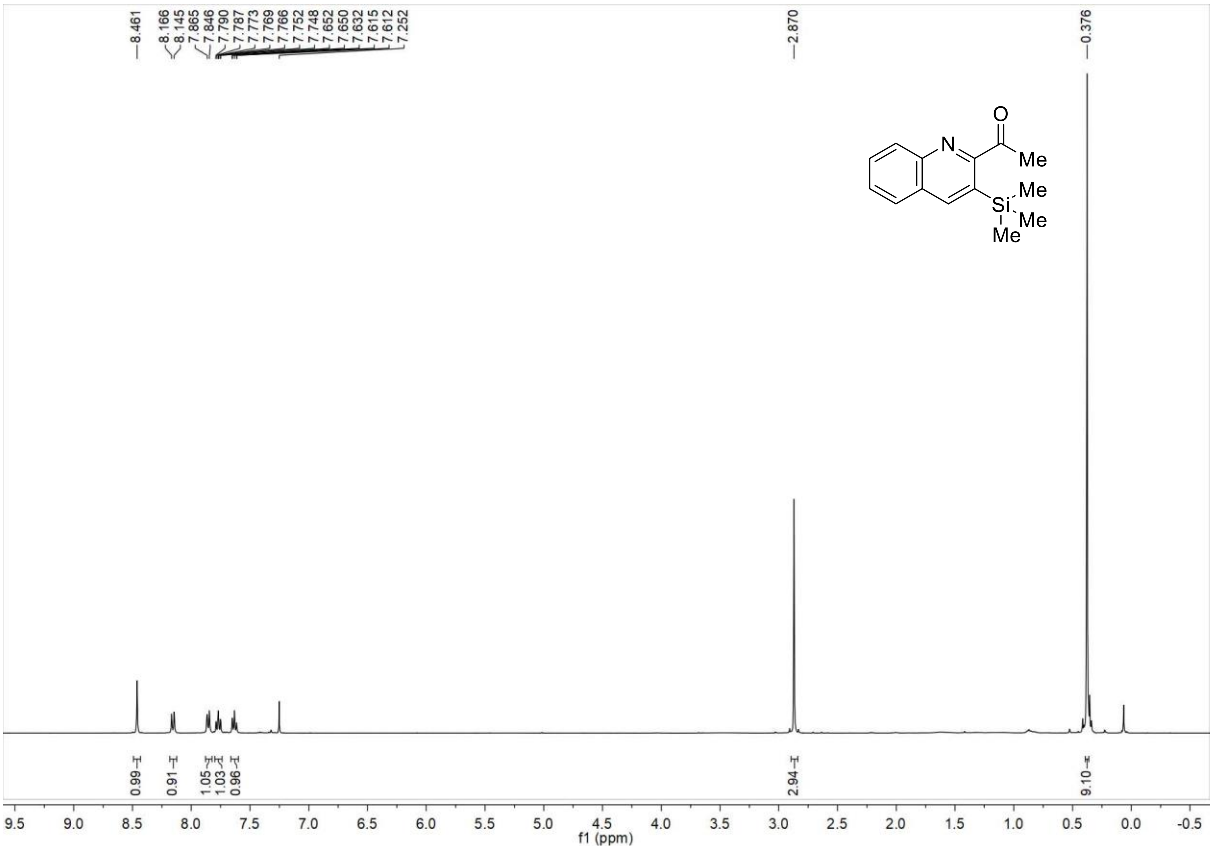

Supplementary Figure 109. ^{1}H-NMR of compound **19**, recorded at 400 MHz and 25 °C in $CDCl_3$

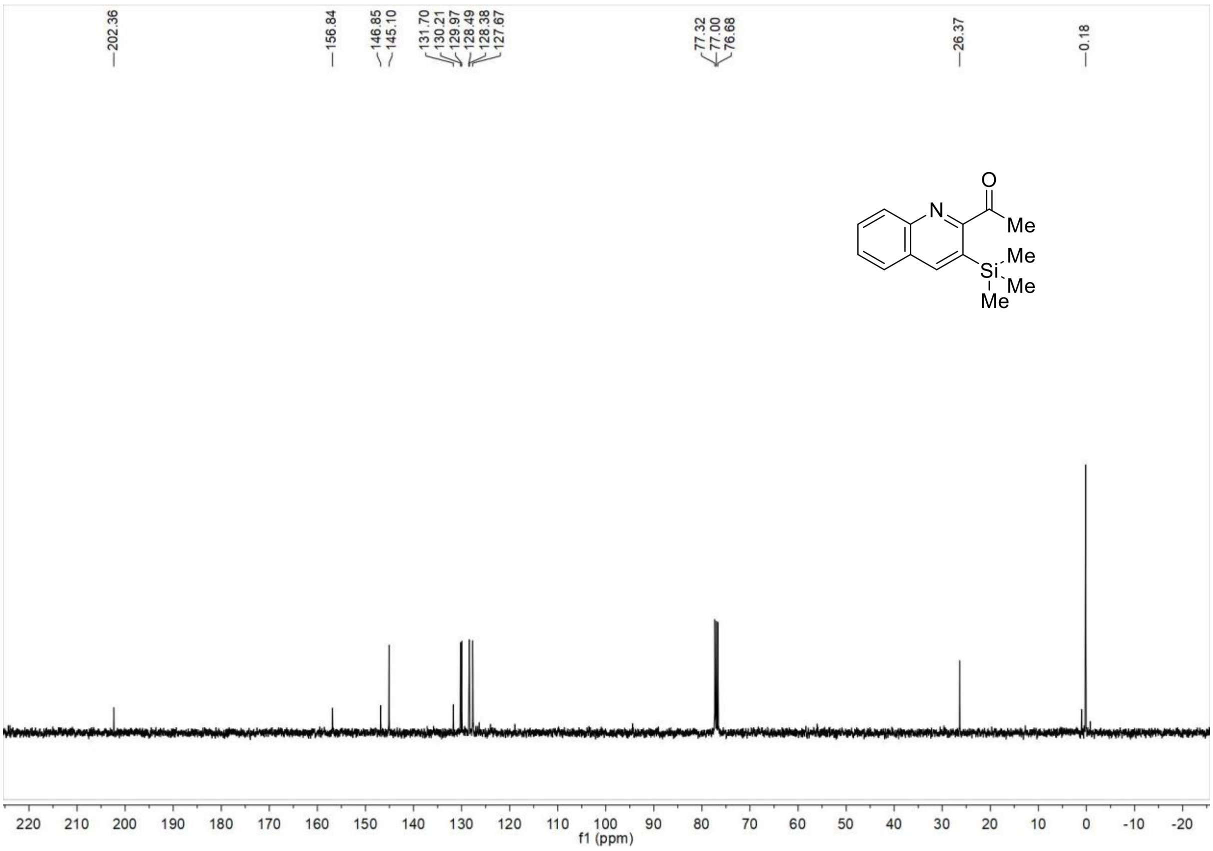

Supplementary Figure 110. ^{13}C-NMR of compound **19**, recorded at 400 MHz and 25 °C in $CDCl_3$

1-(3-allylquinolin-2-yl)Ethan-1-one (20)

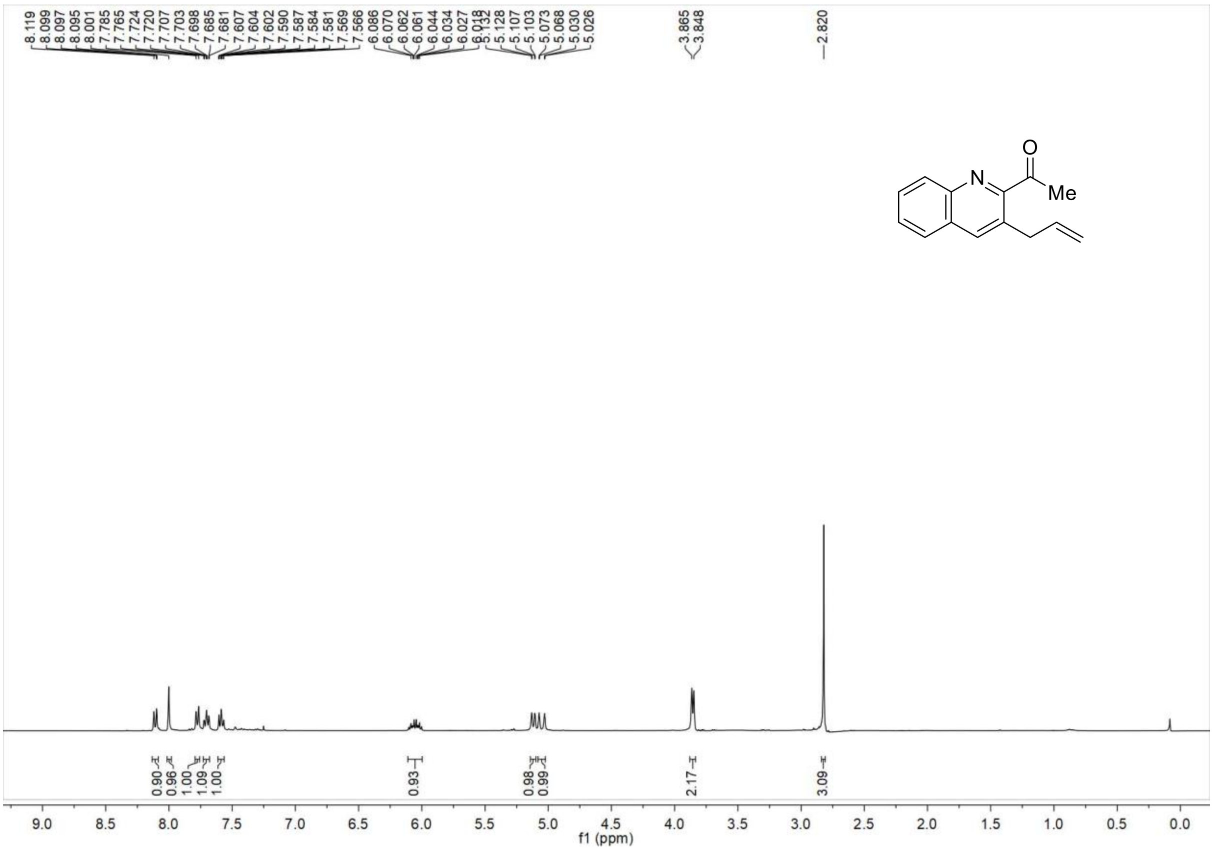

Supplementary Figure 111. ^{1}H-NMR of compound **20**, recorded at 400 MHz and 25 °C in $CDCl_3$

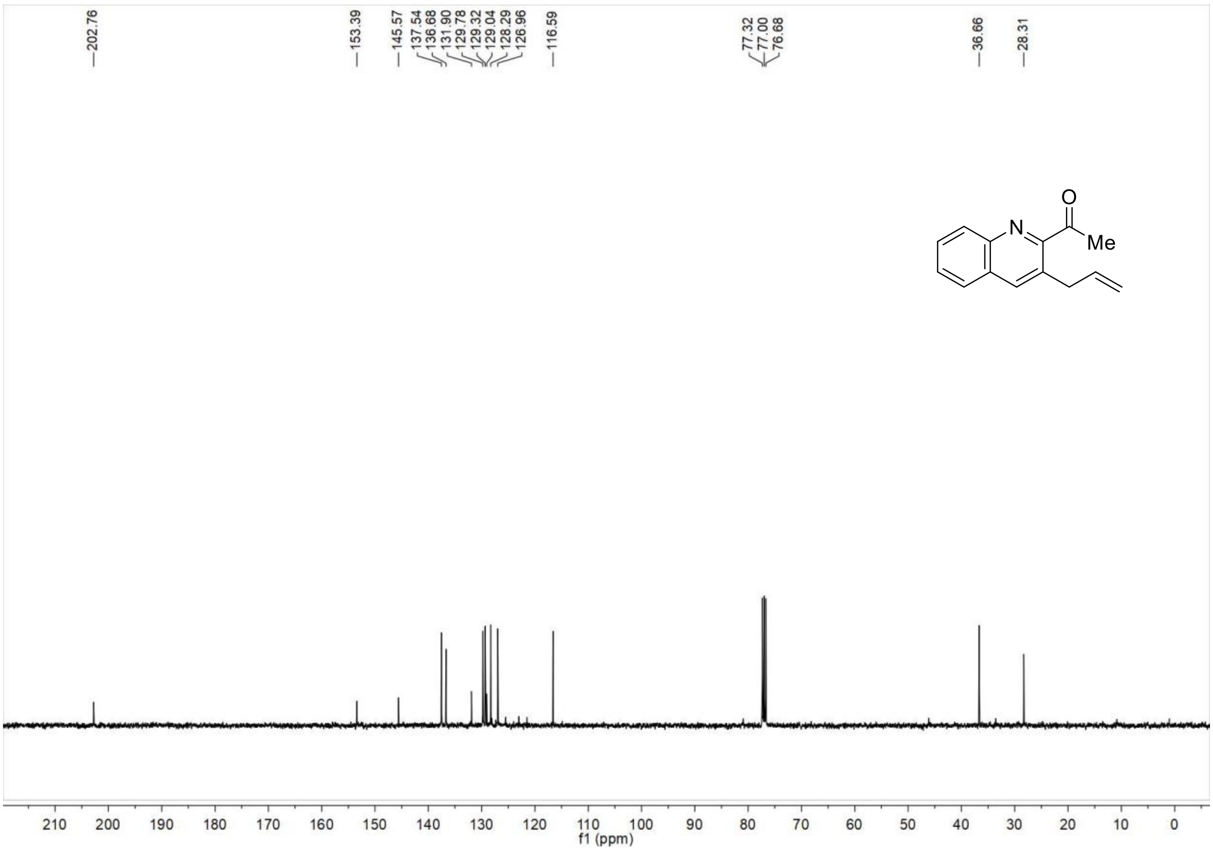

Supplementary Figure 112. ^{13}C-NMR of compound **20**, recorded at 400 MHz and 25 °C in $CDCl_3$

1-(3-(phenylethynyl)quinolin-2-yl)Ethan-1-one (21)

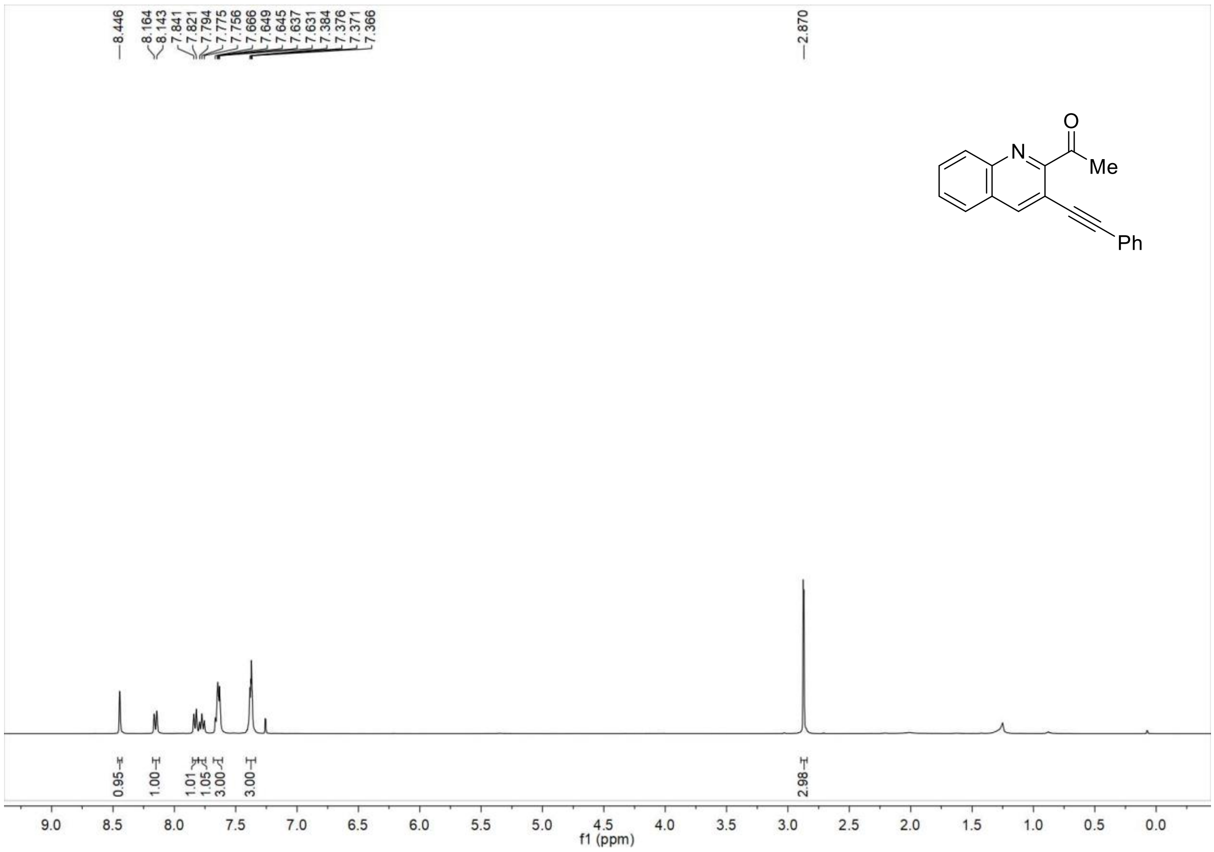

Supplementary Figure 113. ^{1}H-NMR of compound **21** recorded at 400 MHz and 25 °C in $CDCl_3$

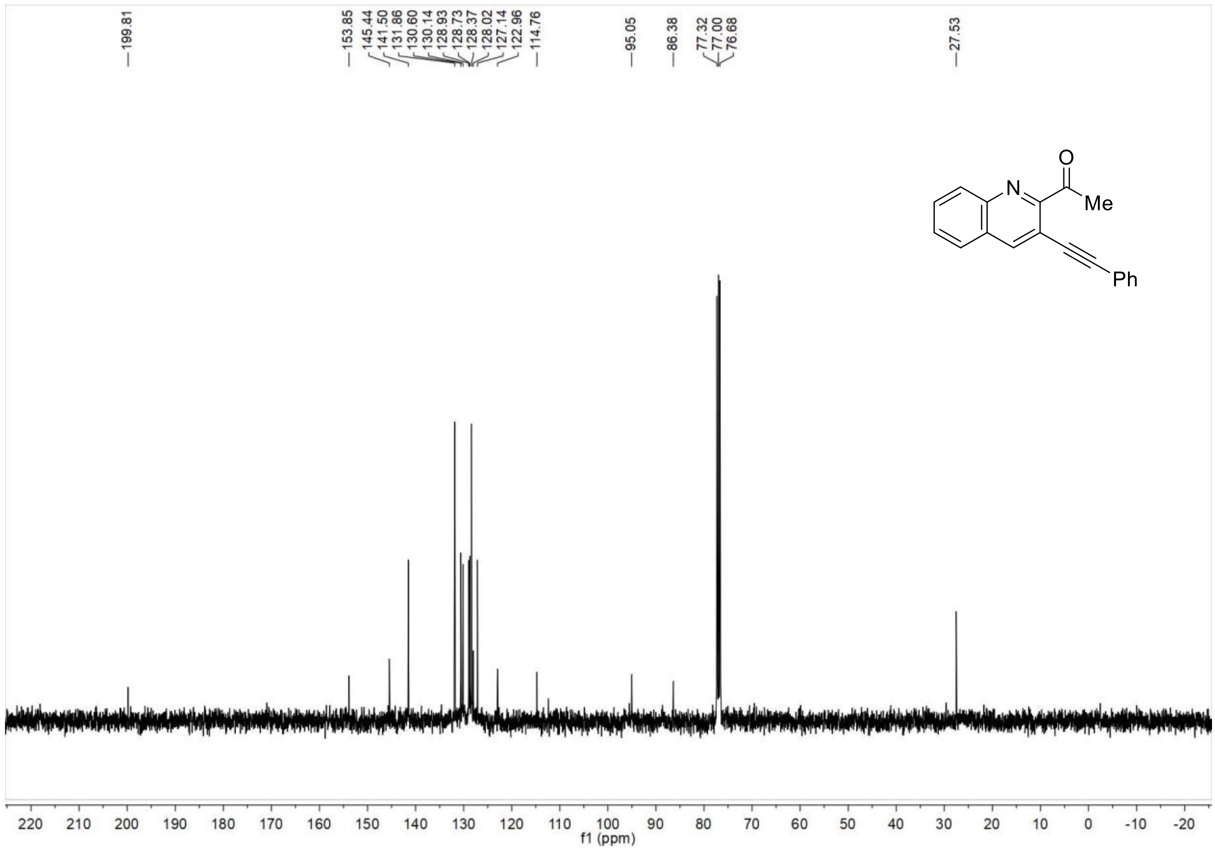

Supplementary Figure 114. ^{13}C-NMR of compound **21**, recorded at 400 MHz and 25 °C in $CDCl_3$

1-(2,3-dihydro-1*H*-cyclopenta[*c*]quinolin-4-yl)Propan-1-one (22)

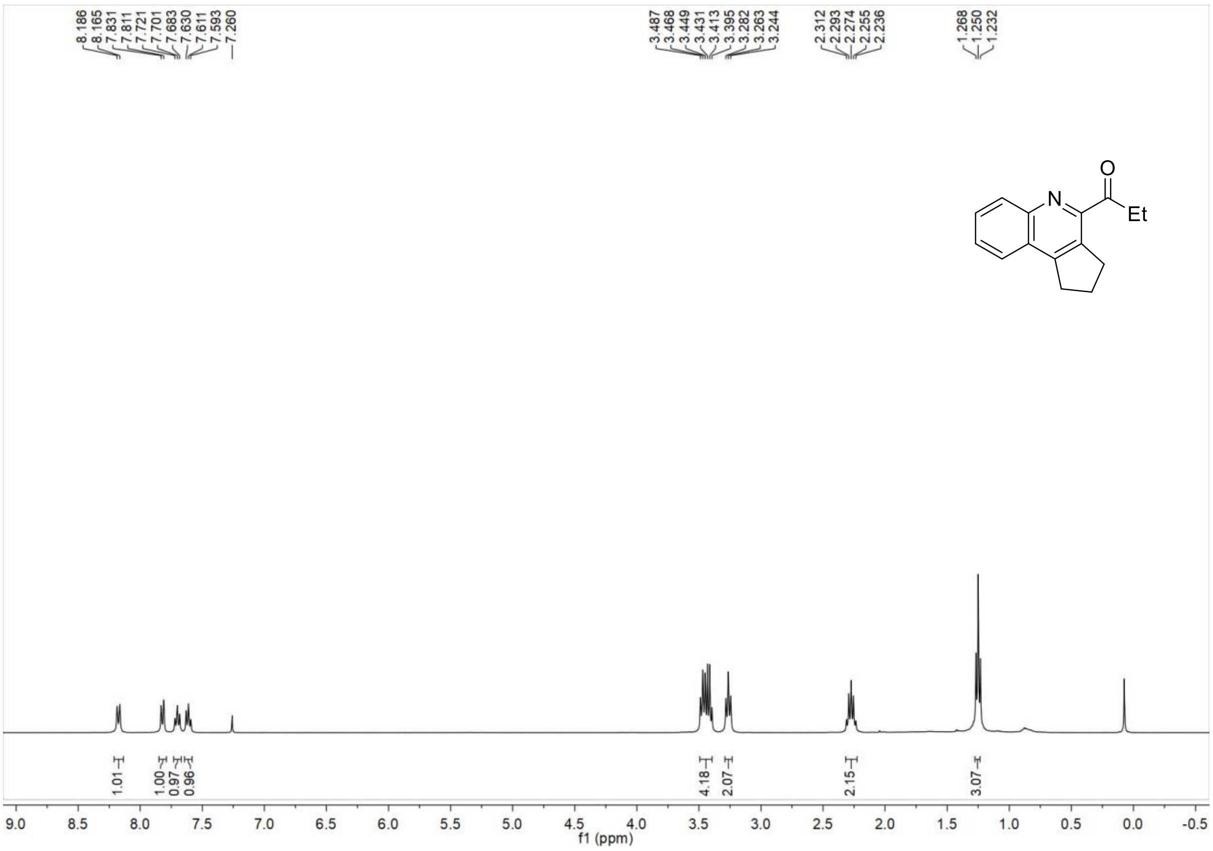

Supplementary Figure 115. ^{1}H-NMR of compound **22** recorded at 400 MHz and 25 °C in $CDCl_3$

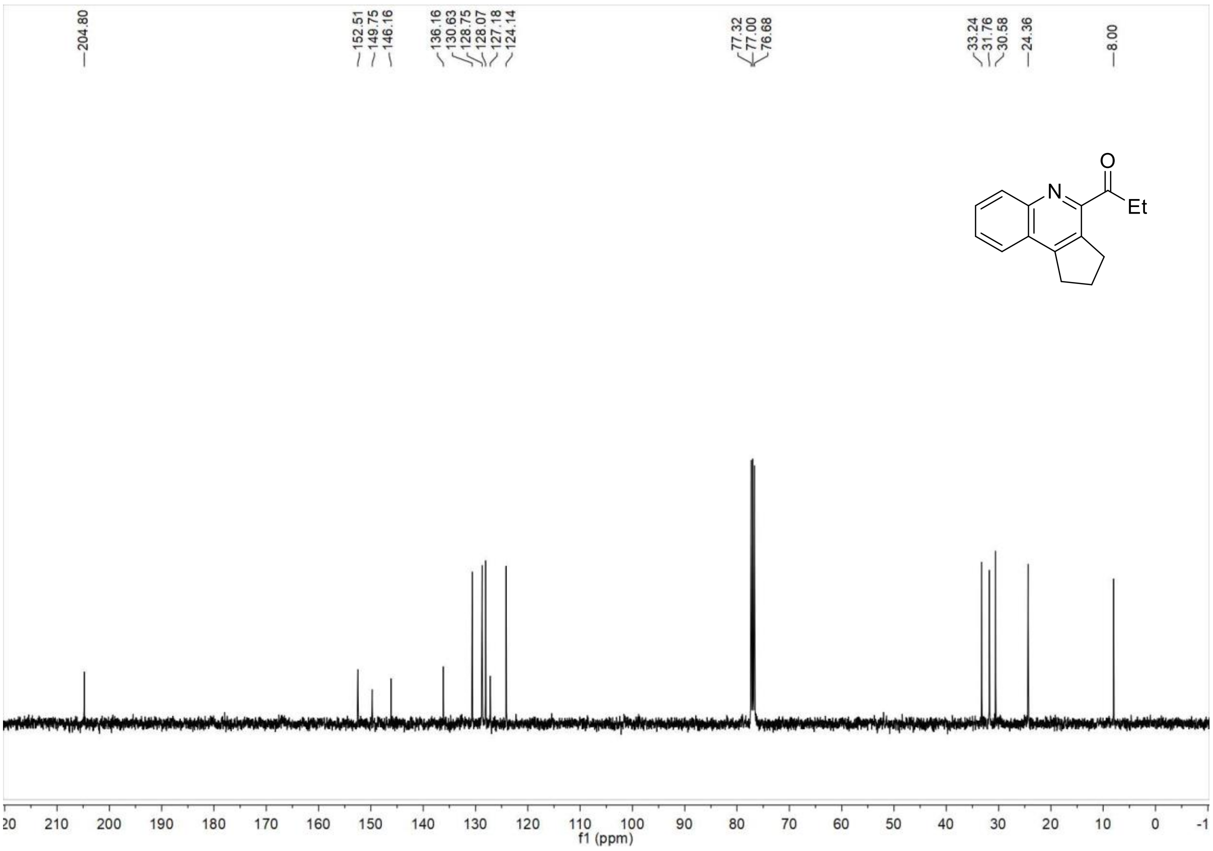

Supplementary Figure 116. ^{13}C-NMR of compound **22**, recorded at 400 MHz and 25 °C in $CDCl_3$

1-(7,8,9,10-tetrahydrophenanthridin-6-yl)Ethan-1-one (23)

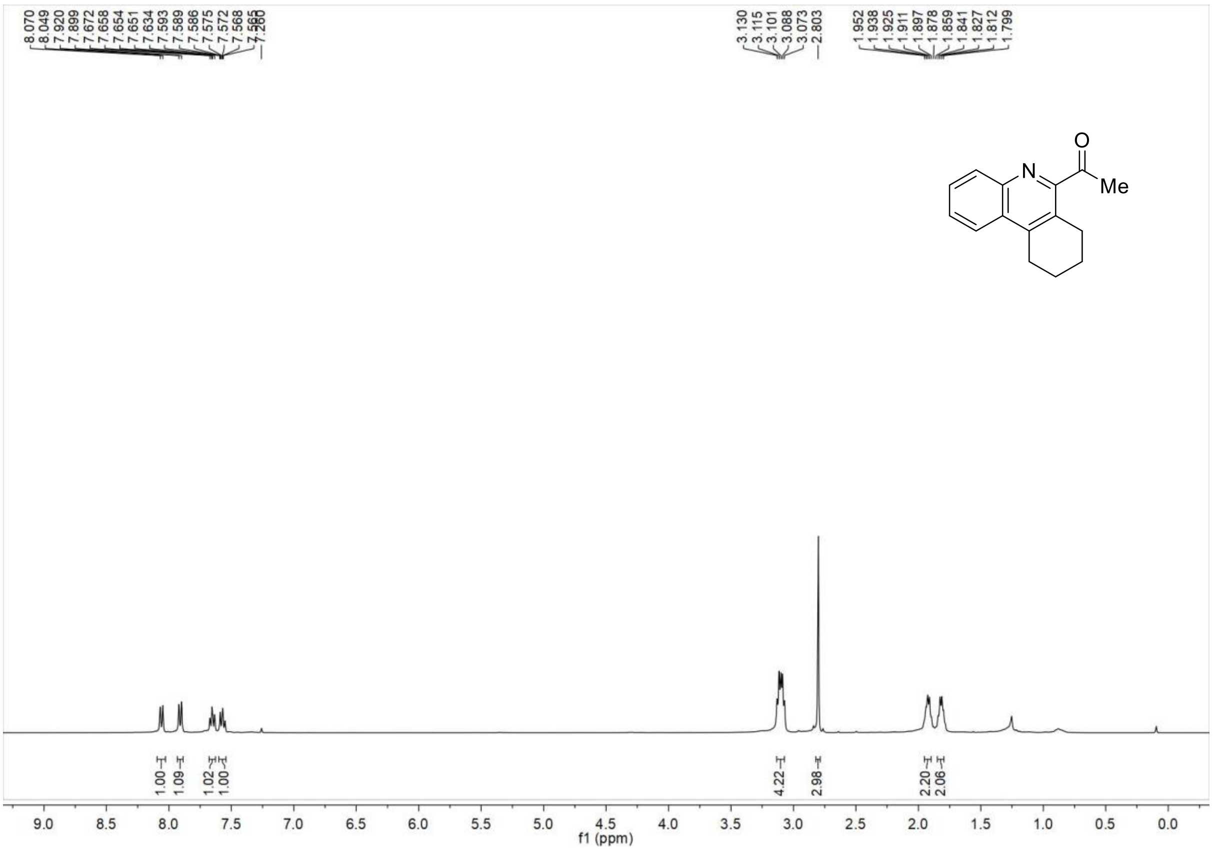

Supplementary Figure 117. ^{1}H-NMR of compound **23** recorded at 400 MHz and 25 °C in $CDCl_3$

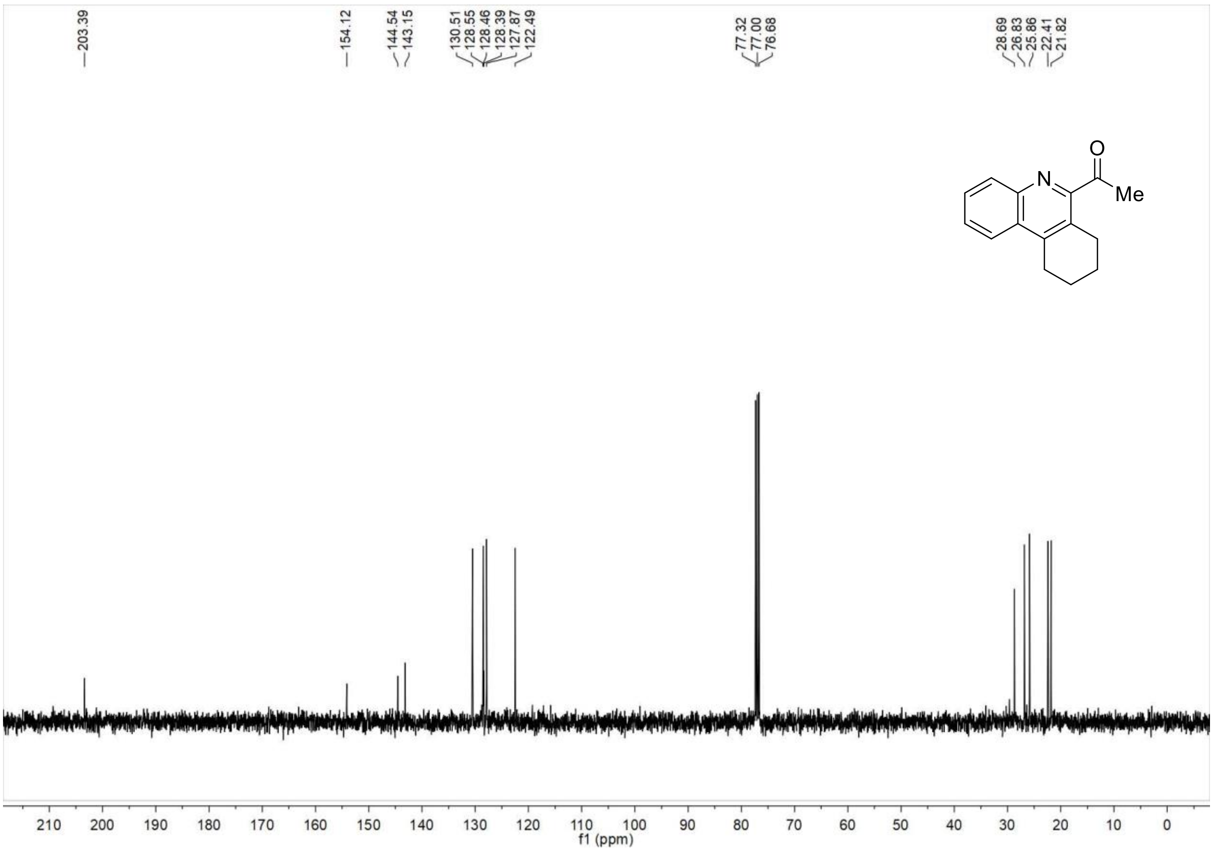

Supplementary Figure 118. ^{13}C-NMR of compound **23**, recorded at 400 MHz and 25 °C in $CDCl_3$

1-(8,9,10,11-tetrahydro-7*H*-cyclohepta[*c*]quinolin-6-yl)Ethan-1-one (24)

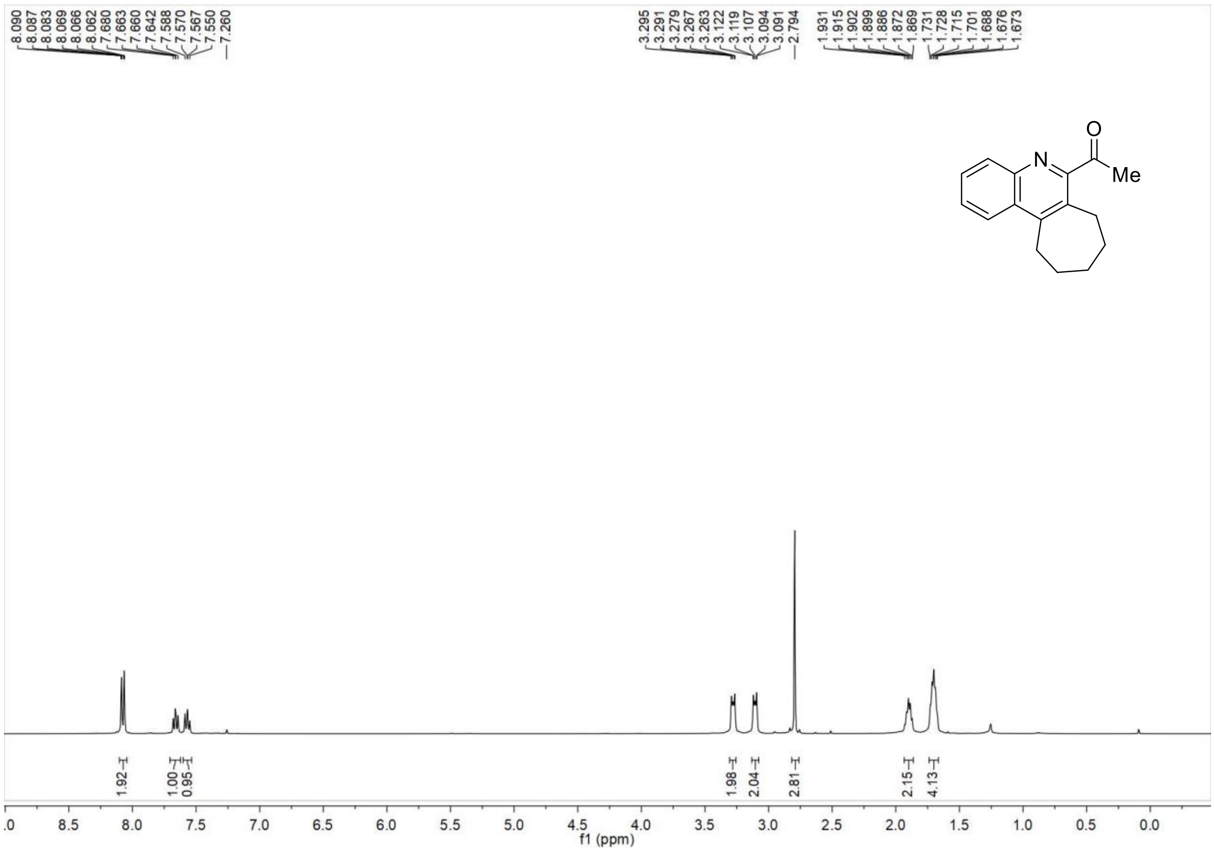

Supplementary Figure 119. ^{1}H-NMR of compound **24** recorded at 400 MHz and 25 °C in $CDCl_3$

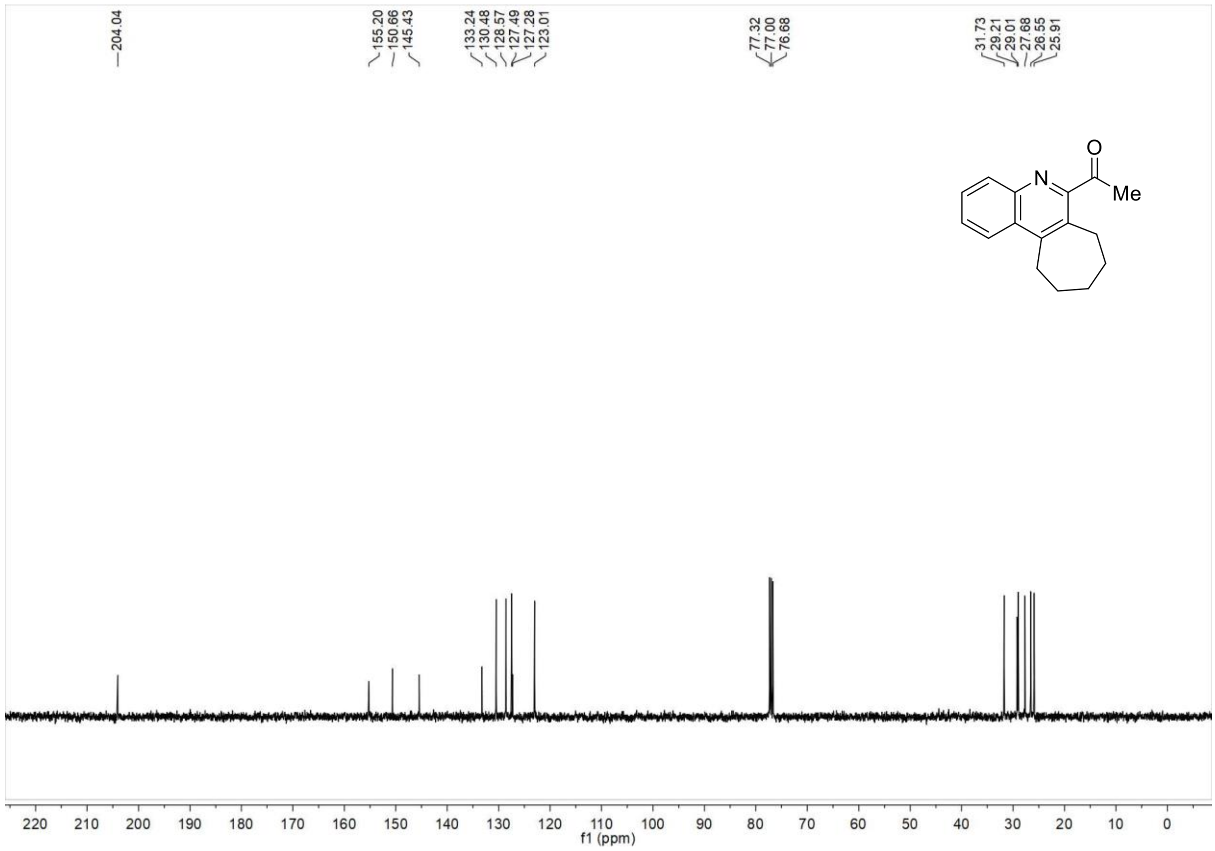

Supplementary Figure 120. ^{13}C-NMR of compound **24**, recorded at 400 MHz and 25 °C in $CDCl_3$

1-(10,11,11-trimethyl-7,8,9,10-tetrahydro-7,10-methanophenanthridin-6-yl)Ethan-1-one (25)

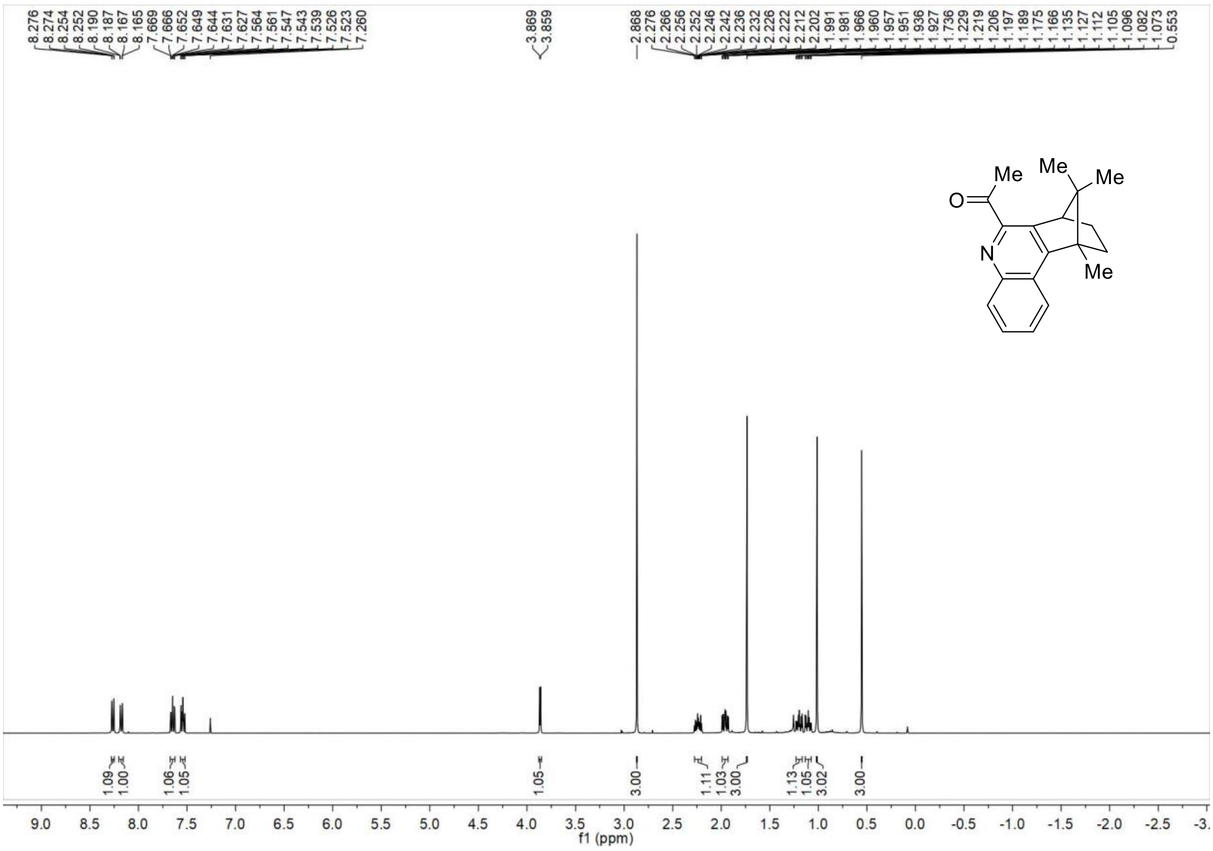

Supplementary Figure 121. ^{1}H-NMR of compound **25** recorded at 400 MHz and 25 °C in $CDCl_3$

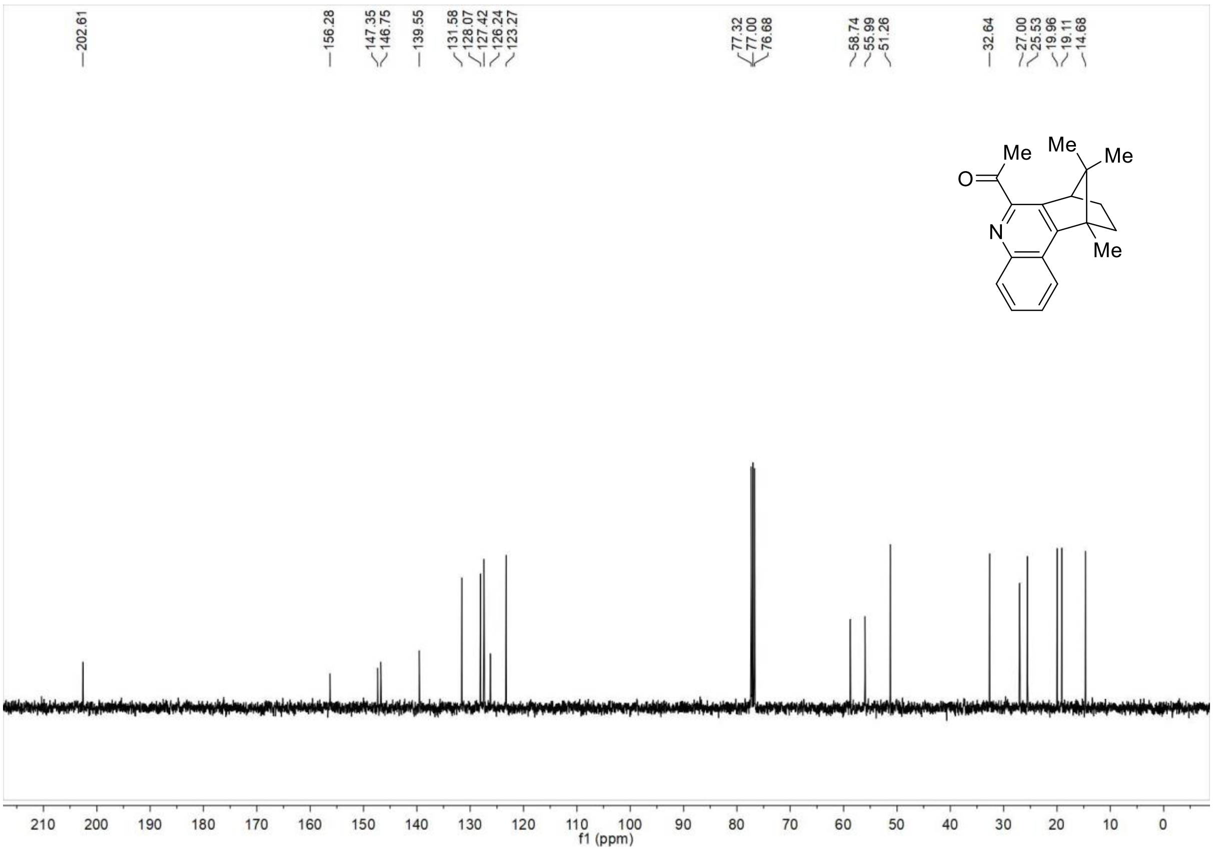

Supplementary Figure 122. ^{13}C-NMR of compound **25**, recorded at 400 MHz and 25 °C in $CDCl_3$

1-(3-(benzofuran-2-yl)quinolin-2-yl)Ethan-1-one (26)

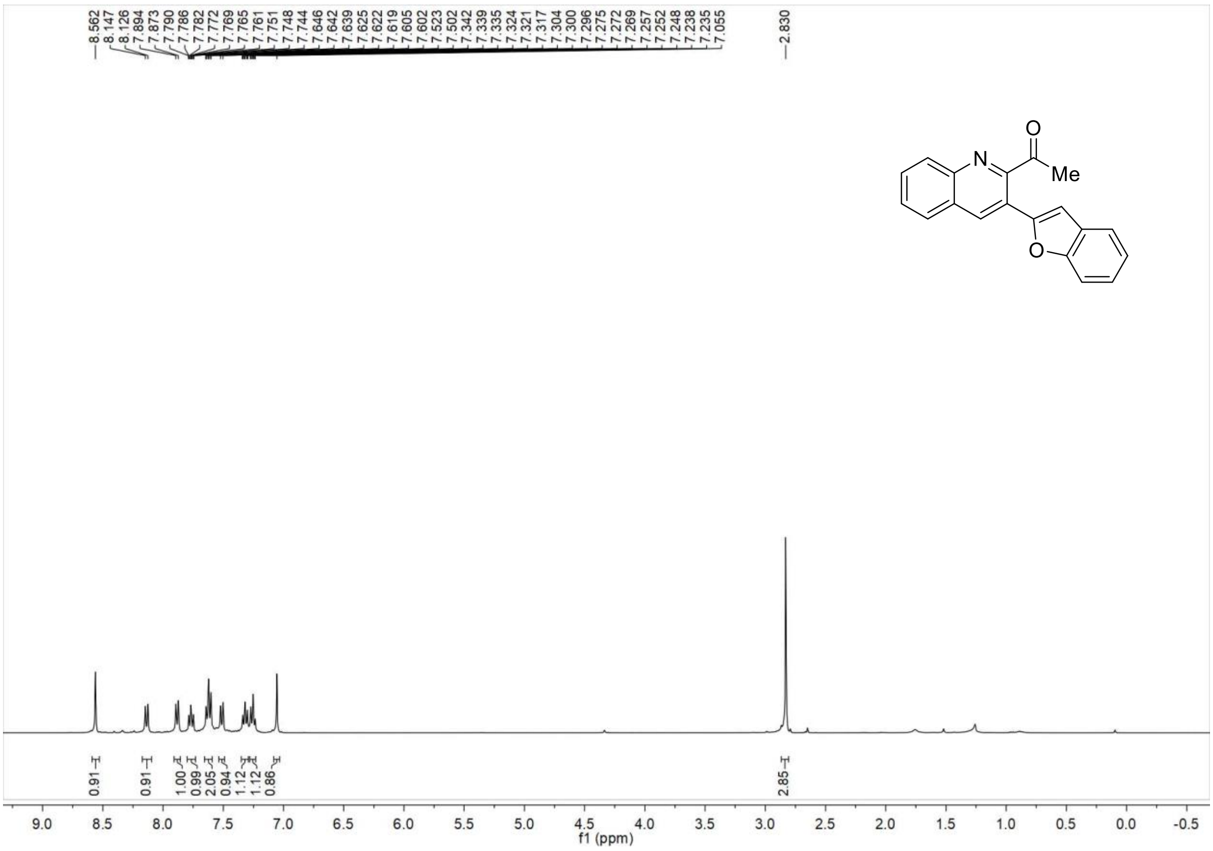

Supplementary Figure 123. ^{1}H-NMR of compound **26** recorded at 400 MHz and 25 °C in $CDCl_3$

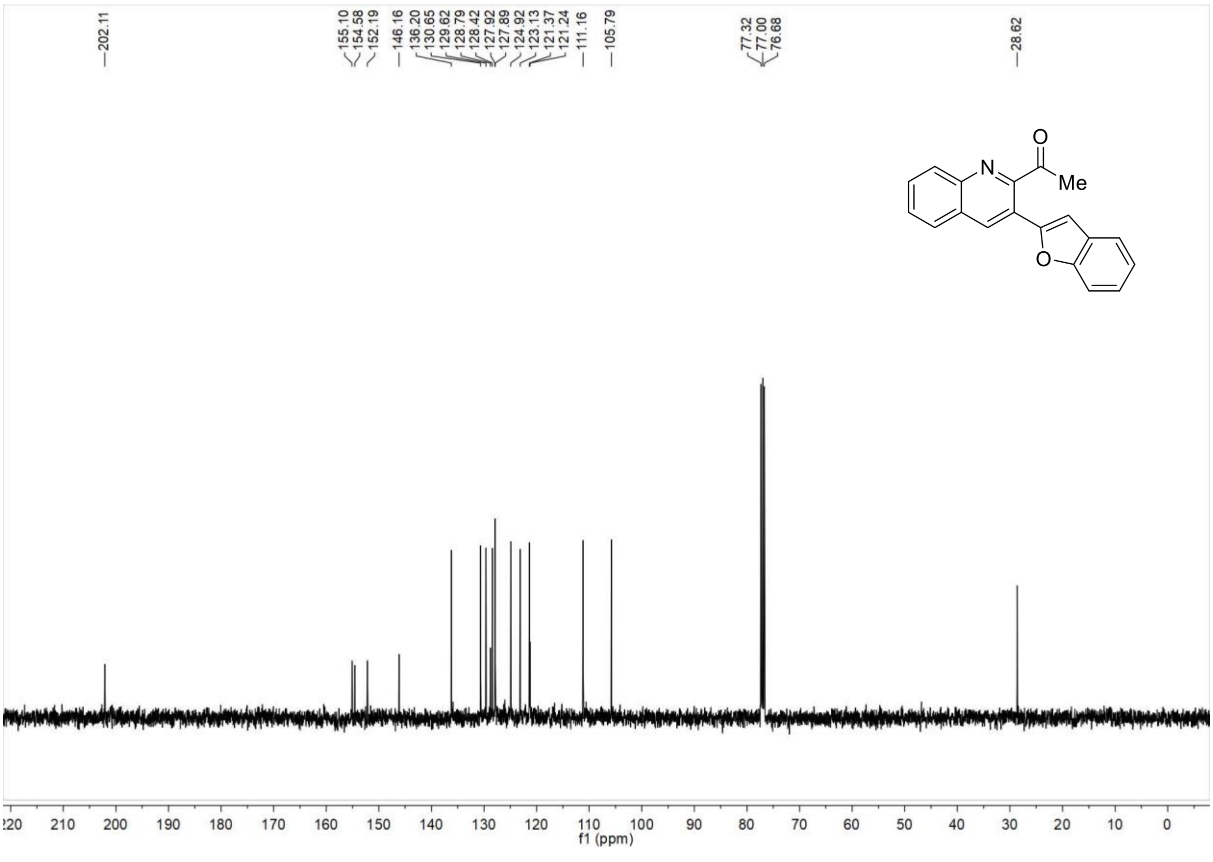

Supplementary Figure 124. ^{13}C-NMR of compound **26**, recorded at 400 MHz and 25 °C in $CDCl_3$

1-(6-(furan-3-yl)quinolin-2-yl)Ethan-1-one (27)

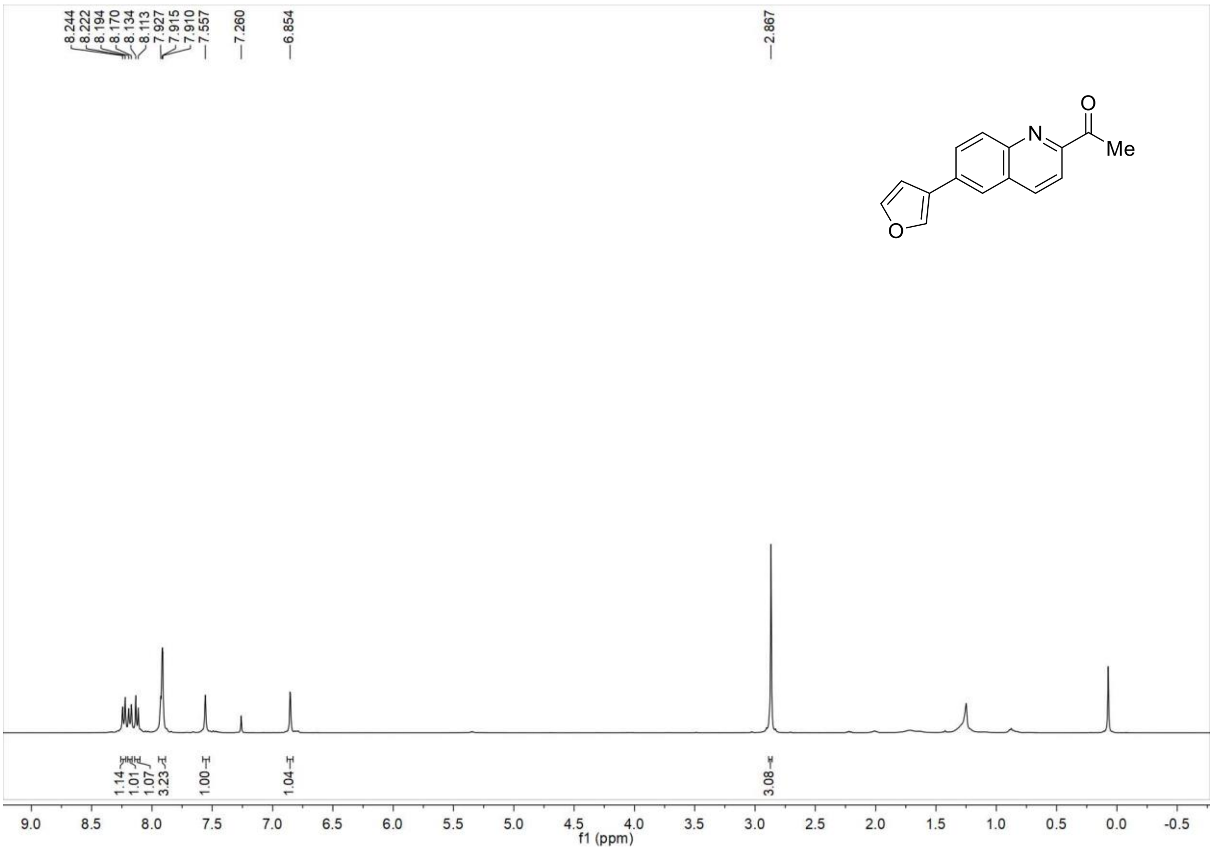

Supplementary Figure 125. ^{1}H-NMR of compound **27** recorded at 400 MHz and 25 °C in $CDCl_3$

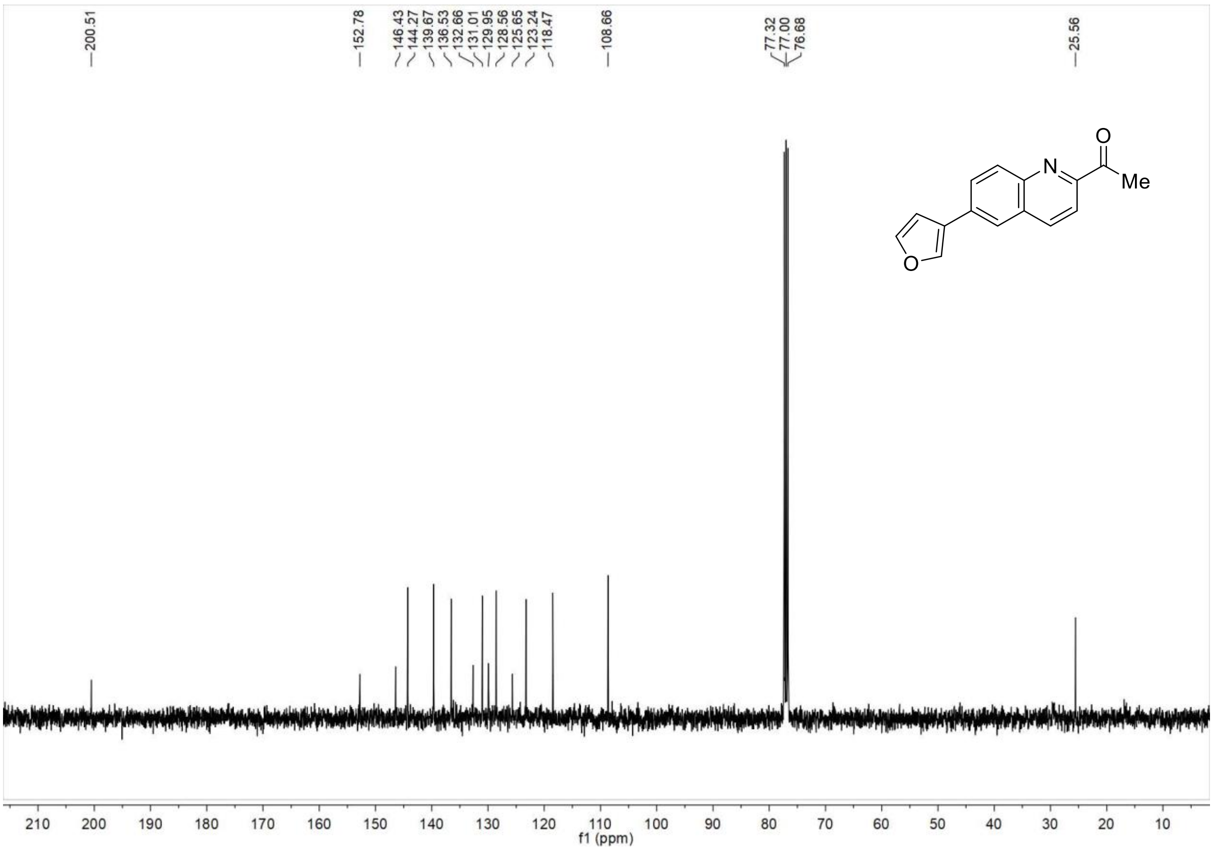

Supplementary Figure 126. ^{13}C-NMR of compound **27**, recorded at 400 MHz and 25 °C in $CDCl_3$

1-(3-(dibenzo[*b*,*d*]furan-3-yl)Quinolin-2-yl)ethan-1-one (28)

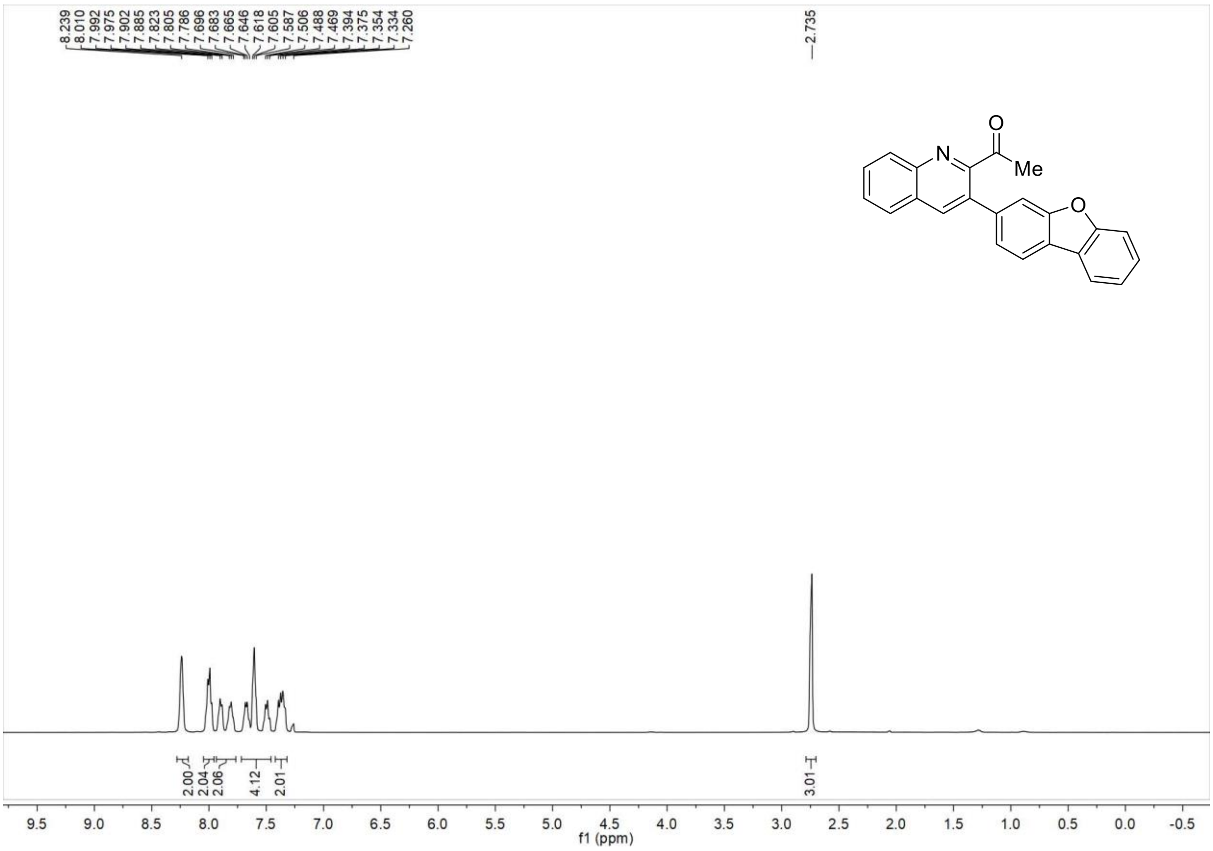

Supplementary Figure 127. ^{1}H-NMR of compound **28** recorded at 400 MHz and 25 °C in $CDCl_3$

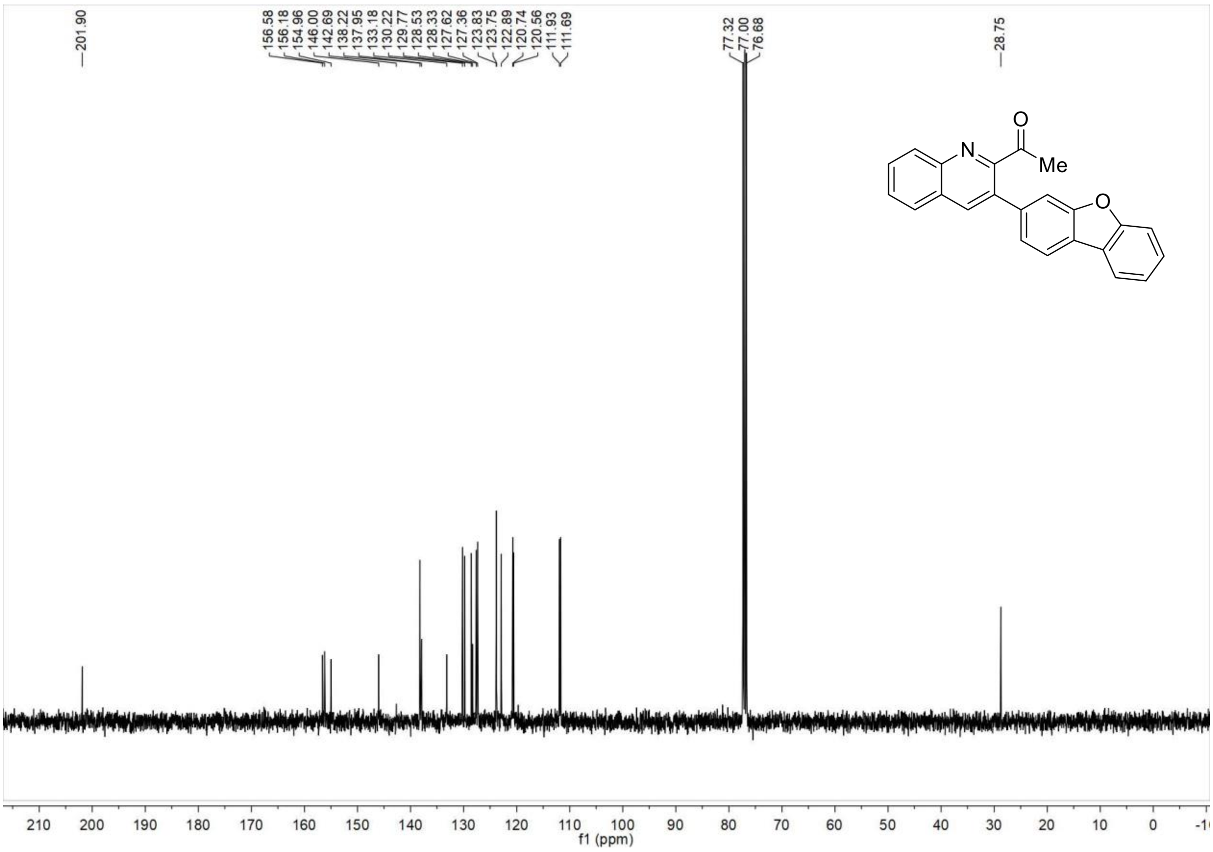

Supplementary Figure 128. ^{13}C-NMR of compound **28**, recorded at 400 MHz and 25 °C in $CDCl_3$

1-([3,6'-biquinolin]-2'-yl)Ethan-1-one (29)

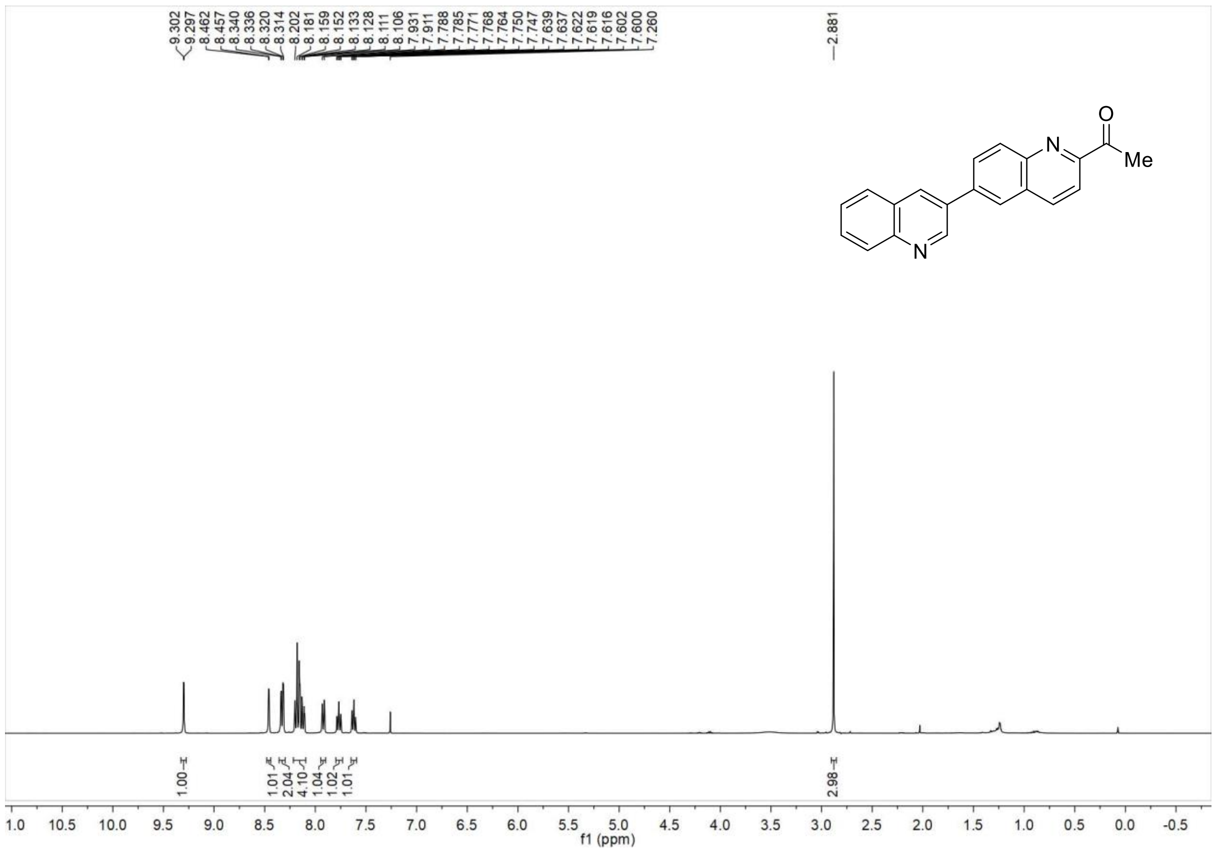

Supplementary Figure 129. ^{1}H-NMR of compound **29** recorded at 400 MHz and 25 °C in $CDCl_3$

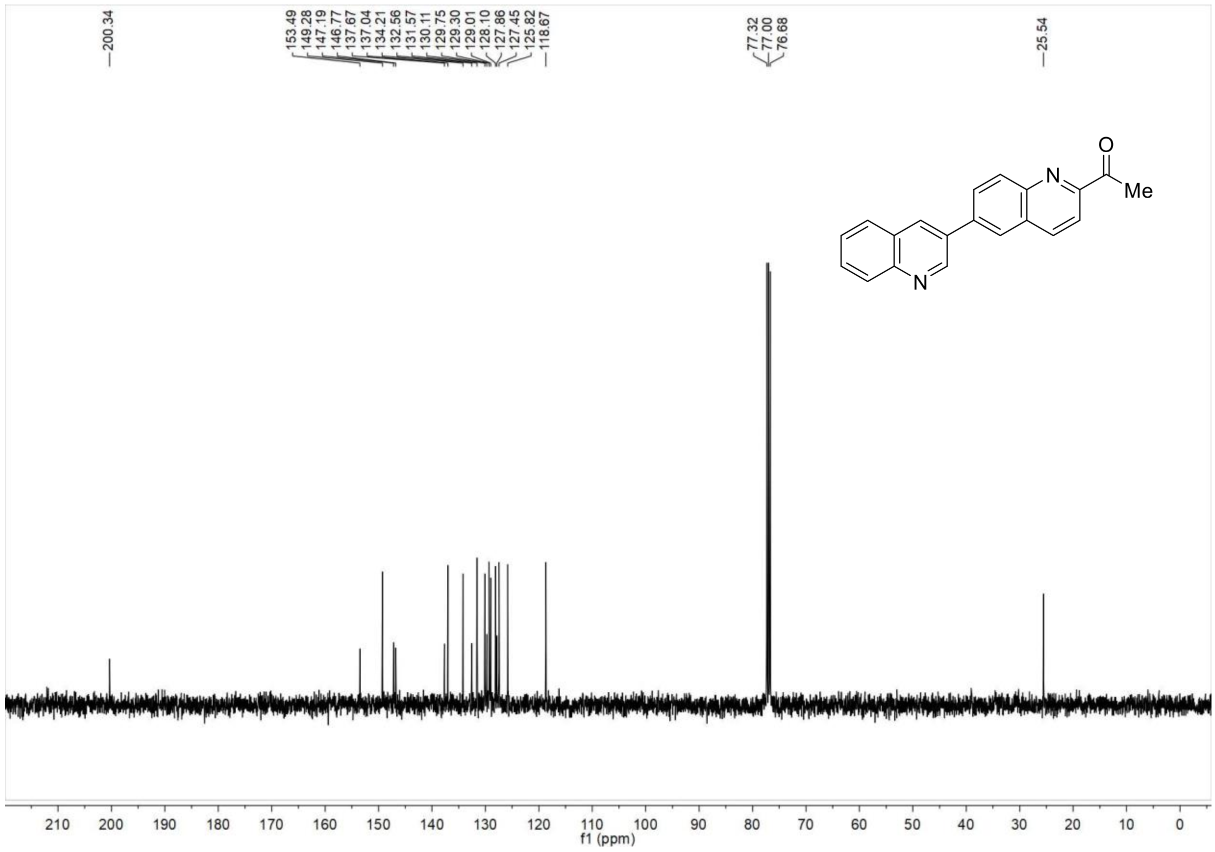

Supplementary Figure 130. ^{13}C-NMR of compound **29**, recorded at 400 MHz and 25 °C in $CDCl_3$

1-(3-(phenoxathiin-4-yl)quinolin-2-yl)Ethan-1-one (30)

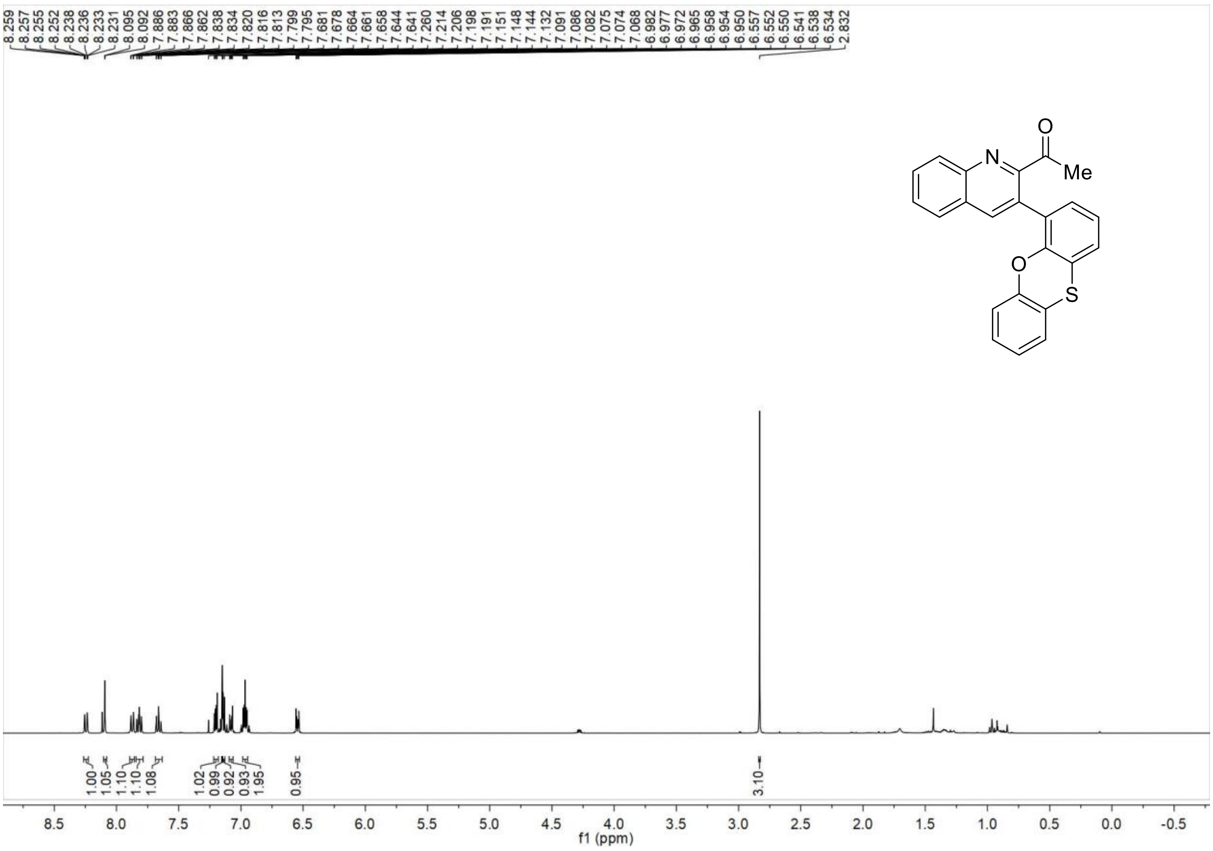

Supplementary Figure 131. ^{1}H-NMR of compound **30** recorded at 400 MHz and 25 °C in CDCl3

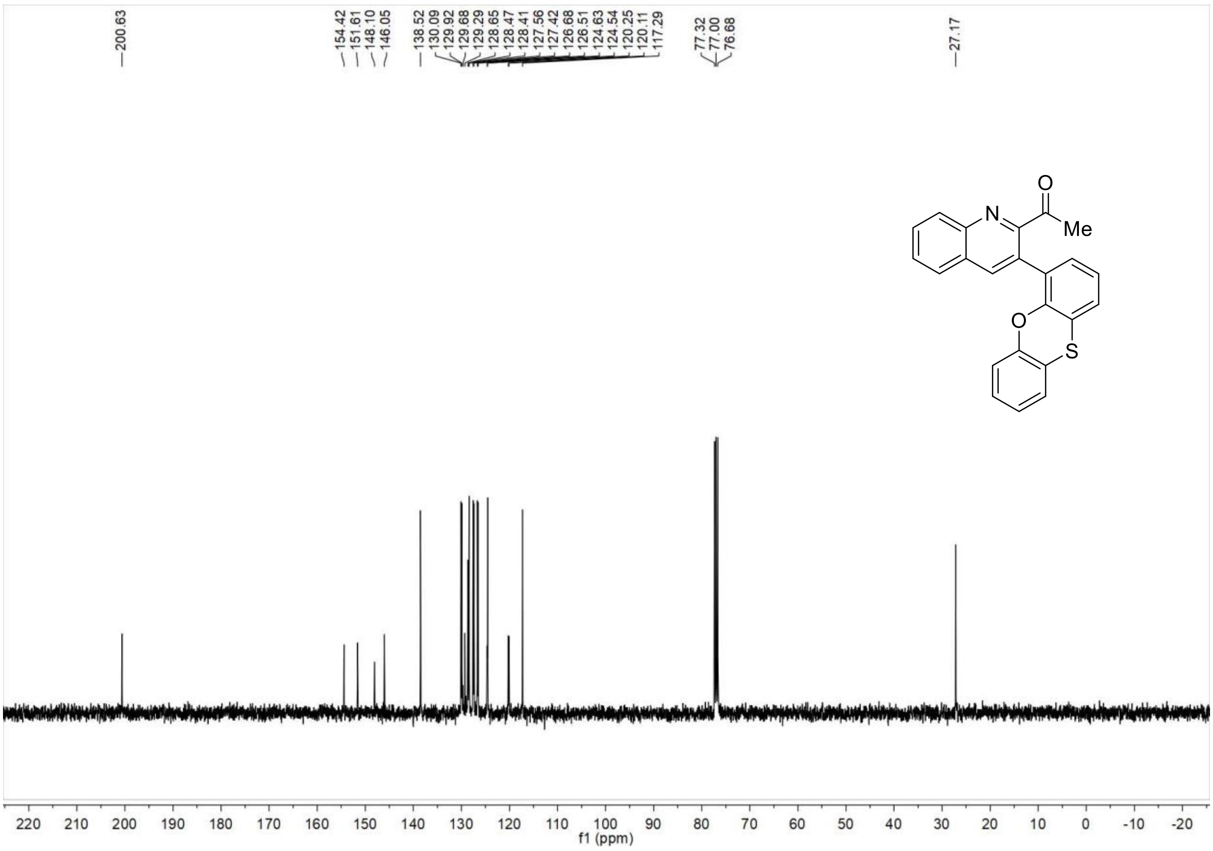

Supplementary Figure 132. ^{13}C-NMR of compound **30**, recorded at 400 MHz and 25 °C in CDCl3

1-(thieno[3,2-*c*]quinolin-4-yl)Ethan-1-one (31)

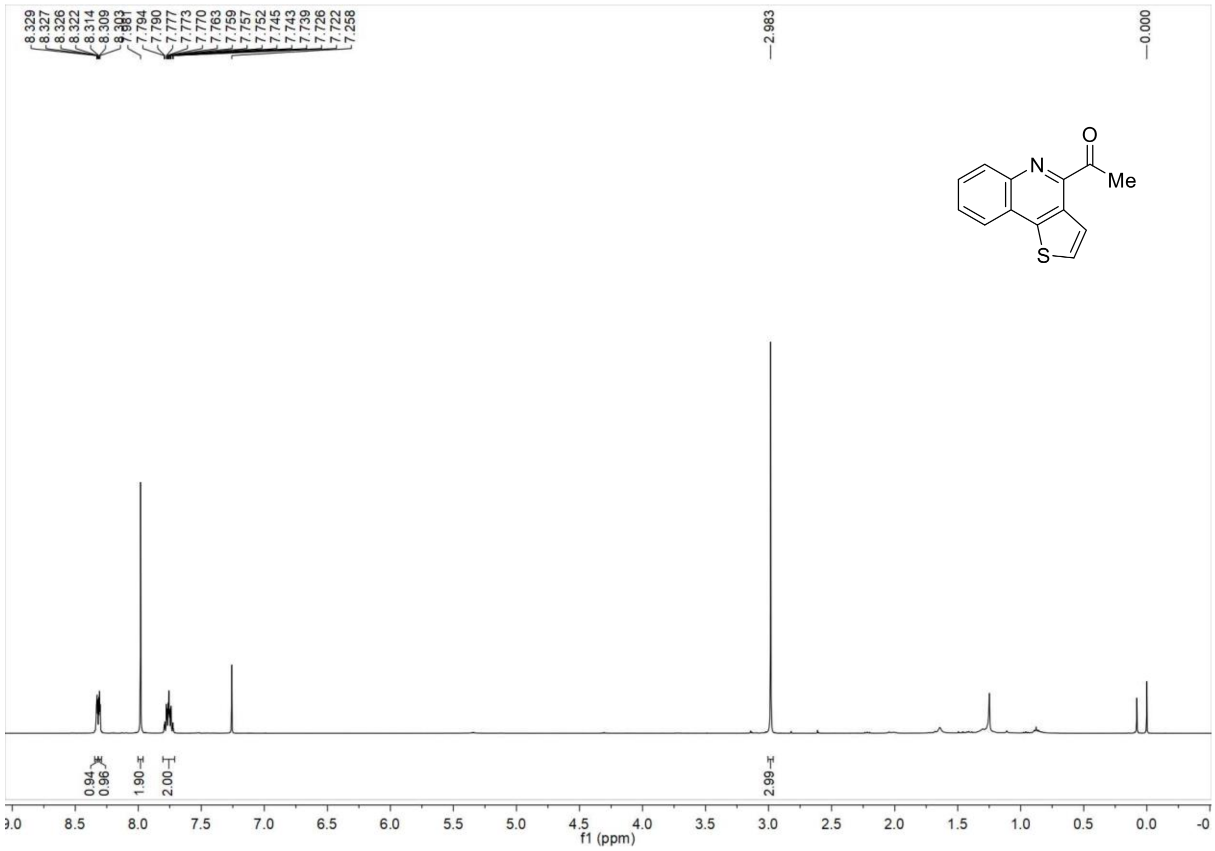

Supplementary Figure 133. ^{1}H-NMR of compound **31** recorded at 400 MHz and 25 °C in $CDCl_3$

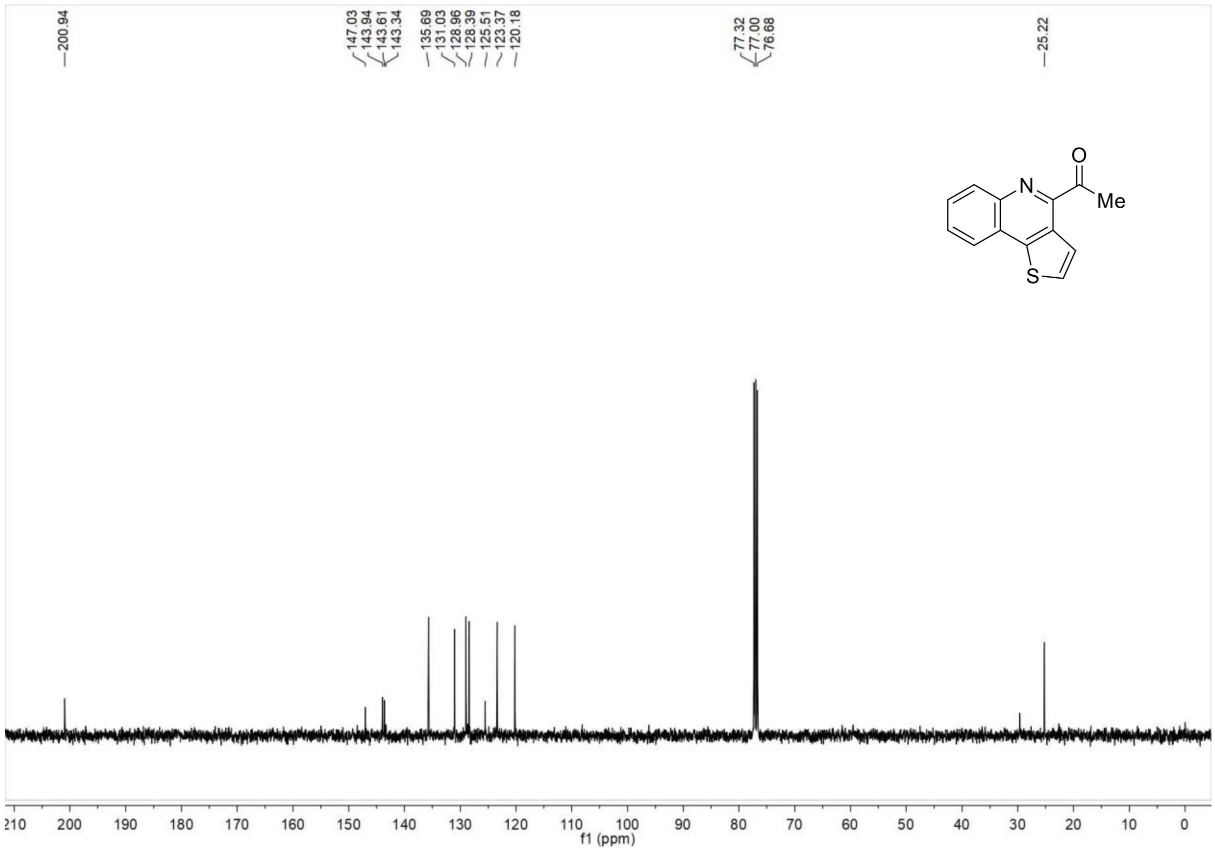

Supplementary Figure 134. ^{13}C-NMR of compound **31**, recorded at 400 MHz and 25 °C in $CDCl_3$

1-(furo[3,2-*c*]quinolin-4-yl)Ethan-1-one (32)

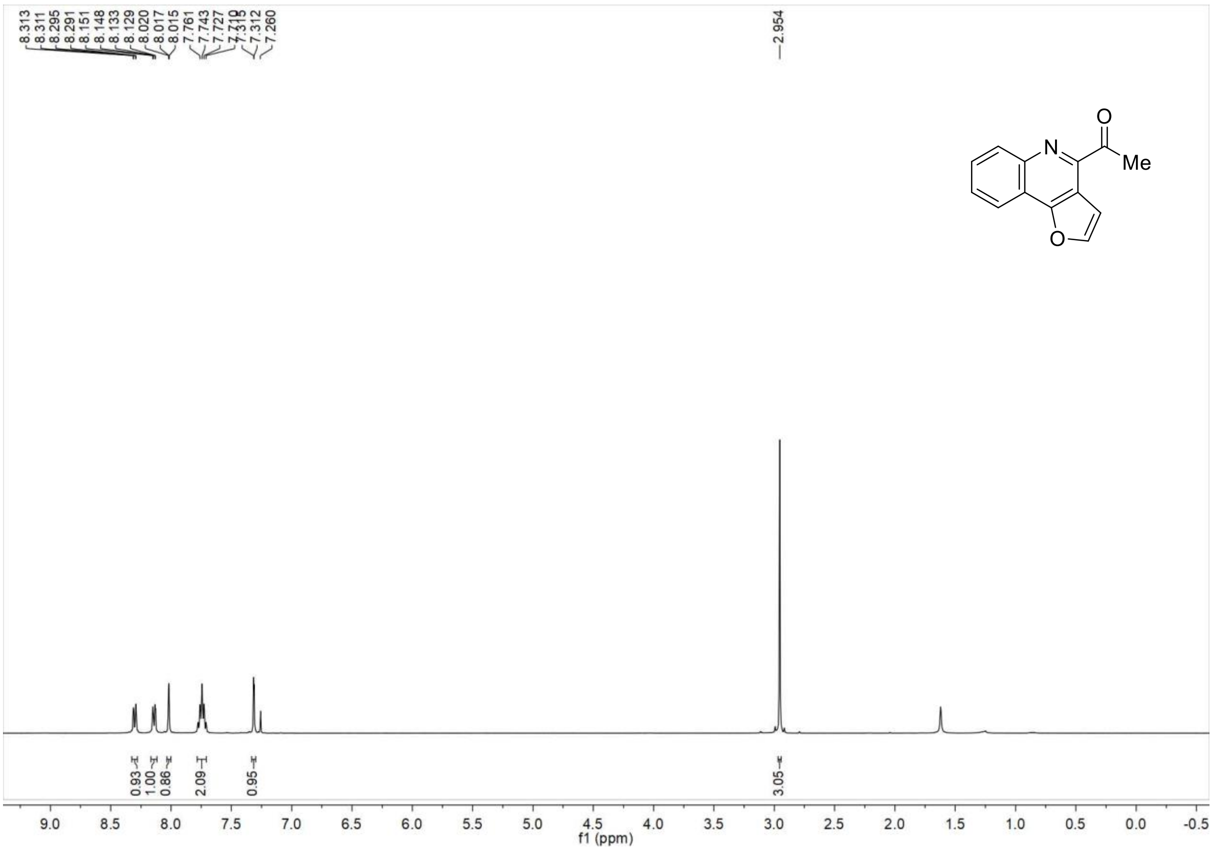

Supplementary Figure 135. ^{1}H-NMR of compound **32** recorded at 400 MHz and 25 °C in $CDCl_3$

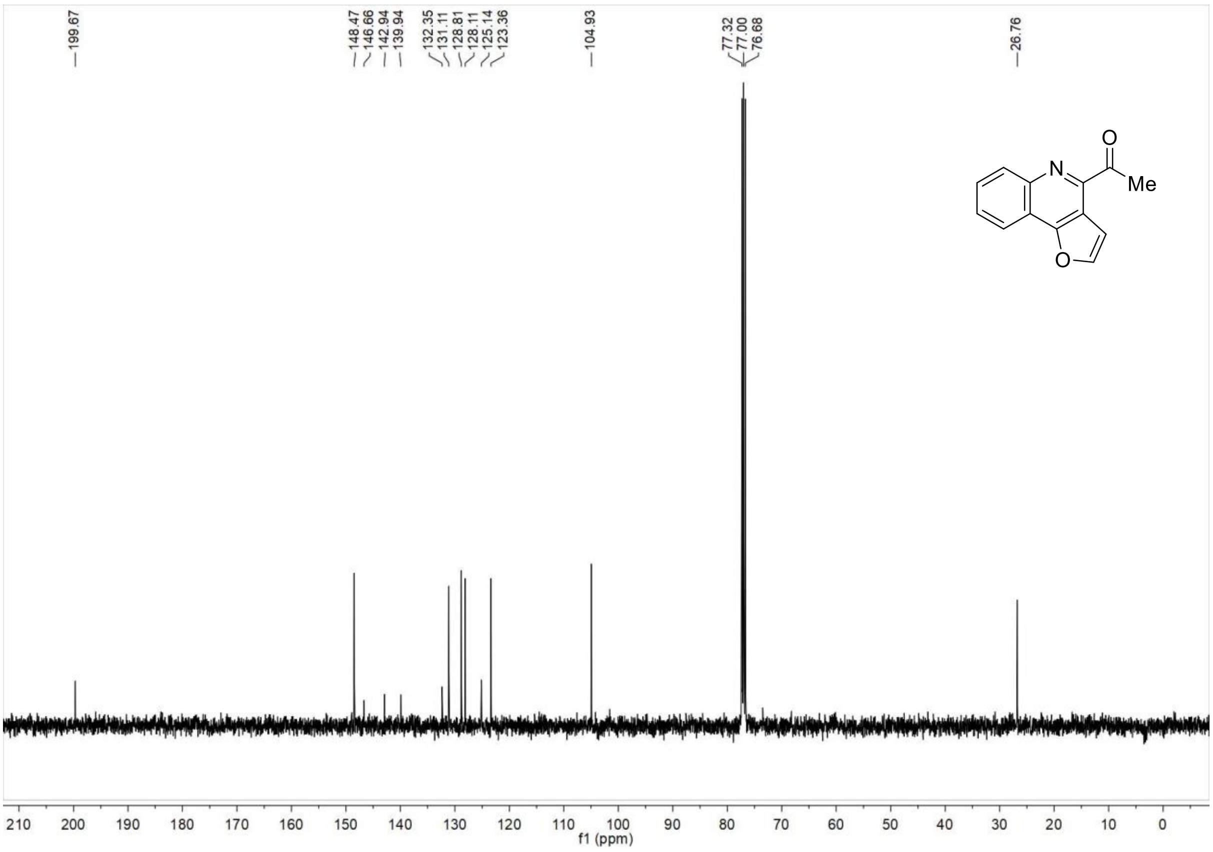

Supplementary Figure 136. ^{13}C-NMR of compound **32**, recorded at 400 MHz and 25 °C in $CDCl_3$

1-(3-methylbenzo[*g*]quinolin-2-yl)Ethan-1-one (33)

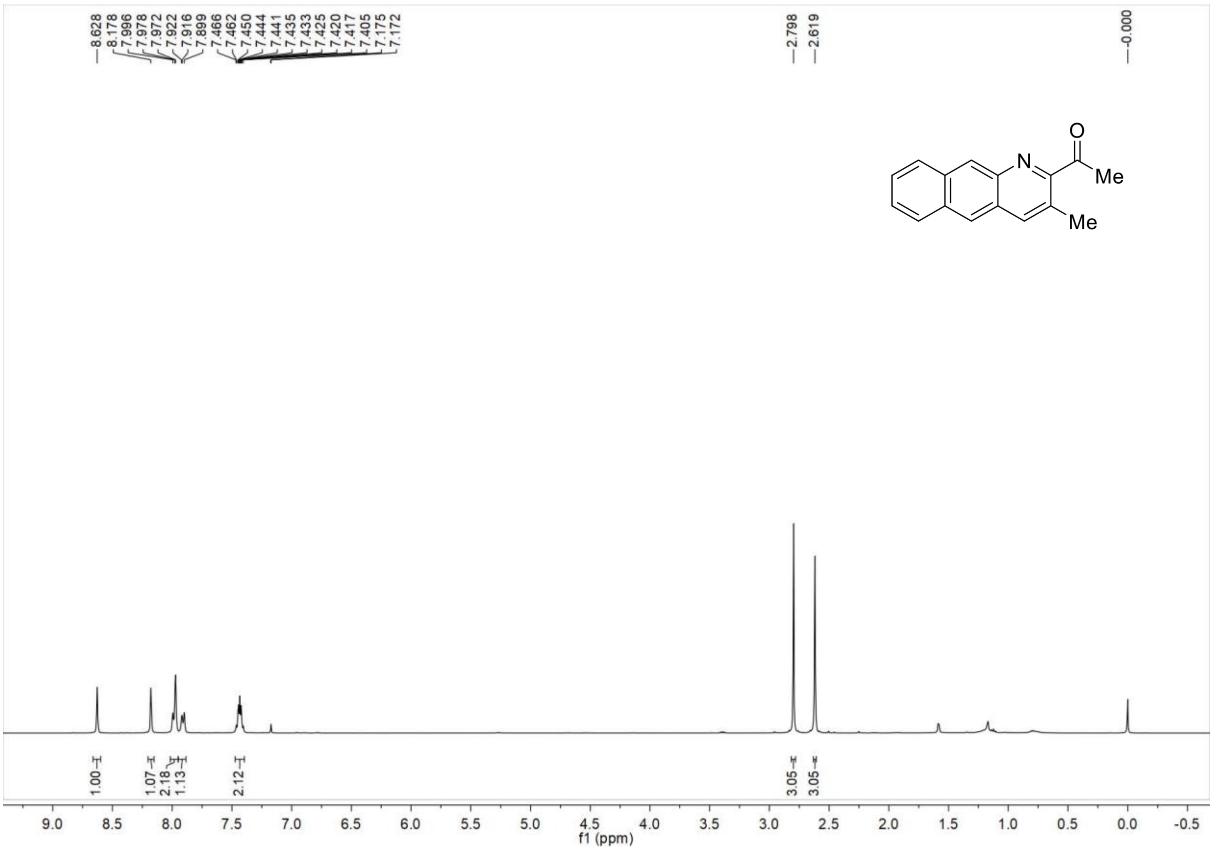

Supplementary Figure 137. ^{1}H-NMR of compound **33** recorded at 400 MHz and 25 °C in $CDCl_3$

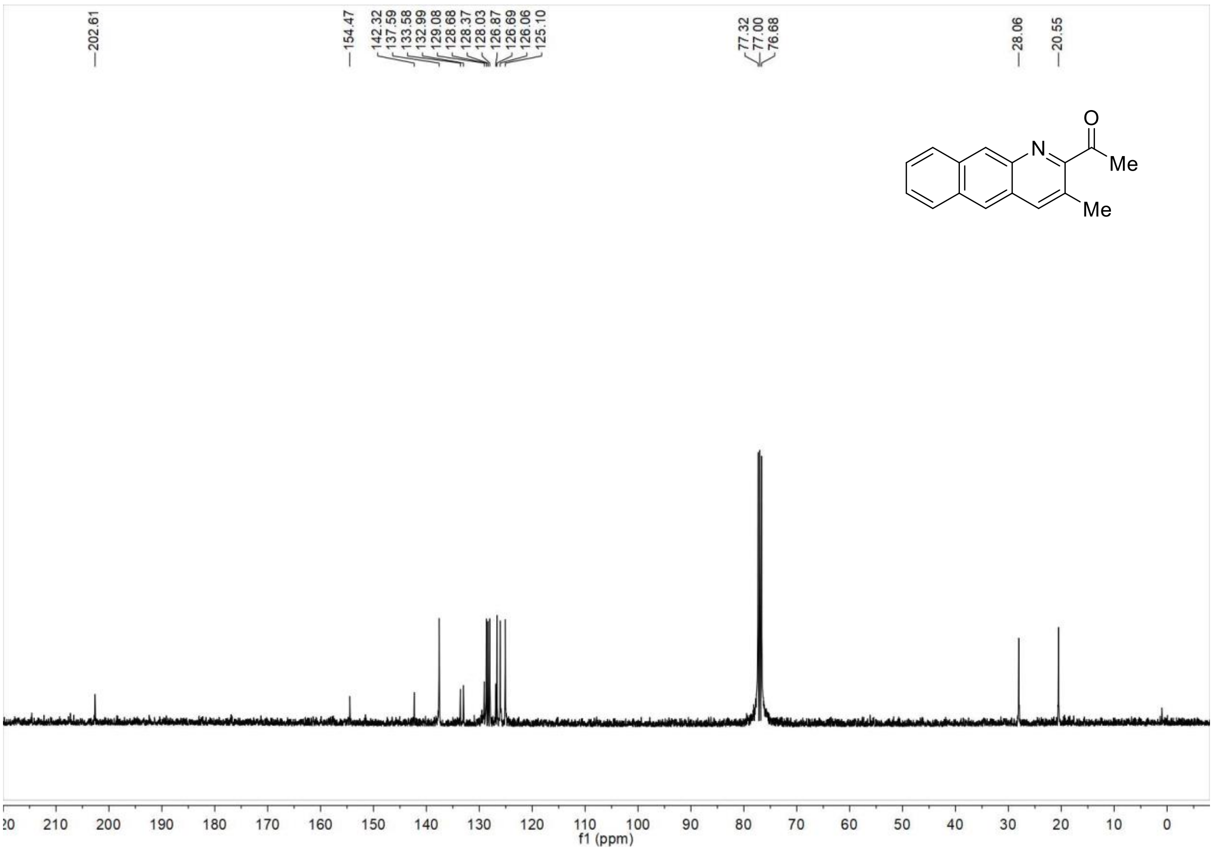

Supplementary Figure 138. ^{13}C-NMR of compound **33**, recorded at 400 MHz and 25 °C in $CDCl_3$

1-(2-methylnaphtho[2,3-*f*]quinolin-3-yl)Ethan-1-one (34)

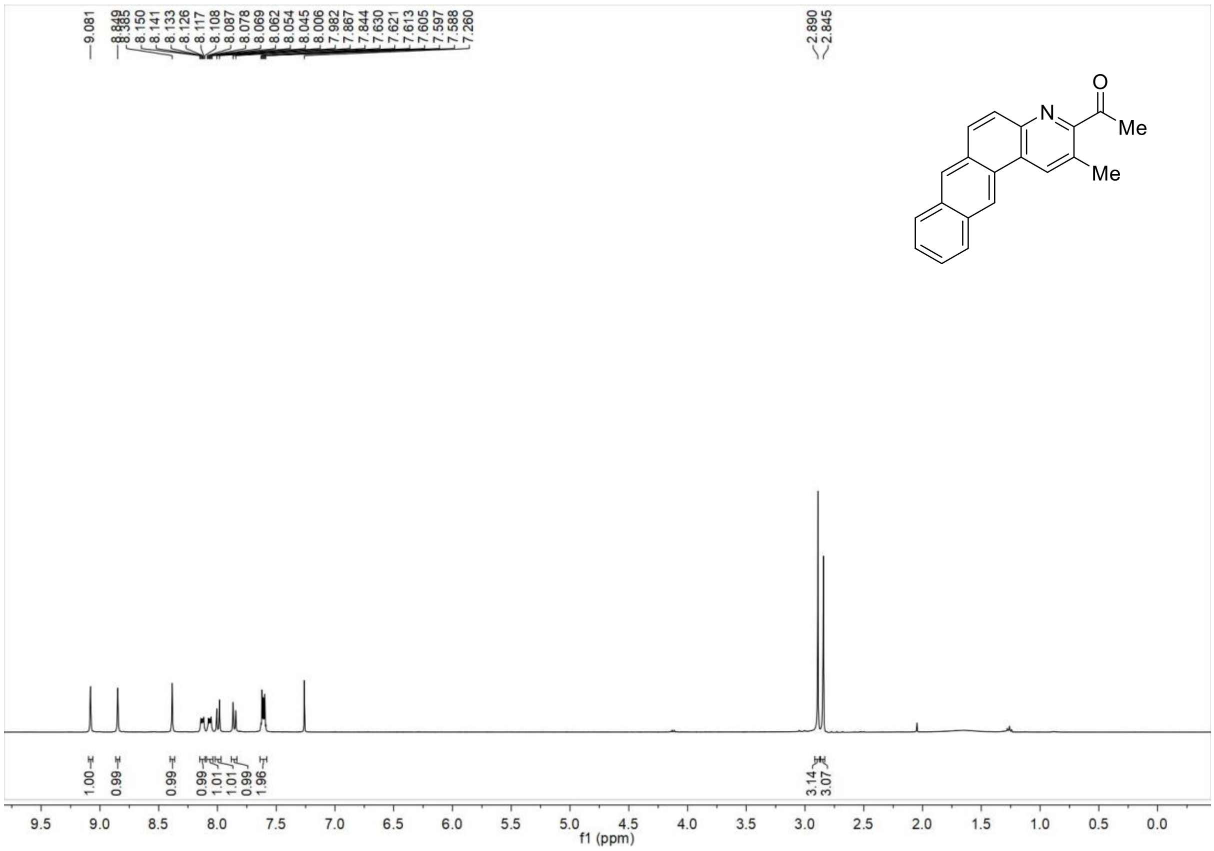

Supplementary Figure 139. ^{1}H-NMR of compound **34** recorded at 400 MHz and 25 °C in $CDCl_3$

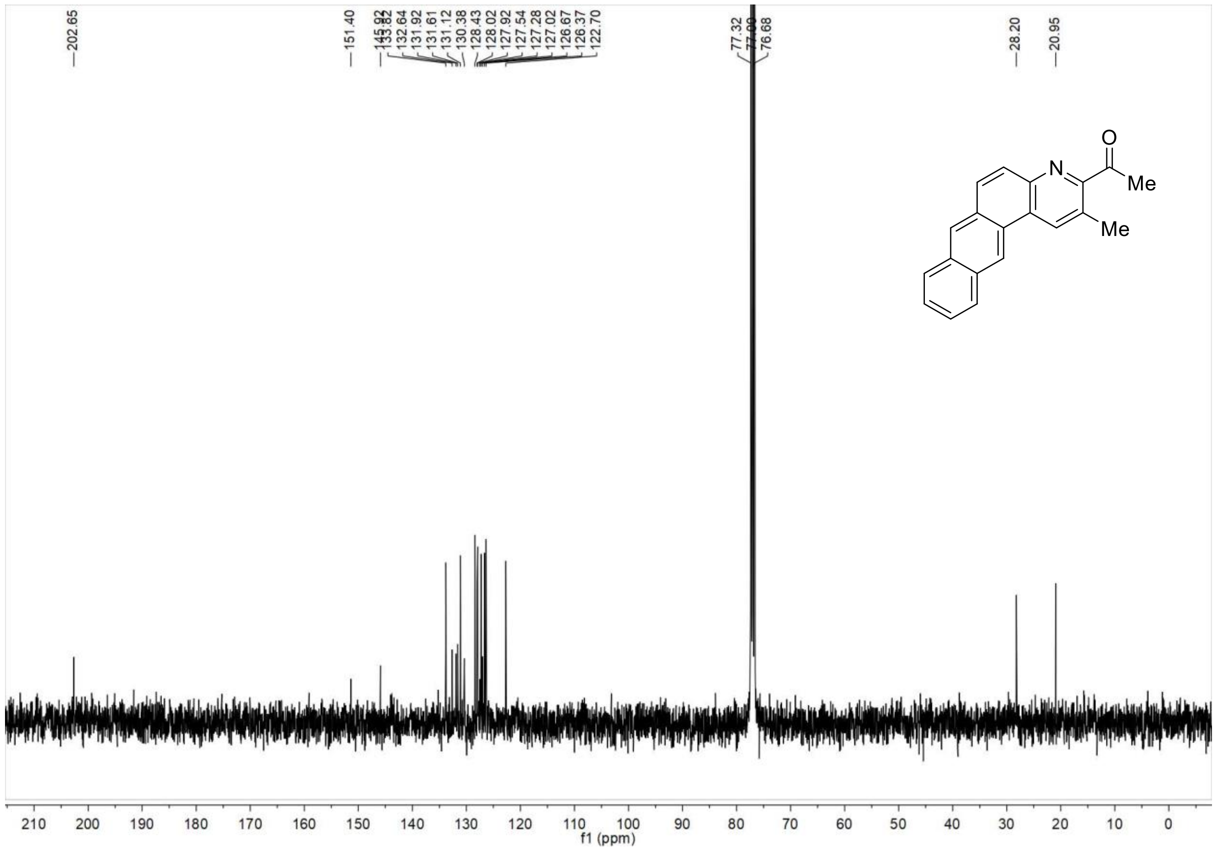

Supplementary Figure 140. ^{13}C-NMR of compound **34**, recorded at 400 MHz and 25 °C in $CDCl_3$

1-(benzo[*k*]phenanthridin-6-yl)Ethan-1-one (**35**)

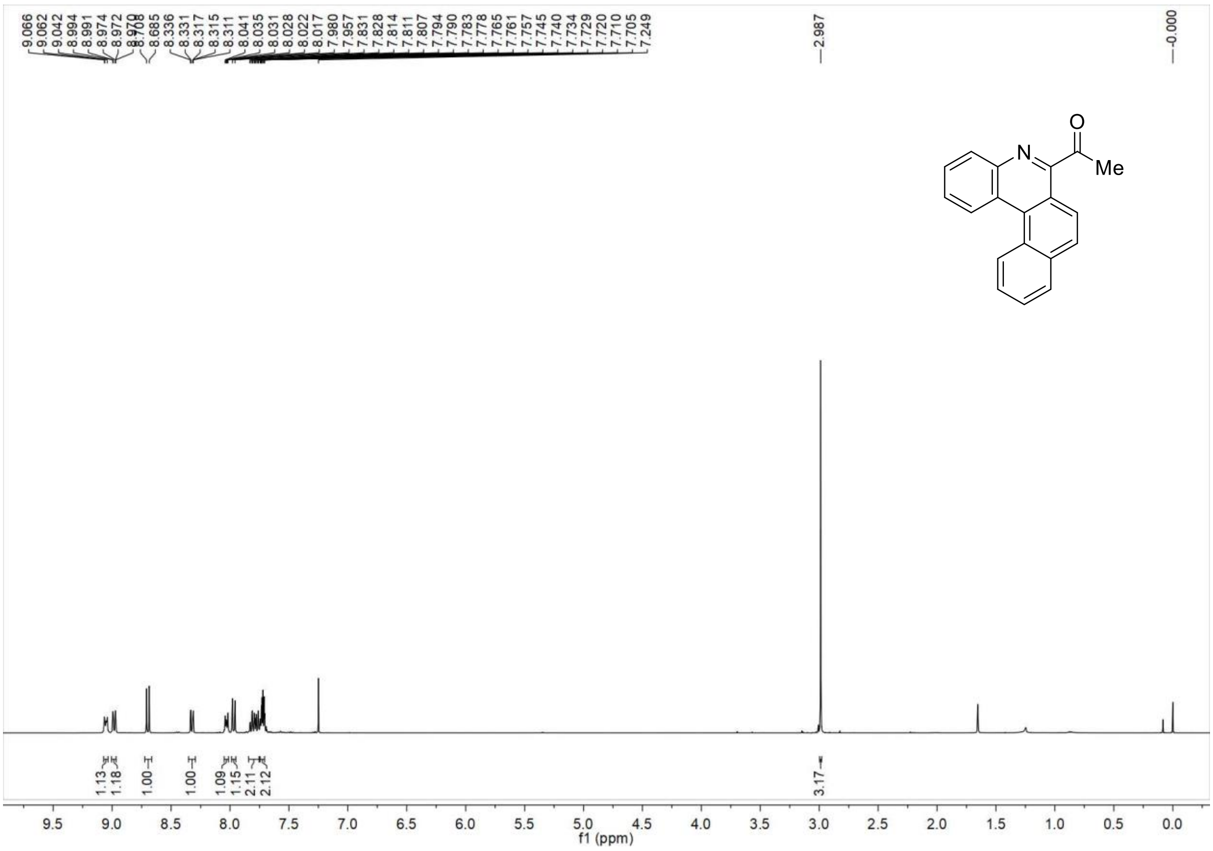

Supplementary Figure 141. ^{1}H-NMR of compound **35** recorded at 400 MHz and 25 °C in $CDCl_3$

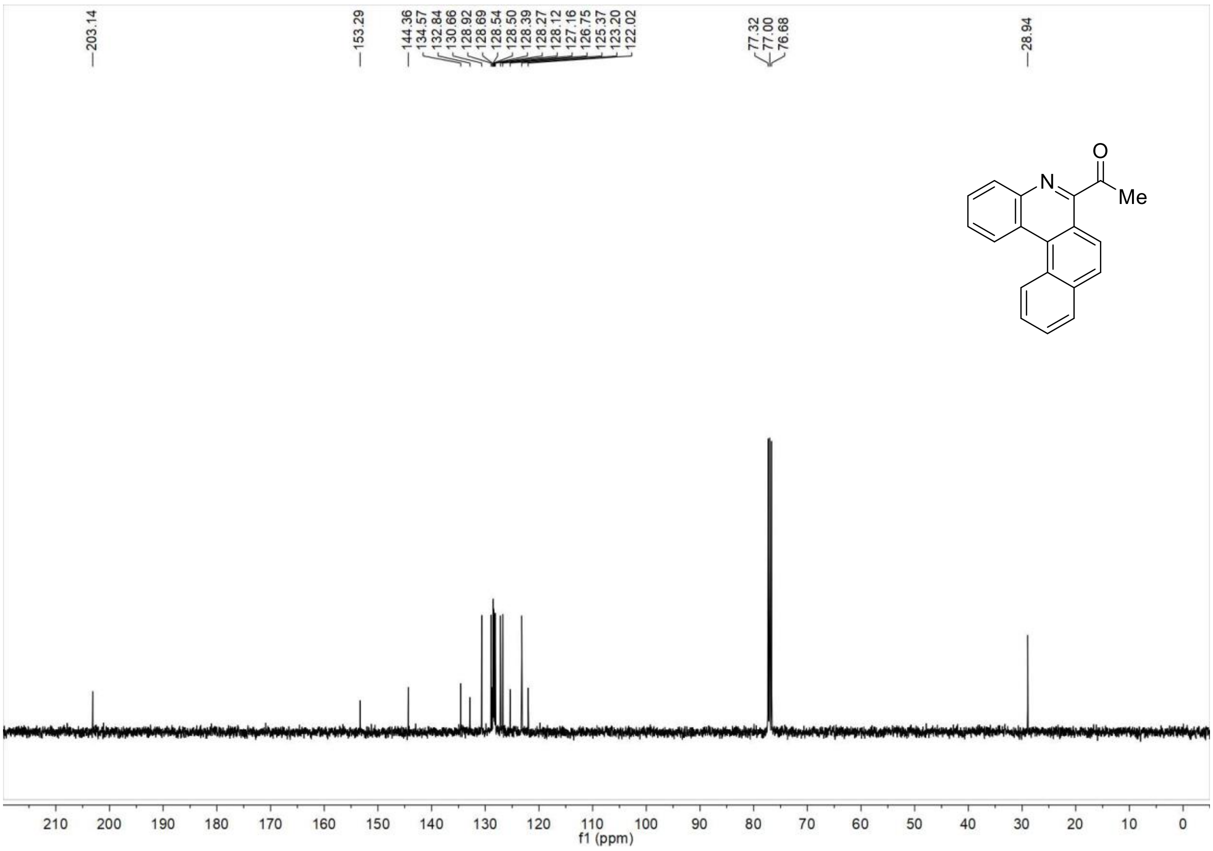

Supplementary Figure 142. ^{13}C-NMR of compound **35**, recorded at 400 MHz and 25 °C in $CDCl_3$

Methyl 6-methyl-4-(phenanthridin-6-yl)quinoline-2-carboxylate (36)

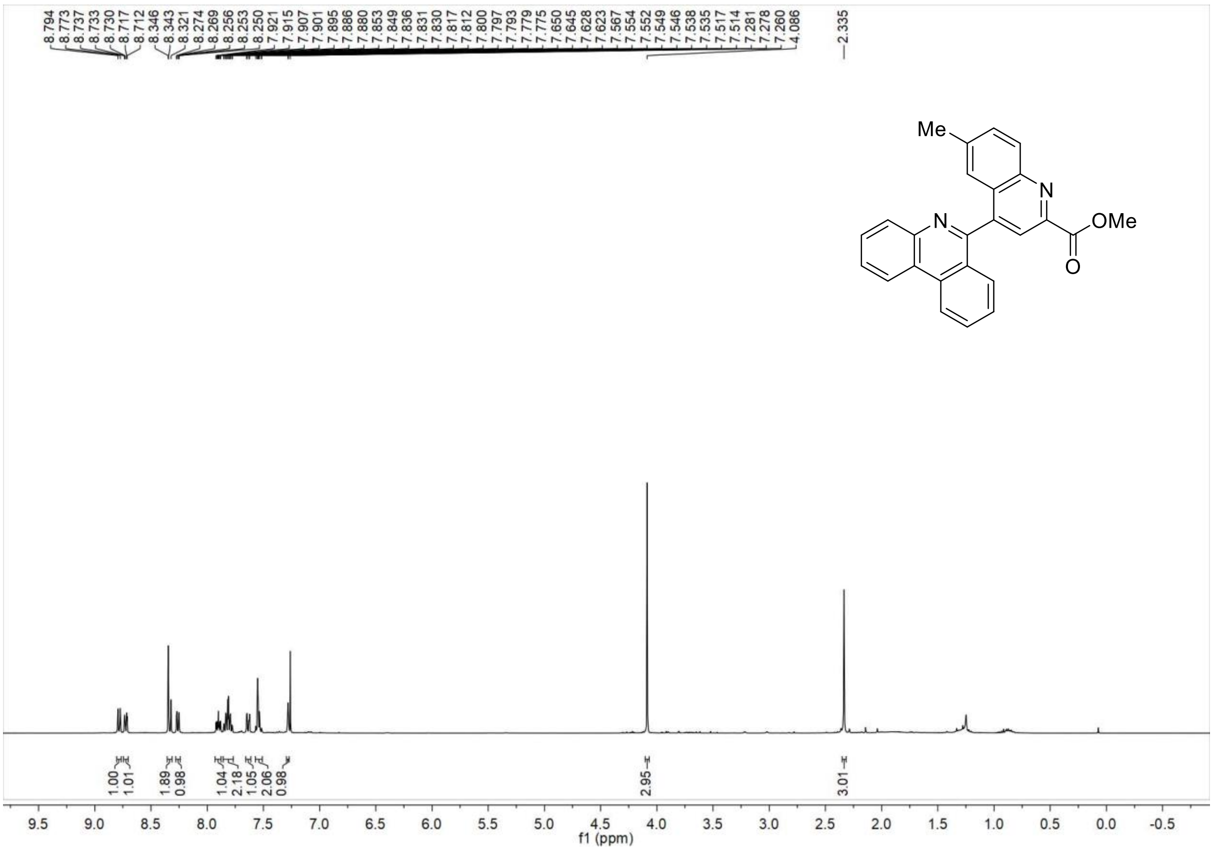

Supplementary Figure 143. ^{1}H-NMR of compound **36** recorded at 400 MHz and 25 °C in $CDCl_3$

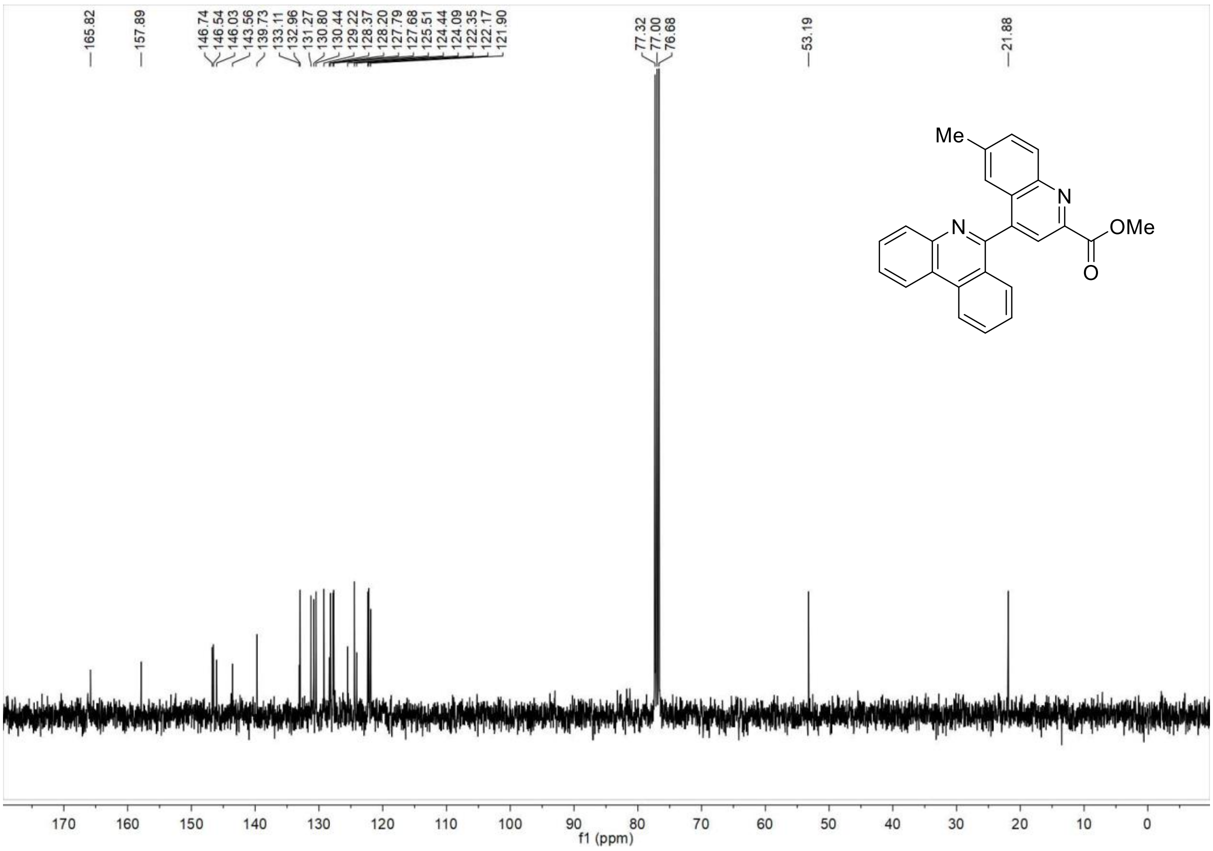

Supplementary Figure 144. ^{13}C-NMR of compound **36**, recorded at 400 MHz and 25 °C in $CDCl_3$

6-(quinolin-2-yl)Phenanthridine (37)

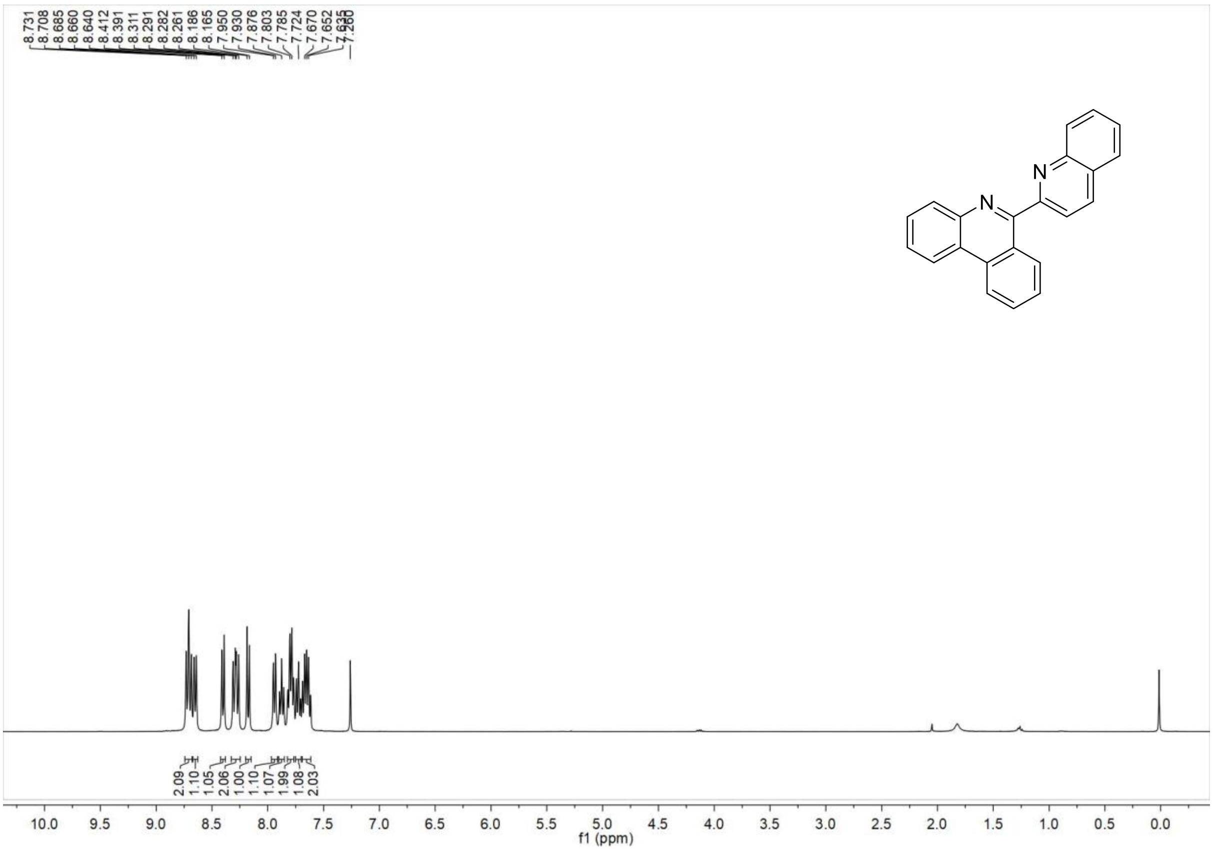

Supplementary Figure 145. ^{1}H-NMR of compound **37** recorded at 400 MHz and 25 °C in $CDCl_3$

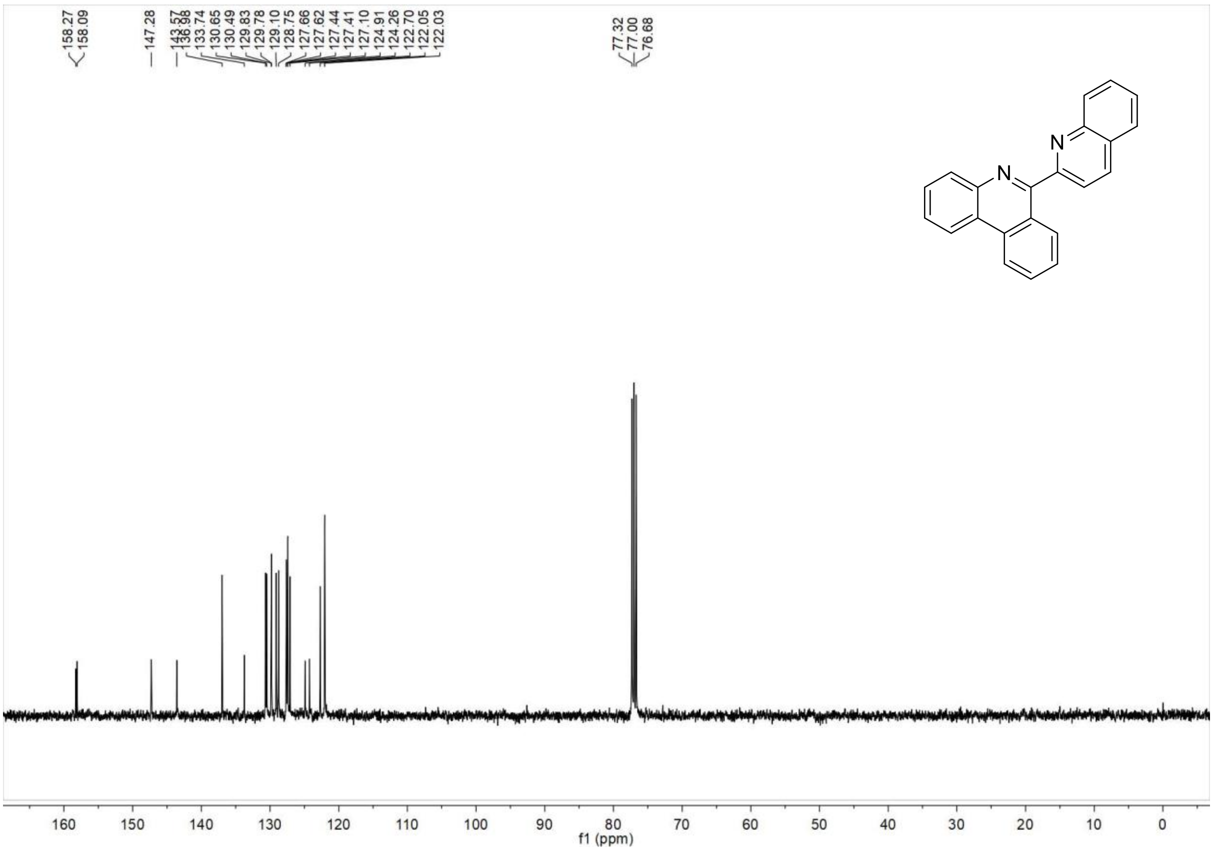

Supplementary Figure 146. ^{13}C-NMR of compound **37**, recorded at 400 MHz and 25 °C in $CDCl_3$

4-(phenanthridin-6-yl)Thiazol-2-amine (38)

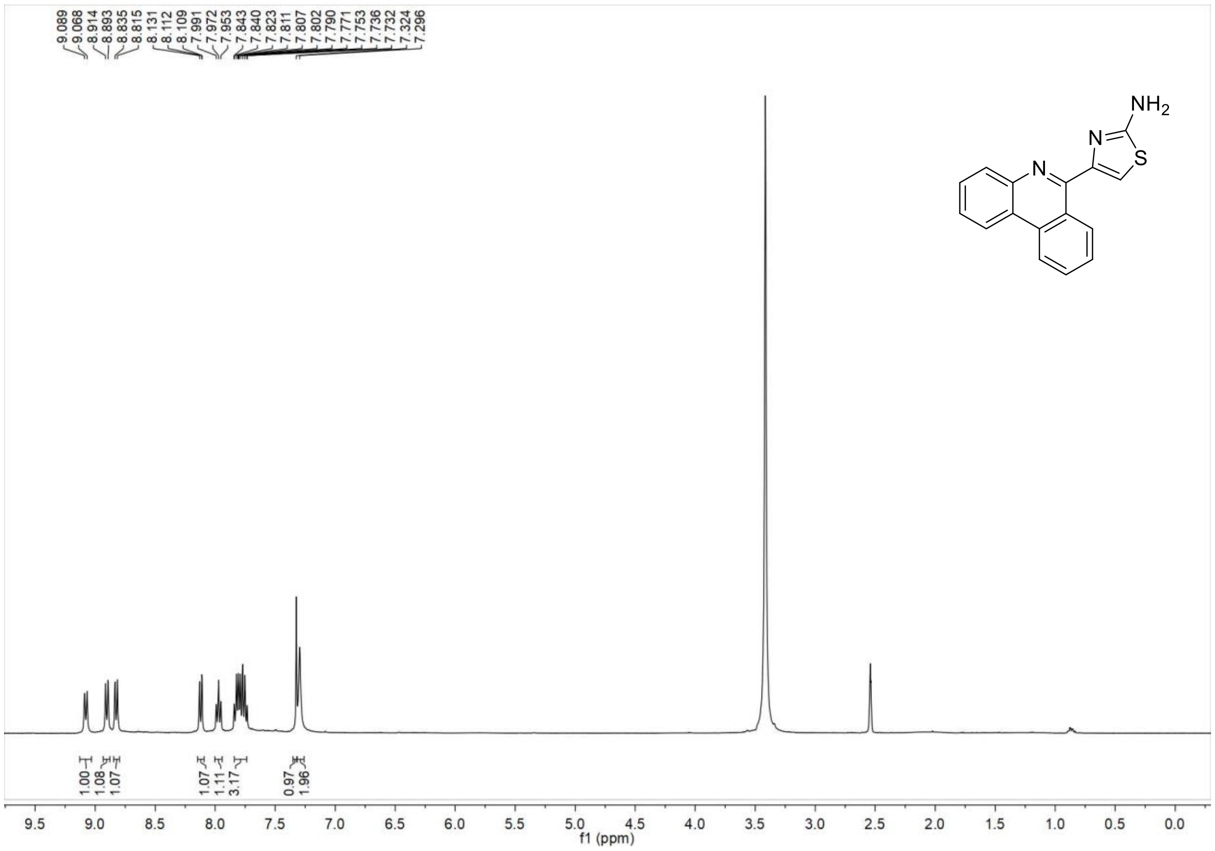

Supplementary Figure 147. ^{1}H-NMR of compound **38** recorded at 400 MHz and 25 °C in DMSO-*d*6

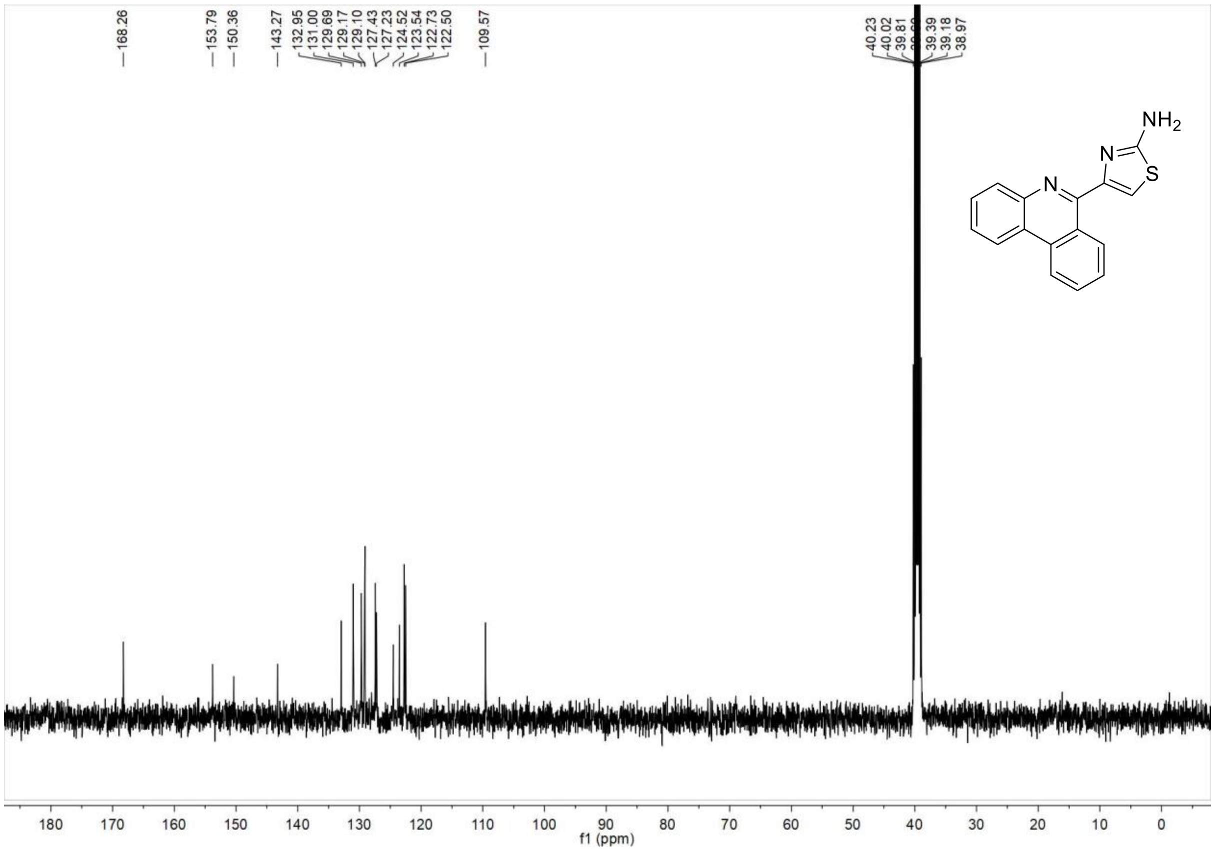

Supplementary Figure 148. ^{13}C-NMR of compound **38**, recorded at 400 MHz and 25 °C in DMSO-*d*6

6-((3*r*,5*r*,7*r*)-adamantan-1-yl)-1-methylnaphthalen-2-ol (40)

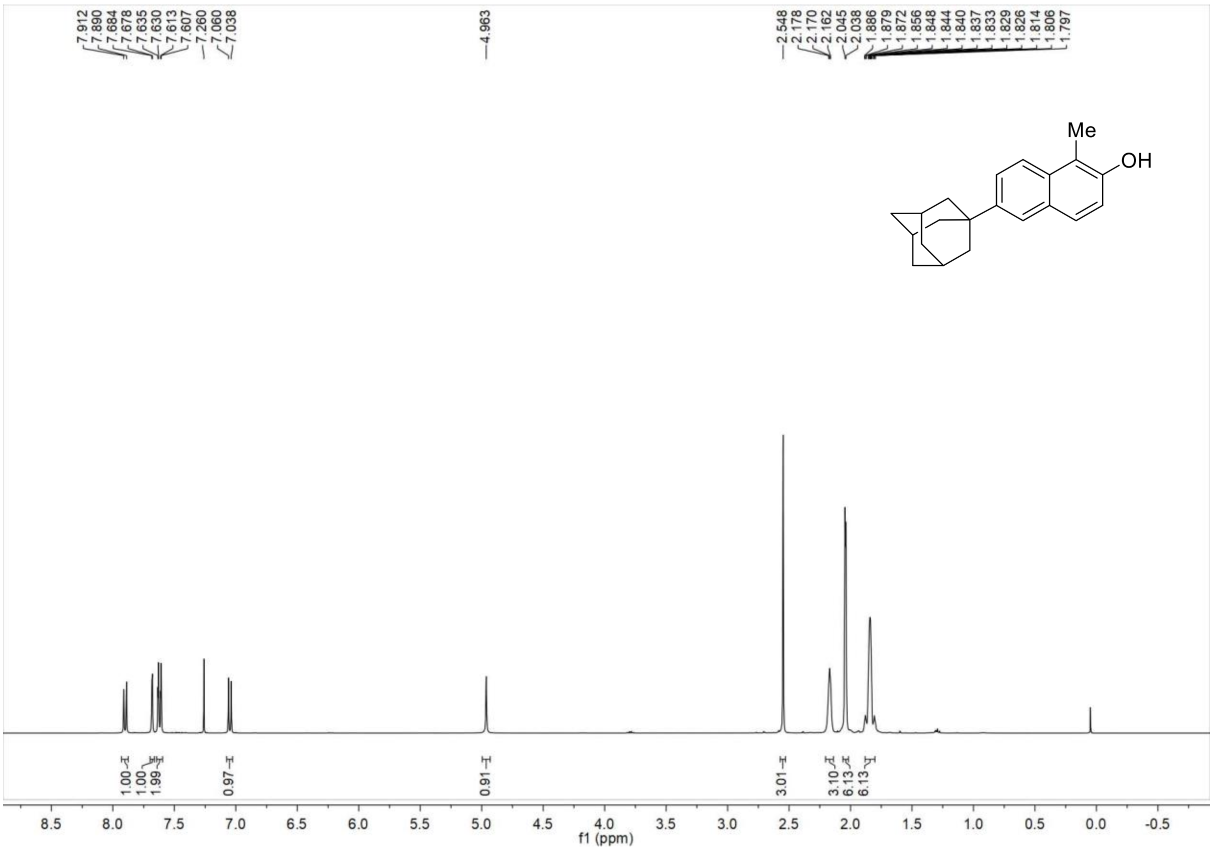

Supplementary Figure 149. ^{1}H-NMR of compound **40** recorded at 400 MHz and 25 °C in $CDCl_3$

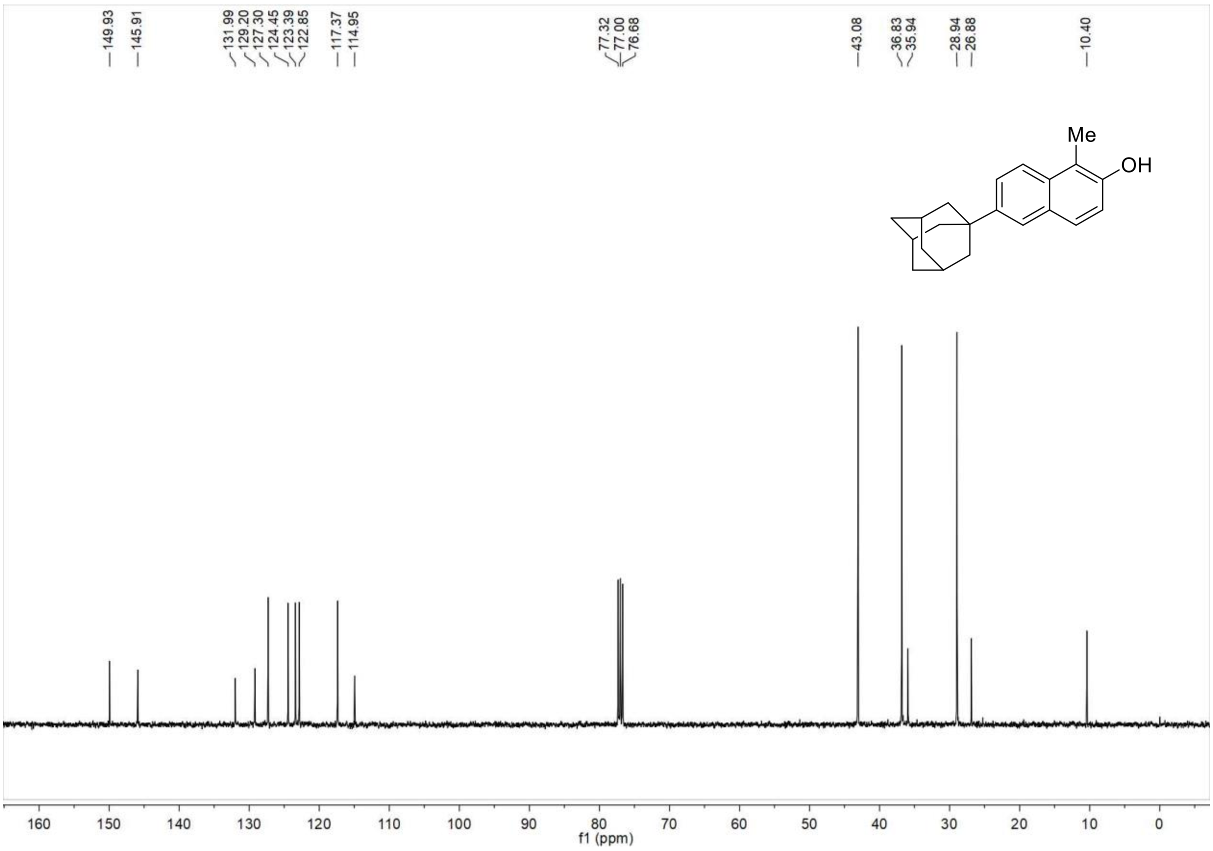

Supplementary Figure 150. ^{13}C-NMR of compound **40**, recorded at 400 MHz and 25 °C in $CDCl_3$

6-((3*r*,5*r*,7*r*)-adamantan-1-yl)-3-(*tert*-butyl)-1-methylnaphthalen-2-ol (41)

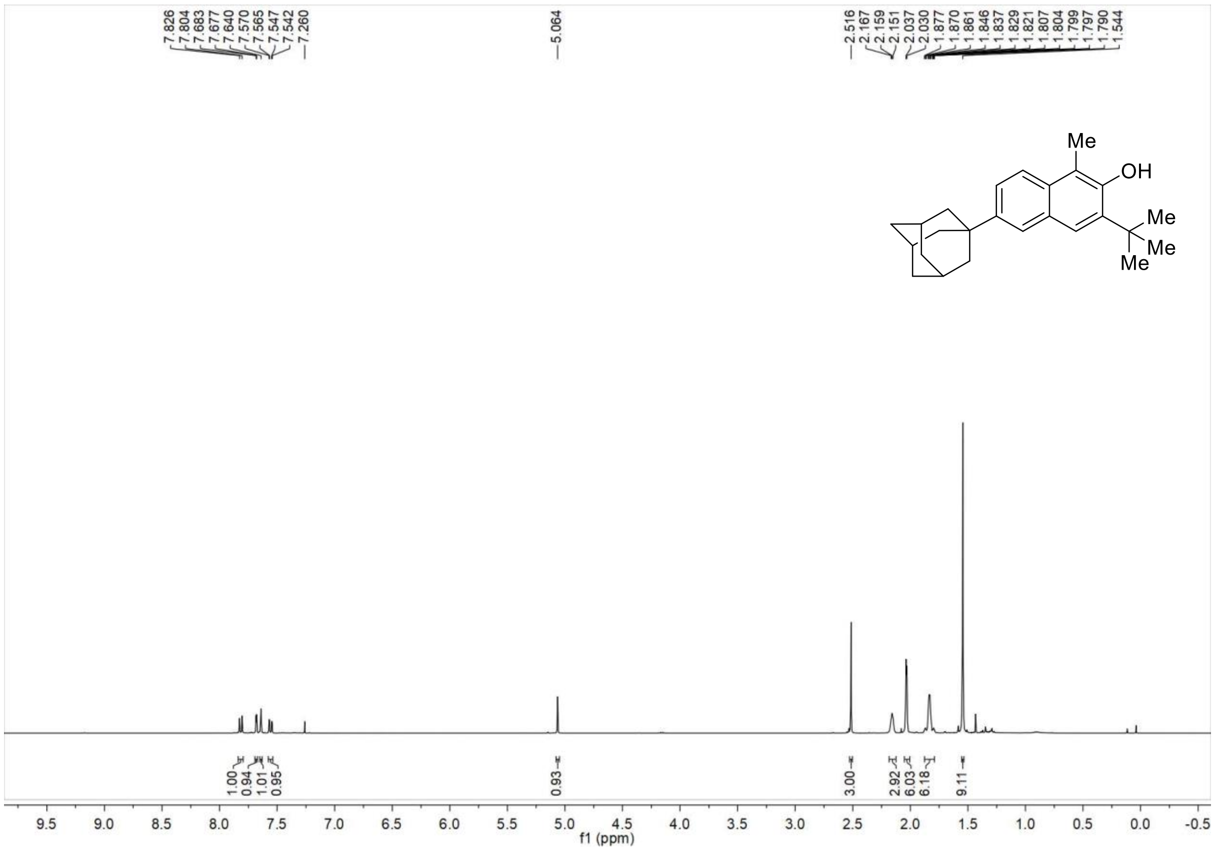

Supplementary Figure 151. ^{1}H-NMR of compound **41** recorded at 400 MHz and 25 °C in $CDCl_3$

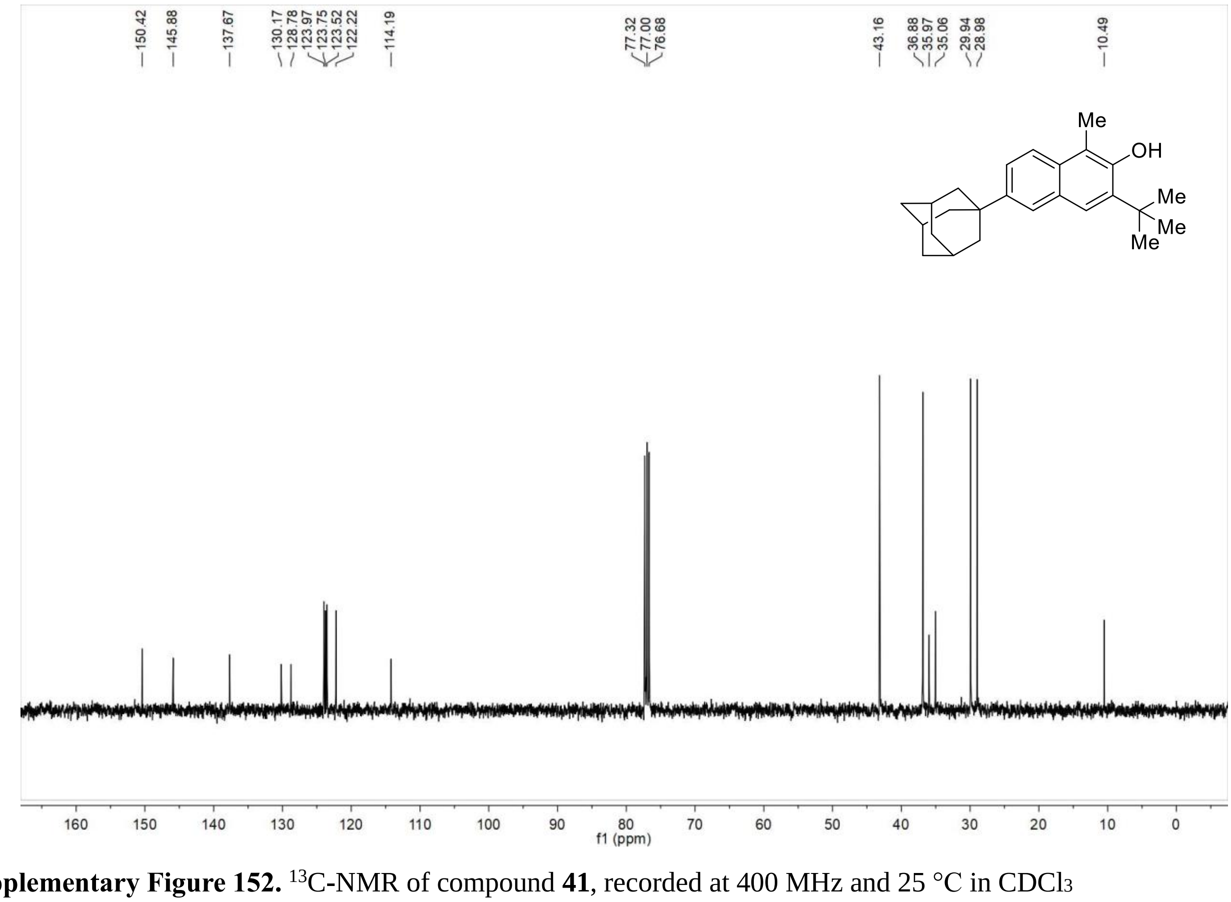

Supplementary Figure 152. ^{13}C-NMR of compound **41**, recorded at 400 MHz and 25 °C in $CDCl_3$

1-(6-((3*r*,5*r*,7*r*)-adamantan-1-yl)-3-(tert-butyl)quinolin-2-yl)ethan-1-one (42)

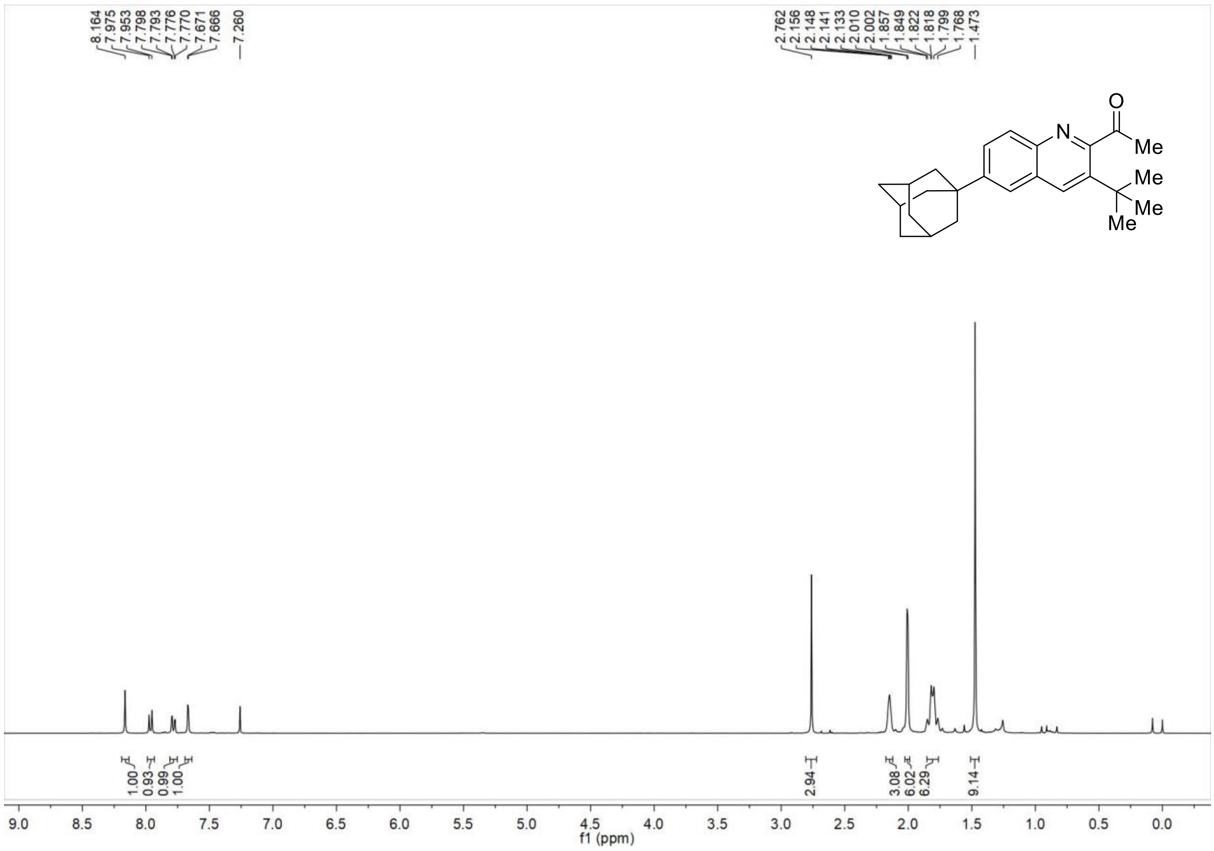

Supplementary Figure 153. ^{1}H-NMR of compound **42** recorded at 400 MHz and 25 °C in $CDCl_3$

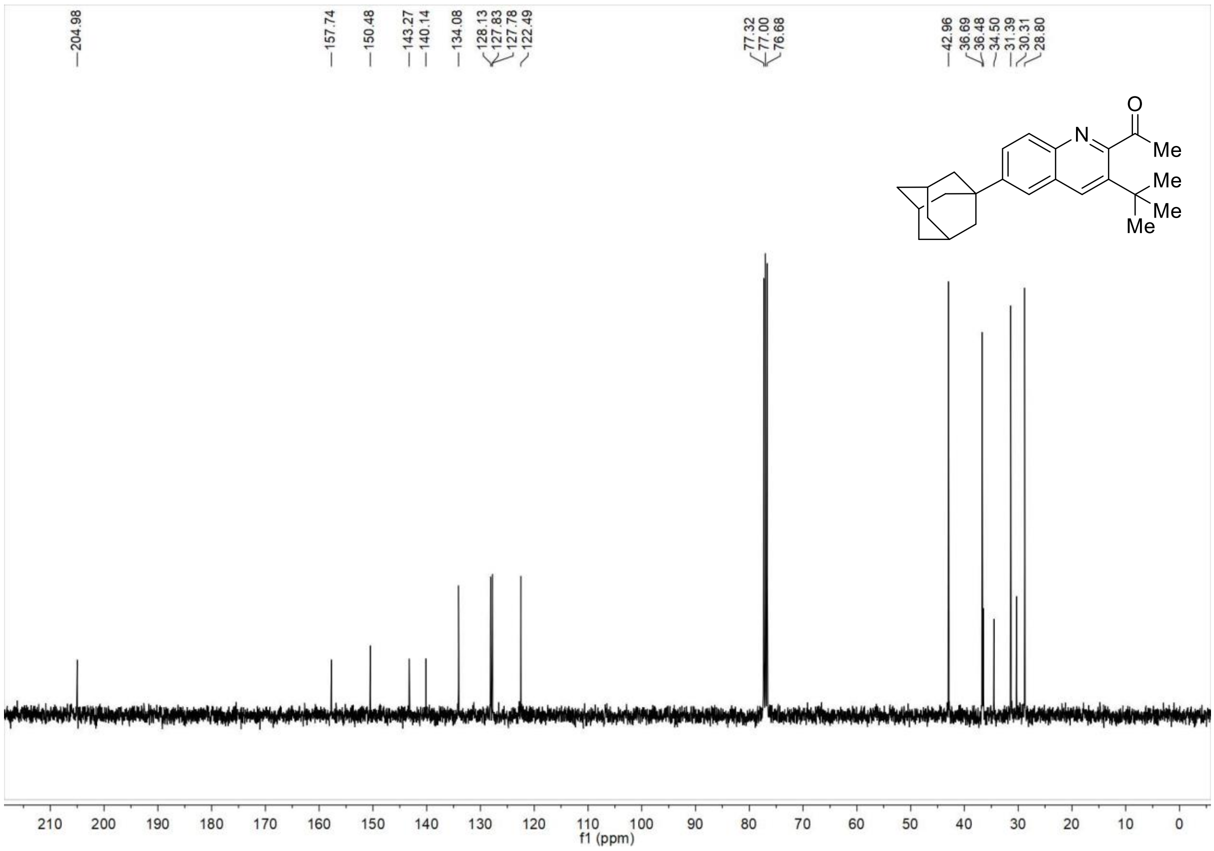

Supplementary Figure 154. ^{13}C-NMR of compound **42**, recorded at 400 MHz and 25 °C in $CDCl_3$

1-(4-chloroquinolin-2-yl)Ethan-1-one (43a)

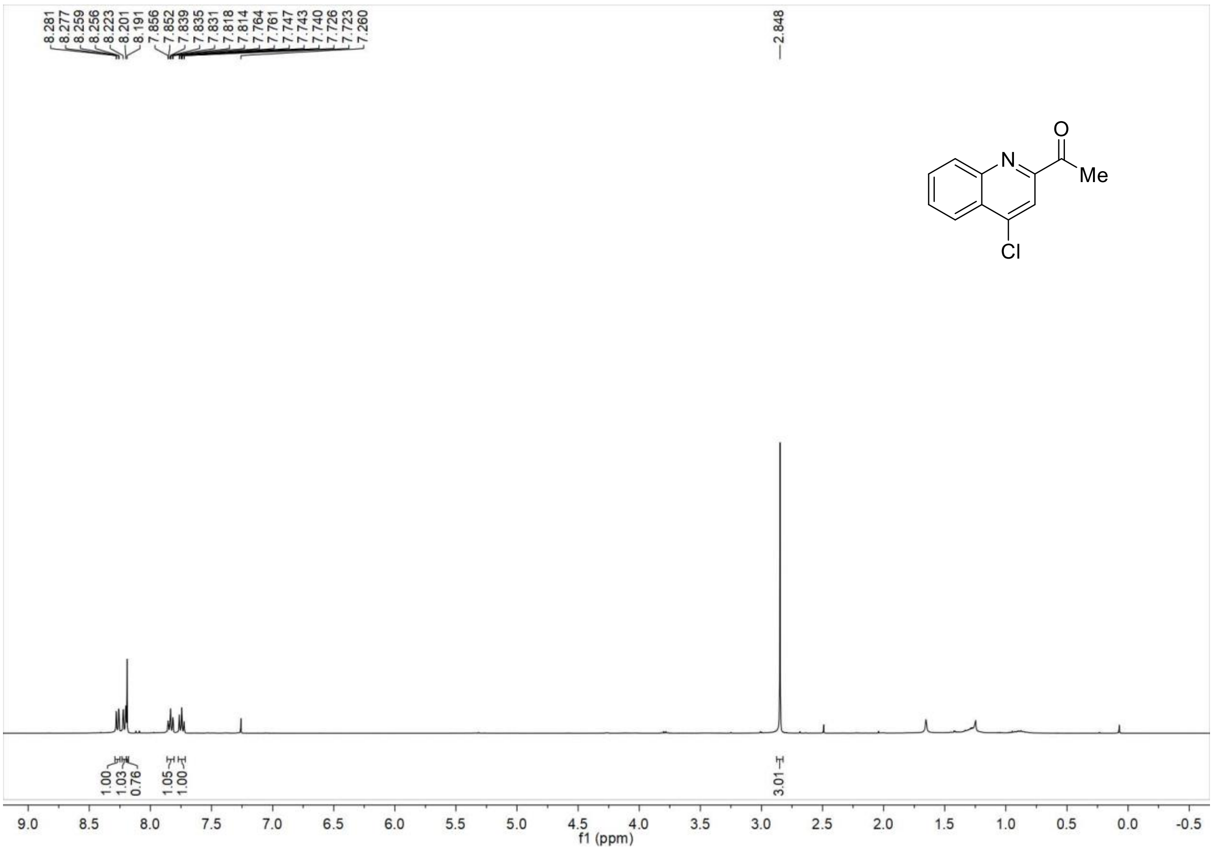

Supplementary Figure 155. ^{1}H-NMR of compound **43a** recorded at 400 MHz and 25 °C in $CDCl_3$

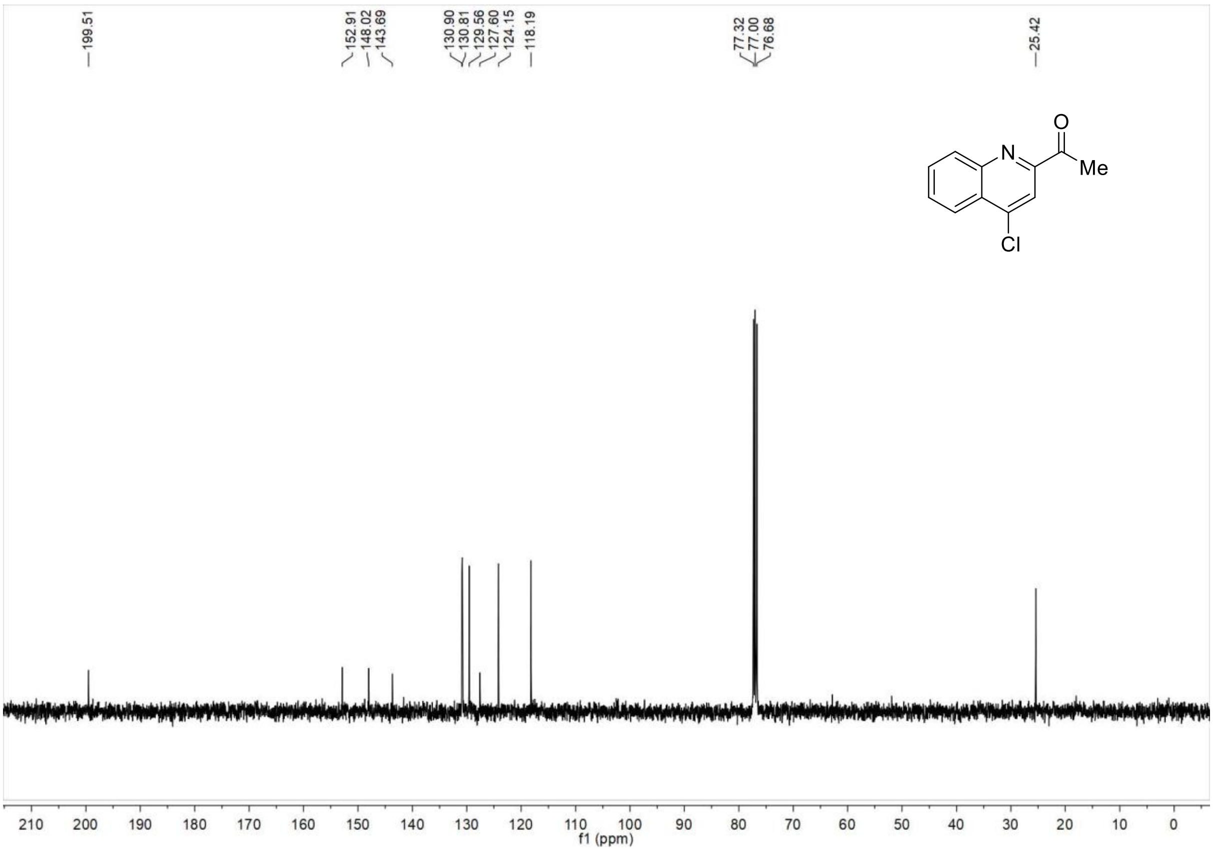

Supplementary Figure 156. ^{13}C-NMR of compound **43a**, recorded at 400 MHz and 25 °C in $CDCl_3$

1-(4-azidoquinolin-2-yl)Ethan-1-one (43b)

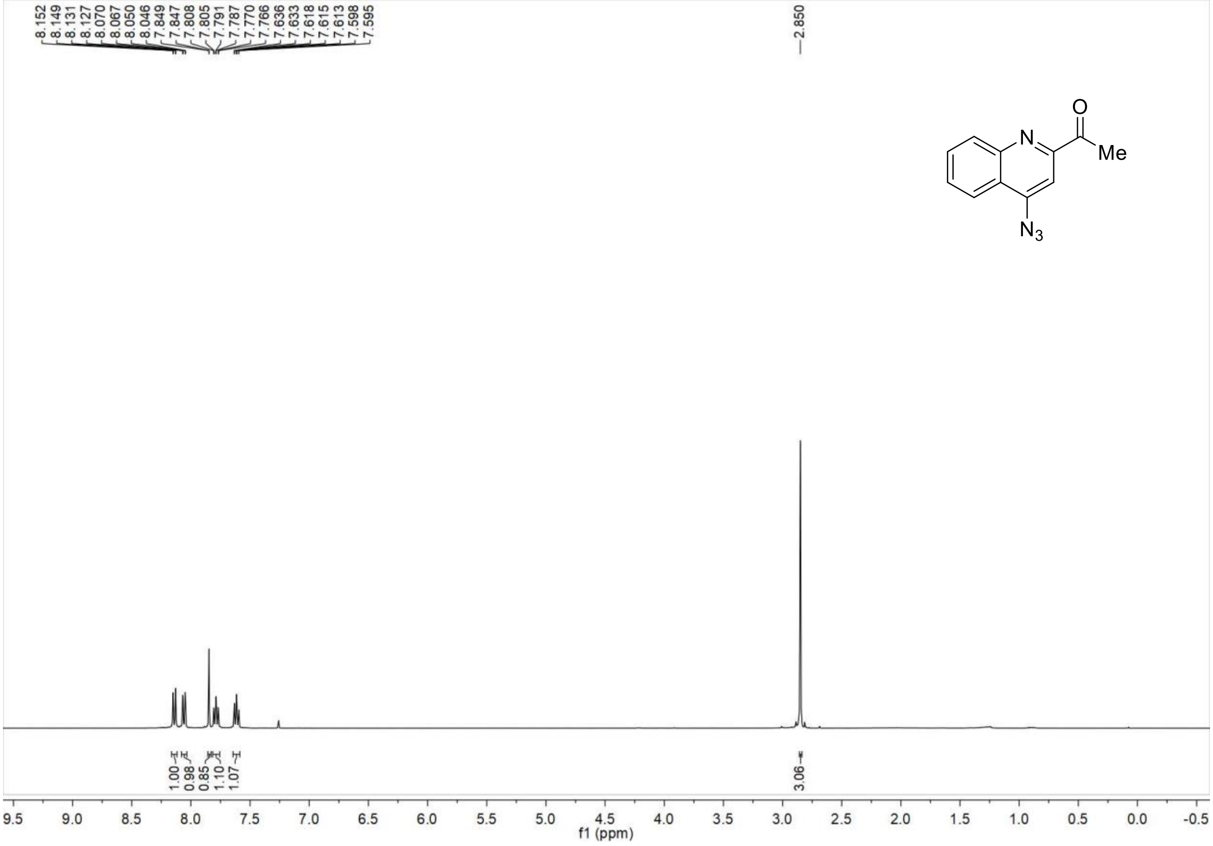

Supplementary Figure 157. ^{1}H-NMR of compound **43b** recorded at 400 MHz and 25 °C in $CDCl_3$

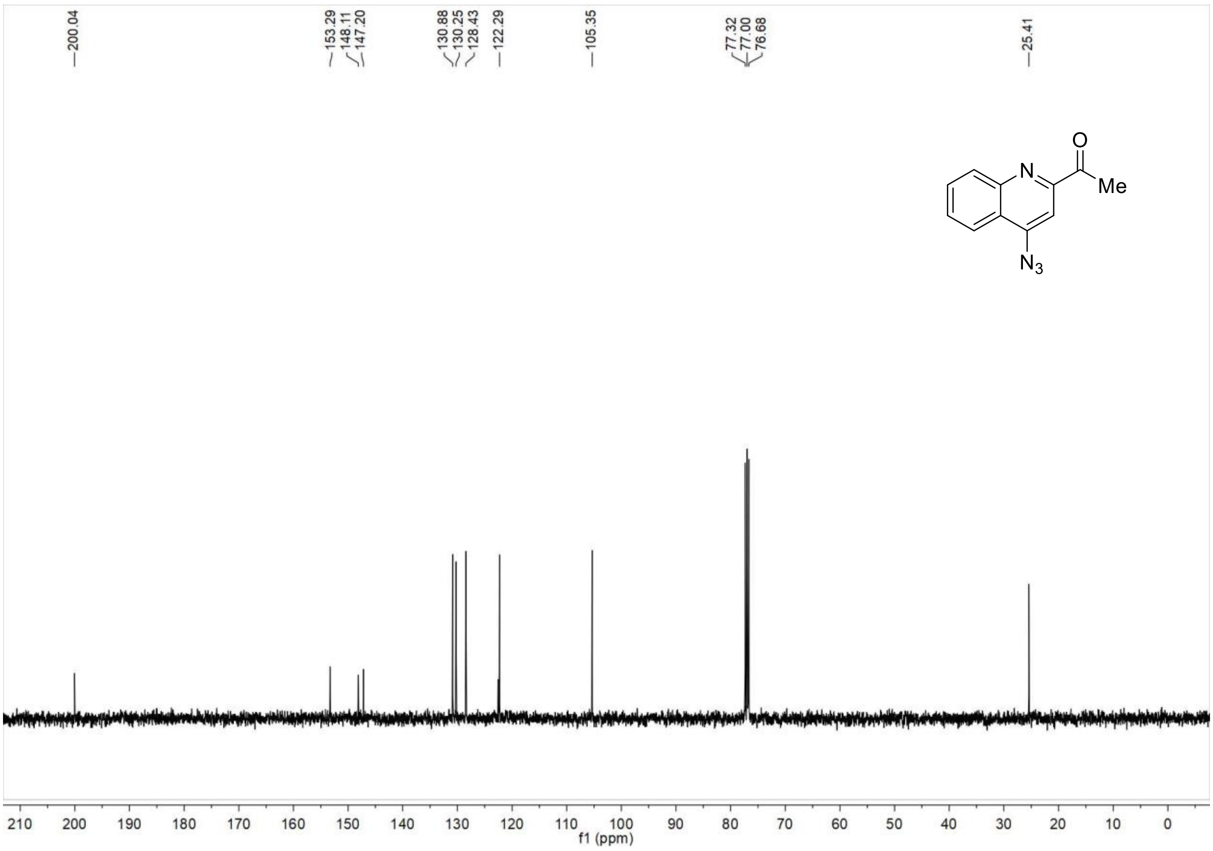

Supplementary Figure 158. ^{13}C-NMR of compound **43b**, recorded at 400 MHz and 25 °C in $CDCl_3$

1-(5-methoxy-1*H*-benzo[e][1,4]diazepin-2-yl)Ethan-1-one (**43**)

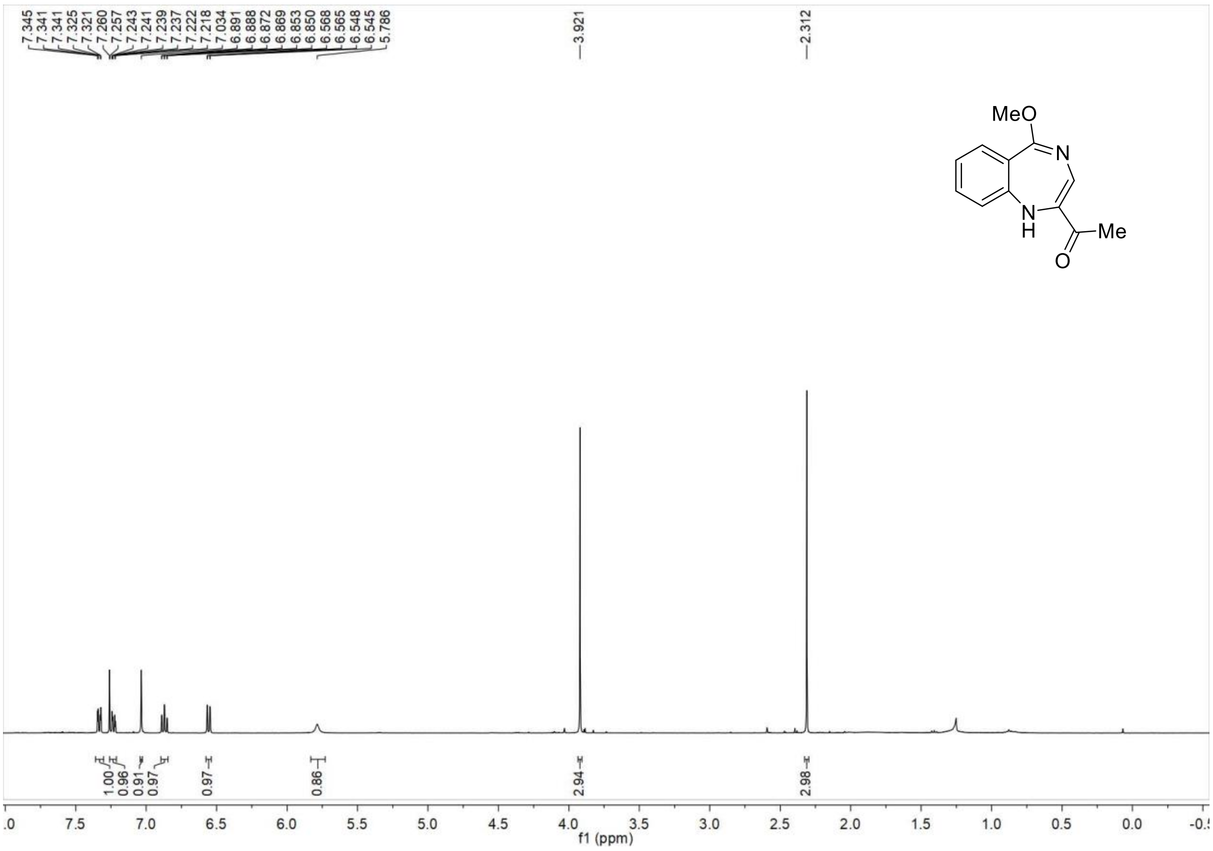

Supplementary Figure 159. ^{1}H-NMR of compound **43** recorded at 400 MHz and 25 °C in $CDCl_3$

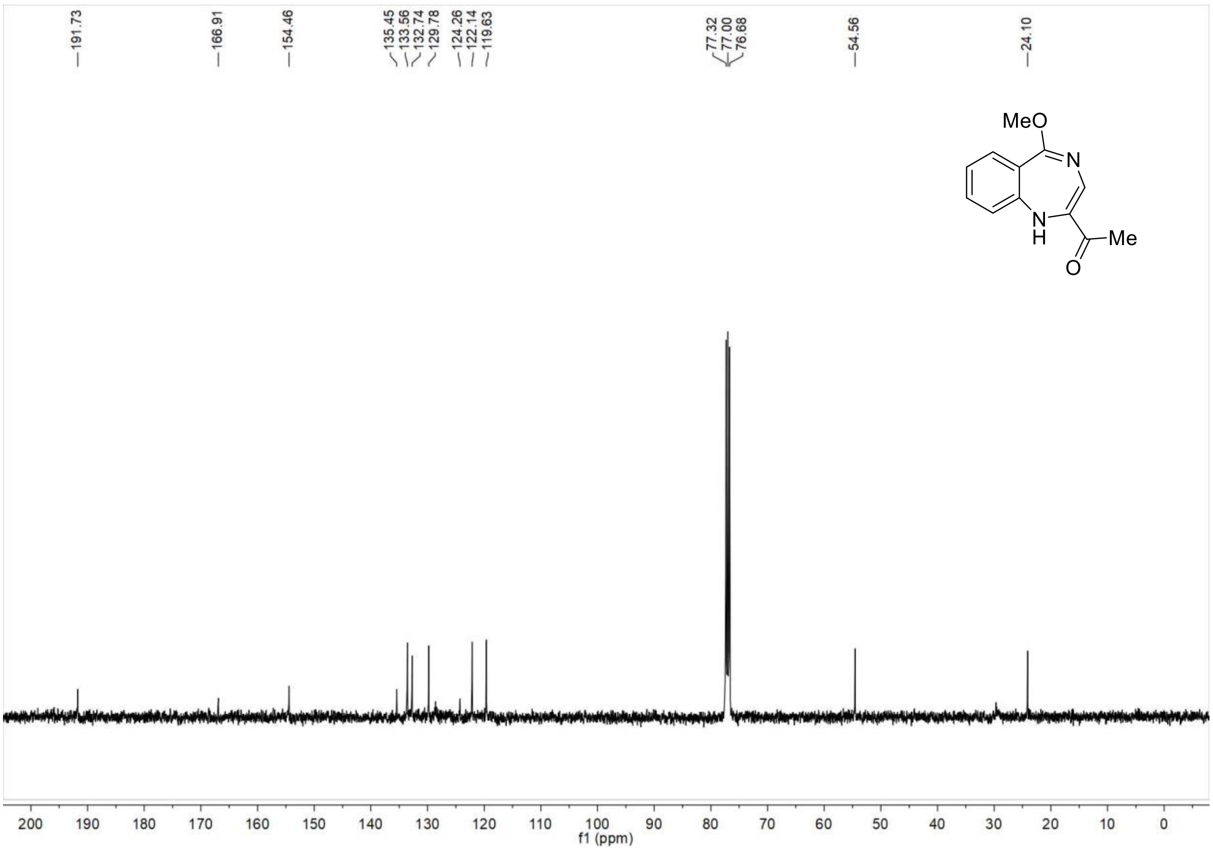

Supplementary Figure 160. ^{13}C-NMR of compound **43**, recorded at 400 MHz and 25 °C in $CDCl_3$

1-Azido-1-methylnaphthalen-2(1*H*)-one (44)

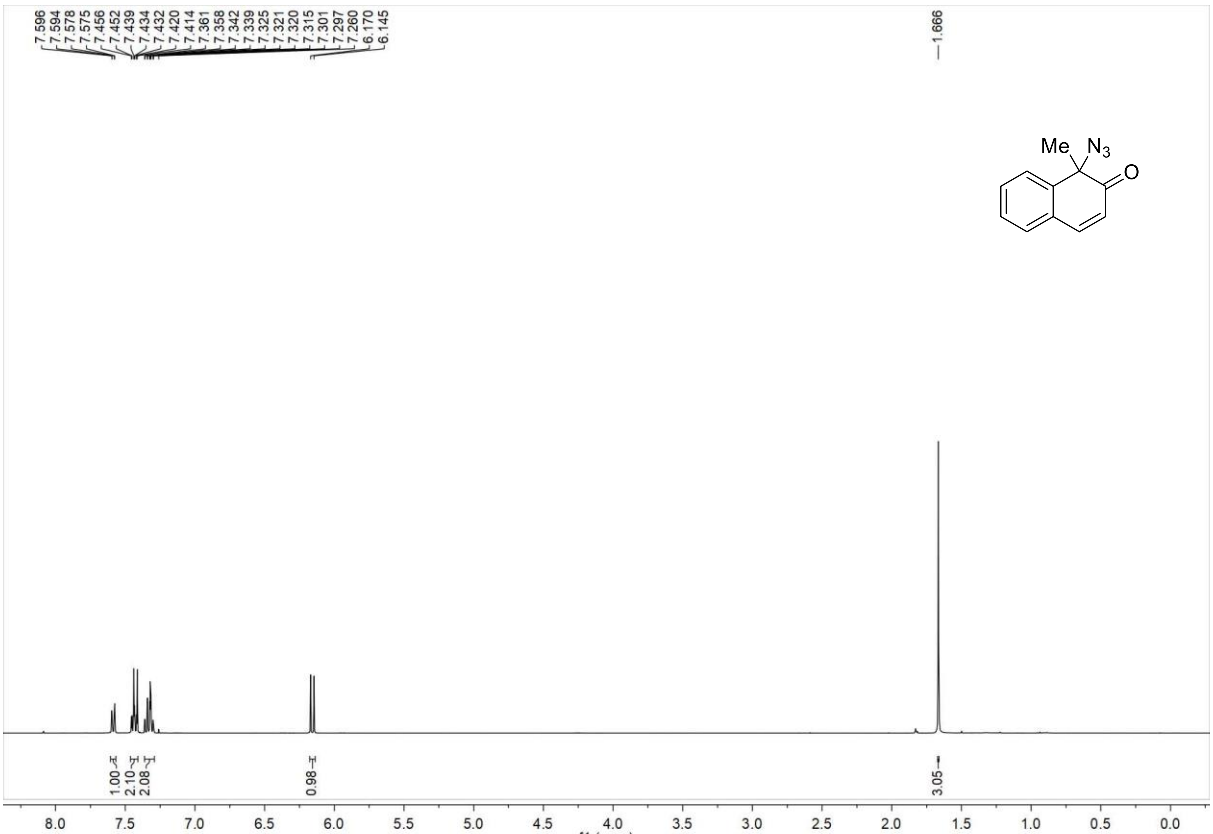

Supplementary Figure 161. ^{1}H-NMR of compound **44** recorded at 400 MHz and 25 °C in $CDCl_3$

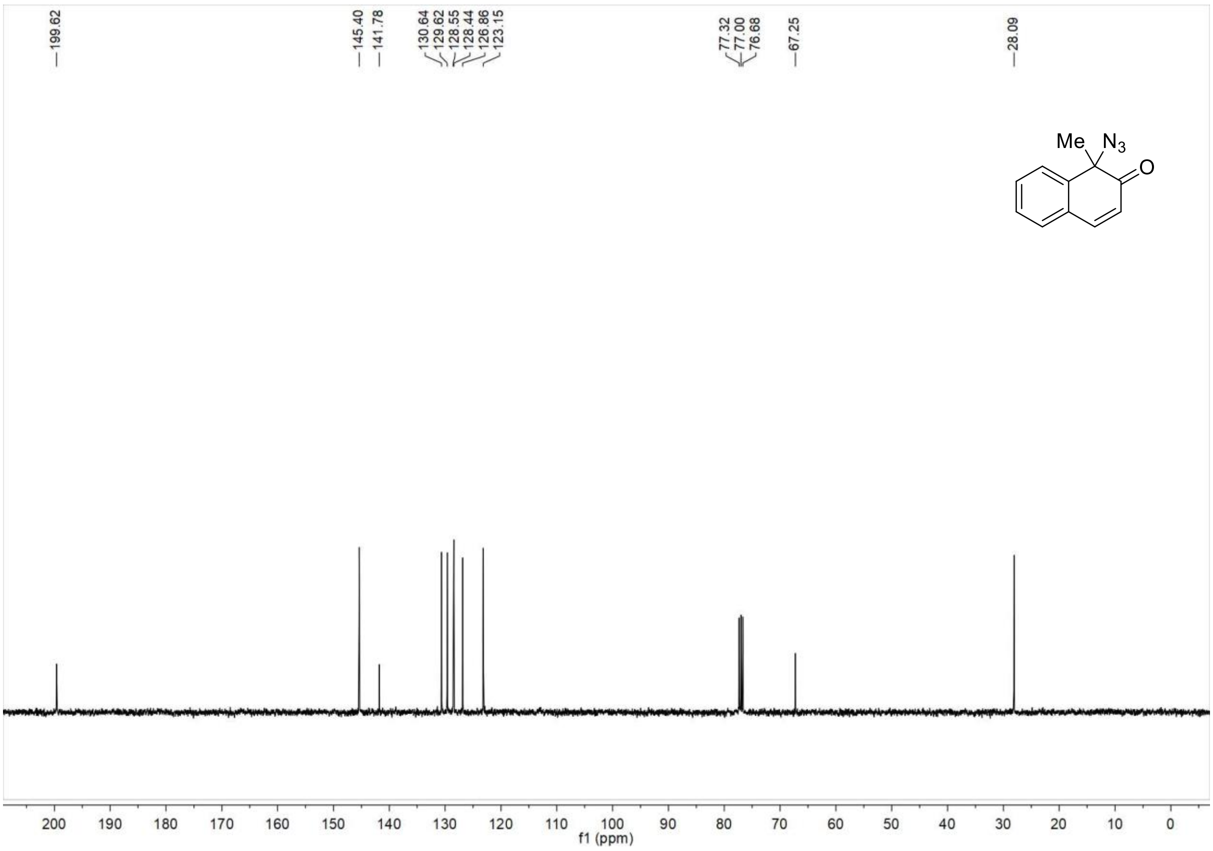

Supplementary Figure 162. ^{13}C-NMR of compound **44**, recorded at 400 MHz and 25 °C in $CDCl_3$

1',2'-Ddihydrospiro[benzo[*b*]azepine-2,3'-benzo[*f*]chromen]-3(1*H*)-one (45)

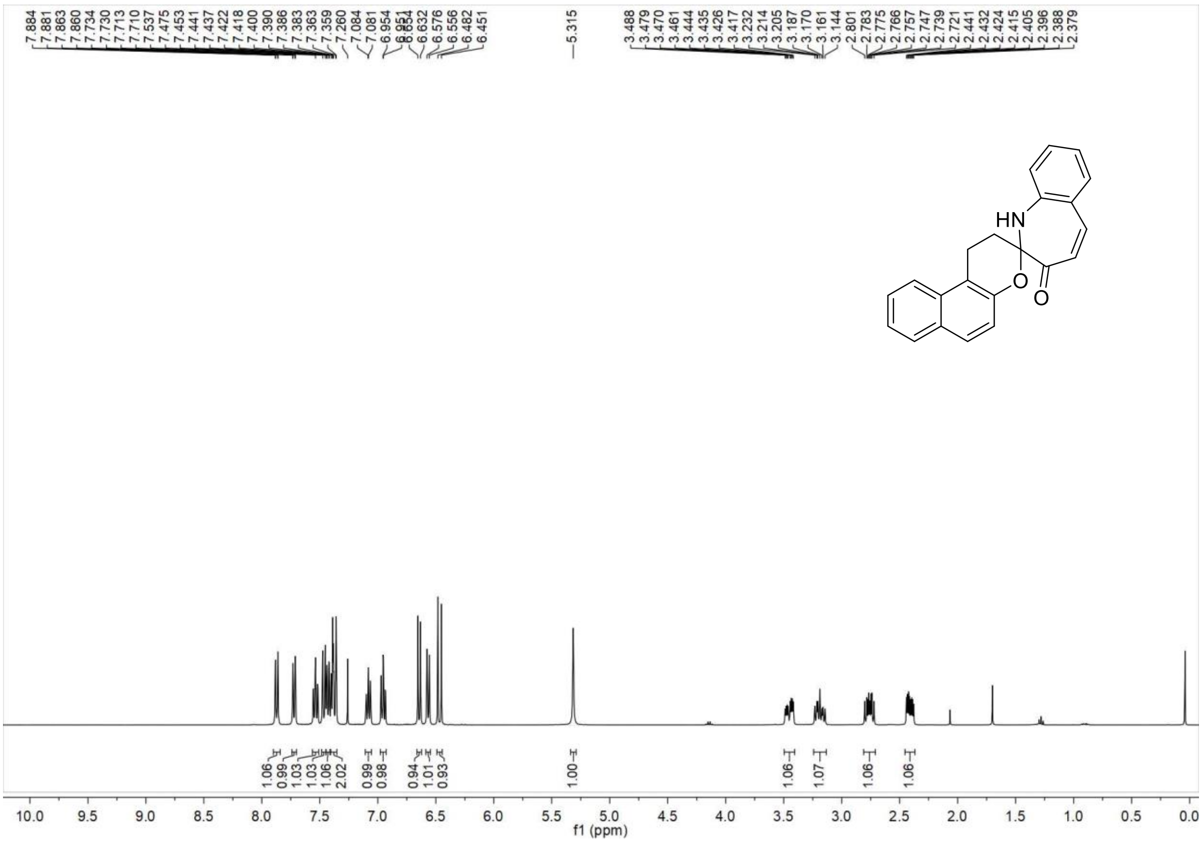

Supplementary Figure 163. ^{1}H-NMR of compound **45** recorded at 400 MHz and 25 °C in $CDCl_3$

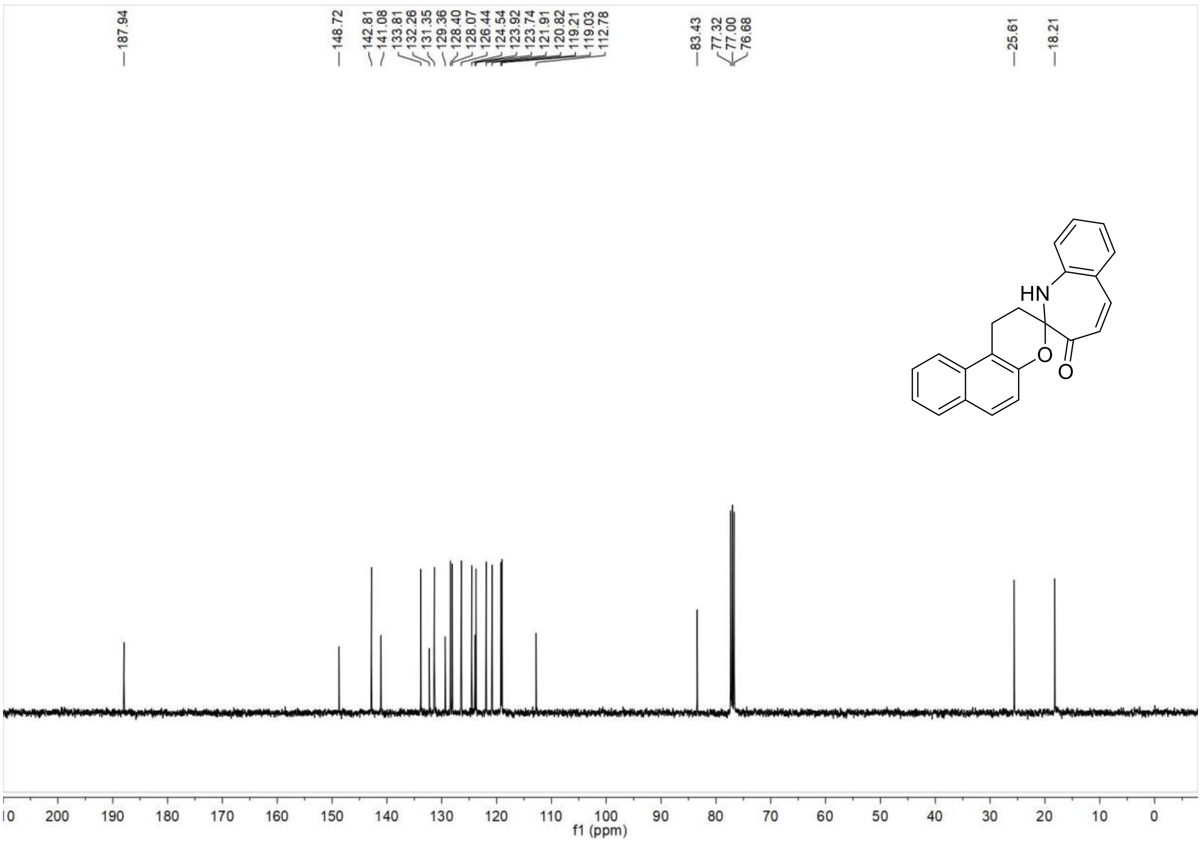

Supplementary Figure 164. ^{13}C-NMR of compound **45**, recorded at 400 MHz and 25 °C in $CDCl_3$

Phenyl(quinolin-2-yl)methanone

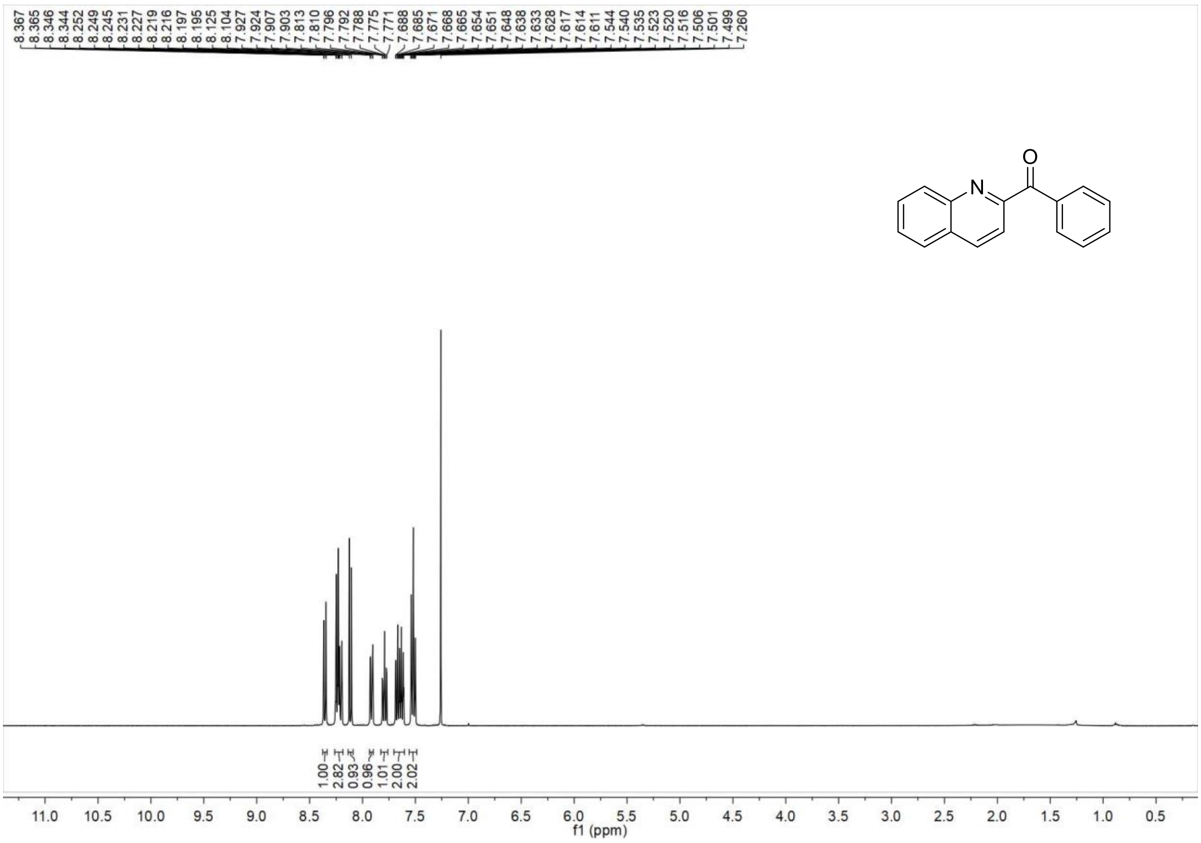

Supplementary Figure 165. ^{1}H-NMR of phenyl(quinolin-2-yl)methanone, recorded at 400 MHz and 25 °C in $CDCl_3$

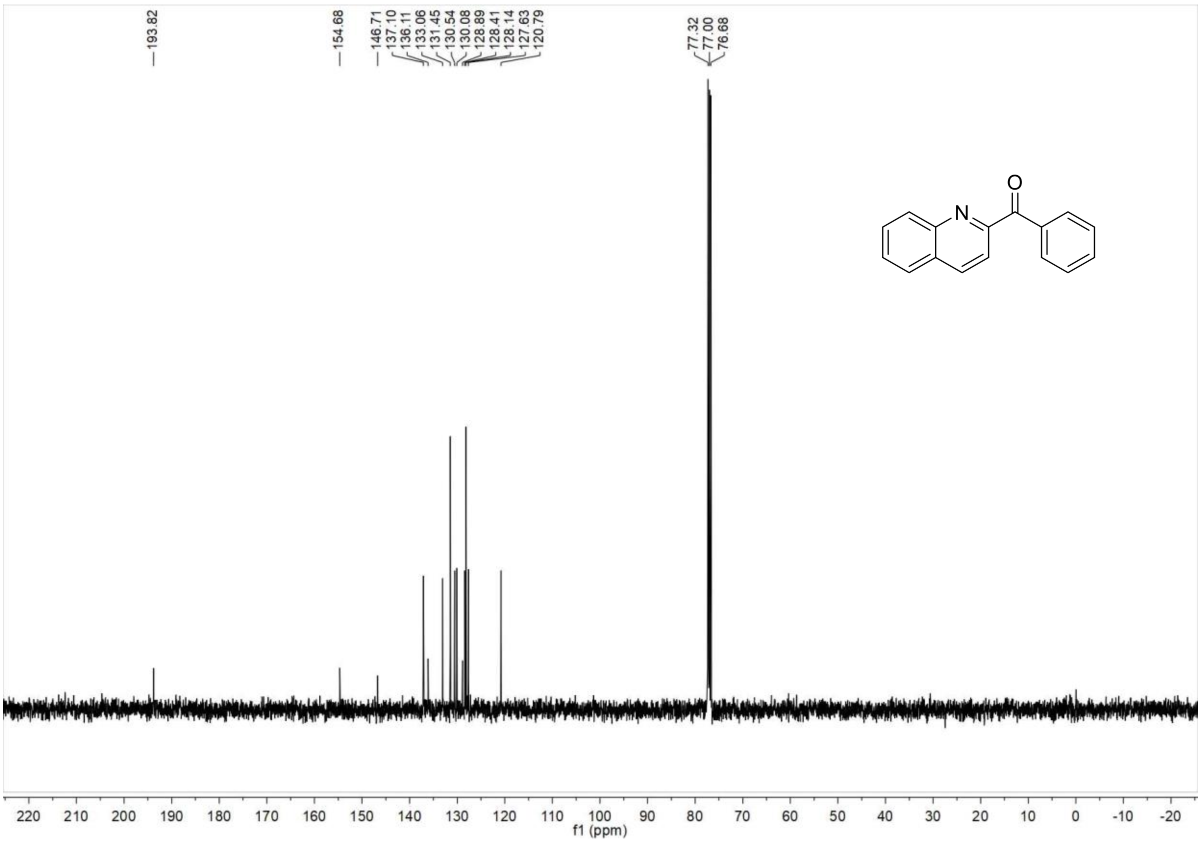

Supplementary Figure 166. ^{13}C-NMR of phenyl(quinolin-2-yl)methanone, recorded at 400 MHz and 25 °C in $CDCl_3$

Copies of ^{19}F spectra

3-Fluoro-10-methylphenanthren-9-ol (7a)

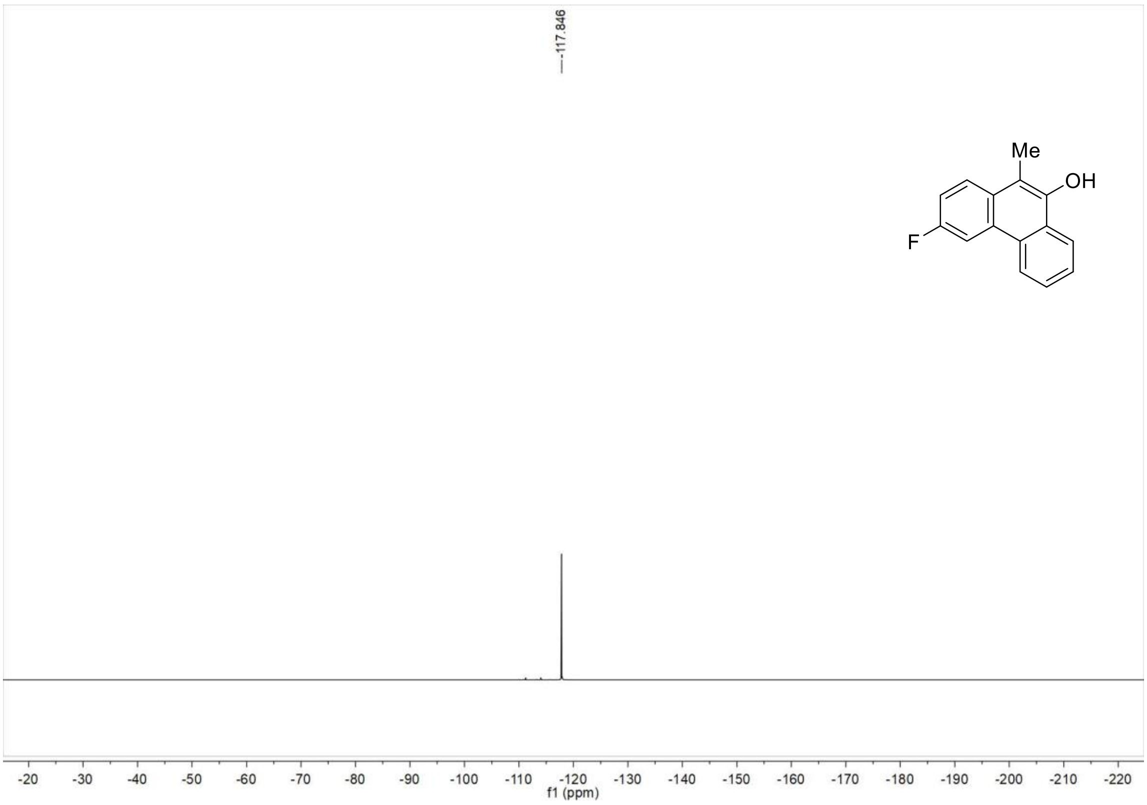

Supplementary Figure 167. ^{19}F-NMR of compound **7a**, recorded at 376 MHz and 25 °C in $CDCl_3$

10-Methyl-7-(trifluoromethyl)phenanthren-9-ol (10a)

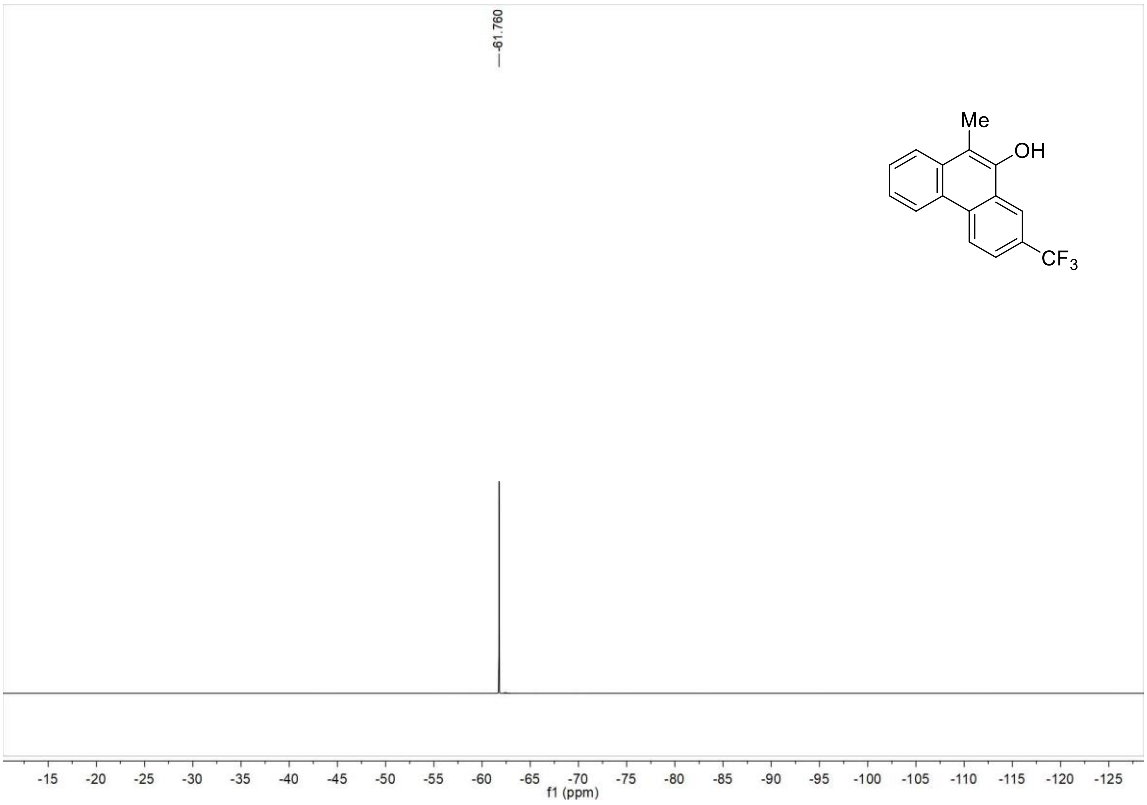

Supplementary Figure 168. ^{19}F-NMR of compound **10a**, recorded at 376 MHz and 25 °C in $CDCl_3$

1-(2-fluorophenanthridin-6-yl)Ethan-1-one (7)

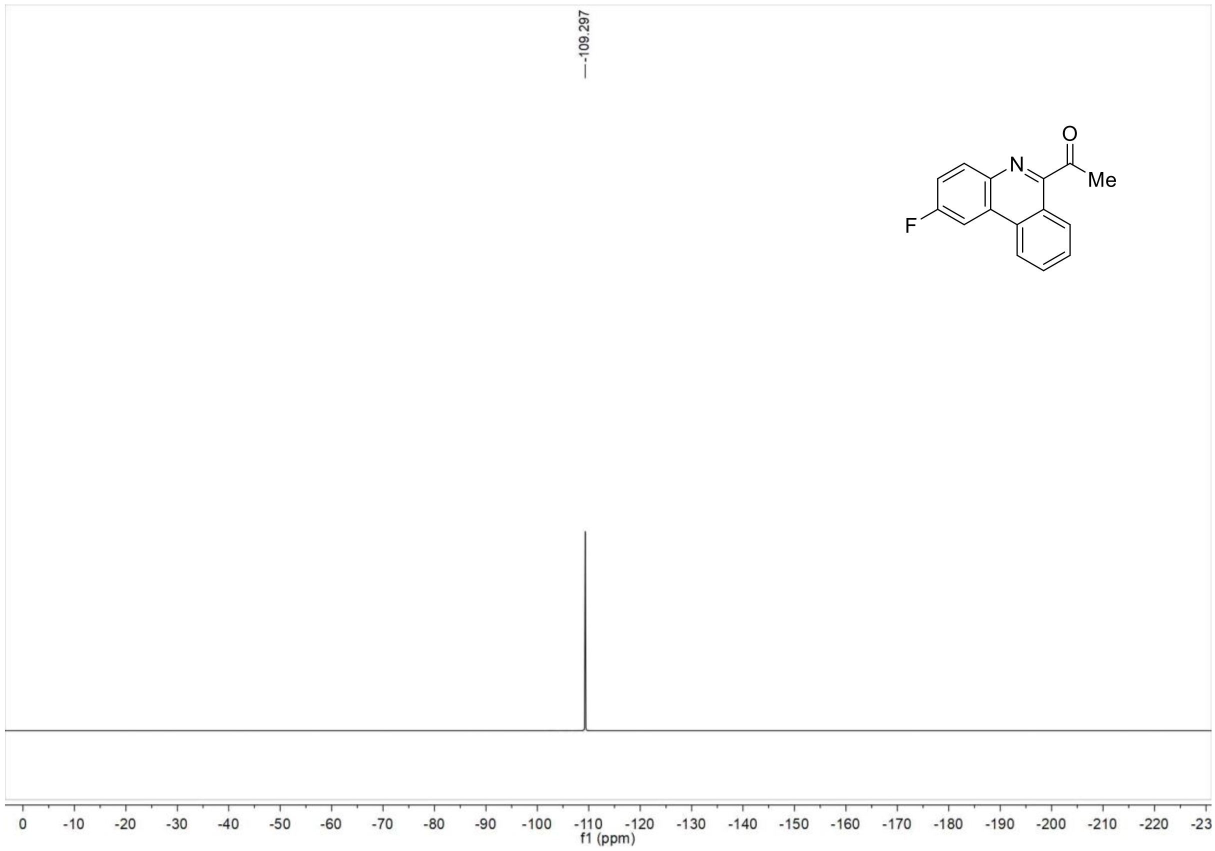

Supplementary Figure 169. ^{19}F-NMR of compound **7**, recorded at 376 MHz and 25 °C in CDCl3

1-(8-(trifluoromethyl)phenanthridin-6-yl)Ethan-1-one (10)

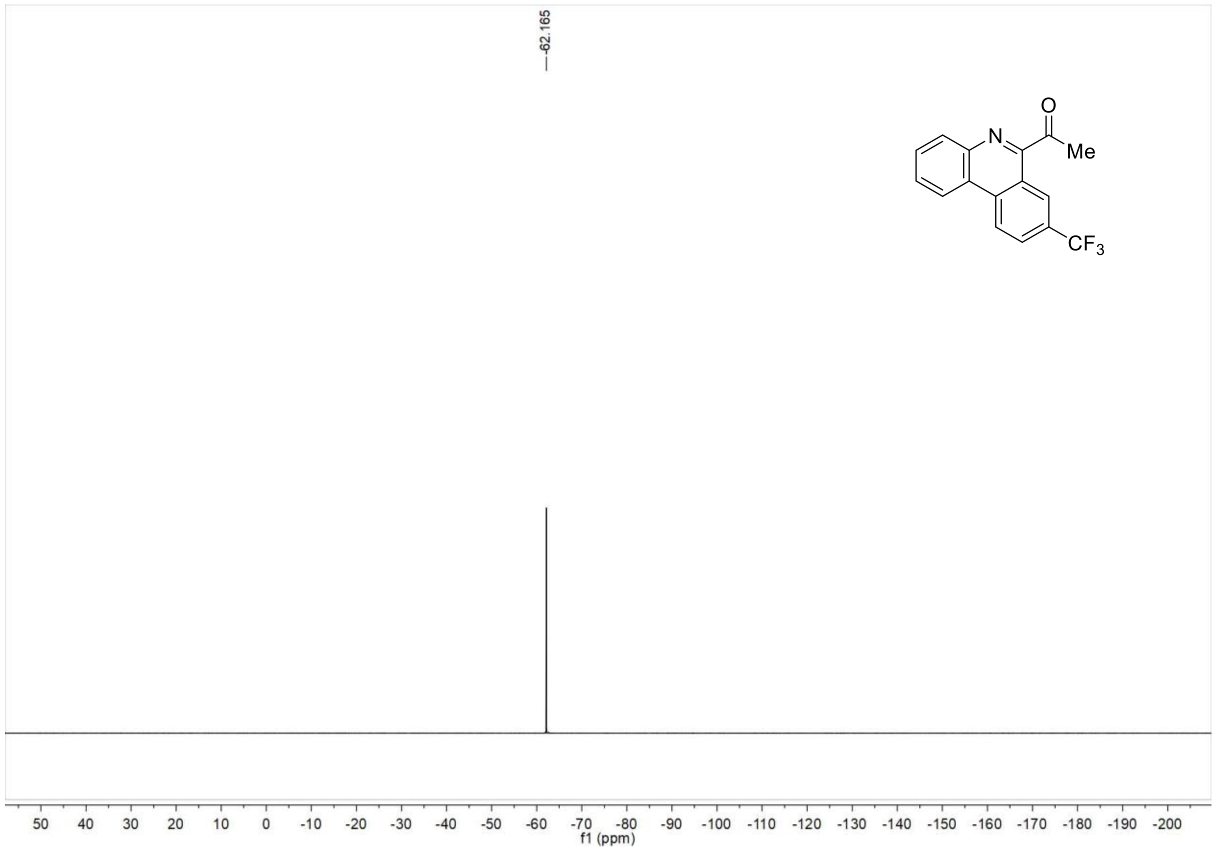

Supplementary Figure 170. ^{19}F-NMR of compound **10**, recorded at 376 MHz and 25 °C in CDCl3

4. References

1. Xia, Y. et al. Catalyst-Free Intramolecular Formal Carbon Insertion into σ-C C Bonds: A New Approach toward Phenanthrols and Naphthols. *Angew. Chem. Int. Ed.* **52**, 2543–2546 (2013).
2. Oguma, T. & Katsuki, T. Iron-Catalyzed Dioxygen-Driven C–C Bond Formation: Oxidative Dearomatization of 2-Naphthols with Construction of a Chiral Quaternary Stereocenter *J. Am. Chem. Soc.* **134**, 20017–20020 (2012).
3. Nan, J. et al. Direct Asymmetric Dearomatization of 2-Naphthols by Scandium-Catalyzed Electrophilic Amination. *Angew. Chem. Int. Ed.* **54**, 2356–2360 (2015).
4. Grandclaudon, C. & Toullec, P. Y. Phase-Transfer-Catalyzed Oxazirid-ine-Mediated Hydroxylative Phenol and Naphthol Dearomatization. *Eur. J. Org. Chem.* **2016**, 260–264 (2016).
5. Kaku, T. et al. 17,20-Lyase inhibitors. Part 4: Design, synthesis and structure–activity relationships of naphthylmethylimidazole derivatives as novel 17,20-lyase inhibitors. *Bioorgan. Med. Chem.* **19**, 1751–1710 (2011).
6. Harvey, R. G., Dai, Q., Ran, C. T. & Penning, M. Synthesis of the o-Quinones and Other Oxidized Metabolites of Polycyclic Aromatic Hydrocarbons Implicated in Carcinogenesis. *J. Org. Chem.* **2004**, *69*, 2024–2032.
7. Dhineshkumar, J., Samaddar, P. & Prabhu, K. R. A copper catalyzed azidation and peroxidation of β-naphthols via an oxidative dearomatization strategy. *Chem. Commun.* **52**, 11084–11087 (2016).
8. Sarkar, D., Ghosh, M. K. Rout, N. & Kuila, P. "A Jack of Trio"-robust one-pot metal free oxidative amination, azidation and peroxidation of phenols. *New J. Chem.* **2017**, *41*, 3715–3718.
9. Harschneck, T., Hummel, S., Kirsch, S. F. & Klahn, P. Practical Azidation of 1,3-Dicarbonyls. *Chem. Eur. J.* **18**, 1187–1193 (2012).
10. Feng, S. et al. Catalytic Asymmetric [4 + 2] Cycloaddition of ortho-Alkenyl Naphthols/Phenols with ortho-Quinone Methides: Highly Stereoselective Synthesis of Chiral 2,3,4-Trisubstituted Chromans. *J. Org. Chem.* **85**, 5231−5244 (2020).
11. Patonay, T., Micskei, K., Juhász-Tóth, É., Fekete, S. & Pardi-Tóth, V. C. α-Azido ketones, Part 6.† Reduction of acyclic and cyclic α-azido ketones into α-amino ketones: old problems and new solutions. *ARKIVOC* 270−290 (2009).
12. Ardiansah, B., Tanimoto, H., Tomohiro, T., Morimoto, Y. & Kakiuchi, K. Sulfonium ion-promoted traceless Schmidt reaction of alkyl azide. *Chem. Commun.* **57**, 8738−8741 (2021).
13. Wu, W. et al. One-pot regioselective synthesis of 2,4-disubstituted quinolines via copper(ii)-catalyzed cascade annulation. *Org. Chem. Front.* **5**, 1713−1718 (2018).
14. Sashida, Kaname, H. M. & Tsychiya,T. Studies of Seven-Membered Heterocycles. XXXII. : Synthesis of N-Unsubstituted 1H-1, 4-Benzodiazepines Stabilized by Intramolecular Hydrogen Bonding. *Chem. Pharm. Bull.* **38**, 2919–2925 (1990).
15. Laha, J. K., Tinwala, U. & Hunjan, M. K. Minisci aroylation of *N*-heterocycles using choline persulfate in water under mild conditions. *New J. Chem.* **45**, 22853–22859 (2021).
